# Genome-Wide Identification and Evolution of HECT Genes in Soybean

**DOI:** 10.3390/ijms16048517

**Published:** 2015-04-16

**Authors:** Xianwen Meng, Chen Wang, Siddiq Ur Rahman, Yaxu Wang, Ailan Wang, Shiheng Tao

**Affiliations:** 1 College of Life Sciences and State Key Laboratory of Crop Stress Biology in Arid Areas, Northwest A&F University, Yangling 712100, China; E-Mails: mxw68@nwsuaf.edu.cn (X.M.); jiafei4321@gmail.com (C.W.); siddiqbiotec88@gmail.com (S.U.R.); yaxuwang@nwsuaf.edu.cn (Y.W.); wangailan@nwsuaf.edu.cn (A.W.); 2 Bioinformatics Center, Northwest A&F University, Yangling 712100, China

**Keywords:** soybean, HECT genes, evolution, segmental duplication

## Abstract

Proteins containing domains homologous to the E6-associated protein (E6-AP) carboxyl terminus (HECT) are an important class of E3 ubiquitin ligases involved in the ubiquitin proteasome pathway. HECT-type E3s play crucial roles in plant growth and development. However, current understanding of plant HECT genes and their evolution is very limited. In this study, we performed a genome-wide analysis of the HECT domain-containing genes in soybean. Using high-quality genome sequences, we identified 19 soybean HECT genes. The predicted HECT genes were distributed unevenly across 15 of 20 chromosomes. Nineteen of these genes were inferred to be segmentally duplicated gene pairs, suggesting that in soybean, segmental duplications have made a significant contribution to the expansion of the HECT gene family. Phylogenetic analysis showed that these HECT genes can be divided into seven groups, among which gene structure and domain architecture was relatively well-conserved. The Ka/Ks ratios show that after the duplication events, duplicated HECT genes underwent purifying selection. Moreover, expression analysis reveals that 15 of the HECT genes in soybean are differentially expressed in 14 tissues, and are often highly expressed in the flowers and roots. In summary, this work provides useful information on which further functional studies of soybean HECT genes can be based.

## 1. Introduction

The ubiquitin-proteasome system (UPS) plays a crucial role in plant growth, development, and response to environmental stress [1,2,3,4,5,6,7]. The ubiquitination pathway consists of an enzymatic cascade mediated by three sequential enzymes: E1 ubiquitin activating enzyme (E1), E2 ubiquitin conjugating enzyme (E2), and E3 ubiquitin ligase (E3) [8,9,10,11]. During the ubiquitination process, the specificity of the selective proteolysis by UPS is usually determined by E3s, which targets substrate proteins with different substrate recognition domains for ubiquitylation [4,12]. In plants, E3s can be classified into three main types according to differences in their action mechanisms, and the presence of specific domains [13,14,15,16,17,18,19,20]: homologous to the E6-associated protein (E6-AP) carboxyl terminus (HECT), really interesting new gene (RING), and U-box.

The HECT ubiquitin ligase is an important class of E3 enzymes. HECT E3s are single polypeptides characterized by the presence of a *C*-terminal 350-amino acid-length HECT domain. The common features of HECT E3s are the *C*-terminal catalytic HECT domain, and the *N*-terminal domains, which recruit specific substrates for ubiquitin ligation [7,12]. The *C*-terminal HECT domain includes two essential binding sites: a ubiquitin-binding site, and an E2-binding site [7,12]. It also includes two sub-structures: the *C*-lobe, which receives ubiquitin from E2 and links itself with ubiquitin, and the *N*-lobe [21]. Classification of a particular HECT E3 protein into one of the different subfamilies is based on the arrangement of the *N*-terminal domains [7,22,23]. These two modular architectures, the *N*-terminal substrate-binding domains and the *C*-terminal HECT domain, govern the polypeptides’ interactions with various substrates, as well as their regulatory functions. Substrates often contain recognition sequences, which can bind directly to the *N*-terminal substrate-binding domains [21,24,25,26,27]. The unique HECT domains are crucial to the identification and evolution of the HECT genes in plant genomes, and merit intensive research.

As the smallest E3 subfamily, HECT comprises seven genes (named *UPL1*–*UPL7*), which have been identified in *Arabidopsis thaliana* [7]. Recently, 413 plant sequences containing the HECT domain were identified via TBlastN analysis, which compared multiple HECT sequences to entries in the NCBI database [22]. However, due to the lack of corresponding data from other genomes, the process of identifying HECT genes in other plant species is not complete. Although a genomic survey of eukaryote HECT ubiquitin ligases was performed, the number plant of species included in the research was limited [23]. The plant species with fully analyzed HECT genes is *Arabidopsis thaliana* [3,6,7]. In this study, we performed a genome-wide analysis of the HECT domain-containing genes in soybean, ultimately identifying 19 HECT genes. We also performed a comprehensive phylogenetic analysis of 365 HECT genes from 41 plant species. These 365 HECT genes included the 19 soybean HECT genes and a subset of HECT genes from four plant species, including *Arabidopsis thaliana*, *Glycine max*, *Medicago truncatula*, and *Phaseolus vulgaris*. A detailed analysis of gene structure, domain architecture, chromosome location, duplication pattern, and expression pattern was performed. It is interesting to note that all 19 soybean HECT genes are located in the duplicated blocks of the genome, which suggests that segmental duplications have made crucial contributions to the expansion of HECT genes in this plant species. Moreover, we used the RNA-seq expression profiles of 14 soybean tissues to study the expression patterns of the different HECT genes. Our work provides information that is useful for further investigation of the various functions of the HECT gene family in soybean.

## 2. Results

### 2.1. Identification of Homologous to the E6-Associated Protein (E6-AP) Carboxyl Terminus (HECT) Gene Family in Soybean

The HECT genes, characterized by the existence of the HECT domain, have previously been analyzed in *Arabidopsis thaliana* [7]. In this study, a total of 365 putative HECT genes (Figure S1) were identified, using a combined approach HMMER–Blast–InterProScan of the 41 plant genomes in Phytozome v9.1 [28] (Tables S1 and S2), including the 19 soybean HECT genes (Table 1), and 41 HECT genes from three legume species: *Glycine max* (19), *Medicago truncatula* (10), and *Phaseolus vulgaris* (12). Seven *Arabidopsis thaliana* HECT genes (*AT1G55860*/*UPL1*, *AT1G70320*/*UPL2*, *AT3G17205*/*UPL6*, *AT3G53090*/*UPL7*, *AT4G12570*/*UPL5*, *AT4G38600*/*UPL3* and *AT5G02880*/*UPL4*) were verified by applying our methods to the *Arabidopsis thaliana* genome sequence database in TAIR10.

**Table 1:** The information relating to 19 homologous to the E6-associated protein (E6-AP) carboxyl terminus (HECT) genes in the soybean genome.

Gene Symbol	Gene Locus	Chromosome	Gene Start	Gene Stop	Amino Acids
*Gma01*	*Glyma02g38020*	2	43347265	43364774	3649
*Gma02*	*Glyma03g34650*	3	42000995	42011419	973
*Gma03*	*Glyma04g00530*	4	285772	296292	1891
*Gma04*	*Glyma04g10481*	4	8701971	8719496	3680
*Gma05*	*Glyma05g26360*	5	32340858	32357248	3762
*Gma06*	*Glyma06g00600*	6	309849	320018	1895
*Gma07*	*Glyma06g10360*	6	7845196	7861448	3654
*Gma08*	*Glyma07g36390*	7	41782618	41798454	1026
*Gma09*	*Glyma07g39546*	7	44005949	44011941	867
*Gma10*	*Glyma08g09270*	8	6626148	6642483	3749
*Gma11*	*Glyma10g05620*	10	4408645	4417572	1557
*Gma12*	*Glyma11g11490*	11	8185583	8196786	1872
*Gma13*	*Glyma12g03640*	12	2443609	2454729	1877
*Gma14*	*Glyma13g19981*	13	23464333	23472965	1558
*Gma15*	*Glyma14g36180*	14	45377087	45394472	3652
*Gma16*	*Glyma15g14591*	15	11013042	11048953	1031
*Gma17*	*Glyma17g01210*	17	704329	710650	867
*Gma18*	*Glyma17g04180*	17	2781543	2800188	1026
*Gma19*	*Glyma19g37310*	19	44504837	44515898	1157

### 2.2. Phylogenetic Analysis of HECT Genes in Soybean

**Figure 1:**
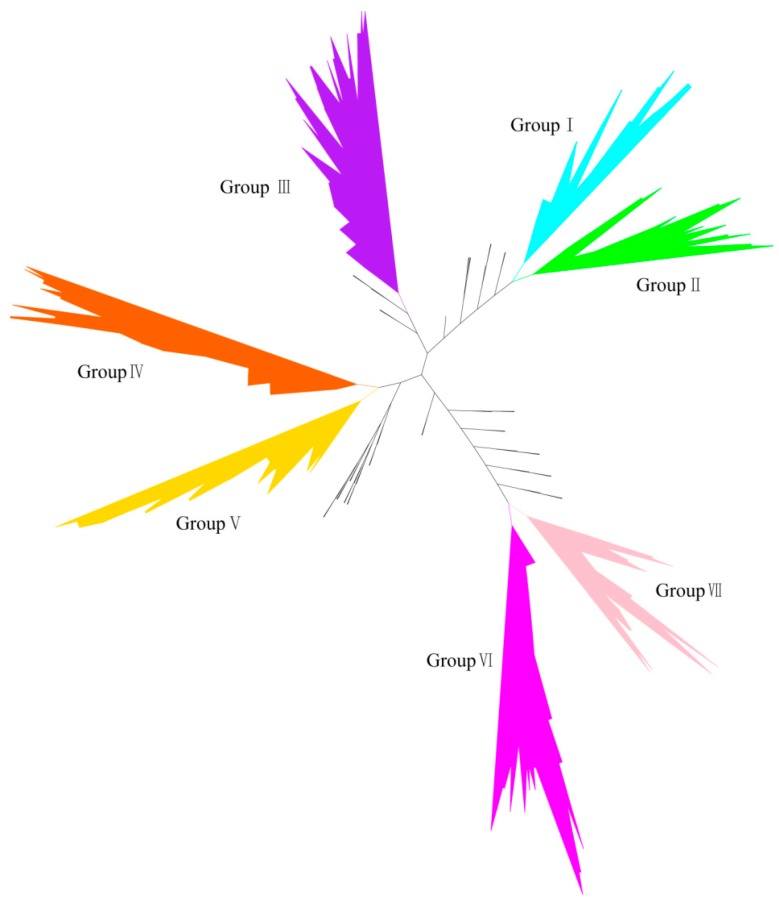
Phylogenetic relationships of 365 plant homologous to E6-associated protein (E6-AP) carboxyl terminus (HECT) genes. The maximum likelihood unrooted tree is shown, and the main branches corresponding to the seven groups are indicated with different colors.

To determine the nature of the evolutionary relationship between soybean HECT genes and those of other plant species, we performed multiple sequence alignments, and constructed a maximum likelihood phylogenetic tree for the 365 plant HECT proteins of the 41 plant species in Phytozome v9.1, including the 19 soybean HECT genes. The conserved HECT domain sequences (File S1) (about 350 amino acids in length) were used in the analysis, because of the different lengths and various domain architectures of the HECT proteins. Three hundred and sixty-five plant HECT genes from Viridiplantae can be classified into seven groups (Group I–VII), with the exception of some genes from the lower land plants (Figures 1 and S2). These seven groups can be further grouped into five subfamilies corresponding to those described in a previous study [22].

**Figure 2:**
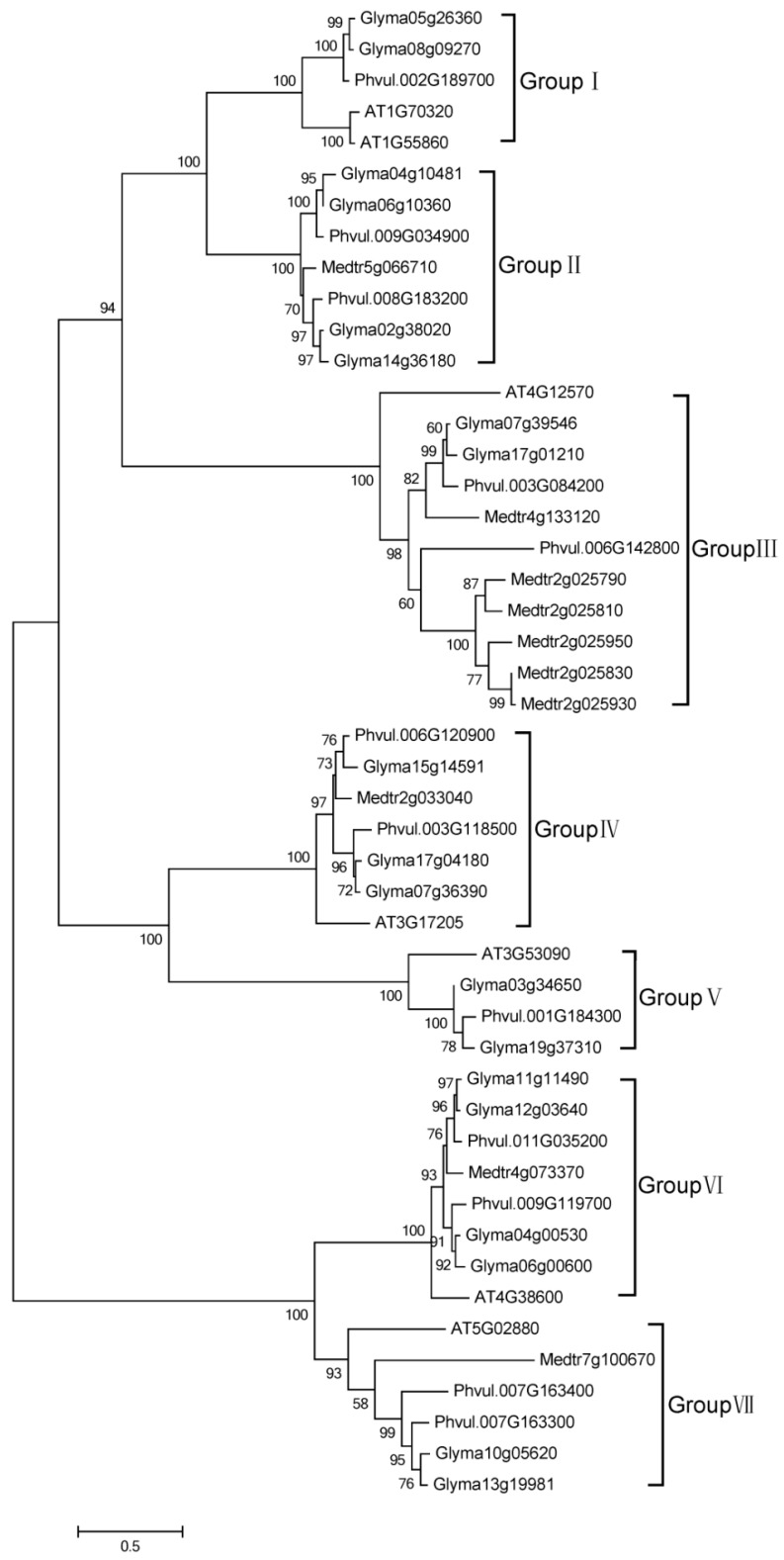
Neighbor-joining (NJ) tree of HECT genes from *Glycine max*, *Medicago truncatula*, *Phaseolus vulgaris*, and *Arabidopsis thaliana*. MEGA6 package was used to construct the NJ tree from the full-length amino acid sequence alignments (File S2) of the four plant species, with 1000 bootstrap replicates. Numbers refer to bootstrap support (in terms of percentage).

**Figure 3:**
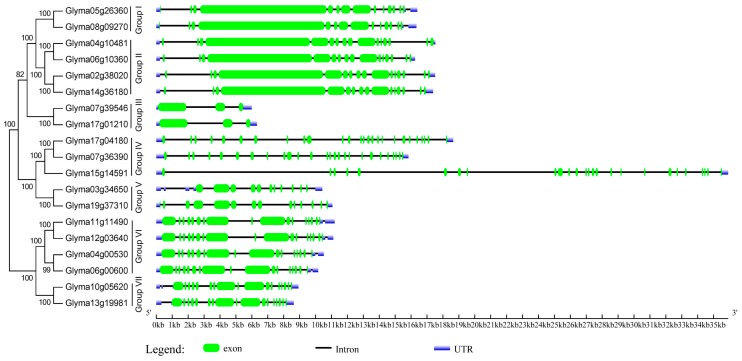
Phylogenetic relationships and exon/intron structures of HECT proteins in soybean. The unrooted neighbor-joining tree was constructed via the alignment of full-length amino acid sequences (File S3), using the MEGA6 package. Lengths of the exons and introns of each HECT gene are displayed proportionally. The green boxes, blue boxes, and lines indicate exons, untranslated regions (UTRs), and introns, respectively.

**Figure 4:**
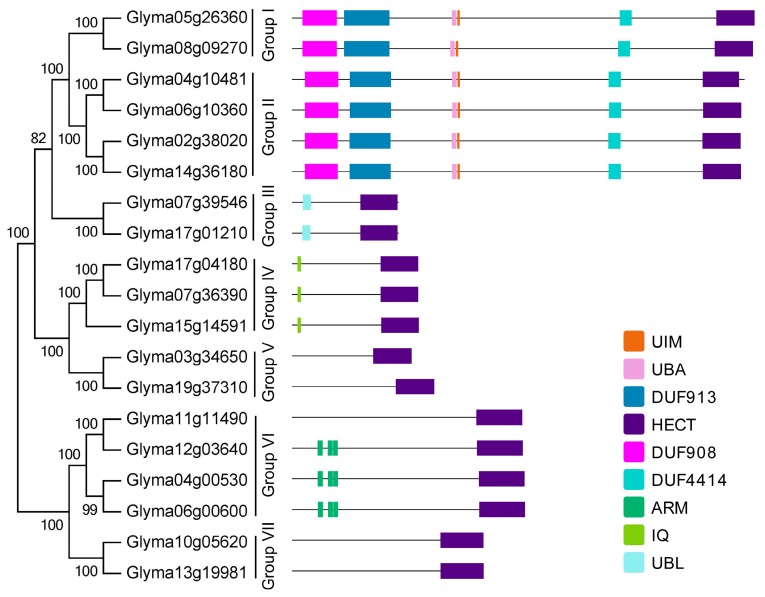
Domain architectures of soybean HECT proteins according to phylogenetic relationships. Each domain is represented by a colored box. UIM: Ubiquitin-interacting motif; UBA: Ubiquitin associated domain; DUF: Domain of unknown function; ARM: Armadillo repeats; IQ: IQ short calmodulin-binding motif; UBL: Ubiquitin like domain.

To further examine the evolutionary characteristics of soybean HECT genes, the phylogenetic relationships of the full-length HECT proteins of *Glycine max*, *Medicago truncatula*, *Phaseolus vulgaris*, and *Arabidopsis thaliana* (outgroup) were analyzed. As shown in Figure 2, *Arabidopsis* HECT genes are consistently separated from those of other species. The 19 soybean HECT genes can also be subdivided into these seven groups (Figure 2, Figure 3 and Figure 4). In soybean, groups I, III, V, and VII each contain two genes, groups II and VI each contain four genes, and group IV contains three genes. However, in *Arabidopsis thaliana*, groups III–VII each contain only one gene, Group I contains two genes as in soybean, and Group II does not contain any HECT genes.

### 2.3. Domain Architecture and Exon-Intron Structure of the Soybean HECT Genes

To better understand the structural diversity of HECT genes, the exon-intron structures of the soybean HECT genomic sequences, and the domain architectures of the soybean HECT proteins were compared, according to their phylogenetic relationships. Each gene structure was obtained by comparing its coding sequences to its genomic sequences. As shown in Figure 3, closely related HECT genes were generally more similar in gene structure, particularly with respect to exon and intron number, and differed mainly in their respective exon and intron lengths. The domain architecture of HECT proteins was analyzed using the InterProScan program with a six-database annotation. A total of nine domains were identified (Figure 4). In addition to the HECT domain, soybean HECT proteins contain additional domains in the *N*-terminal regions, which are assumed to be responsible for governing interactions with various substrates [7].

### 2.4. Chromosome Location and Duplication of Soybean HECT Genes

**Figure 5:**
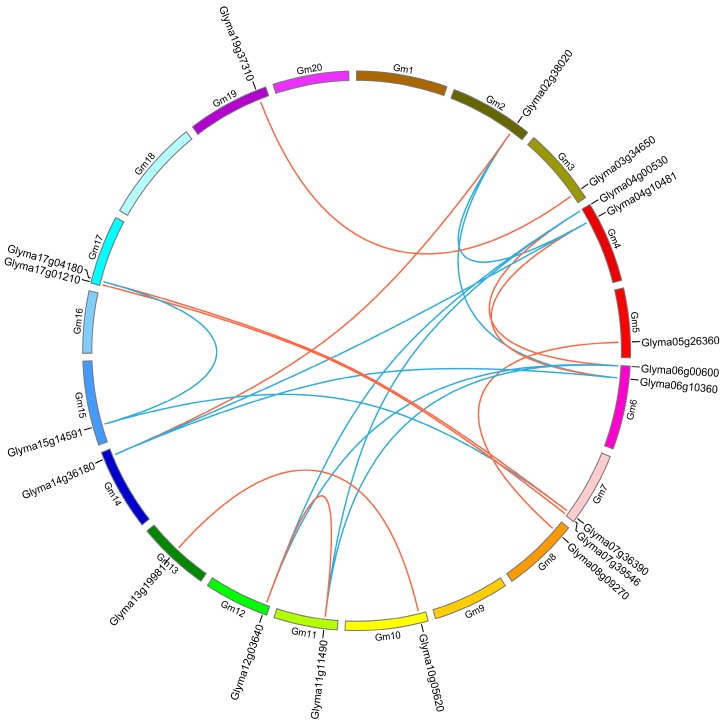
Chromosome locations of HECT genes and segmentally duplicated gene pairs in the soybean genome. Chromosomes 1–20 are shown with different colors and in a circular form. The approximate distribution of each soybean HECT gene is marked on the circle with a short black line. Colored curves denote the details of syntenic regions between soybean HECT genes (Blue and red curves represent the estimated time of duplication events-5–12 Mya (million year ago) and 32–46 Mya, respectively).

To determine the genomic locations of the HECT genes, the 19 soybean HECT genes were mapped on the 20 chromosomes in the soybean sequence database in Phytozome v9.1. The soybean HECT genes are randomly located on 15 of 20 chromosomes: chromosomes 1, 9, 16, 18, and 20 contain no HECT genes, chromosomes 4, 6, 7, and 17 each contain two HECT genes, while the other chromosomes each contain only one HECT gene (Figure 5). Segmental and tandem duplication are the two primary phenomena causing gene family expansion in plants [29,30]. Additionally, in order to examine the duplication patterns of the soybean HECT genes, we identified tandem duplications based on the gene loci, and searched the Plant Genome Duplication Database (PGDD) [31] to locate segmentally duplicated pairs. No tandem duplicated pairs were detected in the 19 soybean HECT genes. However, all 19 HECT genes were found to have been involved in segmental duplication (Figure 5). To date the duplication time of these segmentally duplicated HECT genes, we estimated the synonymous (Ks) and nonsynonymous substitution (Ka) distance, as well as the Ka/Ks ratios. The ratio of Ka/Ks for each segmentally duplicated gene pair varied from 0.13 to 0.44, with an average of 0.23 (Table 2). This analysis suggests that the duplicated HECT genes are under strong negative selection, as their Ka/Ks ratios were estimated to be <1. The approximate date of each duplication event was calculated using Ks (Table 2). We found that in each group, the two closest leaves of the soybean HECT gene phylogeny duplicated about 5–12 Mya, while the others duplicated about 32–46 Mya.

**Table 2:** Estimates of the dates for the segmental duplication events in the HECT gene pairs in soybean.

Group	Gene Locus 1	Gene Locus 2	Ka	Ks	Ka/Ks	Mya
I	*Glyma05g26360*	*Glyma08g09270*	0.02	0.08	0.25	6.56
II	*Glyma02g38020*	*Glyma04g10481*	0.1	0.51	0.2	41.8
	*Glyma02g38020*	*Glyma06g10360*	0.09	0.49	0.18	40.16
	*Glyma02g38020*	*Glyma14g36180*	0.02	0.09	0.22	7.38
	*Glyma04g10481*	*Glyma06g10360*	0.04	0.09	0.44	7.38
	*Glyma04g10481*	*Glyma14g36180*	0.1	0.5	0.2	40.98
	*Glyma06g10360*	*Glyma14g36180*	0.09	0.49	0.18	40.16
III	*Glyma07g39546*	*Glyma17g01210*	0.03	0.14	0.21	11.48
IV	*Glyma07g36390*	*Glyma15g14591*	0.09	0.4	0.23	32.79
	*Glyma07g36390*	*Glyma17g04180*	0.02	0.09	0.22	7.38
	*Glyma15g14591*	*Glyma17g04180*	0.1	0.42	0.24	34.43
V	*Glyma03g34650*	*Glyma19g37310*	0.03	0.07	0.43	5.74
VI	*Glyma04g00530*	*Glyma06g00600*	0.03	0.09	0.33	7.38
	*Glyma04g00530*	*Glyma11g11490*	0.07	0.55	0.13	45.08
	*Glyma04g00530*	*Glyma12g03640*	0.07	0.52	0.13	42.62
	*Glyma06g00600*	*Glyma11g11490*	0.09	0.55	0.16	45.08
	*Glyma06g00600*	*Glyma12g03640*	0.08	0.51	0.16	41.8
	*Glyma11g11490*	*Glyma12g03640*	0.02	0.08	0.25	6.56
VII	*Glyma10g05620*	*Glyma13g19981*	0.03	0.1	0.3	8.2

Ks: synonymous substitution rate; Ka: nonsynonymous substitution rate; Mya: million year ago.

### 2.5. Conserved Residues in the HECT Domain

Despite the lack of information concerning the three-dimensional structure of genes in the plant HECT domain, their architectures have been described by studies of the crystal structure of the HECT domain of human HECT Nedd4 [21,25]. This makes it possible to investigate the structure and function of plant HECT domains.

**Figure 6:**
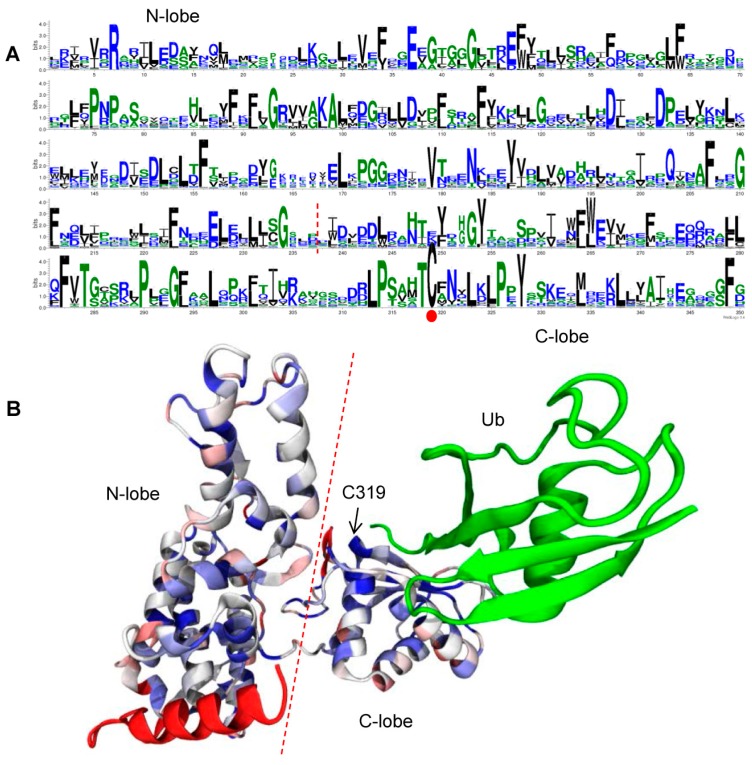
Logo and 3D representations of the highly conserved residues of 365 HECT domains in plants. Bits in the *y*-axis (**A** and Figure S3) represent the amount of informational content at each sequence position; Note that in the 3D representations (**B**), green represents ubiquitin (Ub), and the similarity values are mapped to a color gradient from low (red) to high rate of conservation (blue).

We used WebLogo3 [32] to visualize the conserved residues in the HECT domain, and found that both the *N*-lobe and *C*-lobe of the HECT domain contain critical conserved residues (Figure 6A). In addition, in order to describe these conserved residues in the context of the three-dimensional structure, we aligned the 365 HECT domain sequences with the downloaded HECT domain structure 4BBN chain A [21]. There is an abundance of conserved residues in the 365 plant HECT domain sequences (see Figure 6B, conserved residues shown in blue). In particular, almost half of the sites near the highly conserved catalytic C at site 319 in the *C*-lobe are highly conserved (L313, P314, T318, C319, N321, L323, L325, P326, and Y328) (for convenience, the first residue of the HECT domain is designed as site 1). Furthermore, domain logo results for the 7 HECT gene groups of 41 plant species show that in each group, almost all residues are highly conserved (Figure S3).

### 2.6. Expression Patterns of Soybean HECT Genes

**Figure 7:**
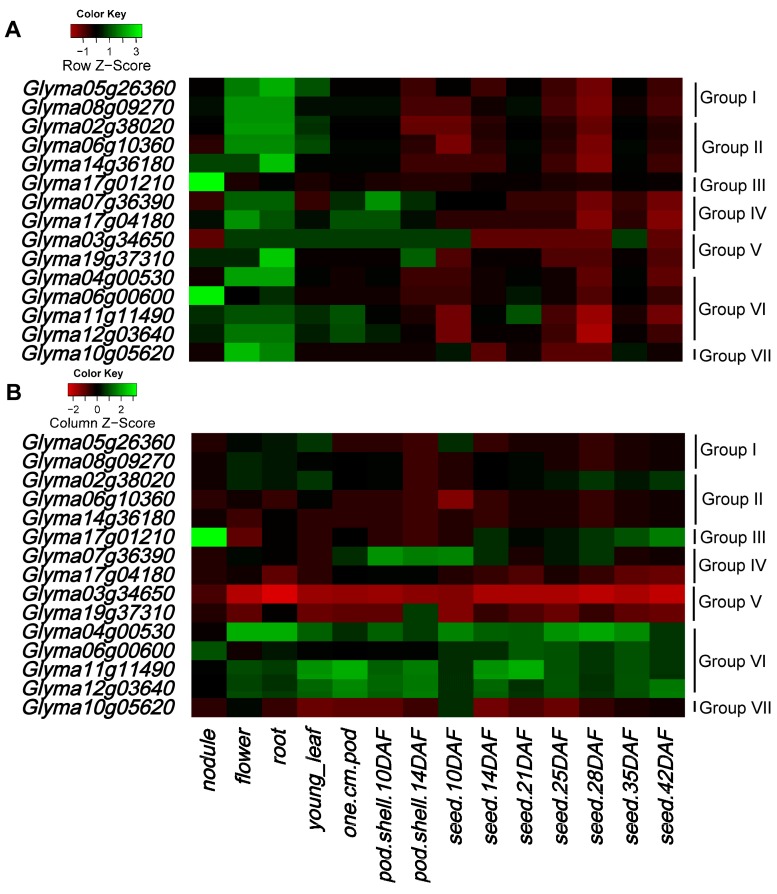
Heatmap of expression profiles of soybean HECT genes in 14 tissues. Normalized transcriptional levels were obtained from Severin *et al.* [33]. The RNA-seq relative expression data of 14 tissues was used to reconstruct the expression patterns of soybean genes. Color in the heatmaps represents *Z*-score of RPKM values of soybean HECT genes calculated per row (gene) (**A**) and per column (tissue) (**B**), respectively. *Z*-scores calculated per row (**A**) were used to show the changes of expression of a gene across different tissues, and *Z*-scores calculated per column (**B**) were used to rank genes for each sample. The sources of the samples are as follows: young leaf, flower, one cm pod, pod shell 10DAF (day after flowering), pod shell 14DAF, seed 10DAF, seed 14DAF, seed 21DAF, seed 25DAF, seed 28DAF, seed 35DAF, seed 42DAF, root, and nodule.

To explore the expression patterns of these soybean HECT genes, we used RNA-seq data from SoySeq [33]. Based on the soybean RNA-seq data, 15 HECT genes were detected in all 14 tissues at the gene level (Figure 7 and Table S3). This suggests that most HECT genes are broadly expressed during soybean development. Most HECT genes in the flowers and roots were relatively highly expressed, while those in the pod shell and seed were relatively lowly expressed (Figure 7A). In addition, genes within each group or in different groups often had similar expression patterns in different tissues, as was the case with the expression of group II (*Glyma02g38020*, *Glyma06g10360*, *Glyma14g36180*) and group VI (*Glyma04g00530*, *Glyma11g11490*, *Glyma12g03640*) (Figure 7A). However, unlike other genes, two genes—*Glyma17g01210* in group III and *Glyma06g00600* in group VI—were relatively highly expressed in the nodules than other tissues (Figure 7A). For each tissue, the group VI HECT genes (*Glyma04g00530*, *Glyma06g00600*, *Glyma11g11490* and *Glyma 12g03640*) were almost relatively highly expressed for all samples (except nodule) (Figure 7B). In nodule, the *Glyma17g01210* in group III had a relatively higher expression than other HECT genes (Figure 7B).

## 3. Discussion

*Arabidopsis thaliana* HECT family genes play crucial roles in various plant developmental and physiological processes [3,6,7,34], including trichome development [7], genome endoreduplication [6], and leaf senescence [3]. However, this gene family has not been studied in soybean. In this study, we performed a comprehensive analysis of the soybean HECT gene family, including an analysis of the genes’ phylogeny, gene structure, domain architecture, chromosome location, duplication patterns, conserved residues, and expression profiles.

In this study, 19 HECT genes were identified in the soybean genome, which is 2.7 times the number present in *Arabidopsis thaliana*. However, a recent study found there to be 15 HECT genes in soybean [22]. Our results revealed that there are four more HECT genes (group I: *Glyma05g26360*, group II: *Glyma06g10360*, *Glyma14g36180*, and group V: *Glyma19g37310*) in soybean genome than previously estimated (Figure S4). There are two possible explanations for this discrepancy. First, the latest update of the soybean genome database includes a number of newly assembled and imported genes. Second, the search methods implemented in our study and differed from those used in the previous study. We used the combined method of HMMER–Blast–InterProScan, while the previous study used TBlastN.

The results of the phylogenetic analysis of 365 plant HECT genes from 41 plant species divided the soybean HECT genes into subfamilies similar to those described in previous reports [7,22,23]. The divisions were based on corresponding HECT domain sequence homology. According to the phylogenetic relationships between the HECT genes in *Glycine max*, *Medicago truncatula*, *Phaseolus vulgaris*, and *Arabidopsis thaliana* (outgroup), soybean HECT genes can be divided into seven groups. Compared with previous study [22], subfamily IV HECT genes were absent in these plants. Subfamily V (3 genes) corresponds to group I (2 genes) and II (4 genes) and subfamily I (6 genes) corresponds to group VI (4 genes) and VII (2 genes) in this study. Subfamily II (1 gene) corresponds to group V (2 genes), subfamily III (3 genes) corresponds to group IV (3 genes), and subfamily VI (2 genes) corresponds to group III (2 genes). Except for group II, all soybean HECT gene groups have orthologous genes in *Arabidopsis thaliana*. This is consistent with the results of a recent plant HECT study [22], which indicated that *Arabidopsis thaliana* HECT group II (*UPL8* in their study) was absent. Members of each group usually have identical gene structures and domain architectures, which suggests that they may interact with identical or similar substrates.

Segmental duplication, tandem duplication, and transposition events are the three principal evolutionary patterns of gene duplication that cause gene family expansion [30,35,36,37]. Of these, segmental duplication events happen most frequently in plants, because most plants are diploidized polyploids and retain numerous duplicated chromosomal blocks within their genomes [30]. In this study, we found that all soybean HECT genes are located in duplicated blocks, suggesting that segmental duplication contributed significantly to the expansion of the soybean HECT gene family. A previous study has shown that the soybean genome has undergone two genome duplication events, at 58 and 13 Mya [28]. By estimating the duplication date of the duplicated pairs of soybean HECT genes, we postulate that the paralogous genes in group II, IV, and VI originate from both the ancient and recent duplication event, while in group I, III, V, and VII they originate from the recent duplication event.

Analysis of the expression patterns of these soybean genes in 14 tissues showed that most HECT genes were relatively highly expressed in flowers and roots. However, *Glyma06g00600* and *Glyma17g01210* were highly expressed in the nodules. From this, we inferred that the highly expressed HECT genes in the flowers may be involved in the degradation of genes relating to flowering, via ubiquitination during soybean flowering stage. Additionally, the results suggested that the highly expressed genes in roots and nodules may directly or indirectly control the expression of nitrogen-fixing genes during symbiotic nitrogen fixation. Previous studies have revealed that *Arabidopsis thaliana* *AT4G38600/UPL3* restricts the rounds of genome endoreduplication and cell branching that occur during trichome development [7], and *AT4G12570*/*UPL5* regulates leaf senescence through the degradation of *AT4G23810*/*WRKY53*, a transcription factor that acts positively in leaf senescence [3]. In our analysis, the soybean genes orthologous to *Arabidopsis thaliana* *AT4G38600*/*UPL3* are four paralogous genes in group VI. These four genes were all expressed in soybean, but display different expression patterns in different tissues. Except for *Glyma06g00600*, which is expressed relatively highly in nodules, the other three genes are relatively highly expressed in roots and flowers. This may be caused by mutations accumulated after the two segmental duplication events, especially the latest duplication events. The soybean genes orthologous to *Arabidopsis thaliana* *AT4G12570*/*UPL5* are two paralogous genes in group III. *Glyma17g01210* was also highly expressed in nodules, while *Glyma7g39546* was not expressed. The differential expression of paralogous genes of the same group indicates that the HECT genes in soybean may have the same or similar function as their orthologues in *Arabidopsis thaliana*; however, they may have evolved functional differences.

A recent report showed that ubiquitin-proteasome system (UPS) dependent proteolysis of the two transcription factors, *AT5G41315*/*GL3* and *AT1G63650*/*EGL3*, is mediated by *AT4G38600*/*UPL3* [34]. *GLABROUS 3* (*GL3*) and *ENHANCER OF GLABROUS 3* (*EGL3*), which function as positive regulators of trichome development, interact with the *N*-terminal ARM domains of *UPL3* via their *C*-terminal domains. Moreover, other recent studies have revealed that the highly conserved residues in the three-dimensional structures of the HECT domain are essential for the ubiquitylation of proteins [21,25,26,27]. Our analysis of 365 plant HECT domains shows that many highly conserved residues are present, suggesting that these conserved residues still play key roles in structural maintenance, and are involved in plant ubiquitination processes. Further functional analysis of these genes would better our understanding of the functional roles of HECT genes in soybean and other plants.

## 4. Experimental Section

### 4.1. Identification of HECT Genes in Soybean

The soybean genome database (release v1.1) was downloaded from Phytozome v9.1 [28]. The HMM profile of the HECT domain (PF00632) was obtained from Pfam [38,39]. To identify potential HECT genes in soybean, the HECT domain profile PF00632 was used as a query for searching the soybean genome database, using the HMMER3.1 [40,41] program, hmmsearch, with its default parameters (*E*-value < 10^−5^). To obtain the complete soybean HECT genes, the HMMER search results were used as queries in searching the soybean genome database a second time, using the BlastP and tBlastN programs [42] with their default parameters (*E*-value < 10^−5^). All hits were subsequently verified using the InterProScan program [43] to confirm the presence of the HECT domain. Finally, the Pfam [38,39], PROSITE [44], SMART [45], SUPERFAMLIY [46], PANTHER [47], and Gene3D [48] databases were used to manually determine the domain architecture of each HECT gene. Sequences with an incomplete HECT domain or fewer than 300 amino acids were excluded from the final sequence dataset. In addition, similar analyses of HECT genes were performed for the other 40 plant species in Phytozome v9.1.

### 4.2. Phylogenetic Analysis and Gene Structure

The retrieved protein sequences were aligned using MUSCLE [49] with its default parameters, and MAFFT [50,51] (L-INS-i strategy). The alignment was filtered for informative sites using trimal v1.4, with the option-gappyout [52]. ProtTest v3.4 [53] was used to estimate the most appropriate model of amino acid substitution using both Akaike information and Bayesian information criterion, which together suggested that the Jones-Taylor-Thornton and γ-distributed site rates (JTT + G) model was the best-fit model. The filtered alignment was then used in the phylogenetic analysis, which in turn utilized maximum likelihood (ML) methods implemented in PhyML3.0 [54]. The analysis included 4 rate substitution categories, the JTT substitution model, a BIONJ starting tree, and 100 bootstrap repetitions. The final alignment was carried out based on the HECT domain alone, using the MAFFT (G-INS-i strategy). The Neighbor-Joining (NJ) trees of full-length amino acids sequences were constructed using the MEGA6 package with 1000 bootstrap repetitions under the JTT substitution model. Phylogenetic trees were visualized and annotated using the Interactive Tree of Life v2 Web server [55] and EvolView [56]. The structures of the HECT genes were made using the Gene Structure Display Server [57], via comparisons of the coding sequences with their corresponding genomic sequences.

### 4.3. Chromosome Location and Duplication

Information about the chromosome location of the HECT genes was obtained from the Phytozome v9.1. Duplication patterns of the soybean HECT genes were inferred based on their locations in the soybean genome. Tandem duplicated genes were defined as adjacent homologous genes located on the same chromosome, and separated by no more than five genes in a 100-kb region [58]. Segmentally duplicated genes were defined as two genes located on duplicated chromosomal blocks [29]. To determine whether an HECT gene was involved in segmental duplication, the syntenic blocks of each HECT gene were searched for in the Plant Genome Duplication Database [31], and visualized using Circos-0.67 [59].

### 4.4. Calculation of Synonymous (Ks) and Nonsynonymous Substitution (Ka) to Date Duplication Events

Synonymous (Ks) and nonsynonymous substitution (Ka) rates were calculated according to methods used in previous studies [29,58]. First, MUSCLE v3.8.31 [49] (with default parameters) was used to construct pairwise alignments of the protein sequences of the duplicated gene pairs. The coding sequence alignments based on these amino acid sequence alignments were guided by trimal v1.4 [52], with the option-backtrans. Then, Ks and Ka were estimated using the CODEML program in PAML (Phylogenetic Analysis by Maximum Likelihood) v4.8 [60]. For each gene pair, the approximate date of the duplication event was calculated using the mean Ks values from T = Ks/2λ, in which the mean synonymous substitution rate (λ) for soybean is 6.1 × 10^−9^ [61].

### 4.5. Logos of HECT Domains and Three-Dimensional Representations of Domain Alignment

Logos of the HECT domains were generated using WebLogo3 online [32] (using the default parameters). Three hundred and sixty-five HECT domain sequences with the downloaded HECT NEDD4 (neural precursor cell expressed developmentally down-regulated protein 4) domain structure (PDB ID: 4BBN, chain A) [21] were aligned using the VMD (Visual Molecular Dynamics) MultiSeq alignment [62,63] method (coloring method: Sequence Similarity BLOSUM 90).

### 4.6. Expression Analyses

RNA-Seq data were downloaded from SoySeq [33] and used to analyze the expression patterns of HECT genes in soybean. These transcript data were obtained from 14 tissues, including underground tissues (root and nodule), seed development (seed 10DAF, seed 14DAF, seed 21DAF, seed 25DAF, seed 28DAF, seed 35DAF, and seed 42DAF), and aerial tissues (leaf, flower, pod-shell 10DAF, pod shell 14DAF, and one cm pod). The expression data were normalized RPKM (reads per kilobase per million mapped reads), and the heatmap was drawn in R.

## Supplementary Materials

Supplementary materials can be found at http://www.mdpi.com/1422-0067/16/04/8517/s1.

## References

[1] Wang F., Deng X.W. (2011). Plant ubiquitin-proteasome pathway and its role in gibberellin signaling. Cell Res..

[2] Liu H., Stone S.L. (2011). E3 ubiquitin ligases and abscisic acid signaling. Plant Signal. Behav..

[3] Miao Y., Zentgraf U. (2010). A HECT E3 ubiquitin ligase negatively regulates *Arabidopsis* leaf senescence through degradation of the transcription factor WRKY53. Plant J..

[4] Kim H.T., Kim K.P., Lledias F., Kisselev A.F., Scaglione K.M., Skowyra D., Gygi S.P., Goldberg A.L. (2007). Certain pairs of ubiquitin-conjugating enzymes (E2s) and ubiquitin-protein ligases (E3s) synthesize nondegradable forked ubiquitin chains containing all possible isopeptide linkages. J. Biol. Chem..

[5] Moon J., Parry G., Estelle M. (2004). The ubiquitin-proteasome pathway and plant development. Plant Cell.

[6] El Refy A., Perazza D., Zekraoui L., Valay J.G., Bechtold N., Brown S., Hulskamp M., Herzog M., Bonneville J.M. (2003). The *Arabidopsis* KAKTUS gene encodes a HECT protein and controls the number of endoreduplication cycles. Mol. Genet. Genomics.

[7] Downes B.P., Stupar R.M., Gingerich D.J., Vierstra R.D. (2003). The HECT ubiquitin-protein ligase (UPL) family in *Arabidopsis*: UPL3 has a specific role in trichome development. Plant J..

[8] Stone S.L. (2014). The role of ubiquitin and the 26S proteasome in plant abiotic stress signaling. Front. Plant Sci..

[9] Wang M., Cheng D., Peng J., Pickart C.M. (2006). Molecular determinants of polyubiquitin linkage selection by an HECT ubiquitin ligase. EMBO J..

[10] Wang M., Pickart C.M. (2005). Different HECT domain ubiquitin ligases employ distinct mechanisms of polyubiquitin chain synthesis. EMBO J..

[11] Scheffner M., Nuber U., Huibregtse J.M. (1995). Protein ubiquitination involving an E1–E2–E3 enzyme ubiquitin thioester cascade. Nature.

[12] Huibregtse J.M., Scheffner M., Beaudenon S., Howley P.M. (1995). A family of proteins structurally and functionally related to the E6-AP ubiquitin-protein ligase. Proc. Natl. Acad. Sci. USA.

[13] Guzman P. (2014). ATLs and BTLs, plant-specific and general eukaryotic structurally-related E3 ubiquitin ligases. Plant Sci..

[14] Duplan V., Rivas S. (2014). E3 ubiquitin-ligases and their target proteins during the regulation of plant innate immunity. Front. Plant Sci..

[15] Chen L., Hellmann H. (2013). Plant E3 ligases: Flexible enzymes in a sessile world. Mol. Plant.

[16] Yee D., Goring D.R. (2009). The diversity of plant U-box E3 ubiquitin ligases: From upstream activators to downstream target substrates. J. Exp. Bot..

[17] Craig A., Ewan R., Mesmar J., Gudipati V., Sadanandom A. (2009). E3 ubiquitin ligases and plant innate immunity. J. Exp. Bot..

[18] Qin F., Sakuma Y., Tran L.S., Maruyama K., Kidokoro S., Fujita Y., Fujita M., Umezawa T., Sawano Y., Miyazono K. (2008). *Arabidopsis* DREB2A-interacting proteins function as RING E3 ligases and negatively regulate plant drought stress-responsive gene expression. Plant Cell.

[19] Schwechheimer C., Calderon Villalobos L.I. (2004). Cullin-containing E3 ubiquitin ligases in plant development. Curr. Opin. Plant Biol..

[20] Mach J. (2008). Ubiquitin ligation RINGs twice: Redundant control of plant processes by E3 ubiquitin ligases. Plant Cell.

[21] Maspero E., Valentini E., Mari S., Cecatiello V., Soffientini P., Pasqualato S., Polo S. (2013). Structure of a ubiquitin-loaded HECT ligase reveals the molecular basis for catalytic priming. Nat. Struct. Mol. Biol..

[22] Marin I. (2013). Evolution of plant HECT ubiquitin ligases. PLoS ONE.

[23] Grau-Bove X., Sebe-Pedros A., Ruiz-Trillo I. (2013). A genomic survey of HECT ubiquitin ligases in eukaryotes reveals independent expansions of the HECT system in several lineages. Genome Biol. Evol..

[24] Kamadurai H.B., Qiu Y., Deng A., Harrison J.S., Macdonald C., Actis M., Rodrigues P., Miller D.J., Souphron J., Lewis S.M. (2013). Mechanism of ubiquitin ligation and lysine prioritization by a HECT E3. Elife.

[25] Maspero E., Mari S., Valentini E., Musacchio A., Fish A., Pasqualato S., Polo S. (2011). Structure of the HECT:ubiquitin complex and its role in ubiquitin chain elongation. EMBO Rep..

[26] Kim H.C., Steffen A.M., Oldham M.L., Chen J., Huibregtse J.M. (2011). Structure and function of a HECT domain ubiquitin-binding site. EMBO Rep..

[27] Rotin D., Kumar S. (2009). Physiological functions of the HECT family of ubiquitin ligases. Nat. Rev. Mol. Cell Biol..

[28] Schmutz J., Cannon S.B., Schlueter J., Ma J., Mitros T., Nelson W., Hyten D.L., Song Q., Thelen J.J., Cheng J. (2010). Genome sequence of the palaeopolyploid soybean. Nature.

[29] Zhu Y., Wu N., Song W., Yin G., Qin Y., Yan Y., Hu Y. (2014). Soybean (*Glycine max*) expansin gene superfamily origins: segmental and tandem duplication events followed by divergent selection among subfamilies. BMC Plant Biol..

[30] Cannon S.B., Mitra A., Baumgarten A., Young N.D., May G. (2004). The roles of segmental and tandem gene duplication in the evolution of large gene families in *Arabidopsis thaliana*. BMC Plant Biol..

[31] Lee T.H., Tang H., Wang X., Paterson A.H. (2013). PGDD: A database of gene and genome duplication in plants. Nucleic Acids Res..

[32] Crooks G.E., Hon G., Chandonia J.M., Brenner S.E. (2004). WebLogo: A sequence logo generator. Genome Res..

[33] Severin A.J., Woody J.L., Bolon Y.T., Joseph B., Diers B.W., Farmer A.D., Muehlbauer G.J., Nelson R.T., Grant D., Specht J.E. (2010). RNA-Seq Atlas of *Glycine max*: A guide to the soybean transcriptome. BMC Plant Biol..

[34] Patra B., Pattanaik S., Yuan L. (2013). Ubiquitin protein ligase 3 mediates the proteasomal degradation of GLABROUS 3 and ENHANCER OF GLABROUS 3, regulators of trichome development and flavonoid biosynthesis in *Arabidopsis*. Plant J..

[35] Kong H., Landherr L.L., Frohlich M.W., Leebens-Mack J., Ma H., dePamphilis C.W. (2007). Patterns of gene duplication in the plant SKP1 gene family in angiosperms: Evidence for multiple mechanisms of rapid gene birth. Plant J..

[36] Gu Z., Steinmetz L.M., Gu X., Scharfe C., Davis R.W., Li W.H. (2003). Role of duplicate genes in genetic robustness against null mutations. Nature.

[37] Bowers J.E., Chapman B.A., Rong J., Paterson A.H. (2003). Unravelling angiosperm genome evolution by phylogenetic analysis of chromosomal duplication events. Nature.

[38] Finn R.D., Bateman A., Clements J., Coggill P., Eberhardt R.Y., Eddy S.R., Heger A., Hetherington K., Holm L., Mistry J. (2014). Pfam: The protein families database. Nucleic Acids Res..

[39] Punta M., Coggill P.C., Eberhardt R.Y., Mistry J., Tate J., Boursnell C., Pang N., Forslund K., Ceric G., Clements J. (2012). The Pfam protein families database. Nucleic Acids Res..

[40] Finn R.D., Clements J., Eddy S.R. (2011). HMMER web server: Interactive sequence similarity searching. Nucleic Acids Res..

[41] Eddy S.R. (2011). Accelerated profile HMM searches. PLoS Comput. Biol..

[42] Camacho C., Coulouris G., Avagyan V., Ma N., Papadopoulos J., Bealer K., Madden T.L. (2009). BLAST+: Architecture and applications. BMC Bioinform..

[43] Jones P., Binns D., Chang H.Y., Fraser M., Li W., McAnulla C., McWilliam H., Maslen J., Mitchell A., Nuka G. (2014). InterProScan 5: Genome-scale protein function classification. Bioinformatics.

[44] Sigrist C.J., de Castro E., Cerutti L., Cuche B.A., Hulo N., Bridge A., Bougueleret L., Xenarios I. (2013). New and continuing developments at PROSITE. Nucleic Acids Res..

[45] Letunic I., Doerks T., Bork P. (2012). SMART 7: Recent updates to the protein domain annotation resource. Nucleic Acids Res..

[46] Wilson D., Pethica R., Zhou Y., Talbot C., Vogel C., Madera M., Chothia C., Gough J. (2009). SUPERFAMILY—Sophisticated comparative genomics, data mining, visualization and phylogeny. Nucleic Acids Res..

[47] Mi H., Muruganujan A., Thomas P.D. (2013). PANTHER in 2013: Modeling the evolution of gene function, and other gene attributes, in the context of phylogenetic trees. Nucleic Acids Res..

[48] Lees J.G., Lee D., Studer R.A., Dawson N.L., Sillitoe I., Das S., Yeats C., Dessailly B.H., Rentzsch R., Orengo C.A. (2014). Gene3D: Multi-domain annotations for protein sequence and comparative genome analysis. Nucleic Acids Res..

[49] Edgar R.C. (2004). MUSCLE: Multiple sequence alignment with high accuracy and high throughput. Nucleic Acids Res..

[50] Kuraku S., Zmasek C.M., Nishimura O., Katoh K. (2013). aLeaves facilitates on-demand exploration of metazoan gene family trees on MAFFT sequence alignment server with enhanced interactivity. Nucleic Acids Res..

[51] Katoh K., Standley D.M. (2013). MAFFT multiple sequence alignment software version 7: Improvements in performance and usability. Mol. Biol. Evol..

[52] Capella-Gutierrez S., Silla-Martinez J.M., Gabaldon T. (2009). trimAl: A tool for automated alignment trimming in large-scale phylogenetic analyses. Bioinformatics.

[53] Darriba D., Taboada G.L., Doallo R., Posada D. (2011). ProtTest 3: Fast selection of best-fit models of protein evolution. Bioinformatics.

[54] Guindon S., Dufayard J.F., Lefort V., Anisimova M., Hordijk W., Gascuel O. (2010). New algorithms and methods to estimate maximum-likelihood phylogenies: Assessing the performance of PhyML 3.0. Syst. Biol..

[55] Letunic I., Bork P. (2011). Interactive Tree Of Life v2: Online annotation and display of phylogenetic trees made easy. Nucleic Acids Res..

[56] Zhang H., Gao S., Lercher M.J., Hu S., Chen W.H. (2012). EvolView, an online tool for visualizing, annotating and managing phylogenetic trees. Nucleic Acids Res..

[57] Guo A.Y., Zhu Q.H., Chen X., Luo J.C. (2007). GSDS: A gene structure display server. Yi Chuan.

[58] Chen X., Chen Z., Zhao H., Zhao Y., Cheng B., Xiang Y. (2014). Genome-wide analysis of soybean HD-Zip gene family and expression profiling under salinity and drought treatments. PLoS ONE.

[59] Krzywinski M., Schein J., Birol I., Connors J., Gascoyne R., Horsman D., Jones S.J., Marra M.A. (2009). Circos: An information aesthetic for comparative genomics. Genome Res..

[60] Yang Z. (2007). PAML 4: Phylogenetic analysis by maximum likelihood. Mol. Biol. Evol..

[61] Lynch M., Conery J.S. (2000). The evolutionary fate and consequences of duplicate genes. Science.

[62] Roberts E., Eargle J., Wright D., Luthey-Schulten Z. (2006). MultiSeq: Unifying sequence and structure data for evolutionary analysis. BMC Bioinform..

[63] Humphrey W., Dalke A., Schulten K. (1996). VMD: Visual molecular dynamics. J. Mol. Graph..

