# Supplementary material for: Genome-Wide Identification and Evolution of HECT Genes in Soybean
Source: Int J Mol Sci. 2015 Apr 16;16(4):8517–35. doi: 10.3390/ijms16048517 (PMC4425094; doi:10.3390/ijms16048517)
Supplement: Supplementary file 1 [file ijms-16-08517-s001.zip › ijms-80517-Supplementary Information/File S1.html]

trimAl v1.4 Summary


```
    Selected Sequences:   365 /Selected Residues:     350
    Deleted Sequences:      0 /Deleted Residues:     2459

    Gaps Scores:                        =0=   <.001  <.050  <.100  <.150  <.200  <.250  <.350  <.500  <.750  <1.00   =1=  

                                              10        20        30        40        50        60        70        80        90       100       110       120
                                      =========+=========+=========+=========+=========+=========+=========+=========+=========+=========+=========+=========+
    Sb01g011845.1                     IEI-VIRR-GHII---EDGYRQL-----------------NCLR--SK-LK-S-CIH-VSFVSECG-------------LPEAGLDYGGLSKEFLTDLSKTAF------------S----
    Sb02g016200.1                     LRI-SVRR-AYVL---EDSYNQL-----------------RLRRT-QD-LK-G-RLT-VQF------------------QGEEGIDAGGLTREWYQLLSRVIF------------D----
    Sb04g000340.1                     QKV-RVSR-NRIL---DSAAKVM-----------------EMFSS----QR-A-VLE-VEY------------------FGEVGT-GLGPTLEFYTLLSHELQ------------S----
    Sb06g003290.1                     QKV-RVSR-NRIL---DSAAKVM-----------------EMFSN----QK-A-VLE-VEY------------------FGEVGT-GLGPTLEFYTLLSRELQ------------R----
    Sb08g012560.1                     VRI-SVRR-AYIL---EDSYNQL-----------------RMRSP-QE-LK-G-RLT-VHF------------------QAEEGIDAGGLTREWYQSLSRVIF------------D----
    Sb09g002120.1                     KKY-RVAR-SAIL---EGAVSVM-----------------TSHGS----SS-R-IID-VEF------------------EGEVGT-GRGPTFEFYTTVTHELQ------------R----
    Sb09g004530.1                     QRF-KIRR-NRLL---EDAFDQL-----------------SLLSE-ED-LK-G-PIR-VSFINEHG-------------EEEAGIDGGGIFKDFMENITRAAF------------D----
    Sb09g022820.1                     KKF-KVDR-DDIL---VSAAKMM-----------------KSHAK----SN-A-LLE-VEY------------------KEEVGT-GLGPTMEFYTLISHEFQ------------K----
    73381                             EEI-RIHR-HDIL---KDSYEEL-----------------GQRSA-EK-LK-KFQLS-VHF------------------RGEEG-EGEGLGREWFQVVSQAIV------------E----
    50844                             QEI-VIQR-SQLL---TESLEQL-----------------VYVEA-EN-IQ-G-GLS-VEF------------------SSEEAT-GPGVLREWFFMVCKEIF------------N----
    89794                             IEI-SIRR-DRIV---EDGFQQL-----------------GALG--AR-FK-G-CIN-VSFVNEHG-------------LTEAGLDYGGLFKEFLTDLAKAAF------------D----
    3542                              -------------------------------------------------------------------------------------DLGGLRREWFHLVSREIG------------E----
    76253                             LIL-RVRR-DFLV---RDTIVQI-----------------QEQL--GD-LK-K-PLK-VVF------------------VGEEGIDEGGVQKEFFQLLVRELF------------N----
    443962                            LQI-KVQR-DRII---EDAFMQF-----------------NVLSD-ES-LR-G-TIR-VSYVNELG-------------AEEAGVDGGGIFKDFMENITSAGF------------D----
    181768                            --L-QVRR-TSLV---QDSLHQL-----------------SLH-H-YE-LK-K-PLM-VIF------------------EGESGVDQGGVTKEFFQLLVRDLF------------N----
    407700                            TRL----RPSCILIAPNNEFSCF-----------------QFAFN-TN-LR-TPNYK-VVYASEI---------GGALVWEESTIDAGGPHAKAIREAVRQIP------------G----
    146155                            QKV-RVSR-TRIL---DSAAKVM-----------------ELYSG----HK-A-VLE-VEY------------------FGEVGT-GLGPTLEFYTLVSRELQ------------K----
    154179                            LRI-SVRR-AYVL---EDSYNQL-----------------RMRTP-DE-VK-G-RLT-VQF------------------QGEEGIDAGGLTREWYQLLSRVIF------------D----
    943823                            QKV-RVSR-NRIL---DSAAKVM-----------------EMYSS----QK-A-VLE-VEY------------------FGEVGT-GLGPTLEFYTLLSHDLQ------------K----
    487067                            KKF-LVCR-EKIL---ESAAKMM-----------------ELYGN----QK-V-VIE-VEY------------------SEEVGT-GLGPTLEFYTLVSRAFQ------------N----
    485684                            IEI-VVRR-GHVV---EDGFQQL-----------------NSIG--SR-LK-S-SIH-VSFVNESG-------------LPEAGLDYGGLSK----------------------------
    490058                            HEM-LIDR-SNLL---AESFEYI-----------------VGASP-EA-LH-G-GLF-MEF------------------KNEEAT-GPGVLREWFYLVCQEIF------------N----
    479191                            NRF-RIRR-DHIL---EDAYNQM-----------------SALSE-DD-LR-G-SIR-VTFVNELG-------------VEEAGIDGGGIFKDFMEKITRAAF------------D----
    916552                            LRI-SVRR-AYIL---EDSYNQL-----------------RMRST-QE-LK-G-RLT-VHF------------------QGEEGIDAGGLTREWYQLLSRVIF------------D----
    940321                            KDI-KIPR-SNLL---VESSLQI-----------------MAASP-ES-LQ-H-GIS-VEF------------------DLEPGI-GDGVTREFLLLLSEEIFRCD--------VK----
    474651                            LRI-SVRR-AYVL---EDSYNQL-----------------RMRSP-QD-LK-G-RLN-VQF------------------QGEEGIDAGGLTREWYQLLSRVIF------------D----
    915021                            LQV-VVSR-TSLF---KDSLNQV-----------------MAADP-WD-FH-A-GIS-IQF------------------EYEEAE-GDGVLREWLCLVCNNLF------------D----
    evm.model.supercontig_146.73      NRF-RIRR-HHIL---EDAYNQM-----------------SALSE-ED-LR-G-LIR-VTFVNEFG-------------VEEAGIDGGGIFKDFMENITRAAF------------D----
    evm.model.supercontig_21.42       LRI-SVRR-AYIL---EDSYNQL-----------------RMRST-QE-LK-G-RLT-VHF------------------QGEEGIDAGGLTREWYQLLSRVIF------------D----
    evm.model.supercontig_37.145      QKV-RVSR-NRIL---DSAAKVM-----------------EMYSS----QK-A-VLE-VEY------------------FGEVGT-GLGPTLEFYTLLSHDLQ------------K----
    evm.model.supercontig_5.113       IEI-VVRR-GHVV---EDGFRQL-----------------NSLG--AR-LK-S-SIH-VSFVSECG-------------LPEAGLDYGGLSKEFLTDISKAAF------------A----
    evm.model.supercontig_959.1       LRI-SVRR-AYVL---EDSYNQL-----------------RMRPT-QD-LK-G-RLN-VQF------------------QGEEGIDAGGLTREWYQLLSRVIF------------D----
    29206.m000140                     HEM-LIDR-SQLL---AESFEYI-----------------ARAEP-EL-LH-G-GLF-MEF------------------KNEEAT-GPGVLREWFFLVVQALF------------N----
    29596.m000712                     LRI-SVRR-AYIL---EDSYNQL-----------------RMRST-QD-LK-G-RLT-VHF------------------QGEEGIDAGGLTREWYQLLSRVIF------------D----
    29602.m000214                     QKV-RVSR-NRIL---DSAAKVM-----------------EMYSS----QK-A-VLE-VEY------------------FGEVGT-GLGPTLEFYTLLSHDLQ------------K----
    29629.m001405                     LRI-SVRR-AYVL---EDSYNQL-----------------RMRPS-QD-LK-G-RLN-VQF------------------QGEEGIDAGGLTREWYQLLSRVIF------------D----
    29805.m001489                     KKF-VVWR-DRIM---ESASQMM-----------------DLYAG----VK-V-PIE-VVY------------------NEEVGS-GLGPTLEFYTLVSHEFQ------------K----
    29815.m000491                     VEI-VVRR-GHIV---EDGFRQL-----------------NTLG--SR-LK-S-SIH-VSFVSECG-------------VPEAGLDYGGLSKEFLTDISKASF------------S----
    29889.m003352                     NRF-RIRR-DRIL---EDAYNQM-----------------STLSE-ED-LR-G-LIR-VTFVNEFG-------------VEEAGIDGGGIFKDFMENITRAAF------------D----
    Cucsa.042120.1                    LRI-SVRR-AYVL---EDSYNQL-----------------RMRPT-QD-LR-G-RLN-VQF------------------QGEEGIDAGGLTREWYQLLSRVIF------------D----
    Cucsa.044750.1                    QEM-LIDR-SQLL---EESFEYI-----------------TNASV-EA-LR-H-GLF-MEF------------------KNEEAT-GPGVLREWFLLVCKSIF------------N----
    Cucsa.160480.1                    LRI-SVRR-AYIL---EDSYNQL-----------------RMRST-QD-LK-G-RLT-VHF------------------QGEEGIDAGGLSREWYQLLSRVIF------------D----
    Cucsa.234290.1                    QKV-RVSR-NRIL---DSAAKVM-----------------EMYSS----QK-A-VLE-VEY------------------FGEVGT-GLGPTLEFYTLLSHELQ------------R----
    Cucsa.307200.1                    KKV-LVHR-SQIL---DSASKMM-----------------NQYAN----QK-V-LLE-VEY------------------DEEVGT-GLGPTLEFYTLVSREFQ------------K----
    Cucsa.378730.1                    FEI-VVRR-SHVV---EDGFRQL-----------------NSLG--SK-LK-S-AIH-VSFVSECG-------------LPEAGQDCGGLSKEFLTDIAKAAF------------S----
    ppa000451m                        VEI-VVHR-GHIV---EDGFRQL-----------------NSLG--SR-LK-S-SIH-VSFVSECG-------------LPEAGLDYGGLSKEFLTDISKAAF------------A----
    ppa000008m                        LRI-SVRR-AYVL---EDSYNQL-----------------RMRPN-QD-MK-G-RLN-VQF------------------QGEEGIDAGGLTREWYQLLSRVIF------------D----
    ppa001143m                        HEM-LIDR-SQLL---AESFEYI-----------------GRAEP-ES-LH-G-GLF-MEF------------------KNEEAT-GPGVLREWFFLVCQAIF------------N----
    ppa000674m                        NRF-RIRR-DRIL---EDAYNQM-----------------SALSE-DD-LR-G-PIR-VTFVNEFG-------------VEEAGIDGGGIFKDFMENITRAAF------------D----
    ppa000169m                        KKF-LVFR-NQIL---DSAAQMM-----------------DLHAS----HK-V-LLE-VEY------------------NEEVGT-GLGPTLEFYTLVSHEFQ------------K----
    ppa000009m                        LRI-SVRR-AYIL---EDSYNQL-----------------RMRST-ED-LK-G-RLT-VHF------------------QGEEGIDAGGLTREWYQLLSRVIF------------D----
    ppa000080m                        QKV-RVSR-NRIL---DSAAKVM-----------------EMYSS----QK-S-VLE-VEY------------------FGEVGT-GLGPTLEFYTLLSHDLQ------------K----
    mgv1a001314m                      HEM-LIDR-AHLL---EESYEYI-----------------VHADL-DS-LR-A-GLF-MEF------------------KNEEAT-GPGVLREWFFLVCQAIF------------N----
    mgv1a000078m                      QKV-RVSR-NRIL---DSAAKVM-----------------EMYSS----QK-A-VLE-VEY------------------FGEVGS-GLGPTLEFYTLLSHELQ------------K----
    mgv1a000005m                      LRI-SVRR-AYIL---EDSYNQL-----------------RMRPS-QD-LK-G-RLN-VHF------------------QGEEGIDAGGLTREWYQLLSRVVF------------D----
    mgv11b024345m                     ALI-VIHR-DRIV---EDGYRQL-----------------AAQPT-HA-LK-G-VIR-VRFINQQG-------------LHEAGIDQDGVFKEFLEETIKKVF------------D----
    mgv1a000436m                      IEI-VIRR-DRIF---EDGMQQL-----------------NSLG--SK-LK-S-AIH-VSFVSESG-------------LPEAGLDYGGLSKEFLTDISKAAF------------S----
    mgv1a000163m                      KKI-LVHR-NKIL---ESAAHMM-----------------ELHSR----QK-V-VLE-VEY------------------SEEVGT-GLGPTLEFYTLVCHEFQ------------R----
    GSVIVT01003328001                 HEM-LIDR-SQLL---AESFEYI-----------------ARAER-ES-LH-G-GLF-MEF------------------KNEEAT-GPGVLREWFFLVCQEIF------------N----
    GSVIVT01009206001                 LRI-SVRR-AYIL---EDSYNQL-----------------RMRST-QD-LK-G-RLT-VHF------------------QGEEGIDAGGLTREWYQSLSRVIF------------D----
    GSVIVT01014698001                 LRI-SVRR-AYVL---EDSYNQL-----------------RLRPT-QE-LK-G-RLN-VQF------------------QGEEGIDAGGLTREWYQLLSRVIF------------D----
    GSVIVT01018731001                 QKV-RVSR-NRIL---DSARKVM-----------------EMYSS----QK-A-VLE-VEY------------------FGEVGT-GLGPTLEFYTLLSHDLQ------------K----
    GSVIVT01024033001                 QKV-RVSR-NRIL---DSAAKVM-----------------EMYSS----QK-A-VLE-VEY------------------FGEVGT-GLGPTLEFYTLLSHDLQ------------K----
    GSVIVT01025537001                 KKF-LVCR-DRIL---DSAAQMM-----------------NLHAC----QK-V-VLE-VEY------------------NEEVGT-GLGPTLEFYTLVCHEFQ------------K----
    GSVIVT01033734001                 VEV-VIRR-GHIV---EDGFQQL-----------------NSLG--SR-LK-S-CIH-VSFISECG-------------LPEAGLDYGGLFKEFLTDIAKAAF------------A----
    GSVIVT01034942001                 NRF-RIRR-DHIL---EDAFNQL-----------------SVLSE-DD-LR-G-LIR-ISFVNEFG-------------VEEAGIDGGGIFKDFMENITRAAF------------D----
    cassava4.1_000003m                LRI-SVRR-AYVL---EDSYNQL-----------------RMRPS-QD-LK-G-RLN-VQF------------------QGEEGIDAGGLTREWYQLLSRVIF------------D----
    cassava4.1_000080m                QKV-RVSR-NRIL---DSAAKVM-----------------EMYSS----QK-A-VLE-VEY------------------FGEVGT-GLGPTLEFYTLLSHDLQ------------K----
    cassava4.1_002295m                NRF-RIRR-DRIL---EDAYNQM-----------------SALSE-ED-LR-G-LIR-VTFVNEFG-------------VEEAGIDGGGIFKDFMENITRAAF------------D----
    cassava4.1_000006m                LRI-SVRR-AYIL---EDSYNQL-----------------RMRST-QD-LK-G-RLT-VHF------------------QGEEGIDAGGLTREWYQLLSRVIF------------D----
    cassava4.1_000011m                LRI-SVRR-AYIL---EDSYNQL-----------------RMRST-QD-LK-G-RLT-VHF------------------QGEEGIDAGGLTREWYQLLSRVIF------------D----
    cassava4.1_000177m                KKF-VVWR-DRIL---ESAAQMM-----------------DFYAN----VR-D-PIE-VVY------------------NGEVGS-GLGPTLEFYTLVSHEFQ------------K----
    Pp1s205_47V6.1                    QEV-VVKR-ATLL---TESFEQL-----------------AYVEP-EV-LQ-G-GIS-VEF------------------ASEEAT-GPGVLREWFCMICREIF------------N----
    Pp1s148_98V6.1                    QEV-VVKR-ATLL---TESFEQL-----------------AYVEP-EV-LQ-G-GIS-VEF------------------ATEEAT-GPGVLREWFSMICREIF------------N----
    Pp1s103_43V6.1                    TEI-AVRR-DHIV---EDGFSQL-----------------NALG--PK-LK-C-CIN-VSFVNELG-------------LAEAGLDYGGLFKEFLTDLAKAAF------------D----
    Pp1s42_128V6.2                    LRI-CVRR-AYVL---EDSYNQL-----------------RMRTP-DE-LK-G-RLT-VQF------------------QGEEGIDAGGLTREWYQLLSRVTF------------D----
    Pp1s263_1V6.1                     QKV-RVSR-QRIL---ESAAKVM-----------------ELYAG----HK-A-VLE-VEY------------------FGEVGT-GLGPTLEFYTLLSHELQ------------K----
    Pp1s263_20V6.1                    ISI-VVKR-DNLF---EDGLAQL-----------------NPLG--PR-LK-S-CIN-VSFENESV-------------LAAADLDHGGSLKELLTDLANTAF------------D----
    Pp1s15_454V6.1                    IEV-TVRR-DHIV---EDGFAQL-----------------NGLG--SK-LK-S-CVN-VSFVNELG-------------LKEAGLDYGGLFKEFLIDLAKAAF------------D----
    Pp1s67_251V6.1                    LQI-VVRR-DQVF---QDSYLQF-----------------VSLSD-DD-LR-N-PLS-VHF------------------VGEVGRDDGGVTRDWYSVLAKEIF------------N----
    Pp1s173_137V6.1                   TRI-KIRR-DHIT---EDAFAQL-----------------NGISA-EA-LK-G-TIR-VMFVNELG-------------VEEAGVDGGGIFKDFMEGITKTGF------------D----
    Pp1s116_90V6.1                    QKV-RVSR-QRIL---ESAAKVM-----------------ELYSG----HK-A-VLE-VEY------------------FGEVGT-GLGPTLEFYTLLSHELQ------------K----
    Pp1s138_130V6.1                   LRI-CVRR-AYVL---EDSYNQL-----------------RMRTP-DE-LK-G-RLT-VQF------------------QGEEGIDAGGLTREWYQLLSRVTF------------D----
    Pp1s229_59V6.1                    LIL-TVRR-SSLV---ADTLRQL-----------------E-Y-E-DD-LK-K-PLK-VIF------------------EGEAGVDEGGVTKEFFQLLIRELF------------N----
    Pp1s88_123V6.1                    IEI-AVRR-DHIV---EDGYAQL-----------------NGLG--SK-LK-S-SVN-VSFVDELG-------------LRETGLEHGGFSKDFLTDLAKEAF------------D----
    orange1.1g000286m                 QKV-RVSR-NRIL---DSAAKVM-----------------EMYSS----QK-A-VLE-VEY------------------FGEVGT-GLGPTLEFYTLLSRDLQ------------R----
    orange1.1g045956m                 IEI-VVRR-GHIV---EDGFRQL-----------------NSLG--SR-LK-S-SIH-VSFVSECG-------------LPEAGLDYGGLSKEFLTDISKSAF------------A----
    orange1.1g000014m                 LRI-SVRR-AYIL---EDSYNQL-----------------RMRST-QD-LK-G-RLT-VHF------------------QGEEGIDAGGLTREWYQLLSRVIF------------D----
    orange1.1g001688m                 SRF-RIRR-DHIL---EDAYSQM-----------------STMSE-ED-LR-G-AIR-VTFVNELG-------------VEEAGIDGGGIFKDFMENITRAAF------------D----
    orange1.1g000012m                 LRI-SVRR-AYVL---EDSYNQL-----------------RMRST-QD-LK-G-RLN-VHF------------------QGEEGIDAGGLTREWYQLLSRVIF------------D----
    AT4G12570.1                       HEM-LIDR-SNLL---SESFEYI-----------------VGASP-EA-LH-G-GLF-MEF------------------KNEEAT-GPGVLREWFYLVCQEIF------------N----
    AT4G38600.1                       QKV-RVSR-NRIL---DSAAKVM-----------------EMYSS----QK-A-VLE-VEY------------------FGEVGT-GLGPTLEFYTLLSHDLQ------------K----
    AT1G55860.1                       LRI-SVRR-AYVL---EDSYNQL-----------------RMRSP-QD-LK-G-RLN-VQF------------------QGEEGIDAGGLTREWYQLLSRVIF------------D----
    AT1G70320.1                       LRI-SVRR-AYVL---EDSYNQL-----------------RMRSP-QD-LK-G-RLN-VQF------------------QGEEGIDAGGLTREWYQLLSRVIF------------D----
    AT3G53090.1                       IEI-VVRR-GHVV---EDGFQQL-----------------NSIG--SR-LK-S-SIH-VSFVNESG-------------LPEAGLDYGGLSKEFLTDITKAAF------------A----
    AT3G17205.1                       NRF-RIRR-DHIL---EDAYNQM-----------------SALSE-DD-LR-S-SIR-VTFVNELG-------------VEEAGIDGGGIFKDFMEKITRAAF------------D----
    AT5G02880.1                       KKF-LACR-ENIL---ESAAKMM-----------------ELYGN----QK-V-VIE-VEY------------------SEEVGT-GLGPTLEFYTLVSRAFQ------------N----
    Si034011m                         IAI-VIRR-GHII---EDGYRQL-----------------NCLR--SK-LK-S-CIH-VSFVSECG-------------LPEAGLDYGGLSKEFLTDLSKTAF------------S----
    Si016079m                         QKV-RVSR-NRIL---DSAAKVM-----------------EMFSS----QR-A-VLE-VEY------------------FGEVGT-GLGPTLEFYTLLSHELQ------------S----
    Si013562m                         TAARDAHR-SRLL---SDSFGYI-----------------ALATP-RA-LRAA-ALV-VAF------------------KHEQAA-GPGVVREWFCLVCQALF------------N----
    Si013264m                         YEL-LVDR-ARLL---PDSFGYI-----------------VHATP-QE-LG-A-AMS-VAF------------------KHEQAT-GPGVLREWFCLVCQALF------------N----
    Si009242m                         HRF-KIRR-NRLL---EDAFDQL-----------------SLLSE-ED-LK-G-PIR-VSFINEHG-------------EEEAGIDGGGIFKDFMENITRAAF------------D----
    Si009164m                         QKV-RVSR-NRIL---DSAAKVM-----------------EMFSN----QK-A-VLE-VEY------------------FGEVGT-GLGPTSEFYTLLSHDLQ------------R----
    Si024055m                         KKY-RVTR-SAII---EGAVSMM-----------------TNHGP----SS-R-IIE-VEF------------------EGEVGT-GRGPTFEFYTTVSHELQ------------R----
    Si020966m                         KKF-KVDR-DNIL---VSAAKMM-----------------KSYAK----SN-A-LLE-VEY------------------KEEVGT-GLGPTMEFYTLISHEFQ------------K----
    Si020939m                         VRI-SVRR-AYIL---EDSYNQL-----------------RMRSP-QD-LK-G-RLT-VHF------------------QGEEGIDAGGLTREWYQSLSRVIF------------D----
    Si028891m                         HEM-LIDR-SHLL---DESFNYI-----------------AQAKH-NE-LR-G-GLF-MEF------------------KNEEAT-GPGVLREWFCLVCQALF------------S----
    Si028637m                         LRI-SVRR-AYVL---EDSYNQL-----------------RLRRT-QD-LK-G-RLT-VQF------------------QGEEGIDAGGLTREWYQLLSRVIF------------D----
    Thhalv10019984m                   NRF-RIRR-DHIL---EDAYNQM-----------------SALSE-DD-LR-G-SIR-VTFVNELG-------------VEEAGIDGGGIFKDFMEKITRAAF------------D----
    Thhalv10011172m                   LRI-SVRR-AYVL---EDSYNQL-----------------RMRSP-QD-LK-G-RLN-VQF------------------QGEEGIDAGGLTREWYQLLSRVIF------------D----
    Thhalv10011171m                   LRI-SVRR-AYVL---EDSYNQL-----------------RMRSP-QD-LK-G-RLN-VQF------------------QGEEGIDAGGLTREWYQLLSRVIF------------D----
    Thhalv10024192m                   QKV-RVSR-NRIL---DSAAKVM-----------------EMYSS----QK-A-VLE-VEY------------------FGEVGT-GLGPTLEFYTLLSHDLQ------------K----
    Thhalv10028412m                   HEM-LIDR-SNLL---SESFEYI-----------------ASATP-GA-LH-G-GLF-MEF------------------KNEEAT-GPGVLREWFYLVCQEIF------------N----
    Thhalv10012430m                   KKF-LACR-ENIL---ESAAKMM-----------------ELYGK----QK-V-VVE-VEY------------------NEEVGT-GLGPTLEFYTLVSRAFQ------------N----
    Thhalv10010078m                   IEI-VVRR-GHVV---EDGFRQL-----------------NSIG--SR-LK-S-SIH-VSFVNESG-------------LPEAGLDYGGLSKEFLTDITKAAF------------A----
    Ciclev10000001m                   LRI-SVRR-AYIL---EDSYNQL-----------------RMRST-QD-LK-G-RLT-VHF------------------QGEEGIDAGGLTREWYQLLSRVIF------------D----
    Ciclev10004231m                   SRF-RIRR-DHIL---EDAYSQM-----------------STMSE-ED-LR-G-AIR-VTFVNELG-------------VEEAGIDGGGIFKDFMENITRAAF------------D----
    Ciclev10007219m                   LRI-SVRR-AYVL---EDSYNQL-----------------RMRST-QD-LK-G-RLN-VHF------------------QGEEGIDAGGLTREWYQLLSRVIF------------D----
    Ciclev10010897m                   KKF-LVCR-NRIL---ESATQMM-----------------DQHAC----NR-T-LVE-VEY------------------DEEVGS-GLGPTLEFYTLVSQEFQ------------K----
    Ciclev10010940m                   IEI-VVRR-GHIV---EDGFRQL-----------------NSLG--SR-LK-S-SIH-VSFVSECG-------------LPEAGLDYGGLSKEFLTDISKSAF------------A----
    Ciclev10027670m                   QKV-RVSR-NRIL---DSAAKVM-----------------EMYSS----QK-A-VLE-VEY------------------FGEVGT-GLGPTLEFYTLLSRDLQ------------R----
    Ciclev10014213m                   HEM-LIDR-SQLL---AESFEYI-----------------ARAEP-EA-LR-G-GLF-MEF------------------KNEEAT-GPGVLREWFFLVCQALF------------N----
    GRMZM2G034622_T02                 ----------------------------------------------------------------------------------------------MENITRAAF------------D----
    GRMZM2G124297_T01                 KKY-RVAR-SAII---EGAVSVM-----------------TNHGP----SN-R-IIE-VEF------------------EGEIGT-GRGPTFEFYSTVSHELQ------------R----
    GRMZM2G411536_T03                 VRI-SVRR-SYIL---EDSYNQL-----------------RMRSP-QD-LK-G-RLT-VHF------------------QGEEGIDAGGLTREWYQSLSRVIF------------D----
    GRMZM2G181378_T01                 HEM-LIDR-SHLL---DESFNYI-----------------AQAEQ-AE-LH-G-ALF-MEF------------------KNEEAT-GPGVLREWFCLVCQALF------------S----
    GRMZM2G049141_T01                 QKV-RVSR-NRIL---DSAAKVM-----------------EMFSN----QK-A-VLE-VEY------------------FGEVGT-GLGPTLEFYTLLSRELQ------------R----
    GRMZM2G080439_T01                 HEM-LIDR-SHLL---DESFNYI-----------------AQAKQ-TE-LR-G-GLF-MEF------------------KNEEAT-GPGVLREWFCLVCQALF------------S----
    GRMZM2G021299_T01                 LHI-SVRR-AYVL---DDSYNQL-----------------RLRRT-QD-LK-G-RLT-VQF------------------QGEEGIDAGGLTREWYQLLSRVIF------------D----
    GRMZM2G328988_T01                 KKF-KVDR-DDIL---VSAAKMM-----------------KSHAK----SN-A-LLE-VEY------------------KEEVGT-GLGPTMEFYTLISHEFQ------------K----
    GRMZM2G331368_T02                 VRI-SVRR-PYIL---EDSYNQL-----------------RMRSP-QD-LK-G-RLT-VQF------------------QGEEGIDAGGLTREWYQSISRVIV------------D----
    GRMZM2G461948_T01                 IEI-VIRR-GHII---DDGYRQL-----------------NCLR--SK-LK-S-CIH-VSFVSECG-------------LPEAGLDYGGLSKEFLTDLSKSAF------------S----
    GRMZM2G374574_T01                 QKV-RVSR-NRIL---DSAAKVM-----------------EMFSS----QR-A-VLE-VEY------------------FGEVGT-GLGPTLEFYTLLSHELQ------------C----
    Carubv10016604m                   IEI-VVRR-GHVV---EDGFRQL-----------------NSIG--SR-LK-S-SIH-VSFVNESG-------------LPEAGLDYGGLSKEFLTDITKAAF------------A----
    Carubv10011657m                   LRI-SVRR-AYVL---EDSYNQL-----------------RMRSP-QD-LK-G-RLN-VQF------------------QGEEGIDAGGLTREWYQLLSRVIF------------D----
    Carubv10007210m                   LYI-ILDR-PNLL---TESLEQM-----------------ANASP-TSLLH-G-DLS-VYF------------------EEETAV-GEGVLREWFYLVCEKLF------------E----
    Carubv10003974m                   QKV-RVSR-NRIL---DSAAKVM-----------------EMYSS----QK-A-VLE-VEY------------------FGEVGT-GLGPTLEFYTLLSHDLQ------------K----
    Carubv10012881m                   NRF-RIRR-DHIL---EDAYNQM-----------------SALSE-DD-LR-G-SIR-VTFVNELG-------------VEEAGIDGGGIFKDFMEKITRAAF------------D----
    Carubv10000054m                   KKF-LAYR-EKVL---ESAAQMM-----------------ELYGN----QK-V-IIE-VEY------------------NEEVGT-GLGPTLEFYTLVSRAFQ------------N----
    Carubv10000186m                   HEM-LIDR-SNLL---SESFEYI-----------------VGASP-EA-LH-G-GLF-MEF------------------KNEEAT-GPGVLREWFYLVCQEIF------------N----
    Carubv10025730m                   LRI-SVRR-AYIL---EDSYNQL-----------------RMRTT-QE-LK-G-RLT-VHF------------------QGEEGIDAGGLTREWYQLLSRVIF------------D----
    Bradi2g34820.1                    HRF-KIRR-SRLL---EDAFDQL-----------------SLLSE-ED-LK-G-PIR-VAFVNEHG-------------VEEAGIDGGGIFKDFMENITRAAF------------D----
    Bradi2g37870.1                    KKH-KVMR-GNIL---EDAASMM-----------------STHAS----SN-E-TLE-VVF------------------EGEVGT-GRGPTFEFYTTVSHELQ------------R----
    Bradi2g22927.2                    KKF-KVDR-DDIL---VSTAKIM-----------------QSYAR----SN-A-VLE-VEY------------------EEEVGT-GLGPTMEFYTLISHEFQ------------K----
    Bradi4g07997.2                    VRI-SVRR-AYIL---EDSYNQL-----------------RMRSP-QD-LK-G-RLT-VHF------------------QGEEGIDAGGLTREWYQLLSRVIF------------D----
    Bradi4g33520.1                    HEM-LIDR-SHLL---DESFEYI-----------------TQARP-SE-LH-S-GLF-MEF------------------KNEEAT-GPGVLREWFCMVCQALF------------S----
    Bradi1g12340.2                    IEI-VIRR-GHIV---EDGYRQL-----------------NCLR--SK-LK-S-CIH-VSFVSECG-------------LPEAGLDYGGLSKEFLTDVSKAAF------------S----
    Bradi5g04567.1                    QKV-RVSR-NRIL---DSAAKVM-----------------EMFSS----QK-A-VLE-VEY------------------FGEVGT-GLGPTLEFYTLLSHDLQ------------R----
    Bradi3g00350.1                    QKV-RVSR-NRIL---DSATKVM-----------------EMFSS----QR-A-VLE-VEY------------------FGEVGT-GLGPTLEFYTLLGHELQ------------S----
    Aquca_017_00766.1                 KKF-KVCR-SRIL---ESAAQAM-----------------SSHVG----RK-A-ILE-VEY------------------PEEVGT-GQGPTMEFFTLVSQEFQ------------K----
    Aquca_006_00259.1                 QKV-RVSR-NRIL---DSAAKVM-----------------EMYSS----QK-A-VLE-VEY------------------FGEVGT-GLGPTLEFYTLLSHDLQ------------K----
    Aquca_028_00189.1                 HEM-LIDR-SQLL---EESFAYI-----------------SRADP-ES-FR-S-GLF-MEF------------------KNEEAT-GPGVLREWFYLVCQAIF------------N----
    Aquca_027_00123.1                 KKF-QVCR-SDIL---NSATKMM-----------------DQHAR----QK-A-ILE-VEY------------------DEEVGS-GLGPTMEFYTLVSHEFQ------------K----
    Aquca_007_00539.1                 LRI-SVRR-AYIL---EDSYNQL-----------------RMRST-QD-LK-G-RLT-VHF------------------QGEEGIDAGGLTREWYQLLSRVIF------------D----
    Aquca_003_00437.1                 NRI-SIRR-NRIF---EDAFNKL-----------------GEMSG-AD-LR-G-LIR-VSYVNEFG-------------VEEAGIDGGGIFKDFMENITRTAF------------D----
    Aquca_019_00105.1                 KEI-VIRR-SHIV---EDGFKQL-----------------NSLG--SM-LK-S-RIH-VSFVSECG-------------LPEAGLDYGGLSKEFLTDISKAAF------------N----
    MDP0000264736                     HEM-LIDR-SQIL---AESFEYI-----------------RRAEP-ES-LH-G-GLF-MEF------------------KNEEAT-GPGVLREWFFLVCQAIF------------N----
    MDP0000320720                     KKF-LVFR-NQIL---DSAAQMM-----------------DLHAS----QK-V-LLE-VEY------------------SEEVGT-GLGPTLEFYTLVSHEFQ------------K----
    MDP0000142676                     HEM-LIDR-SQIL---AESFEYI-----------------GHAEP-ES-LH-G-GLF-MEF------------------KNEEAT-GPGVLREWFFLVCQAIF------------N----
    MDP0000318443                     LRI-SVRR-AYIL---EDSYNQL-----------------RMRST-QD-LK-G-RLT-VHF------------------QGEEGIDAGGLTREWYQLLSRVIF------------D----
    MDP0000206447                     LRI-SVRR-AYVL---EDSYNQL-----------------RMRPT-HD-MK-G-RLN-VQF------------------QGEEGIDAGGLTREWYQLLSRVIF------------D----
    MDP0000196216                     VEI-VVRR-GHIV---EDGFRQL-----------------NSLG--S---------------SECG-------------LPEAGLDYGGLSKEFLTDISKAAF------------S----
    MDP0000186793                     NRF-RIRR-DRIL---EDAYDQM-----------------SALSE-DD-LR-G-PIR-VTFVNEFG-------------VEEAGIDGGGIFKDFMENITQAAF------------D----
    MDP0000822588                     KKF-LVFR-NQIL---DSAAQMM-----------------DLHAR----QK-V-LLE-VEY------------------NEEVGT-GLGPTLEFYTLVSHEFQ------------K----
    MDP0000924418                     VEI-VVRR-GHIV---EDGFQQL-----------------NSLG--SR-LK-S-SIH-VSFVSECG-------------LPEAGLDYGGLSKEFLTDISKAAF------------A----
    MDP0000320505                     QKV-RVSR-NRIL---DSAAKVM-----------------EMYSS----QK-S-VLE-VEY------------------FGEVGT-GLGPTLEFYTLLSHDLQ------------R----
    MDP0000307848                     LRI-SVRR-AYVL---EDSYNQL-----------------RMRPT-LD-MK-G-RLN-VQF------------------QGEEGIDAGGLTREWYQLLSRVIF------------D----
    MDP0000301275                     QKV-RVSR-NRIL---DSAAKVM-----------------EMYSS----QK-S-VLE-VEY------------------FGEVGT-GLGPTLEFYTLLSHDLQ------------R----
    MDP0000317971                     LRI-SVRR-AYIL---EDSYNQL-----------------RMRSA-ED-LK-G-RLT-VHF------------------QGEEGIDAGGLTREWYQLLSRVIF------------D----
    Bra022201                         NRF-RIRR-DHIL---EDAYNQM-----------------SALSE-DD-LR-G-SIR-VTFVNELG-------------VEEAGIDGGGIFKDFMEKITRAAF------------D----
    Bra028860                         KKF-LACR-EKIL---ESAAKMM-----------------ELHGT----QK-V-AVE-VAY------------------SEEVGT-GLGPTLEFYTLVSRAFQ------------N----
    Bra038022                         LRI-SVRR-AYML---EDSYNQL-----------------RMRSL-QD-LR-G-RLN-VQF------------------QGEEGVDAGGLTREWYQLVSRVIF------------D----
    Bra021231                         NRF-RIRR-DHIL---DDAYNQM-----------------SALSE-DD-LR-G-PIR-VTFVNELG-------------VEEAGIDGGGIFKDFMEKITLAAF------------D----
    Bra005748                         KKF-LACR-ETIL---ESASKMM-----------------ELHGN----QK-V-VIE-VEY------------------SEEVGT-GLGPTLEFYTLVSRAFQ------------N----
    Bra000779                         HEM-LIDR-SQVL---KESYVYI-----------------SQASP-AG-LH-G-ALF-MEF------------------KNEEAT-GPGVLREWFYLVCQEIF------------S----
    Bra029461                         HEM-LIDR-SNLF---AESFEYI-----------------SGATP-GS-LH-S-GLF-MEF------------------KNEEAT-GPGVLREWFYLVCQEIF------------N----
    Bra027850                         LRI-SVRR-AYIL---EDSYNQL-----------------RMRST-QE-LK-G-RLT-VHF------------------QGEEGIDAGGLTREWYQLLSRVIF------------D----
    Bra040685                         IEI-VVRR-GHVV---EDGFRQL-----------------NSIG--SR-LK-S-SIH-VSFVNESG-------------LPEAGLDYGGLSKEFLTDITKAAF------------A----
    Bra010737                         QKV-RVSR-NRIL---DSAAKVM-----------------EMYSS----QK-A-VLE-VEY------------------FGEVGT-GLGPTLEFYTLLSHDLQ------------K----
    Medtr2g025830.1                   YEM-LIDR-SQLL---AESFEYI-----------------SQANS-TS-LE-G-GLF-MEF------------------RNEEGT-GPGVVREWLVLVCQEIF------------N----
    Medtr2g025950.1                   YEM-LIDR-SQVL---AESFGYI-----------------SQAMP-RS-LQ-G-DLL-MAF------------------KNEKAT-GPGVLREWFVLVCQEIF------------N----
    Medtr2g025810.1                   YEM-LIDR-SQVL---AESFEYI-----------------SRAMP-KS-LQ-G-DLF-MAF------------------KNEKAT-GPGVLREWFVLVCQEIF------------N----
    Medtr2g033040.1                   NRF-RIRR-DHIL---EDAYNSM-----------------SQLSE-DD-LR-G-LIR-VTFVNEFG-------------VEEAGIDGGGIFKDFMENITRASF------------D----
    Medtr2g025790.1                   YEM-LIDR-SQVL---AESFEYM-----------------SRAKA-KS-LQ-G-GIF-MAF------------------KNEKAT-GPGVLREWFVLVCREIF------------N----
    Medtr2g025930.1                   YEM-LIDR-SQLL---AESFEYI-----------------SQANS-TS-LE-G-GLF-MEF------------------RNEEGT-GPGVVREWLVLVCQEIF------------N----
    Medtr7g100670.1                   KKC-LVFR-DRIL---ESAAQMM-----------------NQNAS----RK-V-VLE-VEY------------------DGEVGS-GFGPTLEFYTLVCKEFQ------------N----
    Medtr5g066710.1                   LRI-SVRR-AYVL---EDSYNQL-----------------RMRPT-QD-LK-G-RLT-VHF------------------QGEEGIDAGGLTREWYQLLSRVIF------------D----
    Medtr4g073370.1                   QKV-RVSR-NRIL---DSAAKVM-----------------ELYSS----QK-A-VLE-VEY------------------FGEVGT-GLGPTLEFYTLLSHDLQ------------K----
    Medtr4g133120.1                   HEM-LIDR-SQLL---TESFEYI-----------------ARADP-ES-LR-A-GLF-MEF------------------KNEEAT-GPGVLREWFLLVCQALF------------N----
    Vocar20002255m                    LDI-AVRR-TSLL---ADAAQQL-----------------SCRPA-HH-LK-R-PLR-VSFVSL-G-------------VAEEGVDQGGVSREFFQLLVAEIF------------Q----
    Vocar20010178m                    LRL-AVRR-EHVF---EDSFYQL-----------------RGRPA-EE-MK-L-KLN-VTF------------------QGEEGIDAGGVTREWYQVMAREMF------------N----
    Vocar20006334m                    TNV-TVRR-SAAF---WDAFEAFRGAGLPA----------E---P-MC-YKYF-----PAFVDVYSQDGSGRGGSGRKPAVEAGE-GHGPRKEFFSLAGQDMAGQAHQQQQ----Q-RSQ
    Vocar20007555m                    HKL-EIRR-NRCF---KDSVAIF-----------------AGKGH-AV-WR-Q-PLK-VTF------------------IGEAGMDSGGVTREWFSTLSSAIS------------R----
    Vocar20012583m                    QKV-RVSR-KRIL---ESAAKVM-----------------ELYAR----SR-A-VLE-LEY------------------FGEVGT-GLGPTLEFYTLLCHELQ------------R----
    Vocar20003001m                    NRFVAIRR-DRLL---FDGFDNL-----------------NSLG--DR-LR-G-RVR-IAYIDAHG-------------AQEAGVDGGGLFKDFMEELMREGL------------S----
    Vocar20004069m                    CIV-RVRR-THLV---EDALEEL-----------------GRQVK-SD-LL-K-PLR-VHF------------------IGEEGIDAGGVKKEFFALLMERLL------------R----
    Vocar20000780m                    ------RR-----------------------------------------------------------------------GSEAGLDFGGLQKELLERVVSAGL------------D----
    Vocar20004842m                    LVL-RVRRGPYLV---QDTLIQI-----------------HRAKE-TDSLK-K-PLKVVKF------------------IGEEGVDEGGVAKEFFQLLVRQLF------------N----
    Vocar20014908m                    PQV-LAYR-SSIT---ESSYYQV-------------------MDA-EA-LP-Y-GVN-VRF------------------EDEQEAEGMGVVREWLSQIAADIF------------S----
    Lus10032589                       LRI-SVRR-AYVL---EDSYNQL-----------------RMRPT-PD-LR-G-RLN-VQF------------------QGEEGIDAGGLTREWYQILSRVLF------------D----
    Lus10035589                       VEI-VVRR-GHIV---EDGYRQL-----------------NYLG--PR-LK-S-SIH-VSFVSESG-------------LPEAGLDYGGLSKEFLTDISKTAF------------S----
    Lus10005068                       QKV-RVSR-NRIL---DSAAKVM-----------------EMYSS----QK-A-VLE-VEY------------------FGEVGT-GLGPTLEFYTLLSHDLQ------------K----
    Lus10010493                       HEM-LIDR-ANLL---EESFAYI-----------------GRAEP-DS-LH-G-GLF-MEF------------------KNEEAT-GPGVLREWFLLVTQAIF------------N----
    Lus10027841                       QKV-RVSR-NRIL---DSAAKVM-----------------EMYSS----QK-A-VLE-VEY------------------FGEVGT-GLGPTLEFYTLLSHDLQ------------K----
    Lus10019908                       KKF-LVSR-DQIL---ESAARMM-----------------ELYNN----VK-T-PIE-IEY------------------NEEVGT-GLGPTLEFYTLVAREFQ------------K----
    Lus10032830                       LRI-SVRR-AYIL---EDSYNQL-----------------RMRST-QD-LK-G-RLT-VHF------------------QGEEGIDAGGLTREWYQLLSRVIF------------D----
    Lus10017098                       NRF-RIRR-DRIL---EDAYNQM-----------------SALSE-ED-LR-G-LIR-VAFVNELG-------------VEEAGIDGGGIFKDFMENITRAAF------------D----
    Lus10002605                       LRI-SVRR-AYIL---EDSYNQL-----------------RMRST-QD-LK-G-RLT-VHF------------------QGEEGIDAGGLTRELYQLLSRGFF------------D----
    Lus10008636                       VEI-VVRR-GHIV---EDGYRQL-----------------NYLG--PR-LK-S-SIH-VSFVSESG-------------LPEAGLDYGGLSKEFLTDISKTAF------------S----
    Eucgr.A01178.1                    KKF-LVHR-DRIM---DSAIQMM-----------------DLYAR----QR-V-ALE-VEF------------------DDEVGT-GLGPTLEFYTLISHEFQ------------K----
    Eucgr.A01586.1                    IEI-VIRR-GNIV---EDGFRQL-----------------NTLG--SR-LK-S-SIH-VSFVSECG-------------LPEAGLDYGGLSKEFLTDLSKAAF------------S----
    Eucgr.B03986.1                    NRF-RIRR-DHIL---EDAYNQM-----------------SALSE-ED-LR-G-LIR-VSFVNELG-------------VEEAGIDGGGIFKDFMENITRAAF------------D----
    Eucgr.D01414.1                    HEM-LIDR-SQLL---SESFEYI-----------------ANAEA-ES-LH-G-GIF-MEF------------------KNEEAT-GPGVLREWFVLVCQAIF------------N----
    Eucgr.D01416.1                    HEM-LIDR-SQLL---SESFDYI-----------------ANAKA-ES-LH-G-GIL-MEF------------------KNEEAT-GPGVLREWFVLVCQAIF------------N----
    Eucgr.F02160.1                    LRI-SVRR-AYIL---EDSYNQL-----------------RLRST-QD-LK-G-RLT-VHF------------------QGEEGIDAGGLTREWYQLLSRVIF------------D----
    Eucgr.I01410.2                    QKV-RVSR-NRIL---DSAAKVM-----------------EMYSS----QK-A-VLE-VEY------------------FGEVGT-GLGPTLEFYTLLSHDLQ------------K----
    Pavirv00038038m                   VRI-SVRR-AYIL---EDSYNQL-----------------RMRSP-QE-LK-G-RLT-VHF------------------QGEEGIDAGGLTREWYQSLSRVIF------------D----
    Pavirv00031244m                   QKV-RVSR-NRIL---DSAAKVM-----------------EMFSS----QR-A-VLE-VEY------------------FGEVGT-GLGPTLEFYTLLSHELQ------------S----
    Pavirv00010575m                   HEM-LIDR-SHLL---DESFNYI-----------------AQARH-SE-LR-G-GLF-MEF------------------KNEEAT-GPGVLREWFCLVCQALF------------S----
    Pavirv00004902m                   HEM-LIDR-GRLL---PDSFGYI-----------------AHATP-QE-LR-A-VLS-VAF------------------KHEQAT-GPGVLREWFCLVCQALF------------N----
    Pavirv00020428m                   KKY-RVSR-SAIL---EGAVSMM-----------------TNHDP----SC-R-IVE-VEF------------------EGEVGT-GRGPTFEFYTTVSHELQ------------R----
    Pavirv00067430m                   HEM-LIDR-SHLL---DESFNYI-----------------AQARH-SE-LR-G-GLF-MEF------------------KNEEAT-GPGVLREWFCLVCQALF------------S----
    Pavirv00058663m                   LRI-SVRR-AYVL---EDSYNQL-----------------RLRRT-QD-LK-G-RLT-VQF------------------QGEEGIDAGGLTREWYQLLSRVIF------------D----
    Pavirv00067620m                   QKV-RVSR-NRIL---DSAAKVM-----------------EMFSS----QR-A-VLE-VEY------------------FGEVGT-GLGPTLEFYTLLSHELQ------------S----
    Pavirv00029557m                   VRI-SVRR-AYIL---EDSYNQL-----------------RMRSP-QE-LK-G-RLT-VHF------------------QGEEGIDAGGLTREWYQSLSRVIF------------D----
    Pavirv00023469m                   HRF-KIRR-NRLL---EDAFDQL-----------------SLLSE-ED-LK-G-PIR-VSFINEHG-------------EEEAGIDGGGIFKDFMENITRAAF------------D----
    Pavirv00024250m                   KKF-KVDR-DDIL---VSAAKMM-----------------QSYAK----SN-A-LLE-VEY------------------NEEVGT-GLGPTMEFYTLISHEFQ------------K----
    Pavirv00023205m                   KKF-KVDR-DDIL---VSAAKMM-----------------KSYAK----SN-A-LLE-VEY------------------KEEVGT-GLGPTMEFYTLISHEFQ------------K----
    Pavirv00029138m                   KKY-RVSR-SAIL---EGAVSMM-----------------TNHGP----SS-R-IVE-VEF------------------KGEVGT-GRGPTFEFYTTVSHELQ------------R----
    LOC_Os03g47949.1                  IEI-VIRR-GHIV---EDGYRQL-----------------NCLG--SK-LK-S-CIH-VSFVSECG-------------LPEAGLDYGGLSKEFLTDLSKAAF------------S----
    LOC_Os02g01170.1                  QKV-RVSR-NRIL---DSAAKVM-----------------EMFSS----QR-A-VLE-VEY------------------FGEVGT-GLGPTLEFYTLLSHELQ------------S----
    LOC_Os09g07900.1                  LRI-SVRR-AYVL---EDSYNQL-----------------RLRRS-QD-LK-G-RLT-VQF------------------QGEEGIDAGGLTREWYQLLSRVIF------------D----
    LOC_Os12g24080.1                  VRI-SVRR-AYIL---EDSYNQL-----------------RMRSP-QD-LK-G-RLT-VHF------------------QGEEGIDAGGLTREWYQLLSRVIF------------D----
    LOC_Os05g38830.1                  KKF-KVDR-DNIL---VSAAKVM-----------------QSHAR----SN-A-MLE-VEY------------------EEEVGT-GLGPTMEFYTLISHEFQ------------K----
    LOC_Os05g03100.1                  KKY-SVTR-SKIL---EDASSML-----------------NKHGS----DT-K-FIE-VEF------------------DGEVGT-GRGPTFEFYTTVSHELQ------------R----
    LOC_Os05g06690.1                  HRF-KIRR-NRLL---EDAFDQL-----------------SMLSE-ED-LK-G-PIR-VVFVNEHG-------------VEEAGIDGGGIFKDFMENITRAAF------------D----
    PGSC0003DMT400075387              YEM-LIDR-SDLL---EASFEYI-----------------VDQDP-AL-LR-G-DLL-MQF------------------KHEEAI-GSGVLREWFFLVCRELF------------N----
    PGSC0003DMT400021802              HEM-LIDR-SQLL---SESFEYI-----------------AHADP-ES-LR-G-GLF-MEF------------------KSEEAT-GPGVLREWFFLVCRAIF------------N----
    PGSC0003DMT400031190              QKV-RVSR-NRIL---DSAAKVM-----------------EMYSS----QK-A-VLE-VEY------------------FGEVGT-GLGPTLEFYTLISHDLQ------------E----
    PGSC0003DMT400072624              YCM-FIDR-SRLL---ENSFEYI-----------------GNATP-KN-LQ-G-CLF-IKF------------------KHEEAT-GPGVLREWFLLVCQAMF------------N----
    Glyma14g36180.1                   LRI-SVRR-AYVL---EDSYNQL-----------------RMRST-QD-LK-G-RLT-VHF------------------QGEEGIDAGGLTREWYQLLSRVIF------------D----
    Glyma02g38020.2                   LRI-SVRR-AYVL---EDSYNQL-----------------RLRST-QD-LK-G-RLT-VHF------------------QGEEGIDAGGLTREWYQLLSRVIF------------D----
    Glyma12g03640.1                   QKV-RVSR-NRIL---DSAAKVM-----------------ELYSS----QK-A-VLE-VEY------------------FGEVGT-GLGPTLEFYTLLSHDLQ------------K----
    Glyma11g11490.1                   QKV-RVSR-NRIL---DSAAKVM-----------------ELYSS----QK-A-VLE-VEY------------------FGEVGT-GLGPTLEFYTLLSHDLQ------------K----
    Glyma06g00600.1                   QKV-RVSR-NRVL---DSAAKVM-----------------GMYSS----QK-A-VLE-VEY------------------FGEVGT-GLGPTLEFYTILSHDLQ------------Q----
    Glyma06g10360.1                   LRI-SVRR-AYVL---EDSYNQL-----------------RMRST-QD-LK-G-RLT-VHF------------------QGEEGIDAGGLTREWYQLLSRVIF------------D----
    Glyma04g00530.1                   QKV-RVSR-NRVL---DSAAKVM-----------------EMYSS----QK-A-VLE-VEY------------------FGEVGT-GLGPTLEFYTILSHDLQ------------K----
    Glyma04g10481.1                   LRI-SVRR-AYVL---EDSYNQL-----------------RMRST-QD-LK-G-RLT-VHF------------------QGEEGIDAGGLTREWYQLLSRVIF------------D----
    Glyma08g09270.3                   LRI-SVRR-AYIL---EDSYNQL-----------------RMRPT-QD-LK-G-RLN-VQF------------------QGEEGIDAGGLTREWYQLLSRVIF------------D----
    Glyma17g01210.2                   HEM-LIDR-SQLL---TESFEYI-----------------ARAEP-ES-LH-A-GLF-MEF------------------KNEEAT-GPGVLREWFLLVCQAIF------------N----
    Glyma17g04180.1                   NRF-RIQR-DHIL---EDAYNQM-----------------SQLTE-DS-LR-G-SIR-VTFVNEFG-------------VEEAGIDGGGIFKDFMENITRAAF------------D----
    Glyma13g19981.1                   KKF-LVHR-DRIL---ESAAQMM-----------------DLHAS----NK-V-VLE-VEY------------------DEEVGT-GLGPTLEFYTLVCQEFQ------------K----
    Glyma05g26360.1                   LRI-SVRR-AYIL---EDSYNQL-----------------RMRPT-QD-LK-G-RLN-VQF------------------QGEEGIDAGGLTREWYQLLSRVIF------------D----
    Glyma19g37310.1                   IEI-VIRR-GHIV---EDGFRQL-----------------NSLG--SR-LK-S-SIH-VSFVSECG-------------LLEAGLDYGGLSKEFLTDISKAAF------------S----
    Glyma15g14591.1                   NRF-RIRR-NHIL---EDAYNQM-----------------SQLSE-DD-LR-G-LIR-VAFVNELG-------------VEEAGIDGGGIFKDFMENITRAAF------------D----
    Glyma03g34650.2                   IEI-VVRR-GHIV---EDGFRQL-----------------NSLG--SR-LK-S-SIH-VSFVSECG-------------LLEAGLDYGGLSKEFLTDISKSAF------------S----
    Glyma10g05620.3                   KKF-LVHR-DRIL---ESAAQMM-----------------DLHAS----NK-V-VLE-VEY------------------DEEVGT-GLGPTLEFYTLVCQEFQ------------K----
    Glyma07g36390.1                   NRF-RIKR-DRIL---EDAYNQM-----------------SQLTE-DS-LR-G-SIR-VTFVNEFG-------------VEEAGIDGGGIFKDFMENITRAAF------------D----
    Glyma07g39546.1                   HEM-LIDR-SQLL---TESFEYI-----------------ARAEP-DS-LH-A-GLF-MEF------------------KNEEAT-GPGVLREWFLLVCQAIF------------N----
    Gorai.010G033100.1                LRI-SVRR-AYIL---EDSYNQL-----------------RMRST-QD-LK-G-RLT-VHF------------------QGEEGIDAGGLTREWYQLLSRVIF------------D----
    Gorai.010G186800.1                HEM-LIDR-SQLL---SESFEYI-----------------AHADP-ES-LH-A-GLF-MEF------------------KNEEAT-GPGVLREWFFLVCQAIF------------N----
    Gorai.009G278900.1                LRI-SVRR-AYIL---EDSYNQL-----------------RLRST-QD-LK-G-RLT-VHF------------------QGEEGIDAGGLTREWYQLLSRVIF------------D----
    Gorai.009G228200.1                LRI-SVRR-AYIL---EDSYNQL-----------------RMRTT-QD-LK-G-RLT-VHF------------------QGEEGIDAGGLTREWYQLLSRVIF------------D----
    Gorai.009G183200.1                HEM-LIDR-SQLL---AESFEYI-----------------ARVDP-ES-LH-A-GLF-MEF------------------KNEEAT-GPGVLREWFLLVCQAIF------------N----
    Gorai.009G420400.1                KKF-LVSR-DQIL---DSATRMM-----------------DLHAR----HK-G-LLE-VEY------------------NEEVGT-GLGPTLEFYTLVSHEFQ------------K----
    Gorai.002G100900.1                LRI-SVRR-AYVL---EDSYNQL-----------------RMRPT-QD-LK-G-RLN-VQF------------------QGEEGIDAGGLTREWYQLLSRVIF------------D----
    Gorai.002G196900.1                QKV-RVSR-NRIL---DSAAKVM-----------------EMYSS----QK-A-VLE-VEY------------------FGEVGT-GLGPTLEFYTLLSHDLQ------------K----
    Gorai.002G245000.1                IEI-VIRR-GHVI---EDGFRQL-----------------NSLG--SR-LK-S-SIH-VSFVSECG-------------LPEAGLDYGGLSKEFLTDISKAAF------------A----
    Gorai.002G003200.1                QKV-RISR-DRIL---DSAAKVM-----------------KMYSV----QK-A-VLE-VEY------------------FGEVGT-GSGPTLEFYTLLSHELQ------------K----
    Gorai.011G204200.1                NRF-RIRR-DHIL---EDAYNQM-----------------SQLSE-ED-LR-G-LIR-VTFVNEFG-------------VEEAGIDGGGIFKDFMENITRAAF------------D----
    Gorai.008G035900.1                QKV-RVSR-NRIL---DSAAKVM-----------------EMYSS----QK-T-VLE-VEY------------------FGEVGT-GLGPTLEFYTLLSHDLQ------------K----
    Gorai.006G265700.1                NRF-RIRR-DHIL---EDAYNQM-----------------SALSE-ED-LR-G-LIR-VTFVNEFG-------------VEEAGIDGGGIFKDFMENITRAAF------------D----
    Potri.010G150000.3                NRF-RIRR-DHIL---EDAYNQM-----------------SALSE-ED-LR-G-LIR-VSFINEFG-------------VEEAGIDGGGIFKDFMENITRAAF------------D----
    Potri.009G134300.1                QKV-RVSR-NRIL---DSAAKVM-----------------EMYSS----QK-A-VLE-VEY------------------FGEVGT-GLGPTLEFYTLLSHDLQ------------K----
    Potri.004G174700.1                QKV-RVSR-NRIL---DSAAKVM-----------------DMYSS----QK-A-VLE-VEY------------------FGEVGT-GLGPTLEFYTLLSHDLQ------------K----
    Potri.011G094100.1                LRI-SVRR-AYVL---EDSYNQL-----------------RMRPT-QD-LR-G-RLN-VQF------------------QGEEGIDAGGLTREWYQLLSRVVF------------D----
    Potri.006G132000.1                KKF-LVLR-DRVL---ESAAQMM-----------------DSYAH----VK-A-PIE-VEY------------------NEEVGT-GLGPTLEFYTLVSREFQ------------K----
    Potri.006G011700.1                HEM-LIDR-SQLL---AESFEYI-----------------VHAES-DT-LH-V-GLF-MEF------------------KNEEAT-GPGVLREWFFLVTQAIF------------D----
    Potri.016G085200.3                KKF-IVLR-DQVL---ESAAQMM-----------------DRYAH----LK-V-PIE-VVY------------------NEEVGT-GLGPTLEFYTLVSKEFQ------------K----
    Potri.016G096500.1                VEI-VVRR-SHIV---EDGFQQL-----------------NSLG--SR-LK-S-SIH-VSFVSECG-------------LPEAGLDYGGLSKEFLTDISKSAF------------S----
    Potri.016G012900.1                HEM-LIDR-SQLL---AESFEYI-----------------VHADS-DA-LH-D-GLF-LEF------------------KNEEAT-GPGVLREWFFLVTQALF------------D----
    Potri.002G110500.1                LRI-SVRR-AYIL---EDSYNQL-----------------RMRST-ID-LK-G-RLT-VHF------------------QGEEGIDAGGLTREWYQLLSRVIF------------D----
    Potri.008G101300.1                NRY-RIRR-DHIL---EDAYNQM-----------------SALSE-ED-LR-G-LIR-VSFINEFG-------------VEEAGIDGGGIFKDFMENITRAAF------------D----
    Potri.001G368600.1                LRI-SVRR-AYVL---EDSYNQL-----------------RMRPT-QD-LR-G-RLN-VQF------------------QGEEGIDAGGLTREWYQLLSRVVF------------D----
    Phvul.003G084200.1                HEM-LIDR-SQLL---AESFEYI-----------------ARAEP-ES-LH-A-GLF-MEF------------------KNEEAT-GPGVLREWFLLVCQAIF------------N----
    Phvul.003G118500.1                NRF-RIKR-DHIL---EDAYNQM-----------------SQLTE-DS-LR-G-LMR-VTFVNEFG-------------VEEAGIDGGGIFKDFMENITRAAF------------D----
    Phvul.009G119700.1                QKV-RVSR-NRVL---DSAAKVM-----------------EMYSS----QK-A-VLE-VEY------------------FGEVGT-GLGPTLEFYTILSHDLQ------------K----
    Phvul.009G034900.1                LRI-SVRR-AYVL---EDSYNQL-----------------RMRST-QD-LK-G-RLT-VHF------------------QGEEGIDAGGLTREWYQLLSRVIF------------D----
    Phvul.011G035200.1                QKV-RVSR-NRIL---DSAAKVM-----------------ELYSS----QK-A-VLE-VEY------------------FGEVGT-GLGPTLEFYTLLSHDIQ------------R----
    Phvul.008G183200.1                LRI-SVRR-AYVL---EDSYNQL-----------------RMRST-QD-LK-G-RLT-VHF------------------QGEEGIDAGGLTREWYQLLSRVIF------------D----
    Phvul.007G163300.1                KKF-LVHR-DRIL---ESAARMM-----------------ELHAS----HK-V-VLE-VEY------------------DEEVGT-GLGPTLEFYTLVCHEFQ------------K----
    Phvul.007G163400.1                KKF-LVHR-NRIL---ESAEQMM-----------------ELHAN----NK-V-VLV-VEY------------------YEEVGT-GLGPTLEFYTLVCHEFQ------------K----
    Phvul.001G184300.1                IEI-VVRR-GHIV---EDGFRQL-----------------NSLG--SR-LK-S-SIH-VSFVSECG-------------LLEAGLDYGGLSKEFLTDLSKAAF------------A----
    Phvul.006G120900.1                NRF-KIRR-NHIL---EDAYNQM-----------------SQLSE-DD-LR-G-LIR-VAFVNEFG-------------VEEAGIDGGGIFKDFMENITRAAF------------D----
    Phvul.006G142800.1                HEM-LIDR-SQVL---VESFEYI-----------------RRANP-ES-LR-S-GLF-IEF------------------KNEEAT-GPGVLREWFALVCRRIF------------D----
    Phvul.002G189700.1                LRI-SVRR-AYIL---EDSYNQL-----------------RMRPT-QD-LK-G-RLN-VQF------------------QGEEGIDAGGLTREWYQLLSRVIF------------D----
    mrna26562.1-v1.0-hybrid           LRI-SVRR-AYIL---EDSYNQL-----------------RMRST-QD-LK-G-RLT-VHF------------------QGEEGIDAGGLTREWYQLLSRVIF------------D----
    mrna05017.1-v1.0-hybrid           NRF-RIRR-DRIL---EDAYNQM-----------------SALSE-ED-LR-G-PIR-VTFVNEFG-------------VEEAGIDGGGIFKDFMENITRAAF------------D----
    mrna09579.1-v1.0-hybrid           QKF-LVFR-NRIL---DSAAQMM-----------------DLHAY----QK-V-LLE-VEY------------------DEEVGT-GLGPTLEFYTLVSHEFQ------------K----
    mrna30084.1-v1.0-hybrid           VDI-VVRR-GHIF---EDGFRQL-----------------NSLG--SR-LK-S-SIH-VSFVSECG-------------LPEAGLDYGGLSKEFLTDISKAAF------------A----
    mrna07649.1-v1.0-hybrid           QKV-RVSR-NRIL---ESAAKVM-----------------EMYAS----QK-S-VLE-VEY------------------FGEVGT-GLGPTLEFYTLLSHDLQ------------K----
    mrna20590.1-v1.0-hybrid           LRI-SVRR-AYVL---EDSYNQL-----------------RMRPN-QD-MK-G-RLN-VQF------------------QGEEGIDAGGLTREWYQLLSRVIF------------D----
    mrna19775.1-v1.0-hybrid           HEM-LIDR-SQLL---SESFEYI-----------------GRADP-DS-LH-A-GLF-MEF------------------KNEEAT-GPGVLREWFFLVCQEIF------------N----
    Solyc04g076620.2.1                LRI-SVRR-AYIL---EDSYNQL-----------------RMRTT-QE-LK-G-RLT-VHF------------------QGEEGIDAGGLTREWYQLLSRVIF------------D----
    Solyc10g083470.1.1                YSM-FIDR-SQLL---ESSFEYI-----------------ITATR-KN-LH-G-CLF-IKF------------------KHEEAT-GPGVLREWFLLVCQAMF------------N----
    Solyc10g055450.1.1                QKV-RVSR-NRIL---DSAAKVM-----------------EMYSS----QK-A-VLE-VEY------------------FGEVGT-GLGPTLEFYTLISHDLQ------------K----
    Solyc07g065630.2.1                LRI-SVRR-AYVL---EDSYNQL-----------------RMRPN-QD-LK-G-RLN-VHF------------------QGEEGIDAGGLTREWYQLLSRVIF------------D----
    Solyc05g054080.2.1                HEM-LIDR-AQLL---SESFEYI-----------------AHADP-ES-LR-G-GLF-MEF------------------KSEEAT-GPGVLREWFFLVCRAIF------------N----
    Solyc01g057900.2.1                HRF-RIRR-DHIL---EDAFNQL-----------------NALSE-ED-LR-G-LIR-VTFVNELG-------------VEEAGIDGGGIFKDFMENITRAAF------------D----
    Solyc01g111530.2.1                QKV-RVSR-NRIL---DSAAKVM-----------------EMYSS----QK-A-VLE-VEY------------------FGEVGT-GLGPTLEFYTLLSRDLQ------------K----
    Solyc12g094560.1.1                YGM-IIDR-SMLL---DESFEYI-----------------VDEDP-AL-LR-G-DLL-LQF------------------KHEEAV-GPGVLREWFFLVCREMF------------N----
    Solyc09g005150.1.1                WEM-LISR-SRLF---EDSFEYI-----------------GHASR-RS-LR-G-QLF-IRF------------------ENEEAT-GPGVLREWFSLVCEAIF------------N----
    Solyc09g007310.2.1                KKF-LVHR-SRIL---DSARQMM-----------------DLHAN----QK-V-VIE-VEY------------------NDEVGT-GLGPTLEFFTFVSHEFQ------------K----
    Solyc09g005160.1.1                YEM-LICR-SRLF---EQSFEYI-----------------GRASP-KS-LQ-G-QLF-IQF------------------ENEEAT-GPGVLREWFSLVCEAIF------------N----
    Solyc09g008700.1.1                VEI-VIRR-GHII---EDGFQQL-----------------NNLG--SR-LK-S-GIH-VSFVNESG-------------LPEAGLDYGGLSKEFLTEIAKAAF------------S----
    69212                             INL-VCDR-ASPL---EDLCMHCTPPRSTGG---------AGGFT-AK-PA-G-GVH-IAF------------------KDEAGQ-GAAVRREWMSIVSAAAC------------D----
    70217                             AEI-TIRR-DLLL---EDALAQI-----------------PRLG--DA-IR-G-RLA-VRYVNAAG-------------GDEAGIDAGGLFKELVSDVLAAGF------------D----
    48481                             LEI-SVNR-ERLL---RDATRAV-----------------ASRSP-AD-LK-K-PMR-VRFSSD-G-------------VEEEGVDEGGVTKEFFQLLVRELF------------LPPEK
    19835                             HHI-QVTR-GRVF---ADALDALGPAALKHENRRWRDDHVGERPP-GS-LK-G-VVR-VNFVNEHG-------------VEEAGVDGGGLFKDFLSALIEEAF------------D----
    213597                            VRA-TINR-KQVL---MDSFTQL-----------------QHLKP-AE-MR-G-RLT-IQF------------------SGEEGIDAGGLTREWYILLAREMF------------N----
    174890                            QKV-RVSR-ERIL---ESAVKVF-----------------DMTGA----HK-M-VLE-VEF------------------LNEVGT-GTGPTLEFYTLLSKELT------------Q----
    154462                            ----------------EDGFERL-----------------ERDEDDDDDARSA---------------------------------GGGST------------------------DL---
    22875                             LVV-RVRR-DTLV---QDVLAQI-----------------SSKRE-RD-LR-K-PLK-VAF------------------VGEQGVDEGGVAKEFFQLFVRRVF------------D----
    172918                            ERV-TVQR-NAIL---DDAELLM-----------------RRHAK----HK-S-VLE-VLF------------------ENEEGF-GGAVTKEFYNKVAAALQSRA--------GN----
    29762                             ------------V---QDGFEKL-----------------NLPG--DA-LR-G-RIR-IQYVDSFG-------------EVEAGVDGGGLFKDFMENLIKEGF------------D----
    67182                             QKV-RISR-KRIL---ESAAKVF-----------------EMYGA----SR-A-VLE-IEY------------------FGEVGT-GLGPTLEFYTMLSHDLQ------------R----
    58691                             LDI-RIRR-DRVL---EDALNQV-----------------VGR-P-HE-LK-K-PLR-VTFISQ-G-------------VDEEGLDQGGVKKEFFQLLTREIF------------N----
    16350                             ----QVRRGEHLV---HDTLLQI-----------------QNAG--SA-IR-R-PLK-VQF------------------IGEEGVDEGGVQKEFFQLLMRELF------------D----
    35876                             CIV-RIRR-QHLL---EDALNEV-----------------ARQRP-KD-LF-K-PLR-VHF------------------IGEDGIDAGGVKKEFFQLLVTELL------------C----
    15978                             LEI-TVRR-SAIL---DDGYAAL-----------------RNVG--GS-IK-G-RLS-VSFVNVHG-------------EMEAGLDHGGLVKEFLEEVVKAGF------------D----
    37891                             LRI-HVRR-EHIF---EDSFHQL-----------------RSKTP-EE-MR-G-KLS-VQF------------------HSEEGIDAGGVTREWYQVMARETF------------N----
    57759                             ERV-TVRR-DRVL---ADADVLM-----------------RHHAR----HK-S-VLE-VLF------------------TDEEGF-GGAVTKEFYNKVADALQLRS--------EN----
    59359                             VRA-QINR-KQVL---TDSFMQL-----------------QHLKP-AE-LR-G-RLT-IQF------------------SGEEGIDAGGVSREWYMLLARDMF------------N----
    108435                            HHI-QINR-GRVF---DDALAALGPAVLSGDQATWRREHPDERPP-GS-LK-G-IVR-VQFTNEHG-------------VEEAGVDGGGLFKDFLNDLIAEAF------------D----
    87459                             LKL-DVRR-DCLL---ADTMRQL-----------------AYHVRCGD-IR-K-PLR-VRF------------------VGEEGVDEGGVQKEFFQLIAPEVF------------S----
    60437                             LEL-VIER-GNLL---KNALDAV-----------------ASKTP-AD-LK-K-PLR-IKFKSD-G-------------VEEEGVDEGGVTKEFFQLMVREMF------------KD---
    60965                             VDF--VDSPPPAVSNHPNRYSAA--------F--------PTNST-DA-LR-GVVVN-ASF------------------RGELGN-GPGVVREAFQLAATALLC-----------D----
    83330                             QKV-RVSR-QRIL---ESAMKVY-----------------EMPGA----HK-M-VLE-VEF------------------FNEVGT-GTGPTLEFYTLMSKEVT------------Q----
    62795                             ADV-YVRR-GSVL---EDATAQI-----------------LPLG--PR-AR-G-RLA-VRYRNAAG-------------MEEAGIDAGGLFKELLADVCGAGL------------D----
    91960                             -------------------------------------------------PR-G-GVL-VKF------------------KGERGA-GAAVRREWMSLVAAAAG------------D----
    52147                             RTL-HIRR-DRIL---EDSFRQL-----------------NSRSI-EE-IR-G-KIS-IVF------------------VGEEGMDGGGLIKEWFTILAREVF------------N----
    36723                             ATI-RVRR-GHLL---EDGIAGL-----------------SDKLT-ET-LG-G-IIR-VQFINQQG-------------LEEAGVDGGGLFKDFLNDLIAEAF------------D----
    31158                             QNL-ALSK-SFTLS-AQDAWHDF---------FV----NVWSVSP-KI-LK-------IRHLSW----------GLRQPSGDVSV-GPGPTREAFSLMANELC------------D----
    39499                             QKV-RVNR-GQIF---ESAKKVF-----------------DIPNT----LK-M-VLE-VEF------------------FEEVGT-GTGPTLEFFTLLSKQFK------------R----
    41776                             ATI-RVRR-GHLL---EDGIAGL-----------------SDKLT-ET-LG-G-IIR-VQFINQQG-------------LEEAGVDGGGLFKDFLNDLIAEAF------------D----
    41898                             RTL-HIRR-DRIL---EDSFRQL-----------------NSRSI-EE-IR-G-KIS-IVF------------------VGEEGMDGGGLIKEWFTILAREVF------------N----
    43113                             AQV-TLRR-ESVL---EDTLTSV-----------------LPLG--AK-AR-G-RIL-VKFVNAAG-------------QEEAGIDAGGLFKELLSQVTEQGL------------D----
    Thecc1EG022084t1                  KKF-LVWR-DRIL---DSATRMM-----------------DLHAR----HK-G-LLE-VEY------------------NEEVGT-GLGPTLEFYTLVCHEFQ------------K----
    Thecc1EG022374t1                  VEI-VIRR-GHIV---EDGFRQL-----------------NSLG--SR-LK-S-SIH-VSFVSECG-------------LPEAGLDYGGLSKEFLTDISKEAF------------A----
    Thecc1EG030368t1                  HEM-LIDR-SQLL---AESFEYI-----------------ARAEP-ES-LH-A-GLF-MEF------------------KNEEAT-GPGVLREWFFLVCQAIF------------N----
    Thecc1EG030623t1                  LRI-SVRR-AYVL---EDSYNQL-----------------RMRPT-PD-LK-G-RLN-VQF------------------QGEEGIDAGGLTREWYQLLSRVIF------------D----
    Thecc1EG021434t2                  NRF-RIRR-DHIL---EDAYNQM-----------------SALSE-ED-LR-G-LIR-VTFVNEFG-------------VEEAGIDGGGIFKDFMENITRAAF------------D----
    Thecc1EG034540t1                  LRI-SVRR-AYIL---EDSYNQL-----------------RMRST-QD-LK-G-RLT-VHF------------------QGEEGIDAGGLTREWYQLLSRVIF------------D----
    Thecc1EG006633t1                  QKV-RVSR-NRIL---DSAAKVM-----------------EMYSS----QK-A-VLE-VEY------------------FGEVGT-GLGPTLEFYTLLSHDLQ------------K----
    Cre08.g364550.t1.3                LEV-SVRR-AHIL---HDAAQQL-----------------AGRPL-HQ-LK-R-PLR-VSFVSQ-G-------------MAEEGVDQGGVSREFFQLLVAEIF------------Q----
    Cre07.g312900.t1.3                QKV-RVSR-KRIL---ESAAKVM-----------------ELYAR----SR-A-VLE-LEY------------------FNEVGT-GLGPTLEFYTLLSHELQ------------R----
    g11539.t1                         LVL-RVRRGPYLV---QDTLIQI-----------------HRAKE-SDSLK-K-PLK-VKF------------------IGEEGVDEGGVAKEFFQLLVRQLF------------N----
    Cre06.g280300.t1.3                NRFVPIRR-DQLL---FDGFDRL-----------------NSLG--ER-LR-G-RVR-IMFIDAHG-------------QPEAGVDGGGLFKDFMEELMRAGL------------S----
    Cre02.g099100.t1.3                CIV-RVRR-SHLV---EDALEEV-----------------GRQTR-SD-LL-K-PLR-VHF------------------IGEEGIDAGGVKKEFFALLMERLL------------D----
    Cre03.g159200.t1.2                IKV-TIRR-ASLI---EDAYAGL-----------------AQAG--SG-LK-A-RLQ-VTFINEAG-------------MTEAGLDFGGLQKELLERVVSAGL------------D----
    Cre01.g022100.t1.2                PVL-QVSR-SDLL---GTSVSEL-----------------MQYDG-YS-LG-AEGIC-ISF------------------DDEQAY-GDGVLREWLTEVAGVVF------------D----
    Cre01.g012450.t1.3                LRL-AVRR-EHVF---EDSFYQL-----------------RGRPA-EE-MR-L-KLN-VTF------------------QGEEGIDAGGVTREWYQVMAREMF------------N----
    Cre10.g433900.t1.3                HKL-EIRR-NRCF---KDSVAIF-----------------AGKGH-AV-WR-Q-PLK-VTF------------------IGEAGMDSGGVTREWFSTLSSAIS------------R----
    Cre12.g533750.t1.3                PQV-LAYR-NSIT---ESSYYQV-------------------MDA-DA-LP-F-GVN-VRF------------------EDEQEAEGMGVVREWLSQIAADLF------------S----
    Cre12.g548100.t1.3                TNV-TVRR-CAAF---WDAFEAARHDGLLS----------ASHGP-KS-HKYF-----PSFVDEMAGAGAG-AASVRKAPVEAGE-GHGPRKEFFALAGQDMAGRASSAAQGAGDD-DKA

    Selected Cols:                                                                                                                                            

    Gaps Scores:                                                                                                                                              

                                             130       140       150       160       170       180       190       200       210       220       230       240
                                      =========+=========+=========+=========+=========+=========+=========+=========+=========+=========+=========+=========+
    Sb01g011845.1                     --------------------------P-EYG-----------LFSQ--------------------------------------------------------------------------
    Sb02g016200.1                     --------------------------K-GAL-----------LFT---------------------------------------------------------------------------
    Sb04g000340.1                     --------------------------A-QLG-----------LWRS--------------------------------------------------------------------------
    Sb06g003290.1                     --------------------------V-DLG-----------LWRS--------------------------------------------------------------------------
    Sb08g012560.1                     --------------------------K-GAL-----------LFT---------------------------------------------------------------------------
    Sb09g002120.1                     --------------------------G-GLG-----------MWRG--------------------------------------------------------------------------
    Sb09g004530.1                     --------------------------V-QYG-----------LFK---------------------------------------------------------------------------
    Sb09g022820.1                     --------------------------S-GLG-----------MWRG--------------------------------------------------------------------------
    73381                             --------------------------E-QAL-----------LFS---------------------------------------------------------------------------
    50844                             --------------------------P-QFA-----------LFL---------------------------------------------------------------------------
    89794                             --------------------------P-GYG-----------LFQQ--------------------------------------------------------------------------
    3542                              --------------------------K---------------LFT---------------------------------------------------------------------------
    76253                             --------------------------P-QYG-----------MFS---------------------------------------------------------------------------
    443962                            --------------------------I-QYG-----------LFK---------------------------------------------------------------------------
    181768                            --------------------------V-GYG-----------MFT---------------------------------------------------------------------------
    407700                            --------------------------S-TRG-----------LFVL--------------------------------------------------------------------------
    146155                            --------------------------N-SLD-----------LWRT--------------------------------------------------------------------------
    154179                            --------------------------K-GAL-----------LFT---------------------------------------------------------------------------
    943823                            --------------------------V-SLG-----------MWRS--------------------------------------------------------------------------
    487067                            --------------------------P-DLG-----------MWRN--------------------------------------------------------------------------
    485684                            ----------------------------EYG-----------LFSQ--------------------------------------------------------------------------
    490058                            --------------------------P-KNT-----------LFL---------------------------------------------------------------------------
    479191                            --------------------------V-QYG-----------LFK---------------------------------------------------------------------------
    916552                            --------------------------K-GAL-----------LFT---------------------------------------------------------------------------
    940321                            --------------------------P-PPP-----------FYV---------------------------------------------------------------------------
    474651                            --------------------------K-GAL-----------LFT---------------------------------------------------------------------------
    915021                            --------------------------P-ENK-----------LFI---------------------------------------------------------------------------
    evm.model.supercontig_146.73      --------------------------V-QYG-----------LF----------------------------------------------------------------------------
    evm.model.supercontig_21.42       --------------------------K-GAL-----------LFT---------------------------------------------------------------------------
    evm.model.supercontig_37.145      --------------------------V-GLG-----------MWRS--------------------------------------------------------------------------
    evm.model.supercontig_5.113       --------------------------P-EYG-----------LFSQ--------------------------------------------------------------------------
    evm.model.supercontig_959.1       --------------------------K-GAL-----------LFT---------------------------------------------------------------------------
    29206.m000140                     --------------------------Q-QNA-----------LFV---------------------------------------------------------------------------
    29596.m000712                     --------------------------K-GAL-----------LFT---------------------------------------------------------------------------
    29602.m000214                     --------------------------V-VLG-----------MWRS--------------------------------------------------------------------------
    29629.m001405                     --------------------------K-GAL-----------LFT---------------------------------------------------------------------------
    29805.m001489                     --------------------------S-GLG-----------IWRD--------------------------------------------------------------------------
    29815.m000491                     --------------------------P-EYG-----------LFSQ--------------------------------------------------------------------------
    29889.m003352                     --------------------------V-QYG-----------LFK---------------------------------------------------------------------------
    Cucsa.042120.1                    --------------------------K-GAL-----------LFT---------------------------------------------------------------------------
    Cucsa.044750.1                    --------------------------P-QNA-----------LFV---------------------------------------------------------------------------
    Cucsa.160480.1                    --------------------------K-GAL-----------LFT---------------------------------------------------------------------------
    Cucsa.234290.1                    --------------------------A-GLG-----------MWRS--------------------------------------------------------------------------
    Cucsa.307200.1                    --------------------------N-GLG-----------MWRG--------------------------------------------------------------------------
    Cucsa.378730.1                    --------------------------P-EYG-----------LFSQ--------------------------------------------------------------------------
    ppa000451m                        --------------------------P-EYG-----------LFSQ--------------------------------------------------------------------------
    ppa000008m                        --------------------------K-GAL-----------LFT---------------------------------------------------------------------------
    ppa001143m                        --------------------------P-QNA-----------LFV---------------------------------------------------------------------------
    ppa000674m                        --------------------------V-QYG-----------LFK---------------------------------------------------------------------------
    ppa000169m                        --------------------------S-GLG-----------MWRE--------------------------------------------------------------------------
    ppa000009m                        --------------------------K-GAL-----------LFT---------------------------------------------------------------------------
    ppa000080m                        --------------------------V-RLG-----------MWRS--------------------------------------------------------------------------
    mgv1a001314m                      --------------------------P-QNA-----------LFV---------------------------------------------------------------------------
    mgv1a000078m                      --------------------------V-GLG-----------TWRS--------------------------------------------------------------------------
    mgv1a000005m                      --------------------------K-GAL-----------LFT---------------------------------------------------------------------------
    mgv11b024345m                     --------------------------P-SLN-----------LFK---------------------------------------------------------------------------
    mgv1a000436m                      --------------------------P-EYG-----------LFSQ--------------------------------------------------------------------------
    mgv1a000163m                      --------------------------S-GLG-----------MWRD--------------------------------------------------------------------------
    GSVIVT01003328001                 --------------------------P-QNA-----------LFV---------------------------------------------------------------------------
    GSVIVT01009206001                 --------------------------K-GAL-----------LFT---------------------------------------------------------------------------
    GSVIVT01014698001                 --------------------------K-GAL-----------LFT---------------------------------------------------------------------------
    GSVIVT01018731001                 --------------------------A-ELG-----------MWRS--------------------------------------------------------------------------
    GSVIVT01024033001                 --------------------------V-GLG-----------MWSP--------------------------------------------------------------------------
    GSVIVT01025537001                 --------------------------T-GLG-----------MWRE--------------------------------------------------------------------------
    GSVIVT01033734001                 --------------------------P-EYG-----------LFSQ--------------------------------------------------------------------------
    GSVIVT01034942001                 --------------------------V-QYG-----------LFK---------------------------------------------------------------------------
    cassava4.1_000003m                --------------------------K-GAL-----------LFT---------------------------------------------------------------------------
    cassava4.1_000080m                --------------------------V-ALG-----------MWRS--------------------------------------------------------------------------
    cassava4.1_002295m                --------------------------V-QYG-----------LFK---------------------------------------------------------------------------
    cassava4.1_000006m                --------------------------K-GAL-----------LFT---------------------------------------------------------------------------
    cassava4.1_000011m                --------------------------K-GAL-----------LFT---------------------------------------------------------------------------
    cassava4.1_000177m                --------------------------S-GLG-----------MWRE--------------------------------------------------------------------------
    Pp1s205_47V6.1                    --------------------------P-QNA-----------LFL---------------------------------------------------------------------------
    Pp1s148_98V6.1                    --------------------------P-QNA-----------LFL---------------------------------------------------------------------------
    Pp1s103_43V6.1                    --------------------------P-GYG-----------LFVQ--------------------------------------------------------------------------
    Pp1s42_128V6.2                    --------------------------K-GAL-----------LFT---------------------------------------------------------------------------
    Pp1s263_1V6.1                     --------------------------E-KLN-----------LWRS--------------------------------------------------------------------------
    Pp1s263_20V6.1                    --------------------------P-EYG-----------LFVQ--------------------------------------------------------------------------
    Pp1s15_454V6.1                    --------------------------P-GYG-----------LFFQ--------------------------------------------------------------------------
    Pp1s67_251V6.1                    --------------------------P-QYA-----------LFTH--------------------------------------------------------------------------
    Pp1s173_137V6.1                   --------------------------V-QYG-----------LFK---------------------------------------------------------------------------
    Pp1s116_90V6.1                    --------------------------E-KLD-----------MWRS--------------------------------------------------------------------------
    Pp1s138_130V6.1                   --------------------------K-GAL-----------LFT---------------------------------------------------------------------------
    Pp1s229_59V6.1                    --------------------------V-GYG-----------MFT---------------------------------------------------------------------------
    Pp1s88_123V6.1                    --------------------------P-GYG-----------LFVQ--------------------------------------------------------------------------
    orange1.1g000286m                 --------------------------V-GLA-----------MWRS--------------------------------------------------------------------------
    orange1.1g045956m                 --------------------------P-EYG-----------LFSQ--------------------------------------------------------------------------
    orange1.1g000014m                 --------------------------K-GAL-----------LFT---------------------------------------------------------------------------
    orange1.1g001688m                 --------------------------V-QYG-----------LFK---------------------------------------------------------------------------
    orange1.1g000012m                 --------------------------K-GAL-----------LFT---------------------------------------------------------------------------
    AT4G12570.1                       --------------------------P-KNT-----------LFL---------------------------------------------------------------------------
    AT4G38600.1                       --------------------------A-SLG-----------MWRS--------------------------------------------------------------------------
    AT1G55860.1                       --------------------------K-GAL-----------LFT---------------------------------------------------------------------------
    AT1G70320.1                       --------------------------K-GAL-----------LFT---------------------------------------------------------------------------
    AT3G53090.1                       --------------------------T-EYG-----------LFSQ--------------------------------------------------------------------------
    AT3G17205.1                       --------------------------V-QYG-----------LFK---------------------------------------------------------------------------
    AT5G02880.1                       --------------------------P-DLG-----------MWRN--------------------------------------------------------------------------
    Si034011m                         --------------------------P-EYG-----------LFSQ--------------------------------------------------------------------------
    Si016079m                         --------------------------S-QLG-----------LWRS--------------------------------------------------------------------------
    Si013562m                         --------------------------P-CLV-----------LFS---------------------------------------------------------------------------
    Si013264m                         --------------------------P-RVV-----------LFS---------------------------------------------------------------------------
    Si009242m                         --------------------------V-QYG-----------LFK---------------------------------------------------------------------------
    Si009164m                         --------------------------V-DLG-----------LWRS--------------------------------------------------------------------------
    Si024055m                         --------------------------A-GLG-----------MWRG--------------------------------------------------------------------------
    Si020966m                         --------------------------S-GLG-----------MWRG--------------------------------------------------------------------------
    Si020939m                         --------------------------K-GAL-----------LFT---------------------------------------------------------------------------
    Si028891m                         --------------------------P-KQV-----------LFS---------------------------------------------------------------------------
    Si028637m                         --------------------------K-GAL-----------LFT---------------------------------------------------------------------------
    Thhalv10019984m                   --------------------------V-QYG-----------LFK---------------------------------------------------------------------------
    Thhalv10011172m                   --------------------------K-GAL-----------LFT---------------------------------------------------------------------------
    Thhalv10011171m                   --------------------------K-GAL-----------LFT---------------------------------------------------------------------------
    Thhalv10024192m                   --------------------------V-ALG-----------MWRS--------------------------------------------------------------------------
    Thhalv10028412m                   --------------------------P-QNT-----------LFR---------------------------------------------------------------------------
    Thhalv10012430m                   --------------------------P-DLG-----------MWRS--------------------------------------------------------------------------
    Thhalv10010078m                   --------------------------T-EYG-----------LFSQ--------------------------------------------------------------------------
    Ciclev10000001m                   --------------------------K-GAL-----------LFT---------------------------------------------------------------------------
    Ciclev10004231m                   --------------------------V-QYG-----------LFK---------------------------------------------------------------------------
    Ciclev10007219m                   --------------------------K-GAL-----------LFT---------------------------------------------------------------------------
    Ciclev10010897m                   --------------------------S-GMG-----------MWRD--------------------------------------------------------------------------
    Ciclev10010940m                   --------------------------P-EYG-----------LFSQ--------------------------------------------------------------------------
    Ciclev10027670m                   --------------------------V-GLA-----------MWRS--------------------------------------------------------------------------
    Ciclev10014213m                   --------------------------P-QNA-----------LFV---------------------------------------------------------------------------
    GRMZM2G034622_T02                 --------------------------V-QYG-----------LFK---------------------------------------------------------------------------
    GRMZM2G124297_T01                 --------------------------A-GLG-----------MWRG--------------------------------------------------------------------------
    GRMZM2G411536_T03                 --------------------------K-SAL-----------LFT---------------------------------------------------------------------------
    GRMZM2G181378_T01                 --------------------------P-KQV-----------LFS---------------------------------------------------------------------------
    GRMZM2G049141_T01                 --------------------------V-DLG-----------LWRS--------------------------------------------------------------------------
    GRMZM2G080439_T01                 --------------------------S-KQV-----------LFS---------------------------------------------------------------------------
    GRMZM2G021299_T01                 --------------------------K-GTL-----------LFT---------------------------------------------------------------------------
    GRMZM2G328988_T01                 --------------------------S-GLG-----------MWRG--------------------------------------------------------------------------
    GRMZM2G331368_T02                 --------------------------K-SAL-----------LFT---------------------------------------------------------------------------
    GRMZM2G461948_T01                 --------------------------P-EYG-----------LFSQ--------------------------------------------------------------------------
    GRMZM2G374574_T01                 --------------------------A-QLG-----------LWRA--------------------------------------------------------------------------
    Carubv10016604m                   --------------------------T-EYG-----------LFSQ--------------------------------------------------------------------------
    Carubv10011657m                   --------------------------K-GAL-----------LFT---------------------------------------------------------------------------
    Carubv10007210m                   --------------------------P-GRK-----------LFV---------------------------------------------------------------------------
    Carubv10003974m                   --------------------------V-SLG-----------MWRS--------------------------------------------------------------------------
    Carubv10012881m                   --------------------------V-QYG-----------LFK---------------------------------------------------------------------------
    Carubv10000054m                   --------------------------P-DLG-----------MWRS--------------------------------------------------------------------------
    Carubv10000186m                   --------------------------P-KNT-----------LFL---------------------------------------------------------------------------
    Carubv10025730m                   --------------------------K-GAL-----------LFT---------------------------------------------------------------------------
    Bradi2g34820.1                    --------------------------V-QYG-----------LFK---------------------------------------------------------------------------
    Bradi2g37870.1                    --------------------------V-GTG-----------MWRG--------------------------------------------------------------------------
    Bradi2g22927.2                    --------------------------S-GLG-----------MWRG--------------------------------------------------------------------------
    Bradi4g07997.2                    --------------------------K-GAL-----------LFT---------------------------------------------------------------------------
    Bradi4g33520.1                    --------------------------P-QQV-----------LFS---------------------------------------------------------------------------
    Bradi1g12340.2                    --------------------------P-EYG-----------LFSQ--------------------------------------------------------------------------
    Bradi5g04567.1                    --------------------------V-GLG-----------LWRS--------------------------------------------------------------------------
    Bradi3g00350.1                    --------------------------A-RLG-----------LWRS--------------------------------------------------------------------------
    Aquca_017_00766.1                 --------------------------V-GMG-----------MWRG--------------------------------------------------------------------------
    Aquca_006_00259.1                 --------------------------I-TLG-----------MWRS--------------------------------------------------------------------------
    Aquca_028_00189.1                 --------------------------P-QNP-----------LFL---------------------------------------------------------------------------
    Aquca_027_00123.1                 --------------------------V-GLG-----------MWRG--------------------------------------------------------------------------
    Aquca_007_00539.1                 --------------------------K-GAL-----------LFT---------------------------------------------------------------------------
    Aquca_003_00437.1                 --------------------------M-QYG-----------LFK---------------------------------------------------------------------------
    Aquca_019_00105.1                 --------------------------P-EYG-----------LFTQ--------------------------------------------------------------------------
    MDP0000264736                     --------------------------P-QNA-----------LFV---------------------------------------------------------------------------
    MDP0000320720                     --------------------------S-GLG-----------MWRE--------------------------------------------------------------------------
    MDP0000142676                     --------------------------P-QNA-----------LFV---------------------------------------------------------------------------
    MDP0000318443                     --------------------------K-GAL-----------LFT---------------------------------------------------------------------------
    MDP0000206447                     --------------------------K-GAL-----------LFT---------------------------------------------------------------------------
    MDP0000196216                     --------------------------P-DYG-----------LFSQ--------------------------------------------------------------------------
    MDP0000186793                     --------------------------V-QYG-----------LFK---------------------------------------------------------------------------
    MDP0000822588                     --------------------------S-GLG-----------MWRD--------------------------------------------------------------------------
    MDP0000924418                     --------------------------P-DYG-----------LFSQ--------------------------------------------------------------------------
    MDP0000320505                     --------------------------V-RLG-----------MWRS--------------------------------------------------------------------------
    MDP0000307848                     --------------------------K-GAL-----------LFT---------------------------------------------------------------------------
    MDP0000301275                     --------------------------V-RLG-----------MWRS--------------------------------------------------------------------------
    MDP0000317971                     --------------------------K-GAL-----------LFT---------------------------------------------------------------------------
    Bra022201                         --------------------------V-QYG-----------LFK---------------------------------------------------------------------------
    Bra028860                         --------------------------P-DLG-----------MWRS--------------------------------------------------------------------------
    Bra038022                         --------------------------K-GAL-----------LFT---------------------------------------------------------------------------
    Bra021231                         --------------------------V-QYG-----------LFK---------------------------------------------------------------------------
    Bra005748                         --------------------------P-DLG-----------MWRC--------------------------------------------------------------------------
    Bra000779                         --------------------------P-GGT-----------LFL---------------------------------------------------------------------------
    Bra029461                         --------------------------P-RNA-----------LFL---------------------------------------------------------------------------
    Bra027850                         --------------------------K-GAL-----------LFT---------------------------------------------------------------------------
    Bra040685                         --------------------------S-EYG-----------LFSQ--------------------------------------------------------------------------
    Bra010737                         --------------------------V-SLG-----------MWRS--------------------------------------------------------------------------
    Medtr2g025830.1                   --------------------------P-EHA-----------LFV---------------------------------------------------------------------------
    Medtr2g025950.1                   --------------------------P-RNA-----------LFV---------------------------------------------------------------------------
    Medtr2g025810.1                   --------------------------P-RNA-----------LFV---------------------------------------------------------------------------
    Medtr2g033040.1                   --------------------------V-QYG-----------LFKLAPRCMLFADDVVLVGESREEVNGRLETWRQALEAYGFRLSRSKTEYMECNFSGRRSRSTLEVKVGDHIIGGRPR
    Medtr2g025790.1                   --------------------------P-KNA-----------LFV---------------------------------------------------------------------------
    Medtr2g025930.1                   --------------------------P-EHA-----------LFV---------------------------------------------------------------------------
    Medtr7g100670.1                   --------------------------P-GLG-----------LWRE--------------------------------------------------------------------------
    Medtr5g066710.1                   --------------------------K-GAL-----------LFT---------------------------------------------------------------------------
    Medtr4g073370.1                   --------------------------V-GLQ-----------MWRS--------------------------------------------------------------------------
    Medtr4g133120.1                   --------------------------Q-EHA-----------LFV---------------------------------------------------------------------------
    Vocar20002255m                    --------------------------P-QYG-----------MFT---------------------------------------------------------------------------
    Vocar20010178m                    --------------------------P-NLA-----------LFV---------------------------------------------------------------------------
    Vocar20006334m                    ------LQQQLPD-----------CPPGPVG-ADSNVPRRPALWV---------------------------------------------------------------------------
    Vocar20007555m                    --------------------------G-SPE-----------LFY---------------------------------------------------------------------------
    Vocar20012583m                    --------------------------K-DLA-----------MWRH--------------------------------------------------------------------------
    Vocar20003001m                    --------------------------A-EYG-----------LFA---------------------------------------------------------------------------
    Vocar20004069m                    --------------------------P-DYG-----------MML---------------------------------------------------------------------------
    Vocar20000780m                    --------------------------A-NYG-----------LFT---------------------------------------------------------------------------
    Vocar20004842m                    --------------------------P-DYG-----------MFT---------------------------------------------------------------------------
    Vocar20014908m                    --------------------------P-ERG-----------LFVR--------------------------------------------------------------------------
    Lus10032589                       --------------------------K-GAL-----------LFT---------------------------------------------------------------------------
    Lus10035589                       --------------------------P-EYG-----------LFSQ--------------------------------------------------------------------------
    Lus10005068                       --------------------------A-SLG-----------MWRS--------------------------------------------------------------------------
    Lus10010493                       --------------------------P-HNA-----------LFL---------------------------------------------------------------------------
    Lus10027841                       --------------------------A-SLG-----------MWRS--------------------------------------------------------------------------
    Lus10019908                       --------------------------S-DLL-----------MWRG--------------------------------------------------------------------------
    Lus10032830                       --------------------------K-GAL-----------LFT---------------------------------------------------------------------------
    Lus10017098                       --------------------------V-QYG-----------LF----------------------------------------------------------------------------
    Lus10002605                       --------------------------K-GAL-----------LFT---------------------------------------------------------------------------
    Lus10008636                       --------------------------P-EYG-----------LFSQ--------------------------------------------------------------------------
    Eucgr.A01178.1                    --------------------------P-GLG-----------MWRE--------------------------------------------------------------------------
    Eucgr.A01586.1                    --------------------------P-EYG-----------LFTQ--------------------------------------------------------------------------
    Eucgr.B03986.1                    --------------------------V-QYG-----------LFK---------------------------------------------------------------------------
    Eucgr.D01414.1                    --------------------------Q-QNP-----------LFV---------------------------------------------------------------------------
    Eucgr.D01416.1                    --------------------------Q-QNP-----------LFV---------------------------------------------------------------------------
    Eucgr.F02160.1                    --------------------------K-GAL-----------LFT---------------------------------------------------------------------------
    Eucgr.I01410.2                    --------------------------V-GLE-----------MWRS--------------------------------------------------------------------------
    Pavirv00038038m                   --------------------------K-GAL-----------LFT---------------------------------------------------------------------------
    Pavirv00031244m                   --------------------------A-KLG-----------LWRS--------------------------------------------------------------------------
    Pavirv00010575m                   --------------------------P-KQV-----------LFS---------------------------------------------------------------------------
    Pavirv00004902m                   --------------------------P-RLV-----------LFS---------------------------------------------------------------------------
    Pavirv00020428m                   --------------------------A-GLG-----------MWRG--------------------------------------------------------------------------
    Pavirv00067430m                   --------------------------P-KQV-----------LFS---------------------------------------------------------------------------
    Pavirv00058663m                   --------------------------K-GAL-----------LFT---------------------------------------------------------------------------
    Pavirv00067620m                   --------------------------A-QLG-----------LWRS--------------------------------------------------------------------------
    Pavirv00029557m                   --------------------------K-GAL-----------LFT---------------------------------------------------------------------------
    Pavirv00023469m                   --------------------------V-QYG-----------LFK---------------------------------------------------------------------------
    Pavirv00024250m                   --------------------------S-GLG-----------MWRG--------------------------------------------------------------------------
    Pavirv00023205m                   --------------------------S-GLG-----------MWRG--------------------------------------------------------------------------
    Pavirv00029138m                   --------------------------A-GLG-----------MWRG--------------------------------------------------------------------------
    LOC_Os03g47949.1                  --------------------------P-EYG-----------LFSQ--------------------------------------------------------------------------
    LOC_Os02g01170.1                  --------------------------V-RLG-----------LWRS--------------------------------------------------------------------------
    LOC_Os09g07900.1                  --------------------------K-GAL-----------LFT---------------------------------------------------------------------------
    LOC_Os12g24080.1                  --------------------------K-GAL-----------LFT---------------------------------------------------------------------------
    LOC_Os05g38830.1                  --------------------------S-GLG-----------MWRG--------------------------------------------------------------------------
    LOC_Os05g03100.1                  --------------------------V-GLG-----------MWRG--------------------------------------------------------------------------
    LOC_Os05g06690.1                  --------------------------V-QYG-----------LFK---------------------------------------------------------------------------
    PGSC0003DMT400075387              --------------------------P-HKA-----------LFV---------------------------------------------------------------------------
    PGSC0003DMT400021802              --------------------------P-QNA-----------LFV---------------------------------------------------------------------------
    PGSC0003DMT400031190              --------------------------L-GLG-----------MWRS--------------------------------------------------------------------------
    PGSC0003DMT400072624              --------------------------P-QNA-----------LFV---------------------------------------------------------------------------
    Glyma14g36180.1                   --------------------------K-GAL-----------LFT---------------------------------------------------------------------------
    Glyma02g38020.2                   --------------------------K-GAL-----------LFT---------------------------------------------------------------------------
    Glyma12g03640.1                   --------------------------V-VLQ-----------MWRS--------------------------------------------------------------------------
    Glyma11g11490.1                   --------------------------I-ILE-----------MWRS--------------------------------------------------------------------------
    Glyma06g00600.1                   --------------------------V-GLQ-----------MWRS--------------------------------------------------------------------------
    Glyma06g10360.1                   --------------------------K-GAL-----------LFT---------------------------------------------------------------------------
    Glyma04g00530.1                   --------------------------V-GLQ-----------MWRS--------------------------------------------------------------------------
    Glyma04g10481.1                   --------------------------K-GAL-----------LFT---------------------------------------------------------------------------
    Glyma08g09270.3                   --------------------------K-GAL-----------LFT---------------------------------------------------------------------------
    Glyma17g01210.2                   --------------------------P-QNA-----------LFV---------------------------------------------------------------------------
    Glyma17g04180.1                   --------------------------V-QYG-----------LFK---------------------------------------------------------------------------
    Glyma13g19981.1                   --------------------------S-GLG-----------MWRE--------------------------------------------------------------------------
    Glyma05g26360.1                   --------------------------K-GAL-----------LFT---------------------------------------------------------------------------
    Glyma19g37310.1                   --------------------------P-EYG-----------LFSQ--------------------------------------------------------------------------
    Glyma15g14591.1                   --------------------------V-QYG-----------LFK---------------------------------------------------------------------------
    Glyma03g34650.2                   --------------------------P-EYG-----------LFSQ--------------------------------------------------------------------------
    Glyma10g05620.3                   --------------------------S-GLA-----------MWRE--------------------------------------------------------------------------
    Glyma07g36390.1                   --------------------------V-QYG-----------LFK---------------------------------------------------------------------------
    Glyma07g39546.1                   --------------------------P-QNA-----------LFV---------------------------------------------------------------------------
    Gorai.010G033100.1                --------------------------K-GAL-----------LFT---------------------------------------------------------------------------
    Gorai.010G186800.1                --------------------------P-ENA-----------LFV---------------------------------------------------------------------------
    Gorai.009G278900.1                --------------------------K-GAL-----------LFT---------------------------------------------------------------------------
    Gorai.009G228200.1                --------------------------K-GAL-----------LFT---------------------------------------------------------------------------
    Gorai.009G183200.1                --------------------------P-ENA-----------LFL---------------------------------------------------------------------------
    Gorai.009G420400.1                --------------------------F-GLG-----------MWRG--------------------------------------------------------------------------
    Gorai.002G100900.1                --------------------------K-GAL-----------LFT---------------------------------------------------------------------------
    Gorai.002G196900.1                --------------------------V-GLG-----------MWRS--------------------------------------------------------------------------
    Gorai.002G245000.1                --------------------------P-EYG-----------LFSQ--------------------------------------------------------------------------
    Gorai.002G003200.1                --------------------------F-ELG-----------MWRS--------------------------------------------------------------------------
    Gorai.011G204200.1                --------------------------V-QYG-----------LFK---------------------------------------------------------------------------
    Gorai.008G035900.1                --------------------------V-GLA-----------MWRS--------------------------------------------------------------------------
    Gorai.006G265700.1                --------------------------V-QYG-----------LFK---------------------------------------------------------------------------
    Potri.010G150000.3                --------------------------V-QYG-----------LFK---------------------------------------------------------------------------
    Potri.009G134300.1                --------------------------V-TLG-----------MWRS--------------------------------------------------------------------------
    Potri.004G174700.1                --------------------------V-SLG-----------MWRS--------------------------------------------------------------------------
    Potri.011G094100.1                --------------------------K-GAL-----------LFT---------------------------------------------------------------------------
    Potri.006G132000.1                --------------------------S-GLG-----------MWRQ--------------------------------------------------------------------------
    Potri.006G011700.1                --------------------------P-QNA-----------LFV---------------------------------------------------------------------------
    Potri.016G085200.3                --------------------------S-GIG-----------MWRE--------------------------------------------------------------------------
    Potri.016G096500.1                --------------------------P-EHG-----------LFSQ--------------------------------------------------------------------------
    Potri.016G012900.1                --------------------------P-QIA-----------LFV---------------------------------------------------------------------------
    Potri.002G110500.1                --------------------------K-GAL-----------LFT---------------------------------------------------------------------------
    Potri.008G101300.1                --------------------------V-QYG-----------LFK---------------------------------------------------------------------------
    Potri.001G368600.1                --------------------------K-GAL-----------LFT---------------------------------------------------------------------------
    Phvul.003G084200.1                --------------------------P-QNA-----------LFV---------------------------------------------------------------------------
    Phvul.003G118500.1                --------------------------V-QYG-----------LFK---------------------------------------------------------------------------
    Phvul.009G119700.1                --------------------------V-GLQ-----------MWRS--------------------------------------------------------------------------
    Phvul.009G034900.1                --------------------------R-GAL-----------LFT---------------------------------------------------------------------------
    Phvul.011G035200.1                --------------------------V-ALR-----------MWRS--------------------------------------------------------------------------
    Phvul.008G183200.1                --------------------------K-GAL-----------LFT---------------------------------------------------------------------------
    Phvul.007G163300.1                --------------------------S-GLD-----------MWRE--------------------------------------------------------------------------
    Phvul.007G163400.1                --------------------------P-GLG-----------MWRE--------------------------------------------------------------------------
    Phvul.001G184300.1                --------------------------P-EYG-----------LFSQ--------------------------------------------------------------------------
    Phvul.006G120900.1                --------------------------V-QYG-----------LFK---------------------------------------------------------------------------
    Phvul.006G142800.1                --------------------------P-RRV-----------LFV---------------------------------------------------------------------------
    Phvul.002G189700.1                --------------------------K-GAL-----------LFT---------------------------------------------------------------------------
    mrna26562.1-v1.0-hybrid           --------------------------K-GAL-----------LFT---------------------------------------------------------------------------
    mrna05017.1-v1.0-hybrid           --------------------------V-QYG-----------LFK---------------------------------------------------------------------------
    mrna09579.1-v1.0-hybrid           --------------------------S-GLG-----------MWRE--------------------------------------------------------------------------
    mrna30084.1-v1.0-hybrid           --------------------------P-EYG-----------LFSQ--------------------------------------------------------------------------
    mrna07649.1-v1.0-hybrid           --------------------------V-RLG-----------MWRS--------------------------------------------------------------------------
    mrna20590.1-v1.0-hybrid           --------------------------K-GAL-----------LFT---------------------------------------------------------------------------
    mrna19775.1-v1.0-hybrid           --------------------------P-QNA-----------LFV---------------------------------------------------------------------------
    Solyc04g076620.2.1                --------------------------K-GAL-----------LFT---------------------------------------------------------------------------
    Solyc10g083470.1.1                --------------------------P-QNA-----------LFV---------------------------------------------------------------------------
    Solyc10g055450.1.1                --------------------------L-GLG-----------MWRS--------------------------------------------------------------------------
    Solyc07g065630.2.1                --------------------------K-GAL-----------LFT---------------------------------------------------------------------------
    Solyc05g054080.2.1                --------------------------P-QNA-----------LFV---------------------------------------------------------------------------
    Solyc01g057900.2.1                --------------------------V-QYG-----------LFK---------------------------------------------------------------------------
    Solyc01g111530.2.1                --------------------------V-GLR-----------MWRT--------------------------------------------------------------------------
    Solyc12g094560.1.1                --------------------------P-HRA-----------LFV---------------------------------------------------------------------------
    Solyc09g005150.1.1                --------------------------P-QNA-----------LFV---------------------------------------------------------------------------
    Solyc09g007310.2.1                --------------------------I-GLG-----------MWRG--------------------------------------------------------------------------
    Solyc09g005160.1.1                --------------------------P-QNA-----------LFV---------------------------------------------------------------------------
    Solyc09g008700.1.1                --------------------------P-EYG-----------LFTQ--------------------------------------------------------------------------
    69212                             --------------------------R-NCL-----------LFT---------------------------------------------------------------------------
    70217                             --------------------------P-SRG-----------VFA---------------------------------------------------------------------------
    48481                             SSTTGDAEEEEEERSTGTDRPTSSSSS-SSS-----------VFT---------------------------------------------------------------------------
    19835                             --------------------------P-KVG-----------LFV---------------------------------------------------------------------------
    213597                            --------------------------P-GNA-----------LFEL--------------------------------------------------------------------------
    174890                            --------------------------R-ALG-----------AWRD--------------------------------------------------------------------------
    154462                            ----------------------------GIA-----------LLR---------------------------------------------------------------------------
    22875                             --------------------------P-SFG-----------MFA---------------------------------------------------------------------------
    172918                            --------------------------V-ETP-----------TWVM--------------------------------------------------------------------------
    29762                             --------------------------P-RIG-----------LFK---------------------------------------------------------------------------
    67182                             --------------------------K-GLA-----------MWRG--------------------------------------------------------------------------
    58691                             --------------------------E-AYG-----------MFV---------------------------------------------------------------------------
    16350                             --------------------------A-NFG-----------MFR---------------------------------------------------------------------------
    35876                             --------------------------P-DYG-----------MLV---------------------------------------------------------------------------
    15978                             --------------------------V-NRG-----------LFT---------------------------------------------------------------------------
    37891                             --------------------------P-NIS-----------LF----------------------------------------------------------------------------
    57759                             --------------------------T-ATM-----------MWVP--------------------------------------------------------------------------
    59359                             --------------------------P-DKA-----------LFEL--------------------------------------------------------------------------
    108435                            --------------------------PVKTG-----------LFA---------------------------------------------------------------------------
    87459                             --------------------------K-RVG------------FE---------------------------------------------------------------------------
    60437                             ------ARTFVE-------------FS-EHGSEDKNSSLAPPMFT---------------------------------------------------------------------------
    60965                             --------------------------E-DRA-----------LFRPWP--------------------------------------------------------------GG--VGG---
    83330                             --------------------------R-SLK-----------AWRD--------------------------------------------------------------------------
    62795                             --------------------------P-NRG-----------VFAS--------------------------------------------------------------------------
    91960                             --------------------------R-SNL-----------LFA---------------------------------------------------------------------------
    52147                             --------------------------P-NIA-----------LFE---------------------------------------------------------------------------
    36723                             --------------------------P-KFG-----------LFV---------------------------------------------------------------------------
    31158                             --------------------------G-NRA--DRQ------LFT---------------------------------------------------------------------------
    39499                             --------------------------R-KLN-----------LWRD--------------------------------------------------------------------------
    41776                             --------------------------P-KFG-----------LFV---------------------------------------------------------------------------
    41898                             --------------------------P-NIA-----------LFE---------------------------------------------------------------------------
    43113                             --------------------------P-NRG-----------LFQ---------------------------------------------------------------------------
    Thecc1EG022084t1                  --------------------------S-GLG-----------IWRE--------------------------------------------------------------------------
    Thecc1EG022374t1                  --------------------------P-EYG-----------LFSQ--------------------------------------------------------------------------
    Thecc1EG030368t1                  --------------------------P-ENA-----------LFV---------------------------------------------------------------------------
    Thecc1EG030623t1                  --------------------------K-GAL-----------LFT---------------------------------------------------------------------------
    Thecc1EG021434t2                  --------------------------V-QYG-----------LFK---------------------------------------------------------------------------
    Thecc1EG034540t1                  --------------------------K-GAL-----------LFT---------------------------------------------------------------------------
    Thecc1EG006633t1                  --------------------------V-GLG-----------MWRS--------------------------------------------------------------------------
    Cre08.g364550.t1.3                --------------------------P-QYG-----------LFT---------------------------------------------------------------------------
    Cre07.g312900.t1.3                --------------------------K-DLG-----------MWRH--------------------------------------------------------------------------
    g11539.t1                         --------------------------P-DYG-----------MFT---------------------------------------------------------------------------
    Cre06.g280300.t1.3                --------------------------A-EYG-----------LFA---------------------------------------------------------------------------
    Cre02.g099100.t1.3                --------------------------P-GYG-----------LMA---------------------------------------------------------------------------
    Cre03.g159200.t1.2                --------------------------A-NYG-----------LFT---------------------------------------------------------------------------
    Cre01.g022100.t1.2                --------------------------P-NAG-----------LFR---------------------------------------------------------------------------
    Cre01.g012450.t1.3                --------------------------P-NLA-----------LFV---------------------------------------------------------------------------
    Cre10.g433900.t1.3                --------------------------G-SPE-----------LFY---------------------------------------------------------------------------
    Cre12.g533750.t1.3                --------------------------P-ERG-----------LFVR--------------------------------------------------------------------------
    Cre12.g548100.t1.3                ------GDQQQHA-----------AAAGSSA-GGHGPGSRPALWV---------------------------------------------------------------------------

    Selected Cols:                                                                                                                                            

    Gaps Scores:                                                                                                                                              

                                             250       260       270       280       290       300       310       320       330       340       350       360
                                      =========+=========+=========+=========+=========+=========+=========+=========+=========+=========+=========+=========+
    Sb01g011845.1                     ---TSA-----------------------------------------------------------------------------------------------------------------S
    Sb02g016200.1                     ----TV-----------------------------------------------------------------------------------------------------------------G
    Sb04g000340.1                     ---SS----YDSG------------------------------------------------------LHIDRKDVI-------SLDPEDDSS-GKGP----------------NTDLPGD
    Sb06g003290.1                     ---HSP---DDSG------------------------------------------------------MQLDGNAD--------------DLT-SEKR----------------ESESLVE
    Sb08g012560.1                     ----TV-----------------------------------------------------------------------------------------------------------------G
    Sb09g002120.1                     ---DSG------------------------------------------------------------------------------------------------------------------
    Sb09g004530.1                     ----ET-----------------------------------------------------------------------------------------------------------------A
    Sb09g022820.1                     ---ELP---------------------------------------------------------------------------------------CESV----------------TDNGH--
    73381                             ----PV-----------------------------------------------------------------------------------------------------------------G
    50844                             ----PC-----------------------------------------------------------------------------------------------------------------P
    89794                             ---TAT-----------------------------------------------------------------------------------------------------------------E
    3542                              ----TV-----------------------------------------------------------------------------------------------------------------G
    76253                             ----YS-----------------------------------------------------------------------------------------------------------------D
    443962                            ----ET-----------------------------------------------------------------------------------------------------------------A
    181768                            ----YS-----------------------------------------------------------------------------------------------------------------E
    407700                            ------------------------------------------------------------------------------------------------------------------------
    146155                            ---ESR---PGSP------------------------------------------------------QQADTEMPDIN------EDVLDEPS-ENQA----------------SVQPVEQ
    154179                            ----TV-----------------------------------------------------------------------------------------------------------------G
    943823                            ---SSG---DKVS------------------------------------------------------MQIDRDEI---------------ED-GK--------------------SSAA-
    487067                            ---DCS---------------------------------------------------------------------------------------SFVG----------------KPG---E
    485684                            ---TPT-----------------------------------------------------------------------------------------------------------------S
    490058                            ----RS-----------------------------------------------------------------------------------------------------------------A
    479191                            ----ET-----------------------------------------------------------------------------------------------------------------A
    916552                            ----TV-----------------------------------------------------------------------------------------------------------------G
    940321                            ----PY-----------------------------------------------------------------------------------------------------------------T
    474651                            ----TV-----------------------------------------------------------------------------------------------------------------G
    915021                            ----PS-----------------------------------------------------------------------------------------------------------------P
    evm.model.supercontig_146.73      ------------------------------------------------------------------------------------------------------------------------
    evm.model.supercontig_21.42       ----TV-----------------------------------------------------------------------------------------------------------------G
    evm.model.supercontig_37.145      ---NFS--SEKSS------------------------------------------------------MEIDGIED---------------KN-GKNN-------------------SPVV
    evm.model.supercontig_5.113       ---TST-----------------------------------------------------------------------------------------------------------------S
    evm.model.supercontig_959.1       ----TV-----------------------------------------------------------------------------------------------------------------G
    29206.m000140                     ----AC-----------------------------------------------------------------------------------------------------------------P
    29596.m000712                     ----TV-----------------------------------------------------------------------------------------------------------------G
    29602.m000214                     ---NSS--SDKPS------------------------------------------------------MEIDEDGN---------------KN-GKVN----------------N-CSDAM
    29629.m001405                     ----TV-----------------------------------------------------------------------------------------------------------------G
    29805.m001489                     ---DSS---------------------------------------------------------------------------------------LFAD----------------RKDLHTE
    29815.m000491                     ---TST-----------------------------------------------------------------------------------------------------------------S
    29889.m003352                     ----ET-----------------------------------------------------------------------------------------------------------------A
    Cucsa.042120.1                    ----TV-----------------------------------------------------------------------------------------------------------------G
    Cucsa.044750.1                    ----AC-----------------------------------------------------------------------------------------------------------------P
    Cucsa.160480.1                    ----TV-----------------------------------------------------------------------------------------------------------------G
    Cucsa.234290.1                    ---NSL--QESTD------------------------------------------------------SGEDGQAR---------------KP-KGGS----------------RLTSDAA
    Cucsa.307200.1                    ---DHD---------------------------------------------------------------------------------------AFIS----------------GKRLNIE
    Cucsa.378730.1                    ---TST-----------------------------------------------------------------------------------------------------------------P
    ppa000451m                        ---TST-----------------------------------------------------------------------------------------------------------------S
    ppa000008m                        ----TV-----------------------------------------------------------------------------------------------------------------G
    ppa001143m                        ----AC-----------------------------------------------------------------------------------------------------------------P
    ppa000674m                        ----ET-----------------------------------------------------------------------------------------------------------------S
    ppa000169m                        ---DHG---------------------------------------------------------------------------------------SFIS----------------GTT-HAE
    ppa000009m                        ----TV-----------------------------------------------------------------------------------------------------------------G
    ppa000080m                        ---NSS--MEKTS------------------------------------------------------MDIDGDEQ---------------KD-GKSN-----------------------
    mgv1a001314m                      ----AC-----------------------------------------------------------------------------------------------------------------P
    mgv1a000078m                      ---SSS--FGRPS------------------------------------------------------MEIEVDN-----------------------------------------SASAG
    mgv1a000005m                      ----TV-----------------------------------------------------------------------------------------------------------------G
    mgv11b024345m                     ----TT-----------------------------------------------------------------------------------------------------------------S
    mgv1a000436m                      ---TST-----------------------------------------------------------------------------------------------------------------S
    mgv1a000163m                      ---DC-------------------------------------------------------------------------------------------------------------------
    GSVIVT01003328001                 ----AC-----------------------------------------------------------------------------------------------------------------P
    GSVIVT01009206001                 ----TV-----------------------------------------------------------------------------------------------------------------G
    GSVIVT01014698001                 ----TV-----------------------------------------------------------------------------------------------------------------G
    GSVIVT01018731001                 ---RS-------------------------------------------------------------------------------------------------------------------
    GSVIVT01024033001                 ---AAS------------------------------------------------------------------------------------------------------------------
    GSVIVT01025537001                 ---DYT---------------------------------------------------------------------------------------SSTS-----------------------
    GSVIVT01033734001                 ---TST-----------------------------------------------------------------------------------------------------------------S
    GSVIVT01034942001                 ----ET-----------------------------------------------------------------------------------------------------------------A
    cassava4.1_000003m                ----TV-----------------------------------------------------------------------------------------------------------------G
    cassava4.1_000080m                ---NSS--SGKPT------------------------------------------------------MEIDEDGD---------------KN-GKAN----------------NDSRAVG
    cassava4.1_002295m                ----ET-----------------------------------------------------------------------------------------------------------------A
    cassava4.1_000006m                ----TV-----------------------------------------------------------------------------------------------------------------G
    cassava4.1_000011m                ----TV-----------------------------------------------------------------------------------------------------------------G
    cassava4.1_000177m                ---DYS---------------------------------------------------------------------------------------SFVD----------------RKIFQTE
    Pp1s205_47V6.1                    ----PC-----------------------------------------------------------------------------------------------------------------P
    Pp1s148_98V6.1                    ----AC-----------------------------------------------------------------------------------------------------------------P
    Pp1s103_43V6.1                    ---TAT-----------------------------------------------------------------------------------------------------------------E
    Pp1s42_128V6.2                    ----TV-----------------------------------------------------------------------------------------------------------------G
    Pp1s263_1V6.1                     ---EMR---LKSE------------------------------------------------------ATVGVE-----------------------------------------------
    Pp1s263_20V6.1                    ---TAT------------------------------------------------------------------------------------------------------------------
    Pp1s15_454V6.1                    ---TAT-----------------------------------------------------------------------------------------------------------------E
    Pp1s67_251V6.1                    ----SA-----------------------------------------------------------------------------------------------------------------V
    Pp1s173_137V6.1                   ----ET-----------------------------------------------------------------------------------------------------------------S
    Pp1s116_90V6.1                    ---ETM---MKSE------------------------------------------------------AMAGVESAEVEAVDADVEMVEEEVA-EVEALSMGEGD-------RGSAEGGAI
    Pp1s138_130V6.1                   ----TV-----------------------------------------------------------------------------------------------------------------G
    Pp1s229_59V6.1                    ----YN-----------------------------------------------------------------------------------------------------------------E
    Pp1s88_123V6.1                    ---IGT------------------------------------------------------------------------------------------------------------------
    orange1.1g000286m                 ---NSS--SENPS------------------------------------------------------MEIDGDEG---------------KS-GKTS---------------------NI
    orange1.1g045956m                 ---TST-----------------------------------------------------------------------------------------------------------------S
    orange1.1g000014m                 ----TV-----------------------------------------------------------------------------------------------------------------G
    orange1.1g001688m                 ----ET-----------------------------------------------------------------------------------------------------------------S
    orange1.1g000012m                 ----TV-----------------------------------------------------------------------------------------------------------------G
    AT4G12570.1                       ----RS-----------------------------------------------------------------------------------------------------------------A
    AT4G38600.1                       ---SSG---DKVS------------------------------------------------------MQIGRDEI---------------ED-GK--------------------PSAA-
    AT1G55860.1                       ----TV-----------------------------------------------------------------------------------------------------------------G
    AT1G70320.1                       ----TV-----------------------------------------------------------------------------------------------------------------G
    AT3G53090.1                       ---TPT-----------------------------------------------------------------------------------------------------------------S
    AT3G17205.1                       ----ET-----------------------------------------------------------------------------------------------------------------A
    AT5G02880.1                       ---DCS---------------------------------------------------------------------------------------FIVG----------------KPV---E
    Si034011m                         ---TSA-----------------------------------------------------------------------------------------------------------------S
    Si016079m                         ---TSP---YDSG------------------------------------------------------LQIDRSDAI-------NLDPEDGLS-GKEL----------------NSDLPGD
    Si013562m                         ----PC-----------------------------------------------------------------------------------------------------------------P
    Si013264m                         ----AC-----------------------------------------------------------------------------------------------------------------P
    Si009242m                         ----ET-----------------------------------------------------------------------------------------------------------------A
    Si009164m                         ---HST---GNSG------------------------------------------------------TQIDGNGY--------------HLI-GIKH----------------ESESLFE
    Si024055m                         ---DNR------------------------------------------------------------------------------------------------------------------
    Si020966m                         ---ELP---------------------------------------------------------------------------------------CEAG----------------TDDAH--
    Si020939m                         ----TV-----------------------------------------------------------------------------------------------------------------G
    Si028891m                         ----PC-----------------------------------------------------------------------------------------------------------------P
    Si028637m                         ----TV-----------------------------------------------------------------------------------------------------------------G
    Thhalv10019984m                   ----ET-----------------------------------------------------------------------------------------------------------------A
    Thhalv10011172m                   ----TV-----------------------------------------------------------------------------------------------------------------G
    Thhalv10011171m                   ----TV-----------------------------------------------------------------------------------------------------------------G
    Thhalv10024192m                   ---NSG---DKVS------------------------------------------------------MQIDRDEI---------------ED-GK--------------------SSAAR
    Thhalv10028412m                   ----RS-----------------------------------------------------------------------------------------------------------------A
    Thhalv10012430m                   ---TR----------------------------------------------------------------------------------------SFVG----------------KSS---E
    Thhalv10010078m                   ---TTT-----------------------------------------------------------------------------------------------------------------S
    Ciclev10000001m                   ----TV-----------------------------------------------------------------------------------------------------------------G
    Ciclev10004231m                   ----ET-----------------------------------------------------------------------------------------------------------------S
    Ciclev10007219m                   ----TV-----------------------------------------------------------------------------------------------------------------G
    Ciclev10010897m                   ---DHS---------------------------------------------------------------------------------------SVTV----------------RKSLEIG
    Ciclev10010940m                   ---TST-----------------------------------------------------------------------------------------------------------------S
    Ciclev10027670m                   ---NSS--SENPS------------------------------------------------------MEIDGDEG---------------KS-GKTS---------------------NI
    Ciclev10014213m                   ----PC-----------------------------------------------------------------------------------------------------------------P
    GRMZM2G034622_T02                 ----ET-----------------------------------------------------------------------------------------------------------------A
    GRMZM2G124297_T01                 ---DSG------------------------------------------------------------------------------------------------------------------
    GRMZM2G411536_T03                 ----TV-----------------------------------------------------------------------------------------------------------------G
    GRMZM2G181378_T01                 ----PC-----------------------------------------------------------------------------------------------------------------P
    GRMZM2G049141_T01                 ---HSS---DNSG------------------------------------------------------MQIDANAD--------------DLIRSKNH----------------ESESLTE
    GRMZM2G080439_T01                 ----PC-----------------------------------------------------------------------------------------------------------------P
    GRMZM2G021299_T01                 ----TV-----------------------------------------------------------------------------------------------------------------G
    GRMZM2G328988_T01                 ---ELP---------------------------------------------------------------------------------------CKSG----------------THDTH--
    GRMZM2G331368_T02                 ----TV-----------------------------------------------------------------------------------------------------------------G
    GRMZM2G461948_T01                 ---TSA-----------------------------------------------------------------------------------------------------------------S
    GRMZM2G374574_T01                 ---TSS---YDSG------------------------------------------------------LHTDMKDVI-------RLDPEDGSS-GKEL----------------NTDLPGD
    Carubv10016604m                   ---TPT-----------------------------------------------------------------------------------------------------------------S
    Carubv10011657m                   ----TV-----------------------------------------------------------------------------------------------------------------G
    Carubv10007210m                   ----HS-----------------------------------------------------------------------------------------------------------------T
    Carubv10003974m                   ---NSG---DKVS------------------------------------------------------MQIDGEEI---------------ED-GK--------------------SSAAR
    Carubv10012881m                   ----ET-----------------------------------------------------------------------------------------------------------------A
    Carubv10000054m                   ---DCS---------------------------------------------------------------------------------------SFVG----------------KPG---E
    Carubv10000186m                   ----RS-----------------------------------------------------------------------------------------------------------------A
    Carubv10025730m                   ----TV-----------------------------------------------------------------------------------------------------------------G
    Bradi2g34820.1                    ----ET-----------------------------------------------------------------------------------------------------------------V
    Bradi2g37870.1                    ---DNA---------------------------------------------------------------------------------------RKPE----------------------G
    Bradi2g22927.2                    ---ELP---------------------------------------------------------------------------------------CKAV----------------TDSAH--
    Bradi4g07997.2                    ----TV-----------------------------------------------------------------------------------------------------------------G
    Bradi4g33520.1                    ----PC-----------------------------------------------------------------------------------------------------------------P
    Bradi1g12340.2                    ---TST-----------------------------------------------------------------------------------------------------------------S
    Bradi5g04567.1                    ---DSP---HDSD------------------------------------------------------S----------------------LE-AKKH----------------DS---AE
    Bradi3g00350.1                    ---SSP---YGSE------------------------------------------------------MEIDWSGMV-------HVNSEEELP-GKEL----------------DSDFPDD
    Aquca_017_00766.1                 ---DPK---------------------------------------------------------------------------------------LTSM----------------SNS-EVD
    Aquca_006_00259.1                 ---SSS---EKPA------------------------------------------------------MDIDGDEQH-------VRKGDNISD-GKKL----------------ESDYATG
    Aquca_028_00189.1                 ----AC-----------------------------------------------------------------------------------------------------------------P
    Aquca_027_00123.1                 ---DYS---------------------------------------------------------------------------------------NLTV----------------VKESIIE
    Aquca_007_00539.1                 ----TV-----------------------------------------------------------------------------------------------------------------G
    Aquca_003_00437.1                 ----ET-----------------------------------------------------------------------------------------------------------------S
    Aquca_019_00105.1                 ---TPT-----------------------------------------------------------------------------------------------------------------S
    MDP0000264736                     ----AC-----------------------------------------------------------------------------------------------------------------P
    MDP0000320720                     ---DHG---------------------------------------------------------------------------------------SFAS----------------GD----G
    MDP0000142676                     ----AC-----------------------------------------------------------------------------------------------------------------P
    MDP0000318443                     ----TV-----------------------------------------------------------------------------------------------------------------G
    MDP0000206447                     ----TV-----------------------------------------------------------------------------------------------------------------G
    MDP0000196216                     ---TST-----------------------------------------------------------------------------------------------------------------S
    MDP0000186793                     ----ET-----------------------------------------------------------------------------------------------------------------S
    MDP0000822588                     ---DRG---------------------------------------------------------------------------------------SFTT----------------GTS-HAG
    MDP0000924418                     ---TST-----------------------------------------------------------------------------------------------------------------S
    MDP0000320505                     ---NSS--MEKTS------------------------------------------------------MDIDGDEH---------------KD-GKSN-----------------------
    MDP0000307848                     ----TV-----------------------------------------------------------------------------------------------------------------G
    MDP0000301275                     ---NSS--MEKTS------------------------------------------------------MDIDGDEP---------------KD-GKSN-----------------------
    MDP0000317971                     ----TV-----------------------------------------------------------------------------------------------------------------G
    Bra022201                         ----ET-----------------------------------------------------------------------------------------------------------------V
    Bra028860                         ---DPS---------------------------------------------------------------------------------------SLAG----------------KP-----
    Bra038022                         ----TV-----------------------------------------------------------------------------------------------------------------G
    Bra021231                         ----ET-----------------------------------------------------------------------------------------------------------------V
    Bra005748                         ---DRS---------------------------------------------------------------------------------------SFAG----------------KPK---E
    Bra000779                         ----RS-----------------------------------------------------------------------------------------------------------------A
    Bra029461                         ----RS-----------------------------------------------------------------------------------------------------------------A
    Bra027850                         ----TV-----------------------------------------------------------------------------------------------------------------G
    Bra040685                         ---TTT-----------------------------------------------------------------------------------------------------------------S
    Bra010737                         ---NSG---DKLS------------------------------------------------------MQTDRDEI---------------QD-GK--------------------SAAAR
    Medtr2g025830.1                   ----AC-----------------------------------------------------------------------------------------------------------------P
    Medtr2g025950.1                   ----AC-----------------------------------------------------------------------------------------------------------------P
    Medtr2g025810.1                   ----AC-----------------------------------------------------------------------------------------------------------------P
    Medtr2g033040.1                   STFGET-----------------------------------------------------------------------------------------------------------------A
    Medtr2g025790.1                   ----AC-----------------------------------------------------------------------------------------------------------------P
    Medtr2g025930.1                   ----AC-----------------------------------------------------------------------------------------------------------------P
    Medtr7g100670.1                   ---DTQ------------------------------------------------------------------------------------------------------------------
    Medtr5g066710.1                   ----TV-----------------------------------------------------------------------------------------------------------------G
    Medtr4g073370.1                   ---GSD------------------------------------------------------------HMEIDGDEK---------------KK-KS------------------SEGNIAR
    Medtr4g133120.1                   ----AC-----------------------------------------------------------------------------------------------------------------P
    Vocar20002255m                    ----YN-----------------------------------------------------------------------------------------------------------------E
    Vocar20010178m                    ----AV-----------------------------------------------------------------------------------------------------------------P
    Vocar20006334m                    --FNRT------------------------------------------------------------------------------------------------------------------
    Vocar20007555m                    ----TA-----------------------------------------------------------------------------------------------------------------G
    Vocar20012583m                    ---EEQ---DDTQQQQEPQQQQEQQEQQEGDTEAPQEEPEAMRLGEGEDAEAKGLRASASASASPDTMEVDGGVGAAAAAAPPPLPLQQRLS-GAVQGALATGG-LVSVPSRRGEAATHE
    Vocar20003001m                    ----AN-----------------------------------------------------------------------------------------------------------------S
    Vocar20004069m                    ----YD-----------------------------------------------------------------------------------------------------------------E
    Vocar20000780m                    ----ST-----------------------------------------------------------------------------------------------------------------P
    Vocar20004842m                    ----AD-----------------------------------------------------------------------------------------------------------------P
    Vocar20014908m                    ----GA-----------------------------------------------------------------------------------------------------------------A
    Lus10032589                       ----TV-----------------------------------------------------------------------------------------------------------------G
    Lus10035589                       ---TST-----------------------------------------------------------------------------------------------------------------S
    Lus10005068                       ---NMS--SDKHL------------------------------------------------------NEGD--------------------------------------------AATTG
    Lus10010493                       ----AC-----------------------------------------------------------------------------------------------------------------P
    Lus10027841                       ---NMS--SDKHL------------------------------------------------------NDGD--------------------------------------------AATTG
    Lus10019908                       ---KHF---------------------------------------------------------------------------------------SSTV----------------KD-----
    Lus10032830                       ----TV-----------------------------------------------------------------------------------------------------------------G
    Lus10017098                       ------------------------------------------------------------------------------------------------------------------------
    Lus10002605                       ----TV-----------------------------------------------------------------------------------------------------------------G
    Lus10008636                       ---TST-----------------------------------------------------------------------------------------------------------------S
    Eucgr.A01178.1                    ---DHG---------------------------------------------------------------------------------------SFSS----------------NKVMETH
    Eucgr.A01586.1                    ---TST-----------------------------------------------------------------------------------------------------------------E
    Eucgr.B03986.1                    ----ET-----------------------------------------------------------------------------------------------------------------A
    Eucgr.D01414.1                    ----AC-----------------------------------------------------------------------------------------------------------------P
    Eucgr.D01416.1                    ----AC-----------------------------------------------------------------------------------------------------------------P
    Eucgr.F02160.1                    ----TV-----------------------------------------------------------------------------------------------------------------G
    Eucgr.I01410.2                    ---TSS---EKRA------------------------------------------------------VGVDLNEQ---------------SN-GKST----------------STDSAVE
    Pavirv00038038m                   ----TV-----------------------------------------------------------------------------------------------------------------G
    Pavirv00031244m                   ---TTP---YDSG------------------------------------------------------LQIDMNDVI-------NLDPEDSLS-GKKL----------------SSDLPDD
    Pavirv00010575m                   ----PC-----------------------------------------------------------------------------------------------------------------P
    Pavirv00004902m                   ----AC-----------------------------------------------------------------------------------------------------------------P
    Pavirv00020428m                   ---DNC------------------------------------------------------------------------------------------------------------------
    Pavirv00067430m                   ----PC-----------------------------------------------------------------------------------------------------------------P
    Pavirv00058663m                   ----TV-----------------------------------------------------------------------------------------------------------------G
    Pavirv00067620m                   ---TTP---YDSG------------------------------------------------------LQIDMNDVI-------NLDPEDGLS-GKKL----------------SLDLPGD
    Pavirv00029557m                   ----TV-----------------------------------------------------------------------------------------------------------------G
    Pavirv00023469m                   ----ET-----------------------------------------------------------------------------------------------------------------A
    Pavirv00024250m                   ---ELP---------------------------------------------------------------------------------------CEAG----------------TDDAH--
    Pavirv00023205m                   ---DLP---------------------------------------------------------------------------------------CEAG----------------TDDAH--
    Pavirv00029138m                   ---DNC------------------------------------------------------------------------------------------------------------------
    LOC_Os03g47949.1                  ---ASA-----------------------------------------------------------------------------------------------------------------S
    LOC_Os02g01170.1                  ---SSP---SDTG------------------------------------------------------MQIDRS-----------ASPDDDLA-AKEL----------------SSDLPDN
    LOC_Os09g07900.1                  ----TV-----------------------------------------------------------------------------------------------------------------G
    LOC_Os12g24080.1                  ----TV-----------------------------------------------------------------------------------------------------------------G
    LOC_Os05g38830.1                  ---ELS---------------------------------------------------------------------------------------GEAG----------------LDNVH-G
    LOC_Os05g03100.1                  ---DDT---------------------------------------------------------------------------------------SQEC-----------------------
    LOC_Os05g06690.1                  ----ET-----------------------------------------------------------------------------------------------------------------A
    PGSC0003DMT400075387              ----AC-----------------------------------------------------------------------------------------------------------------P
    PGSC0003DMT400021802              ----AC-----------------------------------------------------------------------------------------------------------------P
    PGSC0003DMT400031190              ---GLSLTSNEHS------------------------------------------------------MEVHIDNK-----------------------------------------LSRS
    PGSC0003DMT400072624              ----AC-----------------------------------------------------------------------------------------------------------------P
    Glyma14g36180.1                   ----TV-----------------------------------------------------------------------------------------------------------------G
    Glyma02g38020.2                   ----TV-----------------------------------------------------------------------------------------------------------------G
    Glyma12g03640.1                   ---GSS---EKYQ------------------------------------------------------MEIDGDEK---------------KM-KN------------------SEGSFVG
    Glyma11g11490.1                   ---GSS---EKYQ------------------------------------------------------MKIDGDEK---------------KM-KR------------------SEGSFVG
    Glyma06g00600.1                   ---YSS---EKHQ------------------------------------------------------MEIDRDEK---------------KK-KSDG----------------SGPNLAG
    Glyma06g10360.1                   ----TV-----------------------------------------------------------------------------------------------------------------G
    Glyma04g00530.1                   ---YSS---DKHQ------------------------------------------------------MEIDGDEK---------------KK-KSEG----------------SGPNLAG
    Glyma04g10481.1                   ----TV-----------------------------------------------------------------------------------------------------------------G
    Glyma08g09270.3                   ----TV-----------------------------------------------------------------------------------------------------------------G
    Glyma17g01210.2                   ----AC-----------------------------------------------------------------------------------------------------------------P
    Glyma17g04180.1                   ----ET-----------------------------------------------------------------------------------------------------------------A
    Glyma13g19981.1                   ---DAS---------------------------------------------------------------------------------------SFTL----------------KTNMEAE
    Glyma05g26360.1                   ----TV-----------------------------------------------------------------------------------------------------------------G
    Glyma19g37310.1                   ---NST-----------------------------------------------------------------------------------------------------------------S
    Glyma15g14591.1                   ----ET-----------------------------------------------------------------------------------------------------------------A
    Glyma03g34650.2                   ---TST-----------------------------------------------------------------------------------------------------------------S
    Glyma10g05620.3                   ---DDS---------------------------------------------------------------------------------------SFTL----------------KTNLQAE
    Glyma07g36390.1                   ----ET-----------------------------------------------------------------------------------------------------------------A
    Glyma07g39546.1                   ----AC-----------------------------------------------------------------------------------------------------------------P
    Gorai.010G033100.1                ----TV-----------------------------------------------------------------------------------------------------------------G
    Gorai.010G186800.1                ----PC-----------------------------------------------------------------------------------------------------------------S
    Gorai.009G278900.1                ----TV-----------------------------------------------------------------------------------------------------------------G
    Gorai.009G228200.1                ----TV-----------------------------------------------------------------------------------------------------------------G
    Gorai.009G183200.1                ----PC-----------------------------------------------------------------------------------------------------------------A
    Gorai.009G420400.1                ---DHC---------------------------------------------------------------------------------------SFIT----------------STTLP-T
    Gorai.002G100900.1                ----TV-----------------------------------------------------------------------------------------------------------------G
    Gorai.002G196900.1                ---NST--WDKSI------------------------------------------------------MEIDGDEE---------------KN-EKTA--------------------GSV
    Gorai.002G245000.1                ---TST-----------------------------------------------------------------------------------------------------------------S
    Gorai.002G003200.1                ---NST--WNKSL------------------------------------------------------MEIDGDEK---------------KE-GKTA------------------GSTTI
    Gorai.011G204200.1                ----ET-----------------------------------------------------------------------------------------------------------------A
    Gorai.008G035900.1                ---NST--WNKSV------------------------------------------------------MEIDGDGD---------------KN-GKIA------------------GSATI
    Gorai.006G265700.1                ----ET-----------------------------------------------------------------------------------------------------------------A
    Potri.010G150000.3                ----ET-----------------------------------------------------------------------------------------------------------------S
    Potri.009G134300.1                ---NSA--AEKPS------------------------------------------------------MEIDGDDD---------------KN-GKSN----------------NESGTAV
    Potri.004G174700.1                ---NSA--AGKPS------------------------------------------------------MEIDGDDE---------------KN-GKSN----------------NGSGTAV
    Potri.011G094100.1                ----TV-----------------------------------------------------------------------------------------------------------------G
    Potri.006G132000.1                ---DHI---------------------------------------------------------------------------------------SFTT----------------SETLQAE
    Potri.006G011700.1                ----AC-----------------------------------------------------------------------------------------------------------------P
    Potri.016G085200.3                ---DHI---------------------------------------------------------------------------------------SFPT----------------IENLQAE
    Potri.016G096500.1                ---TST-----------------------------------------------------------------------------------------------------------------S
    Potri.016G012900.1                ----AC-----------------------------------------------------------------------------------------------------------------P
    Potri.002G110500.1                ----TV-----------------------------------------------------------------------------------------------------------------G
    Potri.008G101300.1                ----ET-----------------------------------------------------------------------------------------------------------------S
    Potri.001G368600.1                ----TV-----------------------------------------------------------------------------------------------------------------G
    Phvul.003G084200.1                ----AC-----------------------------------------------------------------------------------------------------------------P
    Phvul.003G118500.1                ----ET-----------------------------------------------------------------------------------------------------------------A
    Phvul.009G119700.1                ---HSS---NKHV------------------------------------------------------MEVDGDEK---------------RE-NSVV----------------SRPDLAG
    Phvul.009G034900.1                ----TV-----------------------------------------------------------------------------------------------------------------G
    Phvul.011G035200.1                ---GFS---EKYP------------------------------------------------------MEIDGNER---------------KM-KS------------------SEGSFAG
    Phvul.008G183200.1                ----TV-----------------------------------------------------------------------------------------------------------------G
    Phvul.007G163300.1                ---DVS---------------------------------------------------------------------------------------SFIL----------------KSNLQAE
    Phvul.007G163400.1                ---E--------------------------------------------------------------------------------------------------------------ANLQAG
    Phvul.001G184300.1                ---TST-----------------------------------------------------------------------------------------------------------------S
    Phvul.006G120900.1                ----ET-----------------------------------------------------------------------------------------------------------------A
    Phvul.006G142800.1                ----AC-----------------------------------------------------------------------------------------------------------------P
    Phvul.002G189700.1                ----TV-----------------------------------------------------------------------------------------------------------------G
    mrna26562.1-v1.0-hybrid           ----TV-----------------------------------------------------------------------------------------------------------------G
    mrna05017.1-v1.0-hybrid           ----ET-----------------------------------------------------------------------------------------------------------------S
    mrna09579.1-v1.0-hybrid           ---DGG---------------------------------------------------------------------------------------FFTT----------------GIS-HAE
    mrna30084.1-v1.0-hybrid           ---TST-----------------------------------------------------------------------------------------------------------------S
    mrna07649.1-v1.0-hybrid           ---NSS--LEKAP------------------------------------------------------MDIDGDDQ---------------KD-GKNN-----------------------
    mrna20590.1-v1.0-hybrid           ----TV-----------------------------------------------------------------------------------------------------------------G
    mrna19775.1-v1.0-hybrid           ----AC-----------------------------------------------------------------------------------------------------------------P
    Solyc04g076620.2.1                ----TV-----------------------------------------------------------------------------------------------------------------G
    Solyc10g083470.1.1                ----AC-----------------------------------------------------------------------------------------------------------------P
    Solyc10g055450.1.1                ---GLSLTSNEHS------------------------------------------------------VEVHIDNK-----------------------------------------LSRS
    Solyc07g065630.2.1                ----TV-----------------------------------------------------------------------------------------------------------------G
    Solyc05g054080.2.1                ----AC-----------------------------------------------------------------------------------------------------------------P
    Solyc01g057900.2.1                ----ET-----------------------------------------------------------------------------------------------------------------A
    Solyc01g111530.2.1                ---SSS--SSGHS------------------------------------------------------MEVGVDEK-----------------------------------------LSGG
    Solyc12g094560.1.1                ----AC-----------------------------------------------------------------------------------------------------------------P
    Solyc09g005150.1.1                ----SC-----------------------------------------------------------------------------------------------------------------P
    Solyc09g007310.2.1                ---DYL---------------------------------------------------------------------------------------AHAS----------------MSV--EE
    Solyc09g005160.1.1                ----SC-----------------------------------------------------------------------------------------------------------------P
    Solyc09g008700.1.1                ---TLT-----------------------------------------------------------------------------------------------------------------S
    69212                             ----SN-----------------------------------------------------------------------------------------------------------------D
    70217                             ----SA-------------------------------------------------------------------------------------------------------P---------S
    48481                             ----VD-----------------------------------------------------------------------------------------------------------------E
    19835                             ----ET-----------------------------------------------------------------------------------------------------------------P
    213597                            ----SP-----------------------------------------------------------------------------------------------------------------S
    174890                            ---VPN---------------------------------------------------------------------------------------ADGT---------------------GS
    154462                            ----PT-----------------------------------------------------------------------------------------------------------------S
    22875                             ----HD-----------------------------------------------------------------------------------------------------------------D
    172918                            ---DED---------------------------------------------------------------------------------------GASG-----------------------
    29762                             ----AT-----------------------------------------------------------------------------------------------------------------P
    67182                             ---DDS---AASK------------------------------------------------------VATGAAVA------------------SKAE---DSRIVVSTLPAQAAAGENGR
    58691                             ----FN-----------------------------------------------------------------------------------------------------------------E
    16350                             ----MD-----------------------------------------------------------------------------------------------------------------P
    35876                             ----FQ-----------------------------------------------------------------------------------------------------------------P
    15978                             ----ST-----------------------------------------------------------------------------------------------------------------A
    37891                             -----V-----------------------------------------------------------------------------------------------------------------P
    57759                             ---DED---------------------------------------------------------------------------------------GDDG-----------------------
    59359                             ----SP-----------------------------------------------------------------------------------------------------------------S
    108435                            ----ET-----------------------------------------------------------------------------------------------------------------P
    87459                             ----WD-----------------------------------------------------------------------------------------------------------------D
    60437                             ----LD-----------------------------------------------------------------------------------------------------------------E
    60965                             -GLEES--------------------------------------G--------------------------------------------------------------------------G
    83330                             ---APV---------------------------------------------------------------------------------------DGKP---------------------GP
    62795                             ---SST-----------------------------------------------------------------------------------------------------------------A
    91960                             ----SN-----------------------------------------------------------------------------------------------------------------D
    52147                             ----LS-----------------------------------------------------------------------------------------------------------------H
    36723                             ----ET-----------------------------------------------------------------------------------------------------------------P
    31158                             -----------------------------------------------------------------------------------------------------------------------Q
    39499                             ---SGV---------------------------------------------------------------------------------------AN-------------------------
    41776                             ----ET-----------------------------------------------------------------------------------------------------------------P
    41898                             ----LS-----------------------------------------------------------------------------------------------------------------H
    43113                             ----SN-----------------------------------------------------------------------------------------------------------------A
    Thecc1EG022084t1                  ---DYR---------------------------------------------------------------------------------------SIIT----------------SETLPVV
    Thecc1EG022374t1                  ---TST-----------------------------------------------------------------------------------------------------------------S
    Thecc1EG030368t1                  ----PC-----------------------------------------------------------------------------------------------------------------S
    Thecc1EG030623t1                  ----TV-----------------------------------------------------------------------------------------------------------------G
    Thecc1EG021434t2                  ----ET-----------------------------------------------------------------------------------------------------------------A
    Thecc1EG034540t1                  ----TV-----------------------------------------------------------------------------------------------------------------G
    Thecc1EG006633t1                  ---NST--WDKSV------------------------------------------------------MEIDGDEE---------------KN-GKAA------------------GSATI
    Cre08.g364550.t1.3                ----YS-----------------------------------------------------------------------------------------------------------------E
    Cre07.g312900.t1.3                ---EEQ---DDTQKQKQDKQR------------------------AGSGGGDGGAKPSAPTPGSPDRMDVDGGPATTAAA--------QGQG-GSTPPRVQSGSVLVSVPSRRGEAANHD
    g11539.t1                         ----SD-----------------------------------------------------------------------------------------------------------------P
    Cre06.g280300.t1.3                ----SN-----------------------------------------------------------------------------------------------------------------A
    Cre02.g099100.t1.3                ----YD-----------------------------------------------------------------------------------------------------------------P
    Cre03.g159200.t1.2                ----ST-----------------------------------------------------------------------------------------------------------------P
    Cre01.g022100.t1.2                ----LC--E--------------------------------------------------------------------------------------------------------------G
    Cre01.g012450.t1.3                ----TV-----------------------------------------------------------------------------------------------------------------P
    Cre10.g433900.t1.3                ----TA-----------------------------------------------------------------------------------------------------------------G
    Cre12.g533750.t1.3                ----GA-----------------------------------------------------------------------------------------------------------------A
    Cre12.g548100.t1.3                --FNRT------------------------------------------------------------------------------------------------------------------

    Selected Cols:                                                                                                                                            

    Gaps Scores:                                                                                                                                              

                                             370       380       390       400       410       420       430       440       450       460       470       480
                                      =========+=========+=========+=========+=========+=========+=========+=========+=========+=========+=========+=========+
    Sb01g011845.1                     D---------TSLIPSN---------------------------S---A-KLL-DN---------------GIDMIEFLGRVVGKALYEG---------ILLDYSFSPVFVQK-LL----
    Sb02g016200.1                     N--------NATFQPNP---------------------------N---S-VFQ-TE---------------HLSYFKFVGRVVAKALFDG---------QLLDVHFTRSFYKH-IL----
    Sb04g000340.1                     G-RHL-IQAPLGLFPRP-----------WPPKVD----------A---SEGSRLFK---------------VLEHFRLIGQVMAKVLQDG---------RLLDLPLSTAFYKL-IL----
    Sb06g003290.1                     S-RNI-VQAPLGLFPQP-----------WPPSAA----------A---SEGSKFFK---------------VVEYFRLVGRTMAKALQDG---------RLLDLPLSTAFYKL-LL----
    Sb08g012560.1                     N--------DLTFQPNP---------------------------N---S-VYQ-TE---------------HLSYFKFVGRVVGKALFDG---------QLLDAHFTRSFYKH-IL----
    Sb09g002120.1                     E-HGF-IHAPFGLFPKP-----------WSPSGT----------S---SQGVDFTN---------------MLQKFKLLGNLVARAVLDG---------RILDIPLSKAFYKV-ML----
    Sb09g004530.1                     D---------HLLYPNP---------------------------G---S-GLVHEL---------------HLQYFHFLGSLLGKAMYEG---------ILVDLPFATFFLSK-LK----
    Sb09g022820.1                     V-SEF-VVAPNGLFPRP-----------WSASAD----------S---A---SFQE---------------VSKRFHLLGLVVAKAIKDN---------RILDIPFSKAFYKL-IL----
    73381                             D----------CYQPNP---------------------------S---S-ASC-PG---------------HLSYFKFVGRLAAKAIIDS---------QKLDIRFTRSFYKH-IL----
    50844                             N-------DRRRFYPNP---------------------------A---S-GVN-PG---------------HLTYFKFCGRVIALAMMHR---------VQVDVTFALFFFKQ-LA----
    89794                             E---------RFLFPHP---------------------------A---A-SSL-GQ---------------GLRTIEFLGRIVGKALYEG---------ILLEHLFSPVFVSK-IL----
    3542                              D---------DRYQPYP---------------------------S--LS-----DS---------------NLSDFKFVGQLVAKAIVDD---------ERLDLRFTRAFYKH-IL----
    76253                             E--------ARCFWFNATP-------------------------S---E-LNF--D-----------------TEFELVGILLGLAIYNG---------HILDLHFPTVVYKK-LL----
    443962                            D---------HLLYPNP---------------------------A---S-HMVNDE---------------HLQYFEFFGKILGKAMFEG---------ILVDIPFAMFFLRK-LR----
    181768                            E--------SRYFWFNS---------------------------N---S-IET--D-----------------LEFQLVGIILGLAIYNG---------VILDVHFPLVVYKK-LL----
    407700                            --------RERATYWHPRMP-------V----------------H---S-RTLRQE---------------ERDVAIFVGWLLFQAFECN---------AQVSRGCTIATLAI-AA----
    146155                            N-IDY-VTAPHGLFPRP-----------WHP-------------A---STDARYMK---------------TVEHFRLLGRVMAKALQDG---------RLLDLPFSIPFYKL-VL----
    154179                            N--------ESTFQPNP---------------------------N---S-VYQ-TE---------------HLSYFKFVGRVVSKALFDG---------QLLDVYFTRSFYKH-IL----
    943823                            N-IDI-VLAPLGLFPRP-----------WPSTAD----------I---SEGGQFHK---------------VIEYFRLLGRVMAKALQDG---------RLLDVPLSTAFYKL-IL----
    487067                            H-SGV-LASSSGLFPRP-----------WSGTS------------------TT-SD---------------VLQKFVLLGTVVAKALQDG---------RVLDLPFSKAFYKL-IL----
    485684                            D---------RLLVPSP---------------------------S---A-RHL-EN---------------GIQMIEFLGRIVGKALYEG---------ILLDYSFSHVFIQK-LL----
    490058                            D-------DFRRFSPNP---------------------------A---S-KVD-PL---------------HPDFFEFTGRVIALALMHK---------VQVGVLFDRVFFLQ-LA----
    479191                            D---------HMLYPNP---------------------------G---S-GMIHDQ---------------HLQFFHFLGSLLAKAMFEG---------ILVDIPFATFFLSK-LK----
    916552                            N--------DSTFQPNP---------------------------N---S-VYQ-TE---------------HLSYFKFVGRVVGKALFDG---------QLLDVHFTRSFYKH-IL----
    940321                            E-------HARRFSPNP---------------------------G---F-MVN-TM-------------------FEFAGRVMGVALMHE---------IQLNVFFDRIFFLQ-LA----
    474651                            N--------DATFQPNP---------------------------N---S-VYQ-TE---------------HLSYFKFVGRMVAKALFDG---------QLLDVYFTRSFYKH-IL----
    915021                            D-------DSRRFSPHP---------------------------N---P-LME-EN---------------YLQKYRFSGRIISMALKHE---------MQVGILFDPLFFLH-LA----
    evm.model.supercontig_146.73      ------------------------------------------------------------------------------------KAMFEG---------ILVDIPFATFFLSK-LK----
    evm.model.supercontig_21.42       N--------ESTFQPNP---------------------------N---S-VYQ-TE---------------HLSYFKFVGRVVGKALFDG---------QLLDVHFTRSFYKH-IL----
    evm.model.supercontig_37.145      D-GDM-VQAPLGLFPRP-----------WPPNAD----------I---SEGNQFYK---------------VIEHFRLVGRVMAKALQDG---------RLLDLPLSTAFYKL-VL----
    evm.model.supercontig_5.113       D---------RLLIPNS---------------------------A---A-RHL-EN---------------GIQMIEFLGRVVGKALYEG---------ILLDYSFSHVFVQK-LL----
    evm.model.supercontig_959.1       N--------NVTFQPNP---------------------------N---S-VYQ-TE---------------HLSYFKFVGRVVAKALFDG---------QLLDVYFTRSFYKH-IL----
    29206.m000140                     N-------DRRRFFPNP---------------------------A---S-KVE-PL---------------HLDYFTFCGRVIALALMHK---------VQVGIVFDRVFFLQ-LA----
    29596.m000712                     N--------ESTFQPNP---------------------------N---S-VYQ-TE---------------HLSYFKFIGRVVGKALFDG---------QLLDVHFTRSFYKH-IL----
    29602.m000214                     G-ADV-VQAPLGLFPRP-----------WPPSAD----------A---SEGSQFYK---------------AVEYFRLVGRVMAKALQDG---------RLLDLPLSTAFYKL-VL----
    29629.m001405                     N--------NATFQPNP---------------------------N---S-VYQ-TE---------------HLSYFKFVGRVVAKALFDG---------QLLDVYFTRSFYKH-IL----
    29805.m001489                     D-AGI-VMSPFGLFPCP-----------WSSTLD----------T---SDGIQFSE---------------VIKKFFLMGQLVAKALQDG---------RVLDLPFSKAFYKL-IL----
    29815.m000491                     E---------RLLIPNP---------------------------S---A-KYL-EN---------------GIQMIEFLGRVVGKALYEG---------ILLDYSFSHVFVQK-LL----
    29889.m003352                     D---------HLLYPNP---------------------------G---S-GMIHEQ---------------HLQFFHFLGTLLAKAMFEG---------ILVDIPFATFFLSK-LK----
    Cucsa.042120.1                    N--------NATFQPNP---------------------------N---S-VYQ-TE---------------HLSYFKFVGRVVAKALFDG---------QLLDVYFTRSFYKH-IL----
    Cucsa.044750.1                    N-------DRRRFFPNP---------------------------A---S-KVD-PM---------------HLNYFNFSGRVIALALMYK---------VQVGVVFDRVFFLQ-LA----
    Cucsa.160480.1                    N--------DSTFQPNP---------------------------N---S-AYQ-TE---------------HLSYFKFVGRVVGKALYDG---------QLLDVHFTRSFYKH-IL----
    Cucsa.234290.1                    N-IDI-IQSPLGLFPRP-----------WPANAD----------S---SDGSQFSK---------------VIEYFRLVGRVMAKALQDG---------RLLDLPLSTAFYKL-VL----
    Cucsa.307200.1                    D-RET-TESPFGLFPRP-----------WPSTLD--------------TDKLHLPE---------------VMKKFVLLGQIVAKAIQDC---------RVLDIYFSKAFYKL-IL----
    Cucsa.378730.1                    D---------RHLIPNA---------------------------A---A-RYL-DN---------------GIQMIEFLGRVVGKALYEG---------ILLDYSFSHVFVHK-LL----
    ppa000451m                        D---------RLLIPNS---------------------------S---A-RYL-EN---------------GIQMIEFLGRVVGKALYEG---------ILLDYSFSHVFIQK-LL----
    ppa000008m                        N--------NATFQPNP---------------------------N---S-VYQ-TE---------------HLSYFKFVGRVVAKALFDG---------QLLDVYFTRSFYKH-IL----
    ppa001143m                        H-------DHRRFYPNP---------------------------A---S-KVD-PL---------------HLEYFTFAGRVIALALMHK---------VQVGIVFDRVFFQQ-LA----
    ppa000674m                        D---------HLLYPNP---------------------------G---S-GMIHEQ---------------HLQFFQFLGILLAKAMFEG---------ILVDIPFATFFLSK-LK----
    ppa000169m                        D-TGI-LICPFGLFPRP-----------WSSTLD----------T---SDGIHFSE---------------VMKKFVLLGQIVGKALQDG---------RVLDLHFSKAFYKL-IL----
    ppa000009m                        N--------ESTFQPNP---------------------------N---S-VYQ-TE---------------HLSYFKFVGRVVGKALFDG---------QLLDVHFTRSFYKH-IL----
    ppa000080m                        --GDI-VQAPLGLFPRP-----------WPLNAV----------A---SDGSQFSK---------------VIEYFRLVGRVMAKALQDG---------RLLDLPLSTAFYKL-LL----
    mgv1a001314m                      N-------DRRRFYPNP---------------------------A---S-KVD-PL---------------HLKYFSFSGKVIALALMHK---------VQVGIVLDRVFFLQ-LA----
    mgv1a000078m                      G-KDI-IHAPLGLFPCP-----------WPPNAD----------T---SAASQFSK---------------AIEYYRLLGRVMAKALQDG---------RLLDLPLSSAFYKL-VL----
    mgv1a000005m                      N--------NATFQPNP---------------------------N---S-VYQ-TE---------------HLSYFRFVGRVVAKALFDG---------QLLDVHFTRSFYKH-IL----
    mgv11b024345m                     D---------QRLYPSP---------------------------I---S--YVQDN---------------HLQLFEFVGRMLGKAVYEG---------IVVDVPFASFFLSQ-LL----
    mgv1a000436m                      D---------RLLIPNP---------------------------T---A-RFL-DN---------------GIQMVEFLGRIVGKALYEG---------ILLDFYFSHVFVQK-LL----
    mgv1a000163m                      ------LVSLFGLFPRP-----------WSPSSS----------------STVHSE---------------VIKKFTLLGHIVAKAIQDG---------RLLDLPFAKAFYKL-IL----
    GSVIVT01003328001                 N-------DRRRFFPNP---------------------------A---S-EVD-PM---------------HLQYFRFSGRVIALALMHK---------VQVGVVFDRVFFLQ-LA----
    GSVIVT01009206001                 N--------ESTFQPNP---------------------------N---S-VYQ-TE---------------HLSYFKFVGRVVGKALFDG---------QLLDVHFTRSFYKH-IL----
    GSVIVT01014698001                 N--------NSTFQPNP---------------------------N---S-VYQ-TE---------------HLSYFKFVGRVVAKALFDG---------QLLDVYFTRSFYKH-IL----
    GSVIVT01018731001                 --GVL-VQAPLGLFPRP-----------WSPTAD----------A---SDGSPFAK---------------VIEYYHLLGQVMAKALQDG---------RLLDLPLSMAFYKL-VL----
    GSVIVT01024033001                 ---DI-VQAPLGLFPRP-----------WPPNAD----------A---SDGSQFSK---------------VIEHFRLVGRVIAKALQDG---------RLLDLPLSTALYKL-VL----
    GSVIVT01025537001                 -------------------------------------------------------------------------------CQVVAKALQDG---------RVLDLPFSKAFYKLAIL----
    GSVIVT01033734001                 D---------RLLVPNT---------------------------A---A-RFL-EN---------------GTQMIEFLGKVVGKALYEG---------ILLDYSFSHVFIQK-LL----
    GSVIVT01034942001                 D---------HLLYPNP---------------------------G---S-GMIHEQ---------------HLQFFHFLGTVLGKAMFEG---------ILVDIPFATFFLSK-LK----
    cassava4.1_000003m                N--------NATFQPNP---------------------------N---S-VYQ-TE---------------HLSYFKFVGRVVAKALFDG---------QLLDVYFTRSFYKH-IL----
    cassava4.1_000080m                D-ADV-VQAPLGLFPRP-----------FPPNAD----------A---SEGSQFYK---------------VVEYFRLVGRVMAKALQDG---------RLLDLPLSTAFCKL-VL----
    cassava4.1_002295m                D---------HLLYPNP---------------------------G---S-GMIHEQ---------------HLQFFHFLGTLLAKAMFEG---------ILVDIPFATFFLSK-LK----
    cassava4.1_000006m                N--------ESTFQPNP---------------------------N---S-VYQ-TE---------------HLSYFKFVGRVVGKALFDG---------QLLDVHFTRSFYKH-IL----
    cassava4.1_000011m                N--------DSTFQPNP---------------------------N---S-VYQ-TE---------------HLSYFKFVGRVVGKALFDG---------QLLDVHFTRSFYKH-IL----
    cassava4.1_000177m                D-SGS-LTSPFGLFPRP-----------WPSSLD----------S---SDEIQFSE---------------VIKKFFLLGQVVAKALQDG---------RVLDLPFSKAFYKL-IL----
    Pp1s205_47V6.1                    N-------DRRRFFPNS---------------------------A---S-GVN-PG---------------HLTYFRFCGRVIALALMHR---------VQMDVVFALSFFKQ-LA----
    Pp1s148_98V6.1                    N-------DRRRFFPNP---------------------------A---S-GVN-PG---------------HLTYFRFCGRVIALALMHR---------VQMDVVFALSFFKQ-LA----
    Pp1s103_43V6.1                    E---------GLLFPHA---------------------------A---A-ASL-GH---------------GLRMLEFLGRIVGKALYEG---------ILLEYSFSPLFVSK-IL----
    Pp1s42_128V6.2                    N--------ESTFQPNP---------------------------N---S-VYQ-TE---------------HLSYFKFVGRVVAKALIDG---------QLLDVYFTRSFYKH-IL----
    Pp1s263_1V6.1                     -----------------------------------------------------------------------SAEHFRLLGRVMAKALQDG---------RLLDVPLSTAFCKI-IL----
    Pp1s263_20V6.1                    D---------GLLLPRA---------------------------A---A-GNL-DS---------------ELRKLKFLGQIVGKALYEG---------ILLEHSLSPLFISK-VL----
    Pp1s15_454V6.1                    E---------GYLFPHA---------------------------A---A-GSL-GH---------------GLRMLEFLGRIVGKALYEG---------ILLEYSFSPLFISK-LL----
    Pp1s67_251V6.1                    D--------DYTFQINP---------------------------N---S-GVN-QD---------------HLDFFRFIGTIVGKALYDG---------CLFDAHFTRLVYKR-IL----
    Pp1s173_137V6.1                   D---------HLLYPNP---------------------------A---S-QMVSDE---------------HLQYFEFFGKMLGKAMFEG---------ILVDIPFATFFLSK-LR----
    Pp1s116_90V6.1                    S-GEY-VTAPQGLFPRP-----------WHPDTD----------P---GSNKKYWK---------------VLEHFRLLGRVMAKALQDG---------RLLDVPMSTAFYKI-IL----
    Pp1s138_130V6.1                   N--------ESTFQPNP---------------------------N---S-VYQ-TE---------------HLSYFKFVGRVVAKALFDG---------QLLDVYFTRSFYKH-IL----
    Pp1s229_59V6.1                    D--------TRHFWFNR---------------------------D---S-MET--K-----------------HEFRLVGNILGLAIYNG---------VILDIHFPKAVYKK-LL----
    Pp1s88_123V6.1                    E---------GFLCPHA---------------------------A---A-GTV-GH---------------GLPMLEFLGRIVGKALYEE---------ILLEYSFSPLFLSK-LL----
    orange1.1g000286m                 S-GDL-VHAPLGLFPRP-----------WPPSAD----------A---SEGGQFSK---------------VIEYFRLLGRVMAKALQDG---------RLLDLPFSTAFYKL-VL----
    orange1.1g045956m                 D---------RLLIPNA---------------------------A---A-RYL-EN---------------GIQMFEFLGRVVGKALYEG---------ILLDYAFSHVFVQK-LL----
    orange1.1g000014m                 N--------ESTFQPNP---------------------------N---S-VYQ-TE---------------HLSYFKFVGRVVGKALFDG---------QLLDVHFTRSFYKH-IL----
    orange1.1g001688m                 D---------HLLYPNP---------------------------G---S-GMIHEQ---------------HLQFFHFLGILLAKAMFEG---------ILVDIPFATFFLSK-LK----
    orange1.1g000012m                 N--------NASFQPNP---------------------------N---S-VYQ-TE---------------HLSYFKFVGRVVAKALFDG---------QLLDVHFTRSFYKH-ML----
    AT4G12570.1                       D-------DFRRFSPNP---------------------------A---S-KVD-PL---------------HPDFFEFTGRVIALALMHK---------VQVGVLFDRVFFLQ-LA----
    AT4G38600.1                       N-RDI-VLAPLGLFPRP-----------WPSTAD----------I---SEGGQFHK---------------VIEYFRLLGRVMAKALQDG---------RLLDVPLSTAFYKL-IL----
    AT1G55860.1                       N--------DATFQPNP---------------------------N---S-VYQ-TE---------------HLSYFKFVGRMVAKALFDG---------QLLDVYFTRSFYKH-IL----
    AT1G70320.1                       N--------DATFQPNP---------------------------N---S-VYQ-TE---------------HLSYFKFVGRMVAKALFDG---------QLLDVYFTRSFYKH-IL----
    AT3G53090.1                       D---------RLLVPSP---------------------------S---A-RHL-EN---------------GIQMIEFLGRIVGKALYEG---------ILLDYSFSHVFIQK-LL----
    AT3G17205.1                       D---------HMLYPNP---------------------------G---S-GMIHEQ---------------HLQFFHFLGSLLAKAMFEG---------ILVDIPFATFFLSK-LK----
    AT5G02880.1                       H-SGV-LASSSGLFPRP-----------WSGTS------------------TT-SD---------------VLQKFVLLGTVVAKALQDG---------RVLDLPLSKAFYKL-IL----
    Si034011m                         D---------TSLIPSN---------------------------S---A-RLL-DN---------------GIDMIEFLGRVVGKALYEG---------ILLDYTFSPVFVQK-LL----
    Si016079m                         G-RHL-IQAPLGLFPRP-----------WPPKAD----------S---SEGTRFFK---------------VLEYFRLIGQVMAKVLQDG---------RLLDLPLSTAFYKL-IL----
    Si013562m                         H-------DRRRFFINP---------------------------T---S-VVD-PL---------------HLQYFKFAGRMIALALRHK---------IHVGVLFDRTLFLQ-LA----
    Si013264m                         H-------DRRRFFINP---------------------------T---S-VVD-PL---------------HLEYFEFAGRMIALALRHK---------IHVGVYFDRTLFLQ-LA----
    Si009242m                         D---------HLLYPNP---------------------------A---S-GLVHEL---------------HLQYFHFLGSLLGKAMYEG---------ILVDLPFATFFLSK-LK----
    Si009164m                         S-RNV-VQAHLGLFPQP-----------WPPSAA----------A---LEGSKFFK---------------VVEYFRLVGRVMAKALQDG---------RLLDLPLSTAFYKL-LL----
    Si024055m                         E-DGF-IHASFGLFPKP-----------WSSSS---------------MQGIDFSN---------------VLQKFKLLGHLVVRAVLDG---------RILDIPLSKAFYKI-VL----
    Si020966m                         V-SRF-VVAPKGLFPRP-----------WSTSAD----------C---A---SFQE---------------VSKQFHLLGQVVAKAIKDG---------RILDIPFSKAFYKL-IL----
    Si020939m                         N--------DLTFQPNP---------------------------N---S-VYQ-TE---------------HLSYFKFVGRVVGKALFDG---------QLLDAHFTRSFYKH-IL----
    Si028891m                         E-------DKRRFYLNE---------------------------T---S-AVD-PL---------------HLKYFTFAGRIIGLALMHK---------VQVGIVLDRTLFLH-LA----
    Si028637m                         N--------NATFQPNP---------------------------N---S-VYQ-TE---------------HLSYFKFVGRVVAKALFDG---------QLLDVHFTRSFYKH-IL----
    Thhalv10019984m                   D---------HMLYPNP---------------------------G---S-GMIHEQ---------------HLQFFHFLGTLLAKAMFEG---------ILVDIPFATFFLSK-LK----
    Thhalv10011172m                   N--------DATFQPNP---------------------------N---S-VYQ-TE---------------HLSYFKFVGRMVAKALFDG---------QLLDVYFTRSFYKH-IL----
    Thhalv10011171m                   N--------DATFQPNP---------------------------N---S-VYQ-TE---------------HLSYFKFVGRMVAKALFDG---------QLLDVYFTRSFYKH-IL----
    Thhalv10024192m                   D-GDI-VQAPLGLFPRP-----------WPSTAD----------V---SEGSRFHK---------------VIEYFRLLGRVMAKALQDG---------RLLDVPLSTAFYKL-IL----
    Thhalv10028412m                   D-------DFRRFSPNP---------------------------A---S-QVD-PL---------------HPDFFEFTGRVIALALMHK---------VQVGVLFDRVFFLL-LA----
    Thhalv10012430m                   D-PGA-LGYASGLFPRP-----------WSGTS------------------AAFPG---------------VLQKFVLLGTVVAKALQDG---------RVLDLPFSKAFYKL-IL----
    Thhalv10010078m                   D---------RLLVPSP---------------------------S---A-RHL-EN---------------GIQMIEFLGRIVGKALYEG---------ILLDYSFSHVFIQK-LL----
    Ciclev10000001m                   N--------ESTFQPNP---------------------------N---S-VYQ-TE---------------HLSYFKFVGRVVGKALFDG---------QLLDVHFTRSFYKH-IL----
    Ciclev10004231m                   D---------HLLYPNP---------------------------G---S-GMIHEQ---------------HLQFFHFLGILLAKAMFEG---------ILVDIPFATFFLSK-LK----
    Ciclev10007219m                   N--------NASFQPNP---------------------------N---S-VYQ-TE---------------HLSYFKFVGRVVAKALFDG---------QLLDVHFTRSFYKH-ML----
    Ciclev10010897m                   N-SDI-VMSPFGLFPRP-----------WSSAVD----------T---SYGIQFSD---------------VLKKFVLLGQVVAKALQDG---------RVLDLPFSKAFYKL-IL----
    Ciclev10010940m                   D---------RLLIPNA---------------------------A---A-RYL-EN---------------GIQMFEFLGRVVGKALYEG---------ILLDYAFSHVFVQK-LL----
    Ciclev10027670m                   S-GDL-VHAPLGLFPRP-----------WPPSAD----------A---SEGGQFSK---------------VIEYFRLLGRVMAKALQDG---------RLLDLPFSTAFYKL-VL----
    Ciclev10014213m                   N-------DRRRFYPNH---------------------------A---S-KVH-PL---------------HLDYFCFSGRVIALALMHR---------VQVGVVFDRVFYLQ-LA----
    GRMZM2G034622_T02                 D---------HLLYPNP---------------------------G---S-GLVHEL---------------HLQYFHFLGSLLGKAMYEG---------ILVDLPFATFFLSK-LK----
    GRMZM2G124297_T01                 E-HGF-VHAPFGLFPKP-----------WPPS----------------SQGIDFTN---------------MLQKFKLLGNLVVRAVLDG---------RILDIPLSKAFYKI-ML----
    GRMZM2G411536_T03                 N--------DLTFQPNP---------------------------N---S-VYQ-TE---------------HLSYFKFAGRVVGKALFDG---------QLLDAHFTRSFYKH-IL----
    GRMZM2G181378_T01                 E-------DKRRFYLNE---------------------------T---S-AVD-PL---------------HLKYFTFAGRIIGLALMHK---------VQVGVVLDRTLFLH-LA----
    GRMZM2G049141_T01                 S-RNI-VQSPLGLFPQP-----------WPPTAA----------A---SEGSKFFK---------------VVEYFRLVGRVMAKALQDG---------RLLDLPLSTAFYKL-LL----
    GRMZM2G080439_T01                 E-------DKRRFYLNE---------------------------T---S-AVD-PL---------------HLKYFTFAGRIIGLALMHK---------VQVGVVIDRTLFLH-LA----
    GRMZM2G021299_T01                 N--------NATFQPNP---------------------------N---S-DFQ-TE---------------HLSYFKFVGRVVAKALFDG---------QLLDVHFTRSFYKH-IL----
    GRMZM2G328988_T01                 V-SRF-VVASNGLFPRP-----------WSASED----------P---A---SFQE---------------VSERFHLLGLVVAKAIKDN---------RILDIPFSKAFYKL-IL----
    GRMZM2G331368_T02                 N--------DLTFQPNP---------------------------N---S-VYQ-TE---------------HLSYFKFVGRVVGKALFDG---------QLLDAHFTRSFYKH-IL----
    GRMZM2G461948_T01                 D---------TSLIPSN---------------------------S---A-KLL-DN---------------GIDMIEFLGRVVGKALYEG---------ILLDYSFSPVFVQK-LL----
    GRMZM2G374574_T01                 G-MYL-IQAPLGLFPRP-----------WPPKVD----------T---SEGSRFFK---------------VLEYFRLIGQVIAKVLQDG---------RLLDLPLSTAFYKL-IL----
    Carubv10016604m                   D---------RLLVPSP---------------------------S---A-RHL-EN---------------GVQMIEFLGRIVGKALYEG---------ILLDYSFSHVFIQK-LL----
    Carubv10011657m                   N--------DATFQPNP---------------------------N---S-VYQ-TE---------------HLSYFKFVGRMVAKALFDG---------QLLDVYFTRSFYKH-IL----
    Carubv10007210m                   D-------DDRRFTPDT---------------------------A---S-YHD-EN---------------LLKYFQFAGRFIALALKND---------VQVGVLLDHVFYLQ-LA----
    Carubv10003974m                   D-RDI-VQAPLGLFPRP-----------WPSTAD----------V---SEGGQFHK---------------VTEYFRLLGRVMAKALQDG---------RLLDVPLSTAFYKL-IL----
    Carubv10012881m                   D---------HMLYPNP---------------------------G---S-GMIHEQ---------------HLQFFHFLGSLLAKAMFEG---------ILVDIPFATFFLSK-LK----
    Carubv10000054m                   H-SGV-LLSPSGLFPRP-----------WSGTS------------------TTSSD---------------MLQKFVLLGTVVAKALQDG---------RVLDLPFSKAFYKL-IL----
    Carubv10000186m                   D-------DVRRFSPNP---------------------------A---S-KVD-PL---------------HPDFFEFTGRVIALALMHK---------VQVGVMFDRVFFLQ-LA----
    Carubv10025730m                   N--------DSTFQPNP---------------------------N---S-VYQ-TE---------------HLSYFKFVGRVVGKALFDG---------QLLDVHFTRSFYKH-IL----
    Bradi2g34820.1                    D---------HLLYPNP---------------------------G---S-GLVHEH---------------HLQYFRFLGSLLGKAMYEG---------ILVDLPFATFFLSK-LK----
    Bradi2g37870.1                    E-TVF-LHATFGLFPQP-----------WSSVSS----------S---SRGIELSD---------------VVKKFKLLGHLVARAVLDG---------RILDIPLSKAFYKI-ML----
    Bradi2g22927.2                    V-DPITVAAPNGLFPRP-----------WSPSVD----------C---A---SFLE---------------VNKRFHLLGQVVAKAIKDG---------RILDIPFSRAFYKL-ML----
    Bradi4g07997.2                    N--------DLTFQPNP---------------------------N---S-VYQ-TE---------------HLSYFKFVGRVVGKALFDA---------QLLDVHFTRSFYKH-IL----
    Bradi4g33520.1                    N-------DQRRFYLNG---------------------------T---S-VVD-PL---------------HLKYFIFSGRIIGLAVMHK---------VQVGIVLDRTLFLH-LA----
    Bradi1g12340.2                    D---------SSLIPSN---------------------------S---A-KLL-DN---------------GIDMIEFLGRIVGKALYEG---------ILLEYCFSQVFVQK-LL----
    Bradi5g04567.1                    R-RNL-IQAPLGLFPRP-----------WPSTTV----------S---SEGSKFFK---------------VVEYFRLAGRVMAKALQDG---------RLMDLPLSTAFYKL-LL----
    Bradi3g00350.1                    R-RHL-IQAPLGLFPRP-----------WPSDVD----------A---SEGSRFFK---------------VIEYFRLIGRVMAKVLQDG---------RLLDLPLSTAFYKL-IL----
    Aquca_017_00766.1                 S-SGF-VVAPFGLFPRP-----------WSASS-----------A---PNETQLSE---------------VVKKFVLLGQLVAKALQDG---------RVLDLPLSCSLYKL-IL----
    Aquca_006_00259.1                 D-RDV-VLALLGLFPRP-----------YPPNAD----------V---SDGSQISK---------------VIEYFRLVGRVMAKALQDG---------RLLDLPLSTPFYKL-VL----
    Aquca_028_00189.1                 N-------DQRRFFPNP---------------------------V---S-KVD-SL---------------HLDYFGFCGRMIALALMNR---------VHVGIVFDRVFFLQ-LA----
    Aquca_027_00123.1                 D-PGI-VLAPFGLFPRP-----------LSSLSK----------S---FNELRLVE---------------VIKKFVLLGQIVAKALQDG---------RVLDLLFSKAFYKL-VL----
    Aquca_007_00539.1                 N--------DSTFQPNP---------------------------N---S-VYQ-TE---------------HLSYFKFVGRVVGKALFDG---------QLLDVHFTRSFYKH-IL----
    Aquca_003_00437.1                 D---------HLLYPNP---------------------------G---S-GLVHEQ---------------HLKFFHFLGIVLGKAMLEG---------ILVDIPFATFFLSK-LK----
    Aquca_019_00105.1                 E---------RHLVPNI---------------------------S---A-RLI-EN---------------GIPMIEFLGRIVGKALYEG---------ILLDYSFSHVFVQK-LL----
    MDP0000264736                     N-------DCRRFYPNP---------------------------A---S-KVD-PL---------------HLEYFTFAGRVIALALMHK---------VQVGIVFDRVFFQQ-LA----
    MDP0000320720                     N-TGI-LICPFGLFPRP-----------WLATSD---------------------E---------------VXKKFVLLGQIVGRALQDG---------RVLDVHFSKAFYKL-IL----
    MDP0000142676                     N-------DRRRFYPNPVCDHMCMLHFVRSLVSDAGMCRNIGHQA---S-KVD-PL---------------HLEYFTFAGRVIALALMHK---------VHVGIVFDRVFFQQ-LA----
    MDP0000318443                     N--------ESTFQPNP---------------------------N---S-VYQ-TE---------------HLSYFKFVGRVVGKALFDG---------QLLDVHFTRSFYKH-IL----
    MDP0000206447                     N--------NATFQPNP---------------------------N---S-VYQ-TE---------------HLSYFKFVGRVVAKALFDG---------QLLDVYFTRSFYKH-IL----
    MDP0000196216                     D---------GLLIPNM---------------------------S---A-RFL-EN---------------GIQMIEFLGRVVGKALYEG---------ILLDYSFSHVFVQK-LL----
    MDP0000186793                     D---------HLLYPSP---------------------------G---S-GMIHEQ---------------HLRFFHFLGILLAKAMFEG---------ILVDIPFATFFLSK-LK----
    MDP0000822588                     D-SGI-LICPFGLFPCP-----------WLGTSD----------E---MQ-IQFSE---------------VIKKFVLLGQIVGKALQDG---------RVLDVHFSKAFYKL-LL----
    MDP0000924418                     D---------RLLMPNA---------------------------S---A-RFL-EN---------------GIQMIEFLGRVVGKALYEG---------ILLDYSFSHVFVQK-LL----
    MDP0000320505                     --GDI-VQAPLGLFPRP-----------WPPNAV----------A---SDGSQFSK---------------VIEYFRLVGRVMAKALQDG---------RLLDLPLSTAFYKL-LL----
    MDP0000307848                     N--------NATFQPNP---------------------------N---S-VYQ-TE---------------HLSYFKFVGRVVAKALFDG---------QLLDVYFTRSFYKH-IL----
    MDP0000301275                     --GDI-VQAPLGLFPRP-----------WPPNAV----------A---SDGSQFSK---------------VIEYFRLVGRVMAKALQDG---------RLLDLPLSTAFYKL-LL----
    MDP0000317971                     N--------ESTFQPNP---------------------------N---S-VYQ-TE---------------HLSYFKFVGRVVGKALFDG---------QLLDVHFTRSFYKH-IL----
    Bra022201                         D---------HMLYPNP---------------------------G---S-GMVHDQ---------------HLQFFHFLGTLLAKAMFEG---------ILVDIPFATFFLSK-LK----
    Bra028860                         ------MVPPSGLFPRP-----------WSATS------------------AAFPG---------------VLQKFVLLGTVVAKALQDG---------RVLDIPFSKTFYKL-IL----
    Bra038022                         N--------DATFQPNP---------------------------N---S-VYQ-NE---------------HLSYFKFVGRMVAKALFDG---------QLLDVYFTRSFYKH-IL----
    Bra021231                         D---------HMLYPNP---------------------------G---S-GMIHGQ---------------HLQFFHFLGSLLAKAMFEG---------ILVDLPFATFFLSK-LK----
    Bra005748                         D-SGF-LVAPSGLFPRP-----------WSDTS------------------AAFPD---------------VLQKFVLLGTVVAKALHDG---------RVLDIPFSKAFYKL-II----
    Bra000779                         D-------DFRRFSPNP---------------------------A---S-KVD-PL---------------HLEYFKFAGRVIALALMHK---------VQVGVLFDRVFYLQ-LT----
    Bra029461                         D-------DFRRFSPNP---------------------------A---S-KVD-PL---------------HLSYFEFTGRVIALALMHK---------VQVGVLFDRVFFSQ-LA----
    Bra027850                         N--------DSTFQPNP---------------------------N---S-VYQ-TE---------------HLSYFKFVGRVVGKALFDG---------QLLDVHFTRSFYKH-IL----
    Bra040685                         D---------RLLVPSP---------------------------S---A-RYL-EN---------------GIQMIEFLGRIVGKALYEG---------ILLDYSFSHVFIQK-LL----
    Bra010737                         D-RDI-VQAPLGLFPRP-----------WPSTAD----------V---SEGSRFHK---------------VVEYFRLLGRVMAKALQDG---------RLMDVPLSTAFYKL-IL----
    Medtr2g025830.1                   N-------DRRRFFPNA---------------------------A---S-KVN-PL---------------HLKYFSFSGRMIALALKNK---------VHVGIVFDRVFFKQ-LA----
    Medtr2g025950.1                   N-------DHRRFFPNT---------------------------A---S-MVN-AL---------------HLRYFIFSGRIIALALKKK---------VHVGIVFACVFFKQ-LA----
    Medtr2g025810.1                   N-------DHRRFFPNT---------------------------A---S-MVN-AL---------------HLKYFIFSGRIIALALKKK---------VRVGIVFDRVFFKQ-LA----
    Medtr2g033040.1                   D---------HLLYPNP---------------------------G---S-GMIHEQ---------------HLQFFHFLGTLLAKAMFEG---------ILVDLPFATFFLSK-LK----
    Medtr2g025790.1                   N-------DHRRFFPNA---------------------------A---S-KVN-SL---------------HLKYFIVSGRIIALALKKK---------VHVGIVFDRVFFKQ-LA----
    Medtr2g025930.1                   N-------DRRRFFPNA---------------------------A---S-KVN-PL---------------HLKYFSFSGRMIALALKNK---------VHVGIVFDRVFFKQ-LA----
    Medtr7g100670.1                   ----------YGLFPRP-----------WLKMQD----------E---SDGLKISE---------------VQKKFVLLGQVVAKAIQDG---------RHLDLHISKAFHKL-IC----
    Medtr5g066710.1                   N--------ESTFQPNP---------------------------N---S-VYQ-TE---------------HLSYFKFIGRVVGKALFDG---------QLLDVHFTRSFYKH-IL----
    Medtr4g073370.1                   D-GEL-VQAPLGLFPRP-----------WPANAD----------A---SEGSQLFK---------------VIEYFRLLGRVVAKALQDG---------RLLDLPLSVAFYKL-VL----
    Medtr4g133120.1                   K-------DRTRFLPNS---------------------------A---S-KVQHNL---------------HLEYFSFCGRVIALALMHK---------VQVGIVFDRVFFLQ-LA----
    Vocar20002255m                    E--------SRTFWFNA---------------------------A---A-CLE-DG---------------AAGEFRLVGAVLGLAIYNG---------IILDVHVPQAVYKK-LL----
    Vocar20010178m                    D-------GGSTFQPNP---------------------------N---S-HVQ-ND--RG---------ISHLDYFRFVGRVVGKALYDG---------QLIDAYFTRSFYKH-LL----
    Vocar20006334m                    ---------AGAYWYNT---------------------------T--LT---ESEE---------------LRDAYNFAGWLLGQSLLNR---------APLGLPLPAVLFRA-VLE---
    Vocar20007555m                    P-------QRNQLYVTP---------------------------T---S-SS--PA---------------HLKKFAFVGLFMAKAILESAARGKELGPITLNLPLCEPFWKL-LL----
    Vocar20012583m                    E-CEY-VNAPWGLFPRP-----------LPPAAR----------S-----SPAGLK---------------AVERFRLLGRTLAKALQDN---------RLLDLPLSHVFYAA-AL----
    Vocar20003001m                    S---------HQLYPNP---------------------------A---A-VRVVED---------------APRLLAFLGRMLGKAMYEN---------ILLELPLAGFFLKK-FR----
    Vocar20004069m                    T--------SRTYWFNP---------------------------A---S-LEP--P-----------------DSYFLLGLVLGLAVYNR---------VLLAFPAPLLLYQK-LR----
    Vocar20000780m                    D---------GLIYPNP---------------------------A---A-ERL-DG---------------GLALLEFMGLMFGKALYEG---------ILLPVPLAHFFISR-LQLLTA
    Vocar20004842m                    T--------SHLHWFRP---------------------------S---R-LEM--E-----------------LEFELVGILIGLAIYNS---------HILEFQFPSVLYKK-LM----
    Vocar20014908m                    D--------RRVVHPSP---------------------------H---S-RMQ-ED---------------HLGYMRFAGRIVGLALRAN---------VPLGVVLSTGLFNY-LT----
    Lus10032589                       N--------NATFQPNS---------------------------N---S-VYQ-TE---------------HLSYFRFVGRVVAKALFDG---------QLLDVYFTRSFYKH-IL----
    Lus10035589                       D---------RLLVPNP---------------------------A---A-RYL-EN---------------GIQMIEFLGRVVGKALYEG---------ILLDYSFAHVFVQK-LL----
    Lus10005068                       N-DNL-VLAPLGLFPRP-----------WPPTAD----------A---AEGSQLLK---------------VVEYFRLVGRIMAKALQDG---------RLLDLPLSTAFYKI-VL----
    Lus10010493                       S-------DRRRFHPNP---------------------------A---S-KVE-PR---------------HLEYFAFSGRVIALALMHK---------VQVGIVLDRVLFLQ-LA----
    Lus10027841                       N-ANL-VLAPLGLFPRP-----------WPPTAD----------A---AEGSQLLK---------------VVEYFRLVGRIMAKALQDG---------RLLDLPLSTAFYKI-VL----
    Lus10019908                       -------VSPIGLFPCP-----------IPPMMD----------A---ANGIQFSE---------------ISKKFVLLGQVVAKALQDG---------RVLDLPFSKAFYKL-IL----
    Lus10032830                       N--------DSTFQPNP---------------------------N---S-VYQ-TE---------------HLSYFKFVGRVVGKALFDG---------QLLDVHFTRSFYKH-IL----
    Lus10017098                       ------------------------------------------------------------------------------------KAMFEG---------ILVDIPFATFFLSK-LK----
    Lus10002605                       N--------DSTFQPNP---------------------------N---S-VYQ-TE---------------HLSYFNFVGRVVGKALFDG---------QLLDVHFTRSFYKH-IL----
    Lus10008636                       D---------RLLVPNP---------------------------A---A-RYL-EN---------------GTQMIEFLGRVVGKALYEG---------ILLDYSFAHVFVQK-LL----
    Eucgr.A01178.1                    G-SEM-ILSPFGLFPCP-----------WAPALC----------T---SENVEFAE---------------VINRFILLGKLVAKALQDG---------RVLDLHLSKAFYKL-II----
    Eucgr.A01586.1                    D---------RLLMPNI---------------------------S---A-KYL-EY---------------GLQMIEFLGRIVGKALYEG---------ILLDYSFSHVFVQK-LL----
    Eucgr.B03986.1                    D---------HLLYPNP---------------------------G---S-GLIHEQ---------------HLQFFHFLGTLLAKAMFEG---------ILVDIPFATFFLSK-LK----
    Eucgr.D01414.1                    N-------DRRRFYPNP---------------------------A---S-TVE-PL---------------HLKYFNFSGRMIGLALMHK---------VQVGIVLDRVFFLQ-LG----
    Eucgr.D01416.1                    N-------DRRRFYPNP---------------------------A---S-TVE-PL---------------HLKYFNFSGRMIGLALMHK---------VQVGIVLDRVFFLQ-LG----
    Eucgr.F02160.1                    N--------ESTFQPNP---------------------------N---S-VYQ-TE---------------HLSYFKFIGRVVGKALFDG---------QLLDVHFTRSFYKH-IL----
    Eucgr.I01410.2                    D-RDT-VVAPLGLFPRP-----------WPPNAD----------V---SDGSKFSK---------------VIEYFRLVGRVMAKALQDG---------RLLDLPMSSAFYKL-VL----
    Pavirv00038038m                   N--------DLTFQPNP---------------------------N---S-VYQ-TE---------------HLSYFKFVGRVVGKALFDG---------QLLDAHFTRSFYKH-IL----
    Pavirv00031244m                   G-RHL-IQAPLGLFPRP-----------WPPKAD----------A---SEGSRFFK---------------VLEYFRLIGQVTAKVLQDG---------RLLDLPLSTAFYKL-IL----
    Pavirv00010575m                   E-------DKRRFYLNE---------------------------T-----------------------------------------LMHK---------VQVGIVLDRTLFLH-LA----
    Pavirv00004902m                   Q-------DRRRFFINP---------------------------T---S-VVD-PL---------------HLEYFEFAGRMIALALRHK---------IYAGVFFDRTLFLQ-LA----
    Pavirv00020428m                   E-DRF-IHASFGLFPKP-----------WSPSG---------------TQGIEFSN---------------VLQKFKLLGHLVVRAVLDG---------RILDIPLSKAFYKI-ML----
    Pavirv00067430m                   E-------DKRRFYLNE---------------------------T---S-AVD-PL---------------HLKYFTFAGRIIGLALMHK---------VQVGIVLDRTLFLH-LA----
    Pavirv00058663m                   N--------NATFQPNP---------------------------N---S-VYQ-TE---------------HLSYFKFVGRVVAKALFDG---------QLLDVHFTRSFYKH-IL----
    Pavirv00067620m                   G-RHL-IQAPLGLFPRP-----------WPPKAD----------A---SEGSRFFK---------------VLEYFRLIGQVTAKVLQDG---------RLLDLPLSTAFYKL-IL----
    Pavirv00029557m                   N--------DLTFQPNP---------------------------N---S-VYQ-TE---------------HLSYFKFVGRVVGKALFDG---------QLLDAHFTRSFYKH-IL----
    Pavirv00023469m                   D---------HLLYPNP---------------------------A---S-GLVHEL---------------HLQYFHFLGSLLGKAMYEG---------ILVDLPFATFFLSK-LK----
    Pavirv00024250m                   V-SRF-VVAPKGLFPRP-----------WSASAD----------C---A---SFQE---------------VSKQFHLLGQVVAKAIKDG---------RILDIPFSKAFYKL-IL----
    Pavirv00023205m                   I-SRF-VVAPNGLFPRP-----------WSASAD----------C---A---SFQV---------------VSKHFHLLGQVVAKAIKDG---------RILDIPFSKAFYKL-TL----
    Pavirv00029138m                   E-DGF-IHASFRLFPKP-----------WSSSG---------------TRGVDFSN---------------VLQNFKLLGHLVVRAVLDG---------RILDIPLSKAFYKI-ML----
    LOC_Os03g47949.1                  D---------SSLIPSN---------------------------S---A-KLL-DN---------------GIDMIEFLGRVVGKALYEG---------ILLDYCFSPVFVQK-LL----
    LOC_Os02g01170.1                  G-SHL-IQAPFGLFPRP-----------WPLTVD----------A---SEGSRFSK---------------VIEHFRLVGRVMAKVLQDG---------RLLDLPLSTALYKL-IL----
    LOC_Os09g07900.1                  N--------NATFQPNP---------------------------N---S-VYQ-TE---------------HLSYFKFVGRVVAKALFDG---------QLLDVHFTRSFYKH-IL----
    LOC_Os12g24080.1                  N--------DLTFQPNP---------------------------N---S-VYQ-TE---------------HLSYFKFVGRVVGKALFDG---------QLLDVHFTRSFYKH-IL----
    LOC_Os05g38830.1                  G-SVF-VVAPNGLFPKP-----------WSTHVD----------C---S---SFSE---------------VNKQFHLLGQVVAKAVKDN---------RILDIPFSKAFYRL-IL----
    LOC_Os05g03100.1                  E-AGF-VHAPFGLFPQP-----------WSSANT----------S---SQGISLSN---------------VVQKFKLLGHLVARAVLDG---------RVLDIPLSKAFYKI-ML----
    LOC_Os05g06690.1                  D---------HLLYPNP---------------------------G---S-GLVHEQ---------------HLQYFHFLGSLLGKAIYEG---------ILVDLPFATFFLSK-LK----
    PGSC0003DMT400075387              N-------DRRRFFPNS---------------------------A---S-KVD-PL---------------HLEYFTFCGRMIALALMHK---------IQIGVVFDRLFFLQ-LA----
    PGSC0003DMT400021802              N-------DRRRFFPNP---------------------------A---S-KVD-PL---------------HLEYFSFSGRVIALALMHK---------IQVGIVFDRVFFLQ-LS----
    PGSC0003DMT400031190              D-RDL-VQAPLGLFPRP-----------WSPHTG----------T---VDGGQFCK---------------AIEYFRLLGRVMAKSLQDG---------RLLDLPLSMAFYKL-VL----
    PGSC0003DMT400072624              N-------DRRRFFPNS---------------------------A---S-KVN-PL---------------HLNYFCFSGRMIALTLMHK---------VQIGVVFDRTFFLQ-LA----
    Glyma14g36180.1                   N--------ESTFQPNP---------------------------N---S-VYQ-TE---------------HLSYFKFVGRVVGKALFDG---------QLLDVHFTRSFYKH-IL----
    Glyma02g38020.2                   N--------ESTFQPNP---------------------------N---S-VYQ-TE---------------HLSYFKFVGRVVGKALFDG---------QLLDVHFTRSFYKH-IL----
    Glyma12g03640.1                   D-GEL-VQAPLGLFPRP-----------WPANAD----------A---SEGTQIFK---------------VIEYFRLLGRVMAKALQDG---------RLLDLPLSVAFYKL-VL----
    Glyma11g11490.1                   D-GEL-VQAPLGLFPRP-----------WSANAD----------A---SEGTQFFK---------------VIEYFRLLGRVMAKALQDG---------RLLDLPMSVAFYKL-VL----
    Glyma06g00600.1                   D-GEL-VEAPLGLFPRP-----------WPTNSD----------A---SEGSRFSK---------------VVEYFRLLGRVMAKALQDG---------RLLDLPLSVAFYKL-VL----
    Glyma06g10360.1                   N--------ESTFQPNP---------------------------N---S-VYQ-TE---------------HLSYFKFVGRVVGKALFDG---------QLLDVHFTRSFYKH-VL----
    Glyma04g00530.1                   D-GEL-VQAPLGLFPRP-----------WPTNSD----------A---SESSQFSK---------------VIEYFRLLGRVMAKALQDG---------RLLDLPLSVAFYKL-VL----
    Glyma04g10481.1                   N--------ESTFQPNP---------------------------N---S-VYQ-TE---------------HLSYFKFVGRVVGKALFDG---------QLLDVHFTRSFYKH-IL----
    Glyma08g09270.3                   N--------NATFQPNP---------------------------N---S-VYQ-TE---------------HLSYFKFVGRVVGKALFDG---------QLLDVYFTRSFYKH-IL----
    Glyma17g01210.2                   N-------DQRRFFPNP---------------------------A---S-KVH-PL---------------HLEYFSFAGRVIALALMHR---------VQVGIVFDRVFFLQ-LA----
    Glyma17g04180.1                   D---------HLLYPNP---------------------------G---S-GMIHEQ---------------HFQFFHFLGTLLAKAMFEG---------ILVDIPFATFFLSK-LK----
    Glyma13g19981.1                   D-IG--THSFYGLFPRP-----------WSSMQD----------T---SGGIQFSE---------------VIKNFFLLGQVVAKALQDG---------RILDLHFSKAFYKL-IL----
    Glyma05g26360.1                   N--------NATFQPNP---------------------------N---S-VYQ-TE---------------HLSYFKFVGRVVGKALFDG---------QLLDVYFTRSFYKH-IL----
    Glyma19g37310.1                   D---------RLLIPTA---------------------------S---A-RYL-EN---------------GLQMIEFLGRVVGKALYEG---------ILLDYSFSHVFVQK-LL----
    Glyma15g14591.1                   D---------YLLYPNP---------------------------G---S-GMIHEQ---------------HLQFFHFLGTLLAKAMFEG---------ILVDLPFATFFLSK-LK----
    Glyma03g34650.2                   D---------RLLIPTA---------------------------S---A-RYL-EN---------------GLQMIEFLGRVVGKALYEG---------ILLDYSFSHVFVQK-LL----
    Glyma10g05620.3                   E-IG--VHSFYGLFPRP-----------WSSMQD----------T---SGGIQFSE---------------VTKNFFLLGQVVAKALQDG---------RILDLHFSKAFYKL-IL----
    Glyma07g36390.1                   D---------HLLYANP---------------------------G---S-GMIHEQ---------------HFQFFHFLGTLLAKAMFEG---------ILVDIPFATFFLSK-LK----
    Glyma07g39546.1                   N-------DRRRFFPNP---------------------------A---S-KVH-PL---------------HLEYFSFAGRVIALALMHR---------VQVGIVFDRVFFLQ-LA----
    Gorai.010G033100.1                N--------ESTFQPNP---------------------------N---S-VYQ-TE---------------HLSYFKFVGRVVGKALFDG---------QLLDVHFTRSFYKH-IL----
    Gorai.010G186800.1                N-------DRRRFFPNP---------------------------A---S-RVD-PL---------------HLEYFRFAGRVIALALMHK---------VQVGVVFDRVFFQQ-LA----
    Gorai.009G278900.1                N--------ESTFQPNP---------------------------N---S-VYQ-TE---------------HLSYFKFVGRVVGKALFDG---------QLLDVHFTRSFYKH-IL----
    Gorai.009G228200.1                N--------ESTFQPNP---------------------------N---S-VYQ-TE---------------HLSYFKFVGRVVGKALFDG---------QLLDVHFTRSFYKH-IL----
    Gorai.009G183200.1                Y-------DRRRFFPNP---------------------------A---S-RMD-PL---------------HLEYFSFAGRVIALALMHK---------VQVGIVFDRVFFLQ-LA----
    Gorai.009G420400.1                E-SVI-LRNSSGLFPRP-----------CSPKSD----------A---NNGIQFSQ---------------VLKKFVLLGQIVAKAIQDG---------RVLDVSFSKAFYKL-IL----
    Gorai.002G100900.1                S--------NATFQPNS---------------------------N---S-VYQ-TE---------------HLSYFKFVGRVVAKALFDG---------QLLDVYFTRSFYKH-IL----
    Gorai.002G196900.1                G-GDI-VQAPLGLFPRP-----------WPPNAD----------A---SEGSQFFK---------------VIEYFRLLGRVMAKALQDG---------RLLDLPLSTPFYKL-VL----
    Gorai.002G245000.1                D---------RLLIPNA---------------------------A---A-RFL-EN---------------GIQMIEFLGRVVGKALYEG---------ILLDYSFSHVFVQK-LL----
    Gorai.002G003200.1                D-GVM-IQAPLGLFPRP-----------WQLNAD----------A---SEGSEFSK---------------VIEYFRLVGRVMAKALQDG---------RLLDLPLSTSFYKL-VL----
    Gorai.011G204200.1                D---------HLLYPNP---------------------------G---S-GMIHEQ---------------HLQFFHFLGTLLAKAMFEG---------ILVDIPFATFLLSK-LK----
    Gorai.008G035900.1                N-GDI-VQAPLGLFPRP-----------WPPNAD----------A---SEGSQFFK---------------VIEHFRLVGRVMAKALQDG---------RLLDLPLSMAFYKL-VL----
    Gorai.006G265700.1                D---------HLLYPNP---------------------------G---S-GMIHEQ---------------HLQFFHFLGSLLAKAMFEG---------ILVDIPFATFFLSK-LK----
    Potri.010G150000.3                D---------HLLYPNP---------------------------G---S-GMTHEQ---------------HLQFFHFLGTLLAKAMFEG---------ILVDIPFATFFLSK-LK----
    Potri.009G134300.1                A-ADL-VQTPLGLFPRP-----------WPPTAS----------A---SEGSQIYK---------------TIEYFRLVGRVMAKALQDG---------RLLDLPLSMAFYKL-VL----
    Potri.004G174700.1                A-ADL-VQAPLGLFPRP-----------WPPTTS----------A---SEGSQFYK---------------TIEYFRLVGRVMAKALQDG---------RLLDLPLSMAFYKL-VL----
    Potri.011G094100.1                N--------DVTFQPNP---------------------------N---S-VYQ-TE---------------HLSYFKFVGRVVSKALFDG---------QLLDVYFTRSFYKH-IL----
    Potri.006G132000.1                Y-SGI-VNSSFGLFPRP-----------WPSSVD----------A---SDAAQFSE---------------VIKKFFLLGQIVAKALQDG---------RVLDLPFSKAFYKL-IL----
    Potri.006G011700.1                S-------DRRRFYPNP---------------------------A---S-KVD-PM---------------HLDYFTFSGRVIALALMHK---------VQVGIVFDRVFFLQ-LA----
    Potri.016G085200.3                Y-SGI-VKSPFGLFPRP-----------WSPTVD----------A---SDGVQFSE---------------VIKKFFLLGQIVAKALQDG---------RVLDLPFAKVFYKL-IL----
    Potri.016G096500.1                E---------RHLIPNP---------------------------T---A-KYL-EN---------------GIQMIEFLGRVVGKALYEG---------ILLDYSFSHVFVQK-LL----
    Potri.016G012900.1                S-------DRRRFYPNP---------------------------A---S-KVD-PM---------------HLEYFTFSGRVMALALMHK---------VQVGIVFDRAFFLQ-LA----
    Potri.002G110500.1                N--------ESTFQPNP---------------------------N---S-VYQ-TE---------------HLSYFKFVGRVVGKALFDG---------QLLDVHFTRSFYKH-IL----
    Potri.008G101300.1                D---------HLLYPNP---------------------------G---S-GMLHEQ---------------HLQFFHFLGTLLAKAMFEG---------ILVDIPFATFFLSK-LK----
    Potri.001G368600.1                N--------NVTFQPNP---------------------------N---S-VYQ-TE---------------HLSYFKFVGRVVAKALFDG---------QLLDVYFTRSFYKH-IL----
    Phvul.003G084200.1                N-------DRRRFFPNP---------------------------A---S-KVH-PL---------------HLEYFSFAGRVIALALMHR---------VQVGIVFDRVFFLQ-LA----
    Phvul.003G118500.1                D---------HLLYPNP---------------------------G---S-GMIHEQ---------------HFQFFHFLGTLLAKAMFEG---------ILVDIPFATFFLSK-LK----
    Phvul.009G119700.1                D-EEL-VQAPMGLFPRP-----------WPTNSD----------A---SEGSPFAK---------------VIEYFRLLGRVMAKALQDG---------RLLDLPLSVAFYKL-VL----
    Phvul.009G034900.1                N--------ESTFQPNP---------------------------N---S-VYQ-TE---------------HLSYFKFVGRVVGKALFDG---------QLLDVHFTRSFYKH-IL----
    Phvul.011G035200.1                D-GEL-VHSPLGLFPRP-----------WPANAD----------A---SEGTQFSK---------------VIEYFRLLGRVMAKALQDG---------RLLDLPLSAAFYKL-VL----
    Phvul.008G183200.1                N--------ESTFQPNP---------------------------N---S-VYQ-TE---------------HLSYFKFVGRVVGKALFDG---------QLLDVHFTRSFYKH-IL----
    Phvul.007G163300.1                E-MR--IHSFYGLFPRP-----------WSTMQD----------T---SGDKQLSE---------------VTKRFFLLGQVVAKALQDG---------RILDLHFSKAFYKL-IL----
    Phvul.007G163400.1                E-TR--IRSFCGLFPRP-----------WSSMQD----------T---SGGIKFSE---------------VTKKFFLLGQVFAKALHDG---------RILDFHFSKVFYKL-IL----
    Phvul.001G184300.1                D---------RLLIPTA---------------------------S---A-RYL-EN---------------GLQMIEFLGRVVGKALYEG---------ILLDYSFSHVFVQK-LL----
    Phvul.006G120900.1                D---------HLLYPNP---------------------------G---S-GMIHEQ---------------HLQFFHFLGTLLAKAMFEG---------ILVDLPFATFFLSK-LK----
    Phvul.006G142800.1                N-------DGRRFYPNS---------------------------A---S-KVH-PR---------------HLEYFRFAGRIIALALLKK---------VQVGIVFDRVFFLQ-LA----
    Phvul.002G189700.1                N--------NATFQPNP---------------------------N---S-VYQ-TE---------------HLSYFKFVGRVVGKALFDG---------QLLDVYFTRSFYKH-IL----
    mrna26562.1-v1.0-hybrid           N--------ESTFQPNP---------------------------N---S-VYQ-TE---------------HLSYFKFVGRVVGKALFDG---------QLLDVHFTRSFYKH-IL----
    mrna05017.1-v1.0-hybrid           D---------HLLYPNP---------------------------G---S-GMIHDQ---------------HLQFFHFLGILLAKALFEG---------ILVDIPFATFFLSK-LK----
    mrna09579.1-v1.0-hybrid           D-TGI-LICPCGLFPRP-----------WSSTMD----------A---SDGTQFSE---------------VIKKFTLLGKIVGKALQDG---------RVLDLHFSKAFYKL-IL----
    mrna30084.1-v1.0-hybrid           A---------RLLIPNP---------------------------S---A-RYL-EN---------------GIQMIEFLGRVVGKALYEG---------ILLDYSFSHVFVHK-LL----
    mrna07649.1-v1.0-hybrid           --VDI-VLAPLGLFPRP-----------WPPNAV----------A---SDGNQFSK---------------VIEYFRLVGRAMAKALQDG---------RLLDLPLSTAFYKL-LL----
    mrna20590.1-v1.0-hybrid           N--------NATFQPNP---------------------------N---S-VYQ-TE---------------HLSYFKFVGRVVAKAVFDG---------QLLDVYFTRSFYKH-IL----
    mrna19775.1-v1.0-hybrid           N-------DRRRFYPNP---------------------------GVLPS-KVD-PL---------------HLEYFTFAGRVIALALMHK---------VQVGIVFDRIFFQQ-LA----
    Solyc04g076620.2.1                N--------EATFQPNP---------------------------N---S-VYQ-TE---------------HLSYFKFVGRVVGKALFDG---------QLLDVHFTRSFYKH-IL----
    Solyc10g083470.1.1                N-------DRRRFFPNS---------------------------A---S-KVK-PL---------------HLDYFWFSGRMIALTLTHK---------IQIGIVFDRTFYLQ-LA----
    Solyc10g055450.1.1                D-GDL-VQAPLGLFPRP-----------WSPHTG----------T---VDGGQFYK---------------AIEYFRLLGRVMAKALQDG---------RLLDLPLSMAFYKL-VL----
    Solyc07g065630.2.1                N--------NATFQPNP---------------------------N---S-VYQ-TE---------------HLSYFKFVGRVVAKALFDG---------QLLDVHFTRSFYKH-IL----
    Solyc05g054080.2.1                N-------DRRRFFPNP---------------------------A---S-KVD-PL---------------HLEYFSFSGRVIALALMHK---------IQVGIVFDRVFFLQ-LS----
    Solyc01g057900.2.1                D---------HLLYPNP---------------------------G---S-GLIHDQ---------------HLQYFHFLGTVLAKAMFEG---------ILVDIPFATFFLSK-LK----
    Solyc01g111530.2.1                D-KEL-VQAPLGLFPRP-----------WSSTVE----------T---ADDNHFPK---------------VIEYFRLLGRVMAKALQDG---------RLLDLPLSTAFYKL-LL----
    Solyc12g094560.1.1                N-------DRRRFFPNS---------------------------A---S-KVD-PM---------------HLEYFTFCGRMVALALMHK---------IQIGVVFDRVFFLQ-LA----
    Solyc09g005150.1.1                N-------DGRRFFPNP---------------------------V---S-KVD-PL---------------HLEYFVFSGRIIALALLHR---------VQVSITFDRVFFRQ-LA----
    Solyc09g007310.2.1                E-SGI-IFSPFGLFPRP-----------WSPSPH----------S---LNGLEFSE---------------VLKKFVLLGQIVAKSLQDG---------RVLDLRLSRAFYKL-LL----
    Solyc09g005160.1.1                N-------DGRRFFPNP---------------------------A---S-KVD-PL---------------HLEYFTFSGRMIALALLHR---------VQISITFDRVFFLQ-LA----
    Solyc09g008700.1.1                D---------RHLIPNT---------------------------A---A-RFL-DN---------------GIQMIEFLGRIVGKALYEG---------ILLDYSFSHVFVQK-LL----
    69212                             G--------GVTLHPNP---------------------------M---S-GEVTPE---------------HLSYFYALGRLAAVALYHG---------ETMPLRLTPAFCSR-LL----
    70217                             D---------GALYPCS---------------------------D---A-GDT-RE---------------GLEIVELMGTMTGKALYEG---------ILAEANLAHFFSKA-LL----
    48481                             D--------SGLFWFRA---------------------------G---A-DDA--P---------------SLSRCRLVGAAIGLAIYNG---------VTLDVHLPHVAYKR-LF----
    19835                             D---------RTLYPNP---------------------------A---SWKHAGAD---------------HLRKLEFLGAMLGKAVYEG---------ILVDLPLAGFFLAK-LR----
    213597                            G--------DQSYQPFS---------------------------N---S-SVN-DH---------------HLAYFKFIGRIVGKAVYDG---------HLMDAHFTRPFYKH-ML----
    174890                            D-IKY-VNAPHGLFPAP-----------VSSSAYDVA-------P---PGSPGKLT---------------RGDLFRLLGRVVGKALQDG---------RLLDLGLSPAFFRA-IT----
    154462                            D-------GLAIYHPPP---------------------------L---P-SPL-PR--------------RLEDEYTALGWLLGYAILHE---------SPLPVAFSSAFLKA-LA----
    22875                             E--------TRTFWFAPEA-------------------------D---D-LGL-ED-----------------VEFELIGIVIGLAIYNE---------HILDFRFPMTIYRK-LL----
    172918                            --PEH-IWHTNGLFPAP-----------APPGTP----------E---SEAAR--------------------KRFAFIGRLFGKALLDG---------HILPLPLNPAFLRAAIL----
    29762                             D---------QKLYPNP---------------------------A---A-ATVEPN---------------AFALFEFMGKMVGKALYEG---------ILLEVPLAGFFLKK-LL----
    67182                             P-DAY-VQAPQGLFPAP-----------MQQSKA----------A---G-----SK---------------VVERYRLLGRAMAKALQDS---------RLLDIPLSYTFYRA-AL----
    58691                             E--------TRTYWFNA---------------------------T---S-MEA--E-----------------TEFTLVGQLMGLAIYNS---------VILDAHFPHCLYLE-LL----
    16350                             E--------LRTFWFRA---------------------------S---S-LDL--A-----------------MEFELVGLLLALAIYNN---------HILEVSFPMVVYKM-LM----
    35876                             E--------SRTYWFNP---------------------------S---T-LEA--E-----------------DEFMLIGLVLGLAIYNG---------VLLDFPLPLALYRK-LL----
    15978                             E--------GGLAFPQP---------------------------A---A-AHI-AS---------------APALLHFLGLVFGKALYEG---------ILLDTPLAPFFVAR-LQ----
    37891                             T-------GGSTFQPNP---------------------------N---S-IVQ-NDEARG---------TNHLDFFKFVGRVVGKALYDG---------QLIDAYFTRSFYKH-ML----
    57759                             --PEH-LWQTRGLFPHP-----------LPPRSP----------E---SELAR--------------------RRFRFIGRLVGKALLDG---------HILPLPLNPAFVRVAIL----
    59359                             G--------DGAYQPFG---------------------------N---S-GIN-ET---------------HLAYFKFIGRIIGKAVYDG---------YLVDAHFTRPFYKH-ML----
    108435                            D---------RTLYPNP---------------------------A---SVRRCGPD---------------HLRKLEFLGAMLGKAVYEG---------ILVDLPLAGFFLAK-LR----
    87459                             E--------TNCCWFAK-------------------------------G-KQP--W-----------------AEYELAGVIVGLAVYNG---------HVLDLRLPKAAYKK-LT----
    60437                             E--------SRYHWFNPAA-------------------------SV--S-AES--L-----------------ARFRLFGAALGLAIYNG---------VCLDVHLPPVAYRR-LC----
    60965                             EENGQ-RNACVWYHVDP-------------------------------TCAV--GE---------------SPLTWRFVGRFIGLCITSG---------CHVRMPLVPWLWDQ-LLH---
    83330                             A-DGF-VNAPHGLFPAP-----------VSAKDFDAT-------V---PGAPGGIK---------------RGDLFRMLGRAVGKALQDG---------RLLDMALSPVFFRA-VS----
    62795                             D---------NYVYPAA---------------------------A---A-GDF-PE---------------GLLLLELVGMIVGKGLYEG---------ILQEVRLAPFFAKA-VL----
    91960                             G--------GVTLHPNA---------------------------M---S-GEVTPE---------------HLEYFASLGRLAAVALYHG---------ETLPLRLTSAFCKR-LL----
    52147                             D--------KGCYQPNQ---------------------------N---S-VVH-PD---------------YLSYFRFVGRLVGKALFDD---------ILLNAYFTRPIYKH-LL----
    36723                             E---------RTLYPNP---------------------------A---SELHAGER---------------HLQYFYFLGAILGKACYDG---------ILLDVPLADFFLAS-LK----
    31158                             D--------DARTYKRK---------------------------E-----GIDRPS---------------VLKELQCFGELLAHVVLFG--------SAVLPIPFSKVFLRR-VI----
    39499                             E-DDL-VVAPHGLFPAP-----------ITPPRL---------------GGKTHAS---------------RLKNFKLLGQSIGKVLQDG---------RMLDLPLAPAFYRM-LL----
    41776                             E---------RTLYPNP---------------------------A---SELHAGER---------------HLQYFYFLGAILGKACYDG---------ILLDVPLADFFLAS-LK----
    41898                             D--------KGCYQPNQ---------------------------N---S-VVH-PD---------------YLSYFRFVGRLVGKALFDD---------ILLNAYFTRPIYKH-LL----
    43113                             T---------GLIYPSP---------------------------R---A-GDT-HE---------------GILLLEMIGMMIGKGMYEG---------ILQDINMAPFFAAH-VL----
    Thecc1EG022084t1                  D-SGI-LINPYGLFPHP-----------WSPTTD----------S---CNGIQFSE---------------VLKKFVLLGQIVAKAIQDG---------RVLDVPFSKAFYKI-IL----
    Thecc1EG022374t1                  D---------RLLIPNP---------------------------A---A-RYL-EN---------------GIQMIEFLGRVVGKALYEG---------ILLDYSFSHVFVQK-LL----
    Thecc1EG030368t1                  N-------DRRRFFPNP---------------------------A---S-RVD-PL---------------HLEYFSFAGRVIALALMHK---------VQVGVVFDRVFFLQ-LA----
    Thecc1EG030623t1                  N--------NATFQPNS---------------------------N---S-VYQ-TE---------------HLSYFKFVGRVVAKALFDG---------QLLDVYFTRSFYKH-IL----
    Thecc1EG021434t2                  D---------HLLYPNP---------------------------G---S-GMIHEQ---------------HLQFYHFLGTLLAKAMFEG---------ILVDIPFATFFLSK-LK----
    Thecc1EG034540t1                  N--------ESTFQPNP---------------------------N---S-VYQ-TE---------------HLSYFKFVGRVVGKALFDG---------QLLDVHFTRSFYKH-IL----
    Thecc1EG006633t1                  E-GDI-IQAPLGLFPRP-----------WPPNVD----------A---SEGSQFCT---------------VIEYFRLVGRVMAKALQDG---------RLLDLPLSTPFYKL-VL----
    Cre08.g364550.t1.3                E--------TRTFWFNT---------------------------AM-AA-LEA--DG-EGGGGGGLGADASGLAEYRLVGLVLGLAIYNG---------VILDVHLPQVVYKK-LL----
    Cre07.g312900.t1.3                E-CEY-VNAPWGLFPRP-----------LPPASVSGS-------G---GAGAAAAK---------------VVEHFRLLGRTLAKALQDN---------RLLDLPLSHVFFAA-AL----
    g11539.t1                         A--------SHLHWFRPAGK-----------------------LA---A-EDM--E-----------------LEFELVGILIGLAIYNS---------HILEFQFPPVLYKK-LM----
    Cre06.g280300.t1.3                A---------HQLYPNP---------------------------A---A-MCVVED---------------APRLLAFLGRMLGKAMYEN---------VLLELPLAGFFLKR-FR----
    Cre02.g099100.t1.3                A--------SRTYWFNA---------------------------S---S-LEP--A-----------------SSYFLLGLVLGLAVYNR---------VLLAFPAPLLLYQR-LR----
    Cre03.g159200.t1.2                D---------GLAYPNP---------------------------A---A-ERI-PG---------------GLALLEFMGLMFGKALYEG---------ILLPVPFAHFFVAR-LQ----
    Cre01.g022100.t1.2                D--------PRCLHLSA---------------------------AGCLS-QGQ-ED---------------HLELINFAGRVLGLAMRAR---------VPLGFHLCTALYKL-LQH---
    Cre01.g012450.t1.3                E-------GGSTFQPNP---------------------------N---S-HVQ-ND--RG---------ISHLDYFRFVGRVVGKALHDG---------QLIDAYFTRSFYKH-ML----
    Cre10.g433900.t1.3                P-------QRNQLYVTP---------------------------T---S-SS--PA---------------HLKKFAFVGLFMAKAIVESAARGKELGPITLNLPLCEPFWKL-LL----
    Cre12.g533750.t1.3                D--------RRAVHPSA---------------------------A---A-ALQ-DD---------------HLGYMRFAGRIVGLALRAN---------VPLGVVLSTGLFNF-LT----
    Cre12.g548100.t1.3                ---------AGAYWYNT---------------------------G--LS---ESPE---------------LKGAYAFAGWLMGQSLLNR---------APLGLPLPPVLFRG-LLE---

    Selected Cols:                                                                                                                                            

    Gaps Scores:                                                                                                                                              

                                             490       500       510       520       530       540       550       560       570       580       590       600
                                      =========+=========+=========+=========+=========+=========+=========+=========+=========+=========+=========+=========+
    Sb01g011845.1                     ----G-------------------------------------------------------------------------------------------------------------------
    Sb02g016200.1                     ----G-------------------------------------------------------------------------------------------------------------------
    Sb04g000340.1                     ----G-------------------------------------------------------------------------------------------------------------------
    Sb06g003290.1                     ----G-------------------------------------------------------------------------------------------------------------------
    Sb08g012560.1                     ----G-------------------------------------------------------------------------------------------------------------------
    Sb09g002120.1                     ----E-------------------------------------------------------------------------------------------------------------------
    Sb09g004530.1                     ----Q-------------------------------------------------------------------------------------------------------------------
    Sb09g022820.1                     ----G-------------------------------------------------------------------------------------------------------------------
    73381                             ----G-------------------------------------------------------------------------------------------------------------------
    50844                             ----G-------------------------------------------------------------------------------------------------------------------
    89794                             ----G-------------------------------------------------------------------------------------------------------------------
    3542                              ----G-------------------------------------------------------------------------------------------------------------------
    76253                             ----G-------------------------------------------------------------------------------------------------------------------
    443962                            ----K-------------------------------------------------------------------------------------------------------------------
    181768                            ----G-------------------------------------------------------------------------------------------------------------------
    407700                            ----G------------------------------EVLN------------------------------------------------------------------------------EDD
    146155                            ----G-------------------------------------------------------------------------------------------------------------------
    154179                            ----G-------------------------------------------------------------------------------------------------------------------
    943823                            ----G-------------------------------------------------------------------------------------------------------------------
    487067                            ----G-------------------------------------------------------------------------------------------------------------------
    485684                            ----G-------------------------------------------------------------------------------------------------------------------
    490058                            ----G-------------------------------------------------------------------------------------------------------------------
    479191                            ----Q-------------------------------------------------------------------------------------------------------------------
    916552                            ----G-------------------------------------------------------------------------------------------------------------------
    940321                            ----G-------------------------------------------------------------------------------------------------------------------
    474651                            ----G-------------------------------------------------------------------------------------------------------------------
    915021                            ----G-------------------------------------------------------------------------------------------------------------------
    evm.model.supercontig_146.73      ----Q-------------------------------------------------------------------------------------------------------------------
    evm.model.supercontig_21.42       ----G-------------------------------------------------------------------------------------------------------------------
    evm.model.supercontig_37.145      ----G-------------------------------------------------------------------------------------------------------------------
    evm.model.supercontig_5.113       ----G-------------------------------------------------------------------------------------------------------------------
    evm.model.supercontig_959.1       ----G-------------------------------------------------------------------------------------------------------------------
    29206.m000140                     ----G-------------------------------------------------------------------------------------------------------------------
    29596.m000712                     ----G-------------------------------------------------------------------------------------------------------------------
    29602.m000214                     ----N-------------------------------------------------------------------------------------------------------------------
    29629.m001405                     ----G-------------------------------------------------------------------------------------------------------------------
    29805.m001489                     ----Q-------------------------------------------------------------------------------------------------------------------
    29815.m000491                     ----G-------------------------------------------------------------------------------------------------------------------
    29889.m003352                     ----Q-------------------------------------------------------------------------------------------------------------------
    Cucsa.042120.1                    ----G-------------------------------------------------------------------------------------------------------------------
    Cucsa.044750.1                    ----G-------------------------------------------------------------------------------------------------------------------
    Cucsa.160480.1                    ----G-------------------------------------------------------------------------------------------------------------------
    Cucsa.234290.1                    ----G-------------------------------------------------------------------------------------------------------------------
    Cucsa.307200.1                    ----G-------------------------------------------------------------------------------------------------------------------
    Cucsa.378730.1                    ----G-------------------------------------------------------------------------------------------------------------------
    ppa000451m                        ----G-------------------------------------------------------------------------------------------------------------------
    ppa000008m                        ----G-------------------------------------------------------------------------------------------------------------------
    ppa001143m                        ----G-------------------------------------------------------------------------------------------------------------------
    ppa000674m                        ----Q-------------------------------------------------------------------------------------------------------------------
    ppa000169m                        ----G-------------------------------------------------------------------------------------------------------------------
    ppa000009m                        ----G-------------------------------------------------------------------------------------------------------------------
    ppa000080m                        ----G-------------------------------------------------------------------------------------------------------------------
    mgv1a001314m                      ----G-------------------------------------------------------------------------------------------------------------------
    mgv1a000078m                      ----G-------------------------------------------------------------------------------------------------------------------
    mgv1a000005m                      ----G-------------------------------------------------------------------------------------------------------------------
    mgv11b024345m                     ----G-------------------------------------------------------------------------------------------------------------------
    mgv1a000436m                      ----G-------------------------------------------------------------------------------------------------------------------
    mgv1a000163m                      ----G-------------------------------------------------------------------------------------------------------------------
    GSVIVT01003328001                 ----G-------------------------------------------------------------------------------------------------------------------
    GSVIVT01009206001                 ----G-------------------------------------------------------------------------------------------------------------------
    GSVIVT01014698001                 ----G-------------------------------------------------------------------------------------------------------------------
    GSVIVT01018731001                 ----G-------------------------------------------------------------------------------------------------------------------
    GSVIVT01024033001                 ----G-------------------------------------------------------------------------------------------------------------------
    GSVIVT01025537001                 ----G-------------------------------------------------------------------------------------------------------------------
    GSVIVT01033734001                 ----G-------------------------------------------------------------------------------------------------------------------
    GSVIVT01034942001                 ----Q-------------------------------------------------------------------------------------------------------------------
    cassava4.1_000003m                ----G-------------------------------------------------------------------------------------------------------------------
    cassava4.1_000080m                ----G-------------------------------------------------------------------------------------------------------------------
    cassava4.1_002295m                ----Q-------------------------------------------------------------------------------------------------------------------
    cassava4.1_000006m                ----G-------------------------------------------------------------------------------------------------------------------
    cassava4.1_000011m                ----G-------------------------------------------------------------------------------------------------------------------
    cassava4.1_000177m                ----Q-------------------------------------------------------------------------------------------------------------------
    Pp1s205_47V6.1                    ----G-------------------------------------------------------------------------------------------------------------------
    Pp1s148_98V6.1                    ----G-------------------------------------------------------------------------------------------------------------------
    Pp1s103_43V6.1                    ----G-------------------------------------------------------------------------------------------------------------------
    Pp1s42_128V6.2                    ----G-------------------------------------------------------------------------------------------------------------------
    Pp1s263_1V6.1                     ----G-------------------------------------------------------------------------------------------------------------------
    Pp1s263_20V6.1                    ----G-------------------------------------------------------------------------------------------------------------------
    Pp1s15_454V6.1                    ----G-------------------------------------------------------------------------------------------------------------------
    Pp1s67_251V6.1                    ----E-------------------------------------------------------------------------------------------------------------------
    Pp1s173_137V6.1                   ----K-------------------------------------------------------------------------------------------------------------------
    Pp1s116_90V6.1                    ----GQASEGANERRYWFWSA--------VRMSLFGPCGVVQSGVADTWLWSSRGS--------------SVLQVCVTWVDVGG------WV----------------------------
    Pp1s138_130V6.1                   ----G-------------------------------------------------------------------------------------------------------------------
    Pp1s229_59V6.1                    ----R-------------------------------------------------------------------------------------------------------------------
    Pp1s88_123V6.1                    ----G-------------------------------------------------------------------------------------------------------------------
    orange1.1g000286m                 ----G-------------------------------------------------------------------------------------------------------------------
    orange1.1g045956m                 ----G-------------------------------------------------------------------------------------------------------------------
    orange1.1g000014m                 ----G-------------------------------------------------------------------------------------------------------------------
    orange1.1g001688m                 ----Q-------------------------------------------------------------------------------------------------------------------
    orange1.1g000012m                 ----G-------------------------------------------------------------------------------------------------------------------
    AT4G12570.1                       ----G-------------------------------------------------------------------------------------------------------------------
    AT4G38600.1                       ----G-------------------------------------------------------------------------------------------------------------------
    AT1G55860.1                       ----G-------------------------------------------------------------------------------------------------------------------
    AT1G70320.1                       ----G-------------------------------------------------------------------------------------------------------------------
    AT3G53090.1                       ----G-------------------------------------------------------------------------------------------------------------------
    AT3G17205.1                       ----Q-------------------------------------------------------------------------------------------------------------------
    AT5G02880.1                       ----G-------------------------------------------------------------------------------------------------------------------
    Si034011m                         ----G-------------------------------------------------------------------------------------------------------------------
    Si016079m                         ----G-------------------------------------------------------------------------------------------------------------------
    Si013562m                         ----G-------------------------------------------------------------------------------------------------------------------
    Si013264m                         ----G-------------------------------------------------------------------------------------------------------------------
    Si009242m                         ----Q-------------------------------------------------------------------------------------------------------------------
    Si009164m                         ----G-------------------------------------------------------------------------------------------------------------------
    Si024055m                         ----E-------------------------------------------------------------------------------------------------------------------
    Si020966m                         ----G-------------------------------------------------------------------------------------------------------------------
    Si020939m                         ----G-------------------------------------------------------------------------------------------------------------------
    Si028891m                         ----G-------------------------------------------------------------------------------------------------------------------
    Si028637m                         ----G-------------------------------------------------------------------------------------------------------------------
    Thhalv10019984m                   ----Q-------------------------------------------------------------------------------------------------------------------
    Thhalv10011172m                   ----G-------------------------------------------------------------------------------------------------------------------
    Thhalv10011171m                   ----G-------------------------------------------------------------------------------------------------------------------
    Thhalv10024192m                   ----G-------------------------------------------------------------------------------------------------------------------
    Thhalv10028412m                   ----G-------------------------------------------------------------------------------------------------------------------
    Thhalv10012430m                   ----G-------------------------------------------------------------------------------------------------------------------
    Thhalv10010078m                   ----G-------------------------------------------------------------------------------------------------------------------
    Ciclev10000001m                   ----G-------------------------------------------------------------------------------------------------------------------
    Ciclev10004231m                   ----Q-------------------------------------------------------------------------------------------------------------------
    Ciclev10007219m                   ----G-------------------------------------------------------------------------------------------------------------------
    Ciclev10010897m                   ----G-------------------------------------------------------------------------------------------------------------------
    Ciclev10010940m                   ----G-------------------------------------------------------------------------------------------------------------------
    Ciclev10027670m                   ----G-------------------------------------------------------------------------------------------------------------------
    Ciclev10014213m                   ----G-------------------------------------------------------------------------------------------------------------------
    GRMZM2G034622_T02                 ----Q-------------------------------------------------------------------------------------------------------------------
    GRMZM2G124297_T01                 ----E-------------------------------------------------------------------------------------------------------------------
    GRMZM2G411536_T03                 ----G-------------------------------------------------------------------------------------------------------------------
    GRMZM2G181378_T01                 ----G-------------------------------------------------------------------------------------------------------------------
    GRMZM2G049141_T01                 ----G-------------------------------------------------------------------------------------------------------------------
    GRMZM2G080439_T01                 ----G-------------------------------------------------------------------------------------------------------------------
    GRMZM2G021299_T01                 ----G-------------------------------------------------------------------------------------------------------------------
    GRMZM2G328988_T01                 ----G-------------------------------------------------------------------------------------------------------------------
    GRMZM2G331368_T02                 ----G-------------------------------------------------------------------------------------------------------------------
    GRMZM2G461948_T01                 ----G-------------------------------------------------------------------------------------------------------------------
    GRMZM2G374574_T01                 ----G-------------------------------------------------------------------------------------------------------------------
    Carubv10016604m                   ----G-------------------------------------------------------------------------------------------------------------------
    Carubv10011657m                   ----G-------------------------------------------------------------------------------------------------------------------
    Carubv10007210m                   ----G-------------------------------------------------------------------------------------------------------------------
    Carubv10003974m                   ----G-------------------------------------------------------------------------------------------------------------------
    Carubv10012881m                   ----Q-------------------------------------------------------------------------------------------------------------------
    Carubv10000054m                   ----G-------------------------------------------------------------------------------------------------------------------
    Carubv10000186m                   ----G-------------------------------------------------------------------------------------------------------------------
    Carubv10025730m                   ----G-------------------------------------------------------------------------------------------------------------------
    Bradi2g34820.1                    ----Q-------------------------------------------------------------------------------------------------------------------
    Bradi2g37870.1                    ----G-------------------------------------------------------------------------------------------------------------------
    Bradi2g22927.2                    ----G-------------------------------------------------------------------------------------------------------------------
    Bradi4g07997.2                    ----G-------------------------------------------------------------------------------------------------------------------
    Bradi4g33520.1                    ----G-------------------------------------------------------------------------------------------------------------------
    Bradi1g12340.2                    ----G-------------------------------------------------------------------------------------------------------------------
    Bradi5g04567.1                    ----G-------------------------------------------------------------------------------------------------------------------
    Bradi3g00350.1                    ----G-------------------------------------------------------------------------------------------------------------------
    Aquca_017_00766.1                 ----E-------------------------------------------------------------------------------------------------------------------
    Aquca_006_00259.1                 ----G-------------------------------------------------------------------------------------------------------------------
    Aquca_028_00189.1                 ----G-------------------------------------------------------------------------------------------------------------------
    Aquca_027_00123.1                 ----E-------------------------------------------------------------------------------------------------------------------
    Aquca_007_00539.1                 ----G-------------------------------------------------------------------------------------------------------------------
    Aquca_003_00437.1                 ----Q-------------------------------------------------------------------------------------------------------------------
    Aquca_019_00105.1                 ----G-------------------------------------------------------------------------------------------------------------------
    MDP0000264736                     ----G-------------------------------------------------------------------------------------------------------------------
    MDP0000320720                     ----G-------------------------------------------------------------------------------------------------------------------
    MDP0000142676                     ----G-------------------------------------------------------------------------------------------------------------------
    MDP0000318443                     ----E-------------------------------------------------------------------------------------------------------------------
    MDP0000206447                     ----G-------------------------------------------------------------------------------------------------------------------
    MDP0000196216                     ----G-------------------------------------------------------------------------------------------------------------------
    MDP0000186793                     ----Q-------------------------------------------------------------------------------------------------------------------
    MDP0000822588                     ----G-------------------------------------------------------------------------------------------------------------------
    MDP0000924418                     ----G-------------------------------------------------------------------------------------------------------------------
    MDP0000320505                     ----G-------------------------------------------------------------------------------------------------------------------
    MDP0000307848                     ----G-------------------------------------------------------------------------------------------------------------------
    MDP0000301275                     ----G-------------------------------------------------------------------------------------------------------------------
    MDP0000317971                     ----G-------------------------------------------------------------------------------------------------------------------
    Bra022201                         ----H-------------------------------------------------------------------------------------------------------------------
    Bra028860                         ----G-------------------------------------------------------------------------------------------------------------------
    Bra038022                         ----G-------------------------------------------------------------------------------------------------------------------
    Bra021231                         ----Q-------------------------------------------------------------------------------------------------------------------
    Bra005748                         ----G-------------------------------------------------------------------------------------------------------------------
    Bra000779                         ----N-------------------------------------------------------------------------------------------------------------------
    Bra029461                         ----H-------------------------------------------------------------------------------------------------------------------
    Bra027850                         ----G-------------------------------------------------------------------------------------------------------------------
    Bra040685                         ----G-------------------------------------------------------------------------------------------------------------------
    Bra010737                         ----G-------------------------------------------------------------------------------------------------------------------
    Medtr2g025830.1                   ----G-------------------------------------------------------------------------------------------------------------------
    Medtr2g025950.1                   ----G-------------------------------------------------------------------------------------------------------------------
    Medtr2g025810.1                   ----G-------------------------------------------------------------------------------------------------------------------
    Medtr2g033040.1                   ----Q-------------------------------------------------------------------------------------------------------------------
    Medtr2g025790.1                   ----G-------------------------------------------------------------------------------------------------------------------
    Medtr2g025930.1                   ----G-------------------------------------------------------------------------------------------------------------------
    Medtr7g100670.1                   ----G-------------------------------------------------------------------------------------------------------------------
    Medtr5g066710.1                   ----G-------------------------------------------------------------------------------------------------------------------
    Medtr4g073370.1                   ----G-------------------------------------------------------------------------------------------------------------------
    Medtr4g133120.1                   ----G-------------------------------------------------------------------------------------------------------------------
    Vocar20002255m                    ----G-------------------------------------------------------------------------------------------------------------------
    Vocar20010178m                    ----G-------------------------------------------------------------------------------------------------------------------
    Vocar20006334m                    ----G---------------------------------------------------HGTD---CG-------------------------------------------------------
    Vocar20007555m                    ----G-------------------------------------------------------------------------------------------------------------------
    Vocar20012583m                    ----G-------------------------------------------------------------------------------------------------------------------
    Vocar20003001m                    ----G-------------------------------------------------------------------------------------------------------------------
    Vocar20004069m                    ----GA------------------------------------------------------------------------------------------------------------------
    Vocar20000780m                    VRPPG-------------------------------------------------------------------------------------------------------------------
    Vocar20004842m                    ----G-------------------------------------------------------------------------------------------------------------------
    Vocar20014908m                    ----G-------------------------------------------------------------------------------------------------------------------
    Lus10032589                       ----G-------------------------------------------------------------------------------------------------------------------
    Lus10035589                       ----G-------------------------------------------------------------------------------------------------------------------
    Lus10005068                       ----G-------------------------------------------------------------------------------------------------------------------
    Lus10010493                       ----G-------------------------------------------------------------------------------------------------------------------
    Lus10027841                       ----G-------------------------------------------------------------------------------------------------------------------
    Lus10019908                       ----N-------------------------------------------------------------------------------------------------------------------
    Lus10032830                       ----G-------------------------------------------------------------------------------------------------------------------
    Lus10017098                       ----Q-------------------------------------------------------------------------------------------------------------------
    Lus10002605                       ----G-------------------------------------------------------------------------------------------------------------------
    Lus10008636                       ----G-------------------------------------------------------------------------------------------------------------------
    Eucgr.A01178.1                    ----G-------------------------------------------------------------------------------------------------------------------
    Eucgr.A01586.1                    ----G-------------------------------------------------------------------------------------------------------------------
    Eucgr.B03986.1                    ----Q-------------------------------------------------------------------------------------------------------------------
    Eucgr.D01414.1                    ----G-------------------------------------------------------------------------------------------------------------------
    Eucgr.D01416.1                    ----G-------------------------------------------------------------------------------------------------------------------
    Eucgr.F02160.1                    ----G-------------------------------------------------------------------------------------------------------------------
    Eucgr.I01410.2                    ----G-------------------------------------------------------------------------------------------------------------------
    Pavirv00038038m                   ----G-------------------------------------------------------------------------------------------------------------------
    Pavirv00031244m                   ----G-------------------------------------------------------------------------------------------------------------------
    Pavirv00010575m                   ----G-------------------------------------------------------------------------------------------------------------------
    Pavirv00004902m                   ----G-------------------------------------------------------------------------------------------------------------------
    Pavirv00020428m                   ----D-------------------------------------------------------------------------------------------------------------------
    Pavirv00067430m                   ----G-------------------------------------------------------------------------------------------------------------------
    Pavirv00058663m                   ----G-------------------------------------------------------------------------------------------------------------------
    Pavirv00067620m                   ----G-------------------------------------------------------------------------------------------------------------------
    Pavirv00029557m                   ----G-------------------------------------------------------------------------------------------------------------------
    Pavirv00023469m                   ----Q-------------------------------------------------------------------------------------------------------------------
    Pavirv00024250m                   ----G-------------------------------------------------------------------------------------------------------------------
    Pavirv00023205m                   ----G-------------------------------------------------------------------------------------------------------------------
    Pavirv00029138m                   ----E-------------------------------------------------------------------------------------------------------------------
    LOC_Os03g47949.1                  ----G-------------------------------------------------------------------------------------------------------------------
    LOC_Os02g01170.1                  ----G-------------------------------------------------------------------------------------------------------------------
    LOC_Os09g07900.1                  ----G-------------------------------------------------------------------------------------------------------------------
    LOC_Os12g24080.1                  ----G-------------------------------------------------------------------------------------------------------------------
    LOC_Os05g38830.1                  ----G-------------------------------------------------------------------------------------------------------------------
    LOC_Os05g03100.1                  ----E-------------------------------------------------------------------------------------------------------------------
    LOC_Os05g06690.1                  ----H-------------------------------------------------------------------------------------------------------------------
    PGSC0003DMT400075387              ----G-------------------------------------------------------------------------------------------------------------------
    PGSC0003DMT400021802              ----G-------------------------------------------------------------------------------------------------------------------
    PGSC0003DMT400031190              ----G-------------------------------------------------------------------------------------------------------------------
    PGSC0003DMT400072624              ----G-------------------------------------------------------------------------------------------------------------------
    Glyma14g36180.1                   ----G-------------------------------------------------------------------------------------------------------------------
    Glyma02g38020.2                   ----G-------------------------------------------------------------------------------------------------------------------
    Glyma12g03640.1                   ----G-------------------------------------------------------------------------------------------------------------------
    Glyma11g11490.1                   ----G-------------------------------------------------------------------------------------------------------------------
    Glyma06g00600.1                   ----G-------------------------------------------------------------------------------------------------------------------
    Glyma06g10360.1                   ----G-------------------------------------------------------------------------------------------------------------------
    Glyma04g00530.1                   ----C-------------------------------------------------------------------------------------------------------------------
    Glyma04g10481.1                   ----G-------------------------------------------------------------------------------------------------------------------
    Glyma08g09270.3                   ----G-------------------------------------------------------------------------------------------------------------------
    Glyma17g01210.2                   ----G-------------------------------------------------------------------------------------------------------------------
    Glyma17g04180.1                   ----Q-------------------------------------------------------------------------------------------------------------------
    Glyma13g19981.1                   ----G-------------------------------------------------------------------------------------------------------------------
    Glyma05g26360.1                   ----G-------------------------------------------------------------------------------------------------------------------
    Glyma19g37310.1                   ----G-------------------------------------------------------------------------------------------------------------------
    Glyma15g14591.1                   ----Q-------------------------------------------------------------------------------------------------------------------
    Glyma03g34650.2                   ----G-------------------------------------------------------------------------------------------------------------------
    Glyma10g05620.3                   ----G-------------------------------------------------------------------------------------------------------------------
    Glyma07g36390.1                   ----Q-------------------------------------------------------------------------------------------------------------------
    Glyma07g39546.1                   ----G-------------------------------------------------------------------------------------------------------------------
    Gorai.010G033100.1                ----G-------------------------------------------------------------------------------------------------------------------
    Gorai.010G186800.1                ----G-------------------------------------------------------------------------------------------------------------------
    Gorai.009G278900.1                ----G-------------------------------------------------------------------------------------------------------------------
    Gorai.009G228200.1                ----G-------------------------------------------------------------------------------------------------------------------
    Gorai.009G183200.1                ----G-------------------------------------------------------------------------------------------------------------------
    Gorai.009G420400.1                ----G-------------------------------------------------------------------------------------------------------------------
    Gorai.002G100900.1                ----G-------------------------------------------------------------------------------------------------------------------
    Gorai.002G196900.1                ----G-------------------------------------------------------------------------------------------------------------------
    Gorai.002G245000.1                ----G-------------------------------------------------------------------------------------------------------------------
    Gorai.002G003200.1                ----G-------------------------------------------------------------------------------------------------------------------
    Gorai.011G204200.1                ----Q-------------------------------------------------------------------------------------------------------------------
    Gorai.008G035900.1                ----G-------------------------------------------------------------------------------------------------------------------
    Gorai.006G265700.1                ----Q-------------------------------------------------------------------------------------------------------------------
    Potri.010G150000.3                ----Q-------------------------------------------------------------------------------------------------------------------
    Potri.009G134300.1                ----G-------------------------------------------------------------------------------------------------------------------
    Potri.004G174700.1                ----G-------------------------------------------------------------------------------------------------------------------
    Potri.011G094100.1                ----G-------------------------------------------------------------------------------------------------------------------
    Potri.006G132000.1                ----Q-------------------------------------------------------------------------------------------------------------------
    Potri.006G011700.1                ----G-------------------------------------------------------------------------------------------------------------------
    Potri.016G085200.3                ----Q-------------------------------------------------------------------------------------------------------------------
    Potri.016G096500.1                ----G-------------------------------------------------------------------------------------------------------------------
    Potri.016G012900.1                ----G-------------------------------------------------------------------------------------------------------------------
    Potri.002G110500.1                ----G-------------------------------------------------------------------------------------------------------------------
    Potri.008G101300.1                ----K-------------------------------------------------------------------------------------------------------------------
    Potri.001G368600.1                ----G-------------------------------------------------------------------------------------------------------------------
    Phvul.003G084200.1                ----G-------------------------------------------------------------------------------------------------------------------
    Phvul.003G118500.1                ----Q-------------------------------------------------------------------------------------------------------------------
    Phvul.009G119700.1                ----G-------------------------------------------------------------------------------------------------------------------
    Phvul.009G034900.1                ----G-------------------------------------------------------------------------------------------------------------------
    Phvul.011G035200.1                ----G-------------------------------------------------------------------------------------------------------------------
    Phvul.008G183200.1                ----G-------------------------------------------------------------------------------------------------------------------
    Phvul.007G163300.1                ----G-------------------------------------------------------------------------------------------------------------------
    Phvul.007G163400.1                ----G-------------------------------------------------------------------------------------------------------------------
    Phvul.001G184300.1                ----G-------------------------------------------------------------------------------------------------------------------
    Phvul.006G120900.1                ----Q-------------------------------------------------------------------------------------------------------------------
    Phvul.006G142800.1                ----G-------------------------------------------------------------------------------------------------------------------
    Phvul.002G189700.1                ----G-------------------------------------------------------------------------------------------------------------------
    mrna26562.1-v1.0-hybrid           ----G-------------------------------------------------------------------------------------------------------------------
    mrna05017.1-v1.0-hybrid           ----Q-------------------------------------------------------------------------------------------------------------------
    mrna09579.1-v1.0-hybrid           ----G-------------------------------------------------------------------------------------------------------------------
    mrna30084.1-v1.0-hybrid           ----G-------------------------------------------------------------------------------------------------------------------
    mrna07649.1-v1.0-hybrid           ----G-------------------------------------------------------------------------------------------------------------------
    mrna20590.1-v1.0-hybrid           ----G-------------------------------------------------------------------------------------------------------------------
    mrna19775.1-v1.0-hybrid           ----G-------------------------------------------------------------------------------------------------------------------
    Solyc04g076620.2.1                ----G-------------------------------------------------------------------------------------------------------------------
    Solyc10g083470.1.1                ----G-------------------------------------------------------------------------------------------------------------------
    Solyc10g055450.1.1                ----G-------------------------------------------------------------------------------------------------------------------
    Solyc07g065630.2.1                ----G-------------------------------------------------------------------------------------------------------------------
    Solyc05g054080.2.1                ----G-------------------------------------------------------------------------------------------------------------------
    Solyc01g057900.2.1                ----Q-------------------------------------------------------------------------------------------------------------------
    Solyc01g111530.2.1                ----G-------------------------------------------------------------------------------------------------------------------
    Solyc12g094560.1.1                ----G-------------------------------------------------------------------------------------------------------------------
    Solyc09g005150.1.1                ----G-------------------------------------------------------------------------------------------------------------------
    Solyc09g007310.2.1                ----G-------------------------------------------------------------------------------------------------------------------
    Solyc09g005160.1.1                ----G-------------------------------------------------------------------------------------------------------------------
    Solyc09g008700.1.1                ----G-------------------------------------------------------------------------------------------------------------------
    69212                             ----G-------------------------------------------------------------------------------------------------------------------
    70217                             ----G-------------------------------------------------------------------------------------------------------------------
    48481                             ----G-------------------------------------------------------------------------------------------------------------------
    19835                             ---DG-------------------------------------------------------------------------------------------------------------------
    213597                            ----G-------------------------------------------------------------------------------------------------------------------
    174890                            ----G-------------------------------------------------------------------------------------------------------------------
    154462                            ----G-------------------------------------------------------------------------------------------------------------------
    22875                             ----G-------------------------------------------------------------------------------------------------------------------
    172918                            ----G-------------------------------------------------------------------------------------------------------------------
    29762                             ----R-------------------------------------------------------------------------------------------------------------------
    67182                             ----Q-------------------------------------------------------------------------------------------------------------------
    58691                             ----G-------------------------------------------------------------------------------------------------------------------
    16350                             ----G-------------------------------------------------------------------------------------------------------------------
    35876                             ----G-------------------------------------------------------------------------------------------------------------------
    15978                             ----G-------------------------------------------------------------------------------------------------------------------
    37891                             ----G-------------------------------------------------------------------------------------------------------------------
    57759                             ----R-------------------------------------------------------------------------------------------------------------------
    59359                             ----N-------------------------------------------------------------------------------------------------------------------
    108435                            ---DG-------------------------------------------------------------------------------------------------------------------
    87459                             ----G-------------------------------------------------------------------------------------------------------------------
    60437                             ----G-------------------------------------------------------------------------------------------------------------------
    60965                             ----GA-DGGLARSKDGYVPGFVDPTDRFVGEKLGEVANRVGGVVARSGVEGAEGAEGAEGAEGTKLPAISRIKAAKERLERRGGTTDARWLRTDDSTRTDENLGEFELEFELEEGEEED
    83330                             ----G-------------------------------------------------------------------------------------------------------------------
    62795                             ----G-------------------------------------------------------------------------------------------------------------------
    91960                             ----G-------------------------------------------------------------------------------------------------------------------
    52147                             ----G-------------------------------------------------------------------------------------------------------------------
    36723                             ----G-------------------------------------------------------------------------------------------------------------------
    31158                             ----A-------------------------------------------------------------------------------------------------------------------
    39499                             ----G-------------------------------------------------------------------------------------------------------------------
    41776                             ----G-------------------------------------------------------------------------------------------------------------------
    41898                             ----G-------------------------------------------------------------------------------------------------------------------
    43113                             ----G-------------------------------------------------------------------------------------------------------------------
    Thecc1EG022084t1                  ----G-------------------------------------------------------------------------------------------------------------------
    Thecc1EG022374t1                  ----G-------------------------------------------------------------------------------------------------------------------
    Thecc1EG030368t1                  ----G-------------------------------------------------------------------------------------------------------------------
    Thecc1EG030623t1                  ----G-------------------------------------------------------------------------------------------------------------------
    Thecc1EG021434t2                  ----Q-------------------------------------------------------------------------------------------------------------------
    Thecc1EG034540t1                  ----G-------------------------------------------------------------------------------------------------------------------
    Thecc1EG006633t1                  ----G-------------------------------------------------------------------------------------------------------------------
    Cre08.g364550.t1.3                ----G-------------------------------------------------------------------------------------------------------------------
    Cre07.g312900.t1.3                ----G-------------------------------------------------------------------------------------------------------------------
    g11539.t1                         ----G-------------------------------------------------------------------------------------------------------------------
    Cre06.g280300.t1.3                ----G-------------------------------------------------------------------------------------------------------------------
    Cre02.g099100.t1.3                ----GG------------------------------------------------------------------------------------------------------------------
    Cre03.g159200.t1.2                ----G-------------------------------------------------------------------------------------------------------------------
    Cre01.g022100.t1.2                ---PG-------------------------------------------------------------------------------------------------------------------
    Cre01.g012450.t1.3                ----G-------------------------------------------------------------------------------------------------------------------
    Cre10.g433900.t1.3                ----G-------------------------------------------------------------------------------------------------------------------
    Cre12.g533750.t1.3                ----G-------------------------------------------------------------------------------------------------------------------
    Cre12.g548100.t1.3                ----GG-AGGAA----------------------------------------AHGAHGADGATAT-------------------------------------------------------

    Selected Cols:                                                                                                                                            

    Gaps Scores:                                                                                                                                              

                                             610       620       630       640       650       660       670       680       690       700       710       720
                                      =========+=========+=========+=========+=========+=========+=========+=========+=========+=========+=========+=========+
    Sb01g011845.1                     ---------R-------------Y-NFLDEL----------------STL------DRELYRNLMQ--------------------------------LK-------HY-----------
    Sb02g016200.1                     ---------A-------------K-VTYHDI----------------EAI------DPDYYKNLKW--------------------------------ML-------E------------
    Sb04g000340.1                     ---------Q-------------E-LDLFDI----------------VSF------DSEFGKTLQE--------------------------------LRV-LVERKQFL----------
    Sb06g003290.1                     ---------Q-------------E-LDLYDI----------------LSF------DTEFGKTLQE--------------------------------LQI-LVARKQFL----------
    Sb08g012560.1                     ---------V-------------K-VTYHDI----------------EAI------DPAYYKNLKW--------------------------------ML-------E------------
    Sb09g002120.1                     ---------Q-------------E-LDIYDI----------------PLF------DPELGKIVIE--------------------------------FQA-LVSRKNFL----------
    Sb09g004530.1                     ---------K-------------Y-NFLNDL----------------PSL------DPELYRHLLF--------------------------------LK-------HY-----------
    Sb09g022820.1                     ---------Q-------------E-LNIYDI----------------QSL------DSELATSLVE--------------------------------FQA-LACRRKYA----------
    73381                             ---------R-------------E-VTYMDM-------------------------EPEVSKSLVW--------------------------------LL-------E------------
    50844                             ---------L-------------P-ITWEDS----------------RDA------DPALYASCKN--------------------------------IL-------EM-----------
    89794                             ---------R-------------Y-CPIDDL----------------SSL------DGELHRNLMY--------------------------------LK-------NY-----------
    3542                              ---------Q-------------E-VTYKDM-------------------------DEEFVKNITW--------------------------------LL-------E------------
    76253                             ---------K-------------P-LALDDLGQVWQLRPRKALKTMFLQV------KPDLGKGLEH--------------------------------LL-------RF-----------
    443962                            ---------K-------------H-NYLHDL----------------PSL------DPELYKNLLF--------------------------------LK-------NN-----------
    181768                            ---------I-------------D-PRLQDL----------------RDL------QPQVYRSLNS--------------------------------LL-------AM-----------
    407700                            -----RRLAR--------GG-----LYEMDL----------------KAC------GPHIWNALNQ--------------------------------LRG-ISSLKE------------
    146155                            ---------Q-------------E-LDLYDV----------------KAI------DPELGSTLDE--------------------------------LQG-LVRRKQYL----------
    154179                            ---------T-------------K-VTYHDI----------------EAV------DPDYYKNLKW--------------------------------LL-------E------------
    943823                            ---------Q-------------E-LDLHDI----------------VLF------DAELGKTLQE--------------------------------LRV-LVARKHYL----------
    487067                            ---------Q-------------E-LSSFDI----------------HFV------DPELCKTMVE--------------------------------LQA-LARRKKVF----------
    485684                            ---------R-------------Y-SFIDEL----------------SGL------DPELYRNLMY--------------------------------VK-------HY-----------
    490058                            ---------L-------------K-ISLEDI----------------KDT------DRIMYNSCKQ--------------------------------IL-------EM-----------
    479191                            ---------K-------------Y-NYLNDL----------------PSL------DPELYRHLIF--------------------------------LK-------RY-----------
    916552                            ---------V-------------K-VTYHDI----------------EAI------DPDYYKNLKW--------------------------------ML-------E------------
    940321                            ---------M-------------E-IGLEDI----------------KET------EPILYKSLND--------------------------------ML-------ED-----------
    474651                            ---------V-------------K-VTYHDI----------------EAV------DPDYYKNLKW--------------------------------LL-------E------------
    915021                            ---------K-------------KLFSWKDL----------------IHT------DKELHKKYKE--------------------------------ML-------EM-----------
    evm.model.supercontig_146.73      ---------K-------------Y-NYLNDL----------------PSL------DPELYRHLIF--------------------------------LK-------HY-----------
    evm.model.supercontig_21.42       ---------V-------------K-VTYHDI----------------EAI------DPDYFKNLKW--------------------------------ML-------E------------
    evm.model.supercontig_37.145      ---------Q-------------E-LDLYDI----------------LSF------DSEFGKILQE--------------------------------LQL-LVCRKQYL----------
    evm.model.supercontig_5.113       ---------R-------------Y-SFLDEL----------------STL------DPELYRNLMY--------------------------------VK-------HY-----------
    evm.model.supercontig_959.1       ---------V-------------K-VTYHDI----------------EAV------DPDYYKNLKW--------------------------------ML-------E------------
    29206.m000140                     ---------R-------------H-ISLEDI----------------RDA------DPCLYTSCKQ--------------------------------VL-------EM-----------
    29596.m000712                     ---------A-------------K-VTYHDI----------------EAI------DPDYFKNLKW--------------------------------ML-------E------------
    29602.m000214                     ---------Q-------------E-LDLYDI----------------LSF------DAEFGKVLQE--------------------------------LHA-LVCRKRFL----------
    29629.m001405                     ---------V-------------K-VTYHDI----------------EAV------DPDYYKNLKW--------------------------------ML-------E------------
    29805.m001489                     ---------Q-------------E-LNLYDI----------------QSF------DPGLGKTLIE--------------------------------FQA-VVNRKKFL----------
    29815.m000491                     ---------R-------------Y-SFVDEL----------------STL------DPELYRNLMY--------------------------------VK-------HY-----------
    29889.m003352                     ---------K-------------F-NYLNDL----------------PSL------DPELYRHLIF--------------------------------LK-------HY-----------
    Cucsa.042120.1                    ---------V-------------K-VTYHDI----------------EAV------DPDYYKNLKW--------------------------------ML-------E------------
    Cucsa.044750.1                    ---------M-------------C-ISLEDI----------------RDA------DPCLYNSCKQ--------------------------------IL-------DM-----------
    Cucsa.160480.1                    ---------A-------------K-VTYHDI----------------EAI------DPDYYKNLKW--------------------------------ML-------E------------
    Cucsa.234290.1                    ---------Q-------------D-LDLHDI----------------LSF------DAELGKTLQE--------------------------------LQA-LVCRKQYL----------
    Cucsa.307200.1                    ---------Q-------------E-LSIYDI----------------QSF------DPELGTVLLE--------------------------------FQA-LVNRNKLL----------
    Cucsa.378730.1                    ---------R-------------Y-SFLDEL----------------STL------DPELYRNLMC--------------------------------VK-------SY-----------
    ppa000451m                        ---------R-------------Y-SFLDEL----------------STL------DPELYRNLMY--------------------------------VK-------HY-----------
    ppa000008m                        ---------V-------------K-VTYHDI----------------EAV------DPDYYKNLKW--------------------------------ML-------E------------
    ppa001143m                        ---------TL------------D-LSLEDI----------------RDA------DPFLYNSCKQ--------------------------------IL-------EM-----------
    ppa000674m                        ---------K-------------Y-NYLNDL----------------PSL------DQELYRHLIF--------------------------------LK-------HY-----------
    ppa000169m                        ---------Q-------------E-LGLYDI----------------QSF------DPELGRTLLE--------------------------------FKA-LMDRKKFM----------
    ppa000009m                        ---------A-------------K-VTYHDI----------------EAI------DPDYFKNLKW--------------------------------ML-------E------------
    ppa000080m                        ---------Q-------------D-LDLHDV----------------LSF------DAELGKTLQE--------------------------------LHN-LVCRKLYL----------
    mgv1a001314m                      ---------Q-------------T-ITLEDI----------------KDA------DPYLYNSCKQ--------------------------------IL-------EM-----------
    mgv1a000078m                      ---------H-------------E-LDLHDI----------------ISF------DAELGTTLQE--------------------------------LQA-LVFRKQYL----------
    mgv1a000005m                      ---------V-------------K-VTYHDI----------------EAV------DPDYYKNLKW--------------------------------ML-------E------------
    mgv11b024345m                     ---------QTQQ--------ALY-SCMDEL----------------PSL------DNELYRSLTF--------------------------------IK-------HY-----------
    mgv1a000436m                      ---------R-------------Y-SYVDEL----------------FTL------DPELHRNLMY--------------------------------VK-------HY-----------
    mgv1a000163m                      ---------K-------------E-LSLYDI----------------QSF------DPALGRALLE--------------------------------FQA-VVERKQYL----------
    GSVIVT01003328001                 ---------M-------------D-ISLEDI----------------QDA------DPLLYTSCKQ--------------------------------IL-------DM-----------
    GSVIVT01009206001                 ---------V-------------K-VTYHDI----------------EAI------DPDYFKNLKW--------------------------------ML-------E------------
    GSVIVT01014698001                 ---------V-------------K-VTYHDI----------------EAV------DPDYYKNLKW--------------------------------ML-------E------------
    GSVIVT01018731001                 ---------Q-------------D-LDLHDI----------------LSF------DAELGKILQE--------------------------------LQV-LVCRKQYL----------
    GSVIVT01024033001                 ---------Q-------------E-LDLHDI----------------LSF------DADFGKILQE--------------------------------LQV-LVSRKQYL----------
    GSVIVT01025537001                 ---------Q-------------E-LSVYDI----------------QSF------DPELGRVLLE--------------------------------FQA-LIDRKRYL----------
    GSVIVT01033734001                 ---------R-------------Y-SFLDEL----------------STL------DPELYRNLMY--------------------------------VK-------HY-----------
    GSVIVT01034942001                 ---------K-------------H-NYLNDL----------------PSL------DPELYRHLIF--------------------------------LK-------HF-----------
    cassava4.1_000003m                ---------V-------------K-VTYHDI----------------EAV------DPDYYKNLKW--------------------------------ML-------E------------
    cassava4.1_000080m                ---------Q-------------E-LDLYDI----------------LSF------DVELGKVLQE--------------------------------LHA-LVCRKHYM----------
    cassava4.1_002295m                ---------K-------------F-NYLNDL----------------PSL------DPELYRHLIF--------------------------------LK-------HY-----------
    cassava4.1_000006m                ---------V-------------K-VTYHDI----------------EAI------DPDYFKNLKW--------------------------------ML-------EIL----------
    cassava4.1_000011m                ---------V-------------K-VTYHDI----------------EAI------DPDYFKNLKW--------------------------------ML-------E------------
    cassava4.1_000177m                ---------Q-------------E-LNLYDI----------------QSF------DPELGRTLLE--------------------------------FQA-LVNRRKFL----------
    Pp1s205_47V6.1                    ---------L-------------P-VVWEDA----------------KDA------DPLLYESCKK--------------------------------IL-------EM-----------
    Pp1s148_98V6.1                    ---------L-------------P-VLWEDA----------------KDA------DPTLYESCKK--------------------------------IL-------EL-----------
    Pp1s103_43V6.1                    ---------R-------------Y-SFLDEL----------------SSL------DAELHRNLVY--------------------------------LK-------HY-----------
    Pp1s42_128V6.2                    ---------V-------------K-VTYHDI----------------EAI------DPGYFKNLKW--------------------------------IL-------E------------
    Pp1s263_1V6.1                     ---------Q-------------E-LDLYDM----------------QTV------DPDLGRTLFE--------------------------------MQG-LIQRKQFL----------
    Pp1s263_20V6.1                    ---------R-------------C-SFLDDL----------------SSE------DSELHRKLVY--------------------------------LK-------KA-----------
    Pp1s15_454V6.1                    ---------R-------------Y-SFLDEL----------------SSL------DAELHRNLVY--------------------------------LK-------HY-----------
    Pp1s67_251V6.1                    ---------K-------------P-VTYHDM----------------ASV------DVQFYKSLCW--------------------------------LL-------EN-----------
    Pp1s173_137V6.1                   ---------K-------------H-SYLHDL----------------PSL------DPELYQSMLF--------------------------------LK-------HY-----------
    Pp1s116_90V6.1                    ---------Q-------------E-LDLYDI----------------QTL------DPELGRTLFE--------------------------------MQG-LIRRKQLL----------
    Pp1s138_130V6.1                   ---------V-------------K-VTYHDI----------------EAI------DPDYFKNLKW--------------------------------IL-------E------------
    Pp1s229_59V6.1                    ---------E-------------S-VSLKDL----------------RDL------EPQIAKGLEE--------------------------------LF-------VY-----------
    Pp1s88_123V6.1                    ---------R-------------C-CFLDDL----------------RSI------DAELHRNLVN--------------------------------LK-------HS-----------
    orange1.1g000286m                 ---------H-------------E-LDLHDI----------------IPF------DAEFGKILQE--------------------------------LHV-IVCRKQHL----------
    orange1.1g045956m                 ---------R-------------Y-SFLDEL----------------STL------DPELYRNLMY--------------------------------VK-------HY-----------
    orange1.1g000014m                 ---------V-------------K-VTYHDI----------------EAI------DPDYFKNLKW--------------------------------ML-------E------------
    orange1.1g001688m                 ---------K-------------Y-NYLNDL----------------PSL------DPELYRHLIF--------------------------------LK-------HY-----------
    orange1.1g000012m                 ---------V-------------K-VTYHDI----------------EAV------DPDYYKNLKW--------------------------------ML-------E------------
    AT4G12570.1                       ---------L-------------K-ISLEDI----------------KDT------DRIMYNSCKQ--------------------------------IL-------EM-----------
    AT4G38600.1                       ---------Q-------------E-LDLHDI----------------VLF------DAELGKTLQE--------------------------------LRV-VVARKHYL----------
    AT1G55860.1                       ---------V-------------K-VTYHDI----------------EAV------DPDYYKNLKW--------------------------------LL-------E------------
    AT1G70320.1                       ---------V-------------K-VTYHDI----------------EAV------DPDYYKNLKW--------------------------------LL-------E------------
    AT3G53090.1                       ---------R-------------Y-SFIDEL----------------SGL------DPELYRNLMY--------------------------------VK-------HY-----------
    AT3G17205.1                       ---------K-------------Y-NYLNDL----------------PSL------DPELYRHLIF--------------------------------LK-------RY-----------
    AT5G02880.1                       ---------Q-------------E-LSSFDI----------------HFV------DPELCKTLVE--------------------------------LQA-LVRRKKLF----------
    Si034011m                         ---------R-------------Y-NFLDEL----------------STL------DPELYRNLMQ--------------------------------LK-------HY-----------
    Si016079m                         ---------Q-------------E-LDLFDI----------------VSF------DSEFGKTLQE--------------------------------LRV-LVERKKFL----------
    Si013562m                         ---------R-------------P-ITLDDI----------------ADP------DPSLHASCKK--------------------------------IL-------EM-----------
    Si013264m                         ---------R-------------P-ITLEDI----------------ADT------DPSLHASCKK--------------------------------IL-------EM-----------
    Si009242m                         ---------K-------------Y-NFLNDL----------------PSL------DPELYRHLLF--------------------------------LK-------HY-----------
    Si009164m                         ---------Q-------------E-LELYDV----------------LSF------DTEFGKILQE--------------------------------LQI-LVARKRFL----------
    Si024055m                         ---------Q-------------E-LDIYDI----------------PSF------DPELGKTLIE--------------------------------FQA-LVKRKKFM----------
    Si020966m                         ---------Q-------------E-LNIYDI----------------QLF------DSELAISLME--------------------------------FQA-IACRRKYA----------
    Si020939m                         ---------V-------------K-VTYHDI----------------EAI------DPAYYKNLKW--------------------------------ML-------E------------
    Si028891m                         ---------R-------------S-ITLEDI----------------SVA------DPVKYASCKR--------------------------------IL-------EM-----------
    Si028637m                         ---------A-------------K-VTYHDI----------------EAI------DPDYYKNLKW--------------------------------ML-------E------------
    Thhalv10019984m                   ---------K-------------Y-NYLNDL----------------PSL------DPELYRHLIF--------------------------------LK-------RY-----------
    Thhalv10011172m                   ---------V-------------K-VTYHDI----------------EAV------DPDYYKNLKW--------------------------------LL-------E------------
    Thhalv10011171m                   ---------V-------------K-VTYHDI----------------EAV------DPDYYKNLKW--------------------------------LL-------E------------
    Thhalv10024192m                   ---------Q-------------E-LDLHDI----------------MLF------DAELGKTLQE--------------------------------FRV-LVGRKHYL----------
    Thhalv10028412m                   ---------R-------------K-ICLEDI----------------KHT------DRIMYNSCKQ--------------------------------IL-------EM-----------
    Thhalv10012430m                   ---------Q-------------E-LSSFDI----------------HFV------DPELCKTLVE--------------------------------LQA-LARRRKVI----------
    Thhalv10010078m                   ---------R-------------Y-SFIDEL----------------SGL------DPELYRNLMY--------------------------------IK-------QY-----------
    Ciclev10000001m                   ---------V-------------K-VTYHDI----------------EAI------DPDYFKNLKW--------------------------------ML-------E------------
    Ciclev10004231m                   ---------K-------------Y-NYLNDL----------------PSL------DPELYRHLIF--------------------------------LK-------HY-----------
    Ciclev10007219m                   ---------V-------------K-VTYHDI----------------EAV------DPDYYKNLKW--------------------------------ML-------E------------
    Ciclev10010897m                   ---------K-------------E-LSLYDI----------------QSF------DPELGRTLLE--------------------------------FQA-IANRKKHL----------
    Ciclev10010940m                   ---------R-------------Y-SFLDEL----------------STL------DPELYRNLMY--------------------------------VK-------HY-----------
    Ciclev10027670m                   ---------H-------------E-LDLHDI----------------IPF------DAEFGKILQE--------------------------------LHV-IICRKQHL----------
    Ciclev10014213m                   ---------K-------------Y-ISLEDI----------------RDA------DPSLYSSCKQ--------------------------------IL-------EM-----------
    GRMZM2G034622_T02                 ---------K-------------Y-NFLNDL----------------PSL------DPELYRHLLF--------------------------------LK-------HY-----------
    GRMZM2G124297_T01                 ---------Q-------------E-LDMYDI----------------LSF------DPELGRTVIE--------------------------------FQA-LVSRKNFL----------
    GRMZM2G411536_T03                 ---------V-------------R-VTYHDI----------------EAI------DPAYYKNLKW--------------------------------ML-------E------------
    GRMZM2G181378_T01                 ---------R-------------S-ITLEDI----------------SVA------DPVKYASCKK--------------------------------IL-------EM-----------
    GRMZM2G049141_T01                 ---------Q-------------E-LDLYDI----------------LSF------DTEFGKTLQE--------------------------------LQI-LVARKQFL----------
    GRMZM2G080439_T01                 ---------R-------------S-ITLEDI----------------SVA------DPVKYASCKK--------------------------------IL-------EM-----------
    GRMZM2G021299_T01                 ---------A-------------K-VTYHDI----------------EAI------DPDYYRNLKW--------------------------------ML-------E------------
    GRMZM2G328988_T01                 ---------Q-------------E-LNIYDI----------------QSL------DSELAICLVE--------------------------------FQA-LACQRKYA----------
    GRMZM2G331368_T02                 ---------V-------------K-VTYHDI----------------EAI------DPSYYKNLKW--------------------------------ML-------E------------
    GRMZM2G461948_T01                 ---------R-------------Y-NFLDEL----------------STL------DPELYRNLMQ--------------------------------LK-------HY-----------
    GRMZM2G374574_T01                 ---------Q-------------E-LDLFDI----------------VSF------DSEFGKTLQE--------------------------------LQV-LVERKQFL----------
    Carubv10016604m                   ---------R-------------Y-SFIDEL----------------SGL------DPELYRNLMY--------------------------------IK-------NY-----------
    Carubv10011657m                   ---------V-------------K-VTYHDI----------------EAV------DPDYYKNLKW--------------------------------LL-------E------------
    Carubv10007210m                   ---------K-------------R-VSLEDI----------------RRT------DEAEYNSCLQ--------------------------------IL-------QL-----------
    Carubv10003974m                   ---------Q-------------E-LDLHDI----------------VLF------DAELGKTLQE--------------------------------LRV-LVARKHYL----------
    Carubv10012881m                   ---------K-------------Y-NYLNDL----------------PSL------DPELYRHLIF--------------------------------LK-------RF-----------
    Carubv10000054m                   ---------Q-------------E-LSSFDI----------------HFV------DPALCKTLVE--------------------------------LQA-LARRRKVF----------
    Carubv10000186m                   ---------L-------------K-ISLEDI----------------KET------DRVMYNSCKQ--------------------------------IL-------EM-----------
    Carubv10025730m                   ---------V-------------K-VTYHDI----------------EAI------DPDYYKNLKW--------------------------------ML-------E------------
    Bradi2g34820.1                    ---------K-------------S-NFLNDL----------------PSL------DPELYRHLLF--------------------------------LK-------HY-----------
    Bradi2g37870.1                    ---------Q-------------E-LDIYDI----------------PTF------DPELGKTVLE--------------------------------FQA-LVKRKKFL----------
    Bradi2g22927.2                    ---------Q-------------E-LNIYDI----------------NSF------DPELAMTLTE--------------------------------FKA-LTCQRKYI----------
    Bradi4g07997.2                    ---------A-------------K-VTYHDI----------------EAI------DPAYYRNLKW--------------------------------ML-------E------------
    Bradi4g33520.1                    ---------R-------------S-ITLEDI----------------AAA------DPVTYASCKR--------------------------------IL-------EM-----------
    Bradi1g12340.2                    ---------R-------------Y-SFLDEL----------------STL------DSELYRSLMQ--------------------------------LK-------HY-----------
    Bradi5g04567.1                    ---------Q-------------E-LDLYDI----------------LSF------DTEFGKILQE--------------------------------LQV-LVERKRFL----------
    Bradi3g00350.1                    ---------Q-------------E-LDLFDI----------------ISF------DAELGKTLQE--------------------------------MQV-LIERKRFL----------
    Aquca_017_00766.1                 ---------Q-------------D-LNLYDI----------------FSF------DHGVGRALVE--------------------------------FQA-LVDRKKVL----------
    Aquca_006_00259.1                 ---------Q-------------E-LDLHDI----------------LSF------DAEFGKVLQE--------------------------------LQV-LVCRKQHL----------
    Aquca_028_00189.1                 ---------K-------------S-VSLEDI----------------RDA------DPCLYMSCKK--------------------------------IL-------EM-----------
    Aquca_027_00123.1                 ---------Q-------------E-LNIYDI----------------HSI------DPELGRTLLE--------------------------------FQA-LIDRKKVS----------
    Aquca_007_00539.1                 ---------V-------------K-VTYHDI----------------EAI------DPDYFKNLKW--------------------------------ML-------E------------
    Aquca_003_00437.1                 ---------K-------------Y-NYLNDL----------------PSL------DPELYRHLIF--------------------------------LK--------------------
    Aquca_019_00105.1                 ---------R-------------Y-SFLDEL----------------STL------DPELYRNLIY--------------------------------VK-------HY-----------
    MDP0000264736                     ---------DT------------Y-LSLEDI----------------RDA------DPFLYNSCKR--------------------------------IL-------EM-----------
    MDP0000320720                     ---------K-------------E-LGVYDI----------------LSF------DPELGKTLLE--------------------------------FKA-LVDRKRFS----------
    MDP0000142676                     ---------DI------------Y-LSLEDI----------------RDA------DPFLYNSCKQ--------------------------------IL-------EM-----------
    MDP0000318443                     ---------A-------------K-VTYHDI----------------EAI------DPDYFKNLKW--------------------------------ML-------E------------
    MDP0000206447                     ---------V-------------K-VTYNDI----------------EAV------DPDYYKNLKW--------------------------------ML-------E------------
    MDP0000196216                     ---------R-------------Y-SFLDEL----------------STL------DPELYKNLIY--------------------------------VK-------HY-----------
    MDP0000186793                     ---------K-------------Y-NYLNDL----------------PSL------DPELYRHLIF--------------------------------LK-------RY-----------
    MDP0000822588                     ---------K-------------E-LGVYDI----------------LSF------DPELGKTLLE--------------------------------FKA-LVDRKRFS----------
    MDP0000924418                     ---------R-------------Y-SFLDEL----------------STL------DPELYKNLMY--------------------------------VK-------HY-----------
    MDP0000320505                     ---------Q-------------E-LDLHDV----------------LSF------DAELGKTLQE--------------------------------LHN-LVCRKLYL----------
    MDP0000307848                     ---------V-------------K-VTYHDI----------------EAV------DPDYYKNLKW--------------------------------ML-------E------------
    MDP0000301275                     ---------Q-------------E-LDLHDI----------------LSF------DAELGKTLQE--------------------------------LHN-LVCRKLYL----------
    MDP0000317971                     ---------A-------------K-VTYHDI----------------EAI------DPDYFKNLKW--------------------------------ML-------E------------
    Bra022201                         ---------K-------------Y-NYLNDL----------------PSL------DPELYKHLIF--------------------------------LK-------RY-----------
    Bra028860                         ---------Q-------------E-LSSFDI----------------HFV------DPELCKTLVE--------------------------------LQA-LARRRKVI----------
    Bra038022                         ---------V-------------K-VTYHDI----------------EAV------DPDYYKNLKW--------------------------------LL-------E------------
    Bra021231                         ---------K-------------Y-NYLNDL----------------PSL------DPELYRHLIF--------------------------------LK-------RY-----------
    Bra005748                         ---------Q-------------E-LSSFDI----------------HFI------DPELCKTLVE--------------------------------LQA-LTRRKKVF----------
    Bra000779                         ---------Q-------------T-ISLEDI----------------KDT------DRVIYNSCKQ--------------------------------IL-------EM-----------
    Bra029461                         ---------P-------------E-ISLEDI----------------KDT------DRVMYNSCKQ--------------------------------IL-------EM-----------
    Bra027850                         ---------V-------------K-VTYHDI----------------EAI------DPDYYKALKW--------------------------------ML-------E------------
    Bra040685                         ---------R-------------Y-SFIDEL----------------SGL------DPELYRNLMY--------------------------------IK-------QY-----------
    Bra010737                         ---------Q-------------E-LDLHDV----------------ILF------DAELGKTLQE--------------------------------LRV-LVGRKHYL----------
    Medtr2g025830.1                   ---------N-------------YIITLEDI----------------RNA------DPIMYSSCKQ--------------------------------IL-------EM-----------
    Medtr2g025950.1                   ---------N-------------YIITLEDI----------------RDA------DPIMYSSCKQ--------------------------------IL-------EM-----------
    Medtr2g025810.1                   ---------N-------------YIITLEDI----------------RDA------DPIMYSSCKQ--------------------------------IL-------EM-----------
    Medtr2g033040.1                   ---------K-------------H-NYLNDL----------------PSL------DPELYRHLIF--------------------------------LK-------RY-----------
    Medtr2g025790.1                   ---------N-------------YIITLEDI----------------RDA------DPIMYSSCKQ--------------------------------IL-------EM-----------
    Medtr2g025930.1                   ---------N-------------YIITLEDI----------------RNA------DPIMYSSCKQ--------------------------------IL-------EM-----------
    Medtr7g100670.1                   ---------K-------------K-LSLYDI----------------QSF------DPELGSTLLE--------------------------------FHA-LINRKKQL----------
    Medtr5g066710.1                   ---------V-------------K-VTYHDI----------------EAI------DPDYFKNLKW--------------------------------ML-------E------------
    Medtr4g073370.1                   ---------Q-------------D-LDLHDI----------------LYV------DAELGKTLQE--------------------------------LNA-LVCRKHNI----------
    Medtr4g133120.1                   ---------K-------------P-ITLEDI----------------RDA------DPDLYRSCKQ--------------------------------IL-------DM-----------
    Vocar20002255m                    ---------E-------------A-VGLSDL----------------EEA------FPTLGRSLRA--------------------------------VL-------SM-----------
    Vocar20010178m                    ---------S-------------P-LTHVDL----------------EAV------DPEYYKALAW--------------------------------ML-------S------------
    Vocar20006334m                    ---------S--------GGGGFL-PSLDML----------------SEF------DPDAAAGVRN--------------------------------VA-------SL-----------
    Vocar20007555m                    ---------N-------------P-LSLVDL----------------QQL------DPTEFRSLMS--------------------------------IL-------SM-----------
    Vocar20012583m                    ---------S-------------P-LDMWDI----------------ARI------DPGLGATLAK--------------------------------LHS-ALVAYRAA----------
    Vocar20003001m                    ---------A-------------H-CDLNDL----------------PTL------DPELYRSLLK--------------------------------LR-------DYFASTTSATTTA
    Vocar20004069m                    ---------R-------------E-PGLRDL----------------ETW------QPELARGLKQ--------------------------------LL-------EY-----------
    Vocar20000780m                    ---------R-------------Q-PLFDDL----------------ASL------DPELHKNLLM--------------------------------VK-------RY-----------
    Vocar20004842m                    ---------A-------------T-VA--DL----------------REL------APEVASSLAK--------------------------------LL-------QM-----------
    Vocar20014908m                    ---------R-------------R-ATLQDL----------------QQI------DPQVFTTCQN--------------------------------IL-------SS-----------
    Lus10032589                       ---------V-------------K-VTYHDI----------------EAV------DPDYYKNLKW--------------------------------ML-------E------------
    Lus10035589                       ---------R-------------Y-SFLDEL----------------STL------DQELYRNLMY--------------------------------VK-------HY-----------
    Lus10005068                       ---------Q-------------D-LDLYDI----------------LSF------DAELGKTLQE--------------------------------LHA-LVGRKQYL----------
    Lus10010493                       ---------G-------------P-IYLEDI----------------KDA------DPILYSSCKQ--------------------------------IL-------DM-----------
    Lus10027841                       ---------Q-------------D-LDLYDI----------------LSF------DAELGKTLQE--------------------------------LHA-LVGRKQYL----------
    Lus10019908                       ---------Q-------------E-LNICDI----------------PSF------DPELGRTLLE--------------------------------FQA-LFNRKKLL----------
    Lus10032830                       ---------V-------------K-VTYHDI----------------EAI------DPDYFKNLKW--------------------------------LL-------E------------
    Lus10017098                       ---------K-------------F-NYLNDL----------------PSL------DPELYRHLIF--------------------------------LK-------HY-----------
    Lus10002605                       ---------V-------------K-VTYHDI----------------EAI------DPDYFKNLKW--------------------------------LL-------E------------
    Lus10008636                       ---------R-------------Y-SFLDEL----------------STL------DQELYRNLMY--------------------------------VKMCLL--QHY-----------
    Eucgr.A01178.1                    ---------Q-------------D-LTLYDI----------------PSF------DPELGRTLLE--------------------------------FQA-LVDRKRFL----------
    Eucgr.A01586.1                    ---------R-------------Y-SFLDEL----------------STL------DPEVYRNLMY--------------------------------IK-------HF-----------
    Eucgr.B03986.1                    ---------K-------------Y-NYLHDL----------------PSL------DPELYRHLIF--------------------------------LK-------HY-----------
    Eucgr.D01414.1                    ---------Y-------------S-ISLEDI----------------RDA------DPYMYNSCKQ--------------------------------IL-------EM-----------
    Eucgr.D01416.1                    ---------Y-------------S-ISLEDI----------------RDA------DPDMYNNCKW--------------------------------IL-------ER-----------
    Eucgr.F02160.1                    ---------A-------------K-VTYHDI----------------EAI------DPDYFKNLKW--------------------------------ML-------E------------
    Eucgr.I01410.2                    ---------Q-------------E-LDLHDI----------------ISF------DAEVGKVLEE--------------------------------LHA-LVCRKQFL----------
    Pavirv00038038m                   ---------A-------------K-VTYHDI----------------EAI------DPAYYKNLKW--------------------------------ML-------E------------
    Pavirv00031244m                   ---------Q-------------E-LDLFDI----------------VSF------DSEFGKTLQE--------------------------------LRV-LVERKKFL----------
    Pavirv00010575m                   ---------R-------------S-ITLEDI----------------SVA------DPVKYASCKR--------------------------------IL-------EM-----------
    Pavirv00004902m                   ---------R-------------P-ITLDDI----------------ADT------DPSLHASCKK--------------------------------IL-------EM-----------
    Pavirv00020428m                   ---------K-------------E-LDIYDI----------------ASF------DPELGKTLIE--------------------------------FQA-LVNKKKFL----------
    Pavirv00067430m                   ---------R-------------S-ITLEDI----------------SVA------DPVKYASFKR--------------------------------IL-------EM-----------
    Pavirv00058663m                   ---------A-------------K-VTYHDI----------------EAI------DPDYYKNLKW--------------------------------ML-------E------------
    Pavirv00067620m                   ---------K-------------E-LDLFDI----------------VSF------DSEFGKTLQE--------------------------------LRV-LVERKKFL----------
    Pavirv00029557m                   ---------A-------------K-VTYHDI----------------EAI------DPAYYKNLKW--------------------------------ML-------E------------
    Pavirv00023469m                   ---------K-------------Y-NFLNDL----------------PSL------DPELYRHLLF--------------------------------LK-------HY-----------
    Pavirv00024250m                   ---------K-------------E-LNIYDI----------------QSF------DSELAISLME--------------------------------FQA-IDCRRKYA----------
    Pavirv00023205m                   ---------Q-------------E-LNIYDI----------------QSF------DSELAISLME--------------------------------FQA-IACRRKYA----------
    Pavirv00029138m                   ---------Q-------------E-LDMYDI----------------PSF------DPELGKTLIE--------------------------------FQA-LVNKKKFL----------
    LOC_Os03g47949.1                  ---------R-------------Y-SFLDEL----------------STL------DSELYRSLMQ--------------------------------LK-------HY-----------
    LOC_Os02g01170.1                  ---------Q-------------E-LDLFDI----------------ISF------DAEFGKTLQE--------------------------------LQI-LVERKRFL----------
    LOC_Os09g07900.1                  ---------V-------------K-VTYHDI----------------EAV------DPDYYKNLKW--------------------------------ML-------E------------
    LOC_Os12g24080.1                  ---------V-------------K-VTYHDI----------------EAI------DPAYYKNLKW--------------------------------ML-------E------------
    LOC_Os05g38830.1                  ---------Q-------------E-LNIYDI----------------HSF------DPELAMTLME--------------------------------FKA-LAARRKYL----------
    LOC_Os05g03100.1                  ---------Q-------------D-LDIYDI----------------PSF------DPKLGKTVME--------------------------------FQA-LVKRKKFL----------
    LOC_Os05g06690.1                  ---------K-------------Y-NFLNDL----------------PSL------DPELYRHLLS--------------------------------LK-------HY-----------
    PGSC0003DMT400075387              ---------E-------------D-ISLEDI----------------RDA------DPTLYSSCKQ--------------------------------IL-------EM-----------
    PGSC0003DMT400021802              ---------K-------------S-ISLDDI----------------RDA------DPFLYSSCRQ--------------------------------IL-------EM-----------
    PGSC0003DMT400031190              ---------Q-------------E-LDLYDI----------------LSF------DSELGKTLQE--------------------------------LQA-LVSRKQYI----------
    PGSC0003DMT400072624              ---------K-------------D-ITLEDV----------------RDA------DPPFYKSCKE--------------------------------IL-------EM-----------
    Glyma14g36180.1                   ---------V-------------K-VTYHDI----------------EAI------DPDYFKNLKW--------------------------------ML-------E------------
    Glyma02g38020.2                   ---------V-------------K-VTYHDI----------------EAI------DPHYFRNLKW--------------------------------ML-------E------------
    Glyma12g03640.1                   ---------Q-------------E-LDLHDI----------------LFI------DAELGKTLQE--------------------------------LNA-LVCRKCFI----------
    Glyma11g11490.1                   ---------Q-------------E-LDLHDI----------------LFI------DAELGKTLQE--------------------------------LNA-LVCRKHYI----------
    Glyma06g00600.1                   ---------Q-------------D-LDLHDI----------------LSI------DAELGKTLQE--------------------------------FNA-LVCRKHYI----------
    Glyma06g10360.1                   ---------A-------------K-VTYHDI----------------EAI------DPDYFRNLKW--------------------------------ML-------E------------
    Glyma04g00530.1                   ---------Q-------------D-LDLHDI----------------LFI------DAELGKTLQE--------------------------------FNA-LVCRKHYI----------
    Glyma04g10481.1                   ---------A-------------K-VTYHDI----------------EAI------DPDYFRNLKW--------------------------------ML-------E------------
    Glyma08g09270.3                   ---------V-------------K-VTYHDI----------------EAV------DPDYYKNLKW--------------------------------ML-------E------------
    Glyma17g01210.2                   ---------N-------------Y-IAIEDI----------------RDA------DPYLYTSCKQ--------------------------------IL-------DM-----------
    Glyma17g04180.1                   ---------K-------------H-NYLNDL----------------PSL------DPELYRHLIF--------------------------------LK-------HY-----------
    Glyma13g19981.1                   ---------K-------------E-LSLYDI----------------QSF------DPGLGKVLQE--------------------------------FQA-LVIRKKFV----------
    Glyma05g26360.1                   ---------V-------------K-VTYHDI----------------EAV------DPDYYKNLKW--------------------------------ML-------E------------
    Glyma19g37310.1                   ---------R-------------Y-SFLDEL----------------STL------DPELYRNLMY--------------------------------VK-------NY-----------
    Glyma15g14591.1                   ---------K-------------H-NYLNDL----------------PSL------DPELYRHLIF--------------------------------LK-------HY-----------
    Glyma03g34650.2                   ---------R-------------Y-SFLDEL----------------STL------DPELYRNLMY--------------------------------VK-------NY-----------
    Glyma10g05620.3                   ---------K-------------E-LSLYDI----------------QSF------DPGLGKVLQE--------------------------------FQA-LVMRKKFM----------
    Glyma07g36390.1                   ---------K-------------H-NYLNDL----------------PSL------DPELYRHLIF--------------------------------LK-------HY-----------
    Glyma07g39546.1                   ---------N-------------Y-IAIEDI----------------RDA------DPYLYTSCKQ--------------------------------IL-------DM-----------
    Gorai.010G033100.1                ---------V-------------K-VTYHDI----------------EAI------DPDYFKNLKW--------------------------------ML-------E------------
    Gorai.010G186800.1                ---------M-------------H-ISLEDI----------------RET------DPCLYSSCKK--------------------------------IL-------EM-----------
    Gorai.009G278900.1                ---------A-------------K-VTYHDI----------------EAI------DPDYFKNLKW--------------------------------ML-------E------------
    Gorai.009G228200.1                ---------A-------------K-VTYHDI----------------EAI------DPDYFKNLKW--------------------------------ML-------E------------
    Gorai.009G183200.1                ---------M-------------D-ISLEDI----------------REA------DPCLYSSCKK--------------------------------IL-------EM-----------
    Gorai.009G420400.1                ---------Q-------------D-LSLYDI----------------QSF------DPELGRTLLE--------------------------------FQAIIVNQKRHQ----------
    Gorai.002G100900.1                ---------V-------------K-VTYHDI----------------EAV------DPDYYKNLKW--------------------------------ML-------E------------
    Gorai.002G196900.1                ---------Q-------------E-LDLYDI----------------LSF------DGEFGKILQE--------------------------------LHF-LVCRKQYL----------
    Gorai.002G245000.1                ---------R-------------Y-SFLDEL----------------STL------DPELYRNLMY--------------------------------VK-------HY-----------
    Gorai.002G003200.1                ---------E-------------E-LDLHDI----------------PSF------DSEFGKILLE--------------------------------LHL-IVCRKKYL----------
    Gorai.011G204200.1                ---------K-------------Y-NYLNDL----------------PSL------DPELYRHLIF--------------------------------LK-------HY-----------
    Gorai.008G035900.1                ---------Q-------------E-LDLHDI----------------LSF------DAEFGKILQE--------------------------------LHL-LVRRKQYL----------
    Gorai.006G265700.1                ---------K-------------Y-NYLNDL----------------PSL------DPELYRHLIF--------------------------------LK-------HY-----------
    Potri.010G150000.3                ---------K-------------Y-NYLNDL----------------PSL------DPELYRHLIF--------------------------------LK--RESPSRY-----------
    Potri.009G134300.1                ---------Q-------------E-LDLYDI----------------LSF------DAEFGKTLQE--------------------------------LHA-LVCRKHYL----------
    Potri.004G174700.1                ---------Q-------------E-LDLYDF----------------LSF------DAEFGKTLQE--------------------------------LHA-LVRRKQYL----------
    Potri.011G094100.1                ---------A-------------K-VTYHDI----------------EAV------DPDYYKNLKW--------------------------------ML-------E------------
    Potri.006G132000.1                ---------Q-------------E-LNLYDI----------------QSF------DPELGRTLLE--------------------------------FQA-LVNRKKNM----------
    Potri.006G011700.1                ---------M-------------H-ISLEDI----------------RDA------DPCLYSSCKQ--------------------------------IL-------QM-----------
    Potri.016G085200.3                ---------Q-------------E-LNLYDI----------------QSF------DPELGRTLLE--------------------------------FQA-LVNRKKNM----------
    Potri.016G096500.1                ---------R-------------Y-SFLDEL----------------STL------DPELYRNVLY--------------------------------VK-------HY-----------
    Potri.016G012900.1                ---------M-------------H-ITLEDI----------------RDA------DPCLYSSCKQ--------------------------------IL-------QM-----------
    Potri.002G110500.1                ---------V-------------K-VTYHDI----------------EAI------DPDYFKNLKW--------------------------------ML-------E------------
    Potri.008G101300.1                ---------K-------------Y-NYLNDL----------------PSL------DSELYRHLIF--------------------------------LK-------RY-----------
    Potri.001G368600.1                ---------V-------------K-VTYHDI----------------EAV------DPDYYKNLKW--------------------------------ML-------E------------
    Phvul.003G084200.1                ---------S-------------Y-IALEDI----------------RNA------DPCLHTSCKQ--------------------------------IL-------DM-----------
    Phvul.003G118500.1                ---------K-------------Y-NYLNDL----------------PSL------DPELYRHLIF--------------------------------LK-------HY-----------
    Phvul.009G119700.1                ---------Q-------------E-LDLYDI----------------LFI------DAELGKTLQE--------------------------------LNA-LVRRKHYV----------
    Phvul.009G034900.1                ---------V-------------K-VTYHDI----------------EAI------DPAYFRNLKW--------------------------------ML-------E------------
    Phvul.011G035200.1                ---------Q-------------E-LDLHDI----------------LFI------DAELGKTLQE--------------------------------LNA-LVSRKRYI----------
    Phvul.008G183200.1                ---------V-------------K-VTYHDI----------------EAI------DPDYFKNLKW--------------------------------ML-------E------------
    Phvul.007G163300.1                ---------K-------------E-LSLYDI----------------LSF------DHGLGRVLQE--------------------------------FQA-LIIRKGVM----------
    Phvul.007G163400.1                ---------K-------------E-LFIYDI----------------QSF------DPELYMVLQE--------------------------------FQA-LVMRKKYI----------
    Phvul.001G184300.1                ---------R-------------Y-SFLVEL----------------STL------DPELYRNLMY--------------------------------VK-------NY-----------
    Phvul.006G120900.1                ---------K-------------H-NYLNDL----------------PSL------DPELYRHLIF--------------------------------LK-------HY-----------
    Phvul.006G142800.1                ---------L-------------R-VTLEDI----------------RDT------DPCLYRSCKQ--------------------------------IL-------EM-----------
    Phvul.002G189700.1                ---------V-------------K-VTYHDI----------------EAV------DPDYYKNLKW--------------------------------ML-------E------------
    mrna26562.1-v1.0-hybrid           ---------A-------------K-VTYHDI----------------EAI------DPDYFKNLKW--------------------------------ML-------E------------
    mrna05017.1-v1.0-hybrid           ---------K-------------Y-NYLNDL----------------PSL------DPELYRHLIF--------------------------------LK-------HF-----------
    mrna09579.1-v1.0-hybrid           ---------Q-------------D-LGLYDI----------------QSF------DPVLGRTLLE--------------------------------FKA-LVERKRFL----------
    mrna30084.1-v1.0-hybrid           ---------R-------------Y-SFLDEL----------------STL------DPEIYRNLMY--------------------------------VK-------HY-----------
    mrna07649.1-v1.0-hybrid           ---------Q-------------E-LDLHDV----------------LSF------DAELGKTLQE--------------------------------LHN-LVCRKLHL----------
    mrna20590.1-v1.0-hybrid           ---------V-------------K-VTYHDI----------------EAV------DPDYYKNLKW--------------------------------ML-------E------------
    mrna19775.1-v1.0-hybrid           ---------T-------------L-PSLEDI----------------RDA------DPFLYNSCKQ--------------------------------IL-------EM-----------
    Solyc04g076620.2.1                ---------A-------------K-VTYHDI----------------EAI------DPDYFKNLKW--------------------------------LL-------E------------
    Solyc10g083470.1.1                ---------K-------------D-ITLEDV----------------RDA------DPPFYKSCKE--------------------------------IL-------EM-----------
    Solyc10g055450.1.1                ---------Q-------------E-LDLYDI----------------LSF------DTELGKTLQE--------------------------------LQA-LVSRKQYI----------
    Solyc07g065630.2.1                ---------V-------------K-VTYHDI----------------EAV------DPDYYKNLKW--------------------------------ML-------E------------
    Solyc05g054080.2.1                ---------K-------------S-ISLDDI----------------RDA------DPFLYSSCRQ--------------------------------IL-------EM-----------
    Solyc01g057900.2.1                ---------K-------------Y-NYLNDL----------------PSL------DPELYRHLIF--------------------------------LK-------HY-----------
    Solyc01g111530.2.1                ---------Q-------------E-LDLYDI----------------LSF------DAELGKTLQE--------------------------------LQA-LVSRKQNL----------
    Solyc12g094560.1.1                ---------E-------------D-ISLEDI----------------RDA------DPSLYSSWKM--------------------------------IL-------KM-----------
    Solyc09g005150.1.1                ---------E-------------D-ISFEDI----------------IDA------DPYLYRGCKE--------------------------------IL-------EM-----------
    Solyc09g007310.2.1                ---------K-------------E-LTVYDI----------------QSF------DPELGGVLLE--------------------------------FQA-LVERKRHL----------
    Solyc09g005160.1.1                ---------E-------------D-ISFEDI----------------RDA------DPYLYSGCKK--------------------------------IL-------EM-----------
    Solyc09g008700.1.1                ---------R-------------Y-SFLDEL----------------STL------DPELYRNLMY--------------------------------VK-------HY-----------
    69212                             ---------H-------------A-MTIDDL----------------RSV------DPTLHKNMVEY-------------------------------VR-------EH-----------
    70217                             ---------A-------------P-RTIDDL----------------PSL------DPELHRSLIQ--------------------------------VL-------RY-----------
    48481                             ---------L-------------S-PTLADL----------------REL------SPSLGRGLEA--------------------------------ML-------EM-----------
    19835                             ---------R-------------P-PELNDL----------------ATL------DPELYHHLLS--------------------------------LK-------RL-----------
    213597                            ---------I-------------P-LNYEDM----------------EAF------DPDYHRNLAY--------------------------------ML-------E------------
    174890                            ---------R-------------T-LALADL----------------SEI------DPELGRTLTQ--------------------------------LST-AARRIDAL----------
    154462                            ---------R-----------ADA-AGWEDV----------------EEM------APQTARGYEN--------------------------------VR-------AM-----------
    22875                             ---------Q-------------T-PSMRDL----------------REV------NPWRHQGFAK--------------------------------LL-------KAR----------
    172918                            ---------E-------------A-LTEDDL----------------SSV------TPPAKRV--------------------------------------------RAC----------
    29762                             ---------R-------------G-CDLNDL----------------PSL------DAELYRQLLF--------------------------------LR-------DY-----------
    67182                             ---------R-------------P-IDLYDV----------------RKF------DAALGASLEK--------------------------------LAS----AHRAW----------
    58691                             ---------R-------------R-PTFLDL----------------KQA------MPELGRGLQQ--------------------------------LL-------NF-----------
    16350                             ---------R-------------D-VGFEDL----------------KDV------SPDVYSSLKK--------------------------------LL-------AY-----------
    35876                             ---------Q-------------P-AGMRDL----------------ADM------DPTLGKSLAQ--------------------------------LL-------EV-----------
    15978                             ---------R-------------R-PMFDEL----------------AAL------DPELHRNLLH--------------------------------LK-------RY-----------
    37891                             ---------Q-------------P-LTYQDI----------------EGV------DPEYFKNLTW--------------------------------LL-------E------------
    57759                             ---------E-------------T-LTEEDL----------------PAVYDDRCAGGAVARWLCG--------------------------------AVR-DVRRHREC----------
    59359                             ---------I-------------P-LNYDDM----------------EAF------DPDYHKSLVY--------------------------------ML-------E------------
    108435                            ---------R-------------P-PELNDL----------------ATL------DPELYHHLLS--------------------------------LK-------RL-----------
    87459                             ---------E-------------R-VGLADL----------------RDV------APWHHNAALC--------------------------------LL-------RC-----------
    60437                             ---------M-------------E-PTLVDL----------------CEL------SPSLGEGLQT--------------------------------LL-------DY-----------
    60965                             RATYLRALERVLSIAPGPHGDAAA-LRWLDL---------------MAEV------EPEFHRSLLD--------------------------------LL-------RYPIA--------
    83330                             ---------R-------------T-LALDDL----------------AEV------DPELGRTLSQ--------------------------------LSA-AAKRIDAL----------
    62795                             ---------I-------------P-RTLDDL----------------PGL------DPELHRSLIQ--------------------------------VL-------RY-----------
    91960                             ---------H-------------E-MALSDL----------------ESV------DPALYRNQVVY-------------------------------VR-------EH-----------
    52147                             ---------Q-------------Q-LTYEDM----------------EGV------DPDYYKSLKW--------------------------------ML-------E------------
    36723                             ---------R-------------H-VEFNDL----------------TTL------DPELYRNLVS--------------------------------LK-------RY-----------
    31158                             ---------NEKCD---------A-FTLDDL----------------ADV------EPAVVKSIKV--------------------------------VL-------ET-----------
    39499                             ---------R-------------S-LGLHDL----------------IEI------DPGLGNTLRR--------------------------------LDA-AANEIETM----------
    41776                             ---------R-------------H-VEFNDL----------------TTL------DPELYRNLVS--------------------------------LK-------RY-----------
    41898                             ---------Q-------------Q-LTYEDM----------------EGV------DPDYYKSLKW--------------------------------ML-------E------------
    43113                             ---------T-------------A-RTIDDI----------------PSL------DEDLARSIVQ--------------------------------IL-------EY-----------
    Thecc1EG022084t1                  ---------Q-------------D-LCLYDI----------------QSF------NPELGRTLLE--------------------------------FQA-IVDRKMHL----------
    Thecc1EG022374t1                  ---------R-------------Y-SFLDEL----------------STL------DPELYRNLMY--------------------------------VK-------HY-----------
    Thecc1EG030368t1                  ---------M-------------H-ISLEDI----------------REA------DPCLYSSCKK--------------------------------IL-------EM-----------
    Thecc1EG030623t1                  ---------V-------------K-VTYHDI----------------EAV------DPDYYKNLKW--------------------------------ML-------E------------
    Thecc1EG021434t2                  ---------K-------------Y-NYLNDL----------------PSL------DPELYRHLIF--------------------------------LK-------HY-----------
    Thecc1EG034540t1                  ---------V-------------K-VTYHDI----------------EAI------DPDYFKNLKW--------------------------------ML-------E------------
    Thecc1EG006633t1                  ---------Q-------------E-LDLHDI----------------LSF------DTEFGKTLQE--------------------------------LHL-LVCRKQYL----------
    Cre08.g364550.t1.3                ---------E-------------GPLGLADL----------------AEA------FPALGRSLAA--------------------------------VM-------AM-----------
    Cre07.g312900.t1.3                ---------S-------------P-LDMWDI----------------ARF------DPGLGATLAR--------------------------------LHA-ALVAHRAA----------
    g11539.t1                         ---------A-------------T-CP--EL----------------PAA------AAAAAGAAAEGAAAAAVGQGAGARAAAAAGSAAGAAAAAAGGAA-------DT-----------
    Cre06.g280300.t1.3                ---------A-------------H-CDLNDL----------------PTL------DPELYRNLLR--------------------------------LR-------EHLLS--------
    Cre02.g099100.t1.3                ---------R-------------E-LGLRDL----------------EGW------QPELAKGLRH--------------------------------IL-------EY-----------
    Cre03.g159200.t1.2                ---------R-------------Q-PLFDDL----------------ATL------DPELHKNLLM--------------------------------VK-------RY-----------
    Cre01.g022100.t1.2                ---------R-------PGATRAR-LGLADL----------------HQI------DPRVAATCGQ--------------------------------IA-------AA-----------
    Cre01.g012450.t1.3                ---------S-------------P-LTHVDL----------------EAV------DPEYYKALSW--------------------------------ML-------S------------
    Cre10.g433900.t1.3                ---------N-------------P-LSLVDL----------------QQL------DPTEFRSLMS--------------------------------IL-------SM-----------
    Cre12.g533750.t1.3                ---------R-------------R-GTLQDL----------------QQM------DPQLHSTCQN--------------------------------IM-------SM-----------
    Cre12.g548100.t1.3                ---------A--------ATSGFQ-PTLEML----------------SEF------DPDAANAVRN--------------------------------VA-------GL-----------

    Selected Cols:                                                                                                                                            

    Gaps Scores:                                                                                                                                              

                                             730       740       750       760       770       780       790       800       810       820       830       840
                                      =========+=========+=========+=========+=========+=========+=========+=========+=========+=========+=========+=========+
    Sb01g011845.1                     --------------------D-G------------------------------------D--------V---ED--LF-LDFTVTEE---------------LGG---------------
    Sb02g016200.1                     ----------------------N------------------------------------D--------V---SDL-PD-LTFSMDPDEEK----------HILYE---------------
    Sb04g000340.1                     --------------------E-STC-------------------------GKNQLQVA-D-LRFHGASI---ED--LC-LDFTLP-------------------G---------------
    Sb06g003290.1                     --------------------E-SCS-------------------------SENQ-KIE-E-LCFRGAPI---ED--LC-LDFTLP-------------------G---------------
    Sb08g012560.1                     ----------------------N------------------------------------D--------I---SDV-LD-LTFSMDADEEK----------LILYE---------------
    Sb09g002120.1                     --------------------E-TS--------------------------SRASNPMV-D-LTYKNVKL---ED--LC-LDFTLP-------------------G---------------
    Sb09g004530.1                     --------------------N-G------------------------------------D--------I---SE--LE-LYFVIVNN---------------EYG---------------
    Sb09g022820.1                     --------------------E-SNL-------------------------TRDCQIIS-D-LTYRGCRI---ED--LA-IEFALP-------------------G---------------
    73381                             ----------------------N------------------------------------K--------V---EES-MG-LSFSIDDHS---------------DG---------------
    50844                             --------------------D-P------------------------------------D-------SID--SDT-LG-LTFVTEME---------------LLG---------------
    89794                             --------------------E-G------------------------------------D--------V---SD--MA-LDFTVTEE---------------YFG---------------
    3542                              ----------------------N------------------------------------N--------V---DES-IG-LTFSVDIE--H----------CTSGG---------------
    76253                             --------------------T-G------------------------------------D--------V---EET-YS-RTFQISEI--------------DMFG---------------
    443962                            --------------------P-N------------------------------------M--------V---QQ--LG-LYFVIEDN---------------EYG---------------
    181768                            --------------------E--------------------------------------E--------I---ESM-DL-Y-FEVSYD---------------CFG---------------
    407700                            --------------------E-G------------------------------------D----RRKSV----DSILENLAIT--------------D-----YNAVL------------
    146155                            --------------------E-GVC-------------------------HQMSD----G-LRFRGSRI---ED--LC-LDFTLP-------------------G---------------
    154179                            ----------------------N------------------------------------D--------V---NDI-LG-LTFSIDADEEK----------HILYE---------------
    943823                            --------------------E-AVG-------------------------GDNSSTVS-D-LCLRGSRI---ED--LC-LDFTLP-------------------G---------------
    487067                            --------------------N-EAH-------------------------GDSRPAKC-D-LSFHGTKI---ED--LS-LGFALP-------------------G---------------
    485684                            --------------------D-G------------------------------------D--------L---KE--LC-LDFTVTEE---------------FCG---------------
    490058                            --------------------D-P------------------------------------V-------FFD--SNAGLG-LTFVLETE---------------ELG---------------
    479191                            --------------------K-G------------------------------------D--------I---SD--LE-LYFVILNN---------------EYG---------------
    916552                            ----------------------H------------------------------------D--------I---SDV-LD-LTFSIDADEEK----------LILYE---------------
    940321                            --------------------P-T------------------------------------K-------IGD-------G-LTFEIDVK---------------RGD---------------
    474651                            ----------------------N------------------------------------D--------V---SDI-LD-LTFSMDADEEK----------HILYE---------------
    915021                            --------------------D-A------------------------------------Q-------EFD--ALQGYG-LTFS-------------------------------------
    evm.model.supercontig_146.73      --------------------K-G------------------------------------N--------I---SE--LE-LYFVIVNN---------------EYG---------------
    evm.model.supercontig_21.42       ----------------------N------------------------------------D--------I---SDV-PD-LTFSIDADEEK----------LILYE---------------
    evm.model.supercontig_37.145      --------------------E-AVG-------------------------GDNSDAIS-N-LCFRGAPI---ED--LC-LDFTLP-------------------G---------------
    evm.model.supercontig_5.113       --------------------D-G------------------------------------D--------V---KD--LS-LDFTVTEE---------------SFG---------------
    evm.model.supercontig_959.1       ----------------------N------------------------------------D--------V---SDI-PD-LTFSMDADEEK----------HILYE---------------
    29206.m000140                     --------------------D-A------------------------------------N-------FID--SDA-LG-LTFVREVE---------------ELG---------------
    29596.m000712                     ----------------------N------------------------------------D--------I---SDV-LD-LTFSIDADEEK----------LILYE---------------
    29602.m000214                     --------------------E-SSG-------------------------TDNLDAIS-D-LRFRGTLI---ED--LC-LDFTLP-------------------G---------------
    29629.m001405                     ----------------------N------------------------------------D--------V---SDI-PD-LTFSMDADEEK----------HILYE---------------
    29805.m001489                     --------------------R-LAL-------------------------GENSCSNF-D-AYFRNTRI---ED--LF-LDFTLP-------------------G---------------
    29815.m000491                     --------------------D-G------------------------------------D--------L---KD--LF-LDFTITEE---------------SFG---------------
    29889.m003352                     --------------------Q-G------------------------------------D--------I---SN--LE-LYFVIVNN---------------EYG---------------
    Cucsa.042120.1                    ----------------------N------------------------------------D--------V---SDI-PD-LTFSMDADEEK----------HILYE---------------
    Cucsa.044750.1                    --------------------D-P------------------------------------G-------LVD--SDA-LG-LTFVSDFE---------------ELG---------------
    Cucsa.160480.1                    ----------------------N------------------------------------D--------I---SDV-LD-LTFSVDADEEK----------LILYE---------------
    Cucsa.234290.1                    --------------------G-SLN-------------------------GDNQNTIS-N-LTFRGIPV---ED--LC-LDFTVP-------------------G---------------
    Cucsa.307200.1                    --------------------G-SVY-------------------------EENSSSKL-E-FSYHNTNI---ED--LC-LDFTLP-------------------G---------------
    Cucsa.378730.1                    --------------------E-D------------------------------------D--------V---KE--LS-LDFTVTEE---------------SFG---------------
    ppa000451m                        --------------------E-G------------------------------------D--------V---EE--LC-LDFTVTEE---------------SFG---------------
    ppa000008m                        ----------------------N------------------------------------D--------V---SDI-PD-LTFSMDADEEK----------HILYE---------------
    ppa001143m                        --------------------D-A------------------------------------E-------FID--SDA-LG-LTFVREVE---------------ELG---------------
    ppa000674m                        --------------------K-G------------------------------------D--------I---SE--LE-LYFVIVNN---------------EYG---------------
    ppa000169m                        --------------------E-SVH-------------------------GR-TTFEF-D-SCFRKTKI---ED--LC-LDFTLP-------------------G---------------
    ppa000009m                        ----------------------N------------------------------------D--------I---SDV-LD-LTFSIDADEEK----------LILYE---------------
    ppa000080m                        --------------------E-SSG--------------------------DNCDAIA-E-LRFRGASI---DD--LC-FDFTLP-------------------G---------------
    mgv1a001314m                      --------------------D-P------------------------------------T-------TID--QDA-LG-LTFIDEKE---------------ELG---------------
    mgv1a000078m                      --------------------E-SVG-------------------------SYNPE----E-LRFRGASI---ED--LC-LDFSLP-------------------G---------------
    mgv1a000005m                      ----------------------N------------------------------------D--------V---SDI-PD-LTFSMDADEEK----------HILYE---------------
    mgv11b024345m                     --------------------K-Q------------------------------------D--------V---AD--LN-LTFSVDQD---------------VMG---------------
    mgv1a000436m                      --------------------D-G------------------------------------D--------V---KD--LC-LDFTVTEE---------------SLG---------------
    mgv1a000163m                      --------------------K-SHC--------------------------EDSSRDV-D-VLLRNTKI---ED--MC-LDFSLP-------------------G---------------
    GSVIVT01003328001                 --------------------D-A------------------------------------E-------FMD--SDA-LG-LTFVREIE---------------ELG---------------
    GSVIVT01009206001                 ----------------------N------------------------------------D--------I---TDV-LD-VTFSIDADEEK----------LILYE---------------
    GSVIVT01014698001                 ----------------------N------------------------------------D--------V---SCI-PE-MTFSMDPDEEK----------HILYE---------------
    GSVIVT01018731001                 --------------------D-GVS-------------------------GNGCDATG-G-LCFRGAPV---ED--LC-LDFTLP-------------------G---------------
    GSVIVT01024033001                 --------------------E-STG-------------------------GDNQDAIA-N-LCFRGAPI---ED--LC-LDFTLP-------------------G---------------
    GSVIVT01025537001                 --------------------E-TVC-------------------------GEKSTFDV-D-MCFRNTKI---ED--LY-LDFTLP-------------------G---------------
    GSVIVT01033734001                 --------------------D-G------------------------------------D--------V---KE--LS-LDFTVTEE---------------SLG---------------
    GSVIVT01034942001                 --------------------E-G------------------------------------D--------L---SE--LE-LYFVIVNN---------------EYG---------------
    cassava4.1_000003m                ----------------------N------------------------------------D--------V---SEI-PD-LTFSMDADEEK----------HILYE---------------
    cassava4.1_000080m                --------------------E-ALG-------------------------TDNRDAIA-G-LHFRGTAI---ED--LC-LDFTLP-------------------G---------------
    cassava4.1_002295m                --------------------Q-G------------------------------------D--------I---SE--LE-LYFVIVNN---------------EYG---------------
    cassava4.1_000006m                --------------------Q-N------------------------------------D--------I---SDV-LD-LTFSIDADEEK----------LILYE---------------
    cassava4.1_000011m                ----------------------N------------------------------------D--------I---SDV-LD-LTFSIDADEEK----------LILYE---------------
    cassava4.1_000177m                --------------------K-SAL-------------------------EENSCNTL-E-VCFRNSRI---ED--LS-LDFTLP-------------------G---------------
    Pp1s205_47V6.1                    --------------------D-A------------------------------------D-------LID--TDI-LG-LTFVSEVE---------------ELG---------------
    Pp1s148_98V6.1                    --------------------D-A------------------------------------D-------LID--TDI-LG-LTFVSEVE---------------ELG---------------
    Pp1s103_43V6.1                    --------------------E-G------------------------------------D--------A---RD--LA-LDFTVTEE---------------LFG---------------
    Pp1s42_128V6.2                    ----------------------N------------------------------------D--------I---SDL-PD-LTFSMDADEEK----------HILYE---------------
    Pp1s263_1V6.1                     --------------------E-GHG-------------------------GKRE-EVE-A-LNFRGSKL---ED--LC-LDFTLP-------------------G---------------
    Pp1s263_20V6.1                    --------------------S-E------------------------------------D--------A---QD--LK-LNFTVKED---------------LFD---------------
    Pp1s15_454V6.1                    --------------------G-G------------------------------------D--------A---RD--LA-LDFTVTEE---------------LFG---------------
    Pp1s67_251V6.1                    -----------------------------------------------------------K--------L---EGMDLG-LTFSVDTD---------------NFG---------------
    Pp1s173_137V6.1                   --------------------D-G------------------------------------D--------W---SQ--LA-AYFVVTHN---------------EYG---------------
    Pp1s116_90V6.1                    --------------------E-THG-------------------------GKRE-EVE-A-LTFRGSRL---ED--LC-LDFTLP-------------------G---------------
    Pp1s138_130V6.1                   ----------------------N------------------------------------D--------V---SDI-VG-LTFSMDADEEK----------HILYE---------------
    Pp1s229_59V6.1                    --------------------Q-G------------------------------------D--------V---EST-FC-QNFQITYE---------------YFG---------------
    Pp1s88_123V6.1                    --------------------K-A------------------------------------D--------A---RD--LS-LNFTVAQE---------------LSG---------------
    orange1.1g000286m                 --------------------E-SMT-------------------------SDNCEEVV-D-LRFRGAPI---ED--LC-LDFTLP-------------------G---------------
    orange1.1g045956m                 --------------------D-G------------------------------------D--------V---KE--LC-LDFTVTEE---------------SFG---------------
    orange1.1g000014m                 ----------------------N------------------------------------D--------I---SDV-LD-LTFSIDADEEK----------LILYE---------------
    orange1.1g001688m                 --------------------E-A------------------------------------D--------I---SE--LE-LYFVILNN---------------EYG---------------
    orange1.1g000012m                 ----------------------N------------------------------------D--------V---SDI-PD-LTFSMDADEEK----------HILYE---------------
    AT4G12570.1                       --------------------D-P------------------------------------E-------FFD--SNAGLG-LTFVLETE---------------ELG---------------
    AT4G38600.1                       --------------------E-GVG-------------------------GDNSSTIS-D-LCLRGCRI---ED--LS-LEFTLP-------------------G---------------
    AT1G55860.1                       ----------------------N------------------------------------D--------V---SDI-LD-LTFSMDADEEK----------HILYE---------------
    AT1G70320.1                       ----------------------N------------------------------------D--------V---SDI-LD-LTFSMDADEEK----------HILYE---------------
    AT3G53090.1                       --------------------D-G------------------------------------D--------L---KE--LC-LDFTVTEE---------------FCG---------------
    AT3G17205.1                       --------------------K-G------------------------------------D--------I---SD--LE-LYFVILNN---------------EYG---------------
    AT5G02880.1                       --------------------A-EAH-------------------------GDSGAAKC-D-LSFHGTKI---ED--LC-LEFALP-------------------G---------------
    Si034011m                         --------------------D-G------------------------------------D--------V---ED--LC-LDFTVTEE---------------LGG---------------
    Si016079m                         --------------------E-STP-------------------------GENQLEVA-D-LRFRGAAI---ED--LC-LDFTLP-------------------G---------------
    Si013562m                         --------------------D-P------------------------------------T-------LVD--SDV-LG-LRFIREVD---------------VLG---------------
    Si013264m                         --------------------D-P------------------------------------G-------LVD--SNT-LE-LTFVREDE---------------VLG---------------
    Si009242m                         --------------------N-G------------------------------------D--------I---SE--LE-LYFVIVNN---------------EYG---------------
    Si009164m                         --------------------E-SCC-------------------------SDSR-KIE-E-LCFRGAPV---ED--LC-LDFTLP-------------------G---------------
    Si024055m                         --------------------E-TS--------------------------LRTSSPTA---------------D--LC-LDFTLP-------------------G---------------
    Si020966m                         --------------------E-SNL-------------------------TRDCQIMS-D-LTYRGCRI---ED--LA-IDFALP-------------------G---------------
    Si020939m                         ----------------------N------------------------------------D--------I---TDV-LD-LTFSMDADEEK----------LILYE---------------
    Si028891m                         --------------------N-A------------------------------------A-------EID--D---LY-LTFSRGAH---------------ELG---------------
    Si028637m                         ----------------------N------------------------------------D--------V---SDL-PD-LTFSMDPDEEK----------HILYE---------------
    Thhalv10019984m                   --------------------K-G------------------------------------N--------I---SE--LE-LYFVILNN---------------EYG---------------
    Thhalv10011172m                   ----------------------N------------------------------------D--------V---SDI-LD-LTFSMDADEEK----------HILYE---------------
    Thhalv10011171m                   ----------------------N------------------------------------D--------V---SDI-LD-LTFSMDADEEK----------HILYE---------------
    Thhalv10024192m                   --------------------E-AIG-------------------------GDNCSAIS-D-LSLRGSRI---ED--LC-LDFTLP-------------------G---------------
    Thhalv10028412m                   --------------------D-P------------------------------------V-------EFD--SNGGLG-LTFVLETE---------------VLG---------------
    Thhalv10012430m                   --------------------A-EAQ-------------------------SDSRAAMY-D-LSFRGTKI---ED--LC-LEFALP-------------------G---------------
    Thhalv10010078m                   --------------------D-G------------------------------------D--------L---KE--LC-LDFTVTEE---------------FCG---------------
    Ciclev10000001m                   ----------------------N------------------------------------D--------I---SDV-LD-LTFSIDADEEK----------LILYE---------------
    Ciclev10004231m                   --------------------E-A------------------------------------D--------I---SE--LE-LYFVILNN---------------EYG---------------
    Ciclev10007219m                   ----------------------N------------------------------------D--------V---SDI-PD-LTFSMDADEEK----------HILYE---------------
    Ciclev10010897m                   --------------------E-STS-------------------------EERSMFGL-E-SCFRNTRV---ED--LC-LDFTLP-------------------G---------------
    Ciclev10010940m                   --------------------D-G------------------------------------D--------V---KE--LC-LDFTVTEE---------------SFG---------------
    Ciclev10027670m                   --------------------E-SMT-------------------------SDNCEEAV-D-LRFRGAPI---ED--LC-LDFTLP-------------------G---------------
    Ciclev10014213m                   --------------------D-A------------------------------------E-------FID--SDG-LG-LTFVREVE---------------ELG---------------
    GRMZM2G034622_T02                 --------------------N-G------------------------------------D--------I---SE--LE-LYFVIVNN---------------EYG---------------
    GRMZM2G124297_T01                 --------------------E-TS--------------------------SEESNPTA-D-LSYKNVKL---ED--LC-LDFTLP-------------------G---------------
    GRMZM2G411536_T03                 ----------------------N------------------------------------D--------I---SDV-LD-LTFSMDADEEK----------LILYE---------------
    GRMZM2G181378_T01                 --------------------D-A------------------------------------A-------EID--N---LY-LTFSRGAH---------------ELG---------------
    GRMZM2G049141_T01                 --------------------D-SCS-------------------------SESQ-KI--D-LCFRGAPV---ED--LY-LDFTLP-------------------G---------------
    GRMZM2G080439_T01                 --------------------D-A------------------------------------A-------EID--N---LY-LTFSRGAH---------------ELG---------------
    GRMZM2G021299_T01                 ----------------------N------------------------------------D--------V---SDL-PY-LTFSMDPDEEK----------HILYE---------------
    GRMZM2G328988_T01                 --------------------E-SNL-------------------------TRDCQ-IS-D-LTYRGCRI---ED--LA-IEFALP-------------------G---------------
    GRMZM2G331368_T02                 ----------------------N------------------------------------D--------I---SDV-LD-LTFSMDADEEK----------LILYE---------------
    GRMZM2G461948_T01                 --------------------D-G------------------------------------D--------V---ED--LF-LDFTVTEE---------------LGG---------------
    GRMZM2G374574_T01                 --------------------E-STS-------------------------GKNQLQVA-D-LCFHGASI---ED--LC-LDFTLP-------------------G---------------
    Carubv10016604m                   --------------------D-G------------------------------------D--------L---KE--LC-LDFTVTEE---------------FCG---------------
    Carubv10011657m                   ----------------------N------------------------------------D--------V---SDI-LD-LTFSMDADEEK----------HILYE---------------
    Carubv10007210m                   --------------------S-R------------------------------------E-------EFD--KAD-LG-LTFSVEIED--------------NLK---------------
    Carubv10003974m                   --------------------E-AVG-------------------------GDNNSTVS-D-LCLRGSRI---ED--LC-LDFTLP-------------------G---------------
    Carubv10012881m                   --------------------K-G------------------------------------D--------I---SE--LE-LYFVILNN---------------EYG---------------
    Carubv10000054m                   --------------------T-ETQ-------------------------GDSLAAKC-D-LSFHGTKI---ED--LC-LEFVLP-------------------G---------------
    Carubv10000186m                   --------------------D-P------------------------------------V-------FFD--SNAGLD-LTFVLETE---------------ELG---------------
    Carubv10025730m                   ----------------------H------------------------------------D--------I---SDV-LD-LTFSIDADEEK----------LILYE---------------
    Bradi2g34820.1                    --------------------H-G------------------------------------D--------L---SD--LE-LYFVIVNN---------------EYG---------------
    Bradi2g37870.1                    --------------------E-TSS-------------------------ERASNPSA-Y-LSYKNVRL---ED--LC-LDFTLP-------------------G---------------
    Bradi2g22927.2                    --------------------E-SCS-------------------------TRDRQSTS-D-LSYRGCRI---ED--LA-IDFAVP-------------------G---------------
    Bradi4g07997.2                    ----------------------N------------------------------------D--------I---SDV-LD-LTFSMDADEEK----------LILYE---------------
    Bradi4g33520.1                    --------------------G-A------------------------------------T-------DID--E---LT-LTFSRDIH---------------TLG---------------
    Bradi1g12340.2                    --------------------E-G------------------------------------D--------V---ED--LC-LDFTLTEE---------------FGG---------------
    Bradi5g04567.1                    --------------------E-SC--------------------------DLNQ-QVR-D-LCFHGSPI---ED--LC-LDFTLP-------------------G---------------
    Bradi3g00350.1                    --------------------E-STS-------------------------GRNQLQVT-D-LCFRGTPV---ED--LC-LDFTLP-------------------G---------------
    Aquca_017_00766.1                 --------------------D-SVS-------------------------VKRS-----D-SYFQNTRI---ED--LC-LDFTLP-------------------G---------------
    Aquca_006_00259.1                 --------------------E-TIS-------------------------GKDHEGIA-D-LRFRGTPI---ED--LC-LDFTLP-------------------G---------------
    Aquca_028_00189.1                 --------------------D-A------------------------------------D-------FLD--SDA-LG-LTFVREVE---------------ELG---------------
    Aquca_027_00123.1                 --------------------H-SV---------------------------SELPFIS-D-SCFRNTRI---ED--LW-LDFTLP-------------------G---------------
    Aquca_007_00539.1                 ----------------------N------------------------------------D--------I---SDV-LD-LTFSMDADEEK----------LILYE---------------
    Aquca_003_00437.1                 -------------------------------------------------------------------------------------------------------YG---------------
    Aquca_019_00105.1                 --------------------E-G------------------------------------D--------V---KE--LS-LDFTVTDE---------------YFG---------------
    MDP0000264736                     --------------------D-A------------------------------------K-------VID--SDA-LM-LTFVREVE---------------ELG---------------
    MDP0000320720                     --------------------E-SVP-------------------------GGSTTLEF-D-SCFRKTQI---ED--LC-LDFTLP-------------------G---------------
    MDP0000142676                     --------------------D-A------------------------------------H-------FID--SDA-LG-LTFVREVE---------------ELG---------------
    MDP0000318443                     ----------------------N------------------------------------D--------I---SDV-LD-LTFSIDADEEK----------LILYE---------------
    MDP0000206447                     ----------------------N------------------------------------D--------V---SDI-PD-LTFSMDADEEK----------HILYE---------------
    MDP0000196216                     --------------------D-G------------------------------------D--------V---EE--LS-LDFTVTEE---------------SLG---------------
    MDP0000186793                     --------------------K-G------------------------------------D--------I---SE--LE-LYFVIVNN---------------EYG---------------
    MDP0000822588                     --------------------E-SIH-------------------------GESTTLKF-D-SCFRKTQI---ED--LC-LDFTLP-------------------G---------------
    MDP0000924418                     --------------------D-G------------------------------------D--------V---EE--LC-LDFTVTEE---------------SFG---------------
    MDP0000320505                     --------------------E-SNG--------------------------DNCDAIA-E-LRFRGASV---DD--LC-LDFTLP-------------------G---------------
    MDP0000307848                     ----------------------N------------------------------------D--------V---SDI-PD-LTFSMDADEEK----------HILYE---------------
    MDP0000301275                     --------------------E-SNG--------------------------DNRDAIV-E-LHLRGVSI---DD--LC-LDFTLP-------------------G---------------
    MDP0000317971                     ----------------------N------------------------------------D--------I---TDV-LD-LTFSIDADEEK----------LILYE---------------
    Bra022201                         --------------------K-G------------------------------------N--------I---AE--LE-LYFVILNN---------------EYG---------------
    Bra028860                         --------------------S-ESQ-------------------------SDVRAAKC-D-LSFRGTKI---ED--LC-LDFSLP-------------------G---------------
    Bra038022                         ----------------------N------------------------------------D--------V---SDI-LD-LTFSMDADEEK----------HILYE---------------
    Bra021231                         --------------------K-G------------------------------------D--------I---SD--LE-LYFVILNN---------------EYG---------------
    Bra005748                         --------------------S-ESQ-------------------------TDARAAKC-D-LSFRGTNI---ED--LC-LEFVLP-------------------G---------------
    Bra000779                         --------------------D-P------------------------------------V-------FFD--SNAGLG-LNFVLETE---------------ELG---------------
    Bra029461                         --------------------D-P------------------------------------A-------FFD--SNAGLG-LTFELETE---------------ELG---------------
    Bra027850                         ----------------------N------------------------------------D--------I---SDV-LD-LTFSVDADEEK----------LILYE---------------
    Bra040685                         --------------------E-G------------------------------------D--------L---QE--LC-LDFTVTEE---------------FCG---------------
    Bra010737                         --------------------E-AGG-------------------------GDNSSGIS-D-LCLRGSRI---ED--LC-LDFTLP-------------------G---------------
    Medtr2g025830.1                   --------------------D-A------------------------------------D-------YID--SDA-LG-LTFSIEVE---------------ELG---------------
    Medtr2g025950.1                   --------------------D-A------------------------------------D-------YID--SDA-LG-LTFSIEVE---------------ELG---------------
    Medtr2g025810.1                   --------------------E-A------------------------------------D-------YID--SDA-LG-LTFSIEVE---------------ELG---------------
    Medtr2g033040.1                   --------------------E-G------------------------------------D--------I---SD--LE-LYFVILNN---------------EYG---------------
    Medtr2g025790.1                   --------------------D-A------------------------------------D-------YID--SDA-LG-LTFSTEVE---------------ELG---------------
    Medtr2g025930.1                   --------------------D----------------------------------------------------------------VE---------------ELG---------------
    Medtr7g100670.1                   --------------------E-SDC-------------------------KENSMLES-D-LTFRNSKI---ED--LC-LDFSLP-------------------G---------------
    Medtr5g066710.1                   ----------------------N------------------------------------D--------I---SDV-LD-LTFSIDADEEK----------LILYE---------------
    Medtr4g073370.1                   --------------------E-SIG-------------------------GGNTGTVS-N-LHYRGAPI---AD--LC-LDFTLP-------------------G---------------
    Medtr4g133120.1                   --------------------D-S------------------------------------D-------FID--SDA-LG-LTFIREVE---------------ELG---------------
    Vocar20002255m                    --------------------D-P------------------------------------D-------QV---EDV-LC-RNFEVQYD---------------FFG---------------
    Vocar20010178m                    ----------------------N------------------------------------D--------I---TDV-LD-LTFTAETD---------------FFG---------------
    Vocar20006334m                    --------------------P-A------------------------------------D------------Q---LR--------------------------S---------------
    Vocar20007555m                    -----------------------------------------------------------D--------I----DGLIF-ENFVWSFQHPNAAAGGANQVATALAGAGAGMLSQAPSTVPD
    Vocar20012583m                    --------------------G-GSG----------------------------------T-LLVDGVSV---ED--LC-ITFVLP-------------------G---------------
    Vocar20003001m                    AAVEGSSSGGGG--------D-GGGPI--------------------------------D--------VDSGSD--LG-LCFVVTDD------------AAAALG---------------
    Vocar20004069m                    --------------------NGP------------------------------------E-------PV---SEV-FG-LTFSVDVD---------------RFG---------------
    Vocar20000780m                    --------------------E-G------------------------------------D--------V---AD--LG-LTFSADTD---------------YLG---------------
    Vocar20004842m                    --------------------P-P------------------------------------D--------L---VDS-LG-LVFQVDME--------------VGFG---------------
    Vocar20014908m                    --------------------P-G-------------------------------------------------AAA-LE-LFHVWHVSTGDE-----------SGG---------------
    Lus10032589                       ----------------------N------------------------------------D--------V---SDI-PD-LTFSMDPDEEK----------HILYE---------------
    Lus10035589                       --------------------D-G------------------------------------D--------V---KE--LL-LDFTVTEE---------------SFG---------------
    Lus10005068                       --------------------E-SSG---------------------------CHDAVA-D-LRFRGANV---ED--LC-LDFTLP-------------------G---------------
    Lus10010493                       --------------------D-A------------------------------------E-------FID--SDA-LG-LTFVREVE---------------ELG---------------
    Lus10027841                       --------------------E-SSG---------------------------CHDAVV-D-LRFRGSNV---ED--LC-LDFTLP-------------------G---------------
    Lus10019908                       --------------------E-SNV-------------------------GDNSSSNF-D-GCFRNTRI---ED--LC-LDFTLP-------------------G---------------
    Lus10032830                       ----------------------N------------------------------------D--------I---SDL-LD-LTFSIDADEEK----------LILYE---------------
    Lus10017098                       --------------------R-G------------------------------------D--------I---SG--LE-LYFVIVNN---------------EYG---------------
    Lus10002605                       ----------------------N------------------------------------D--------I---SDL-LD-LTFSIDADEEK----------LILYE---------------
    Lus10008636                       --------------------D-G------------------------------------D--------V---KE--LL-LDFTVTEE---------------SFG---------------
    Eucgr.A01178.1                    --------------------E-SVH-------------------------GESSTLRN-D-LCFRNIAM---ED--LC-LDFTVP-------------------G---------------
    Eucgr.A01586.1                    --------------------D-G------------------------------------D--------V---KE--LS-LDFTITEE---------------SFG---------------
    Eucgr.B03986.1                    --------------------K-G------------------------------------D--------I---SD--LE-LYFVIVNN---------------EYG---------------
    Eucgr.D01414.1                    --------------------D-A------------------------------------E-------FID--SDA-LG-LTFVREVE---------------ELG---------------
    Eucgr.D01416.1                    --------------------D-P------------------------------------E-------YID--SDA-LG-LTFVREVE---------------ELG---------------
    Eucgr.F02160.1                    ----------------------N------------------------------------D--------I---TDV-LD-LTFSIDADEEK----------LILYE---------------
    Eucgr.I01410.2                    --------------------E-SSS-------------------------DHNRGAIA-D-LHFRGARI---ED--LC-FDFTLP-------------------G---------------
    Pavirv00038038m                   ----------------------N------------------------------------D--------I---SDV-LD-LTFSMDADEEK----------LILYE---------------
    Pavirv00031244m                   --------------------E-STP-------------------------GENQLEVA-D-LRFRGAAI---ED--LC-LDFTLP-------------------G---------------
    Pavirv00010575m                   --------------------N-A------------------------------------A-------EID--D---LY-LTFSRGAH---------------ELG---------------
    Pavirv00004902m                   --------------------D-A------------------------------------S-------LVD--SNI-LE-LTFVREDE---------------VLG---------------
    Pavirv00020428m                   --------------------E-KS--------------------------SRTSNHTA-V-LSYKNVKL---ED--LC-LDFTLP-------------------G---------------
    Pavirv00067430m                   --------------------N-A------------------------------------A-------EID--D---LY-LTFSRGAH---------------ELG---------------
    Pavirv00058663m                   ----------------------N------------------------------------D--------V---SDL-PD-LTFSMDPDEEK----------HILYE---------------
    Pavirv00067620m                   --------------------E-STP-------------------------GENQLEVA-D-LRFRGAAI---ED--MC-LDFTLP-------------------G---------------
    Pavirv00029557m                   ----------------------N------------------------------------D--------I---SDV-LD-LTFSMDADEEK----------LILYE---------------
    Pavirv00023469m                   --------------------N-G------------------------------------D--------I---SE--LE-LYFVIVNN---------------EYG---------------
    Pavirv00024250m                   --------------------E-SNS-------------------------TSDCQIIS-D-LTYRGCRI---ED--LA-IDFTLP-------------------G---------------
    Pavirv00023205m                   --------------------E-SNL-------------------------TRDCQIMS-D-LTYRGCRI---ED--LA-IDFALP-------------------G---------------
    Pavirv00029138m                   --------------------E-TS--------------------------STTSNHTA-V-LSYKNVKL---ED--LC-LDFTLP-------------------G---------------
    LOC_Os03g47949.1                  --------------------E-G------------------------------------D--------V---ED--LC-LDFTLTEE---------------LGG---------------
    LOC_Os02g01170.1                  --------------------E-STY-------------------------GMNQLEVT-D-LRFRGTPI---ED--LC-LDFTLP-------------------G---------------
    LOC_Os09g07900.1                  ----------------------N------------------------------------D--------V---SDI-PD-LTFSMDPDEEK----------HILYE---------------
    LOC_Os12g24080.1                  ----------------------N------------------------------------D--------I---SDV-LD-LSFSMDADEEK----------RILYE---------------
    LOC_Os05g38830.1                  --------------------E-SSS-------------------------SGDCKSTS-D-LSYRGCRI---ED--LA-IEFALP-------------------G---------------
    LOC_Os05g03100.1                  --------------------E-----------------------------ERASNPAA-D-LSYKNVRL---ED--LC-LDFTLP-------------------G---------------
    LOC_Os05g06690.1                  --------------------D-G------------------------------------D--------I---SQ--LE-LYFVIVNN---------------EYG---------------
    PGSC0003DMT400075387              --------------------D-P------------------------------------G-------TVD--QDI-LS-LTFAYDVE---------------ELG---------------
    PGSC0003DMT400021802              --------------------D-P------------------------------------E-------MVD--QDT-LG-LTFVREVE---------------ELG---------------
    PGSC0003DMT400031190              --------------------E-SIK-------------------------DQNLDKSY-D-MRFRGTPV---ED--LC-LDFTLP-------------------G---------------
    PGSC0003DMT400072624              --------------------D-P------------------------------------E-------MVD--GDN-LG-LRFICDVE---------------SMG---------------
    Glyma14g36180.1                   ----------------------N------------------------------------D--------I---SDV-LD-LTFSIDADEEK----------LILYE---------------
    Glyma02g38020.2                   ----------------------N------------------------------------D--------I---SDV-LD-LTFSIDADEEK----------LILYE---------------
    Glyma12g03640.1                   --------------------E-SIG-------------------------GSYTDTFA-N-LHFRGAPI---ED--LC-LDFTLP-------------------G---------------
    Glyma11g11490.1                   --------------------Q-STG-------------------------GSYTDTFA-N-LHFRGAPI---ED--LC-LDFTLP-------------------G---------------
    Glyma06g00600.1                   --------------------E-SIG-------------------------GSYTDTIV-N-LHFHGVPI---ED--LC-LDFTLP-------------------G---------------
    Glyma06g10360.1                   ----------------------N------------------------------------D--------I---SEI-LD-LTFSIDADEEK----------LILYE---------------
    Glyma04g00530.1                   --------------------E-SIG-------------------------GSYTDTIV-N-LYFHGAPI---ED--LC-LDFTLP-------------------G---------------
    Glyma04g10481.1                   ----------------------N------------------------------------D--------I---SDV-LD-LTFSIDADEEK----------LILYE---------------
    Glyma08g09270.3                   ----------------------N------------------------------------D--------V---SDI-PD-LTFSMDADEEK----------HILYE---------------
    Glyma17g01210.2                   --------------------D-A------------------------------------D-------FID--SDA-LG-LTFVREVE---------------ELG---------------
    Glyma17g04180.1                   --------------------K-G------------------------------------D--------I---SE--LE-LYFVIVNN---------------EYG---------------
    Glyma13g19981.1                   --------------------E-SVS-------------------------GGNSELQH-G-LSFRDTRI---ED--LC-LDFTLP-------------------G---------------
    Glyma05g26360.1                   ----------------------N------------------------------------D--------V---SDI-PD-LTFSMDADEEK----------HILYE---------------
    Glyma19g37310.1                   --------------------D-G------------------------------------D--------V---KE--LS-LDFTVTEE---------------SLG---------------
    Glyma15g14591.1                   --------------------E-R------------------------------------D--------I---SE--LE-LYFVIVNN---------------EYG---------------
    Glyma03g34650.2                   --------------------D-G------------------------------------D--------V---KE--LS-IDFTVTEE---------------SLG---------------
    Glyma10g05620.3                   --------------------E-SVS-------------------------GGNSELQY-G-LSFRDMSI---ED--LC-LDFTLP-------------------G---------------
    Glyma07g36390.1                   --------------------K-G------------------------------------D--------I---SE--LE-LYFVIVNN---------------EYG---------------
    Glyma07g39546.1                   --------------------D-A------------------------------------D-------FID--SDS-LG-LTFVREVE---------------ELG---------------
    Gorai.010G033100.1                ----------------------N------------------------------------D--------I---SDV-LD-LTFSIDADEEK----------LILYE---------------
    Gorai.010G186800.1                --------------------D-A------------------------------------E-------FID--SDA-LG-LTFVREVE---------------ELG---------------
    Gorai.009G278900.1                ----------------------N------------------------------------D--------I---SDV-LD-LTFSIDADEEK----------LILYE---------------
    Gorai.009G228200.1                ----------------------N------------------------------------D--------I---SDV-LG-LTFSIDADEEK----------LILYE---------------
    Gorai.009G183200.1                --------------------D-A------------------------------------E-------FID--SDA-LG-LTFVREVE---------------ELG---------------
    Gorai.009G420400.1                --------------------E-SIC-------------------------VENAALKQ-D-LCFRNTRI---ED--LY-LDFTLP-------------------G---------------
    Gorai.002G100900.1                ----------------------N------------------------------------D--------V---SDI-PD-LTFSMDADEEK----------HILYE---------------
    Gorai.002G196900.1                --------------------E-STG-------------------------GESSAAVD-E-LRFRGTPI---ED--LC-LDFTLP-------------------G---------------
    Gorai.002G245000.1                --------------------E-G------------------------------------N--------V---ED--LC-LDFTVTEE---------------SFG---------------
    Gorai.002G003200.1                --------------------E-LMG-------------------------DDSGDAIA-D-LRFRGAPI---ED--LC-LDFTLP-------------------G---------------
    Gorai.011G204200.1                --------------------K-G------------------------------------D--------I---SE--LE-LYFVIVNN---------------EYG---------------
    Gorai.008G035900.1                --------------------D-SLG-------------------------GDNSDAIP-D-LRFRGASI---ED--LC-LDFTLP-------------------G---------------
    Gorai.006G265700.1                --------------------K-E------------------------------------D--------I---SG--LE-LYFVIVNN---------------EYG---------------
    Potri.010G150000.3                --------------------Q-G------------------------------------D--------I---SD--LE-LYFVIVNN---------------EYG---------------
    Potri.009G134300.1                --------------------E-SIG-------------------------SDH-EAIA-D-LHFHGTPI---ED--LC-LDFTLP-------------------G---------------
    Potri.004G174700.1                --------------------E-SIS-------------------------AENNEVNA-D-LCFRGTPI---KD--LC-LDFTLP-------------------G---------------
    Potri.011G094100.1                ----------------------N------------------------------------D--------V---SDI-PD-LTFSMDADEEK----------HILYE---------------
    Potri.006G132000.1                --------------------G-SAF-------------------------GENSSSAL-D-ACFWNTKI---ED--LY-LDFTLP-------------------G---------------
    Potri.006G011700.1                --------------------D-P------------------------------------E-------FID--SDA-LG-LTFVREVE---------------ELG---------------
    Potri.016G085200.3                --------------------G-LVI-------------------------VENSSSTQ-D-ACFWNTRI---ED--LC-LDFTLP-------------------G---------------
    Potri.016G096500.1                --------------------D-G------------------------------------D--------V---KD--LS-LDFTVTEE---------------LFG---------------
    Potri.016G012900.1                --------------------D-P------------------------------------E-------FID--SDA-LS-LTFVQEVE---------------ELG---------------
    Potri.002G110500.1                ----------------------N------------------------------------D--------I---SDV-LD-LTFSIDADEEK----------LILYE---------------
    Potri.008G101300.1                --------------------Q-G------------------------------------D--------I---SD--LE-LYFVIVNN---------------EYG---------------
    Potri.001G368600.1                ----------------------N------------------------------------D--------V---SCV-PD-LTFSMDADEEK----------HILYE---------------
    Phvul.003G084200.1                --------------------D-A------------------------------------D-------FID--SDA-LG-LTFVREVE---------------ELG---------------
    Phvul.003G118500.1                --------------------K-G------------------------------------D--------I---SE--LE-LYFVIVNN---------------EYG---------------
    Phvul.009G119700.1                --------------------E-SIG-------------------------GSYTDTLF-N-LHFHGAPI---ED--LC-LDFTLP-------------------G---------------
    Phvul.009G034900.1                ----------------------N------------------------------------D--------I---SDV-LD-LTFSIDADEEK----------LILYE---------------
    Phvul.011G035200.1                --------------------E-SFG-------------------------GCYTDTIG-N-LHFRGAPI---ED--LC-LDFTLP-------------------G---------------
    Phvul.008G183200.1                ----------------------N------------------------------------D--------I---SDV-LD-LTFSIDADEEK----------LILYE---------------
    Phvul.007G163300.1                --------------------E-SVN-------------------------GGNSELQQCG-LTFRDTRI---ED--LC-LDFTLP-------------------G---------------
    Phvul.007G163400.1                --------------------E-SIS-------------------------GGNSDI-----VSFRDERI---ED--LF-LDFTLP-------------------G---------------
    Phvul.001G184300.1                --------------------D-G------------------------------------D--------V---ME--LC-LDFTVTEE---------------SLG---------------
    Phvul.006G120900.1                --------------------E-G------------------------------------D--------I---SE--LE-LYFVIVNN---------------EYG---------------
    Phvul.006G142800.1                --------------------D-A------------------------------------D-------FID--TDA-LG-LTFVRELE---------------ELG---------------
    Phvul.002G189700.1                ----------------------N------------------------------------D--------V---SDV-PD-LTFSMDADEEK----------HILYE---------------
    mrna26562.1-v1.0-hybrid           ------------------------------------------------------------------------------------------------------------------------
[truncated: 982,315 more chars]
