# Supplementary material for: Genome-Wide Identification and Evolution of HECT Genes in Soybean
Source: Int J Mol Sci. 2015 Apr 16;16(4):8517–35. doi: 10.3390/ijms16048517 (PMC4425094; doi:10.3390/ijms16048517)
Supplement: Supplementary file 1 [file ijms-16-08517-s001.zip › ijms-80517-Supplementary Information/ijms-80517-supplementary figures.pdf]

Supplementary Information

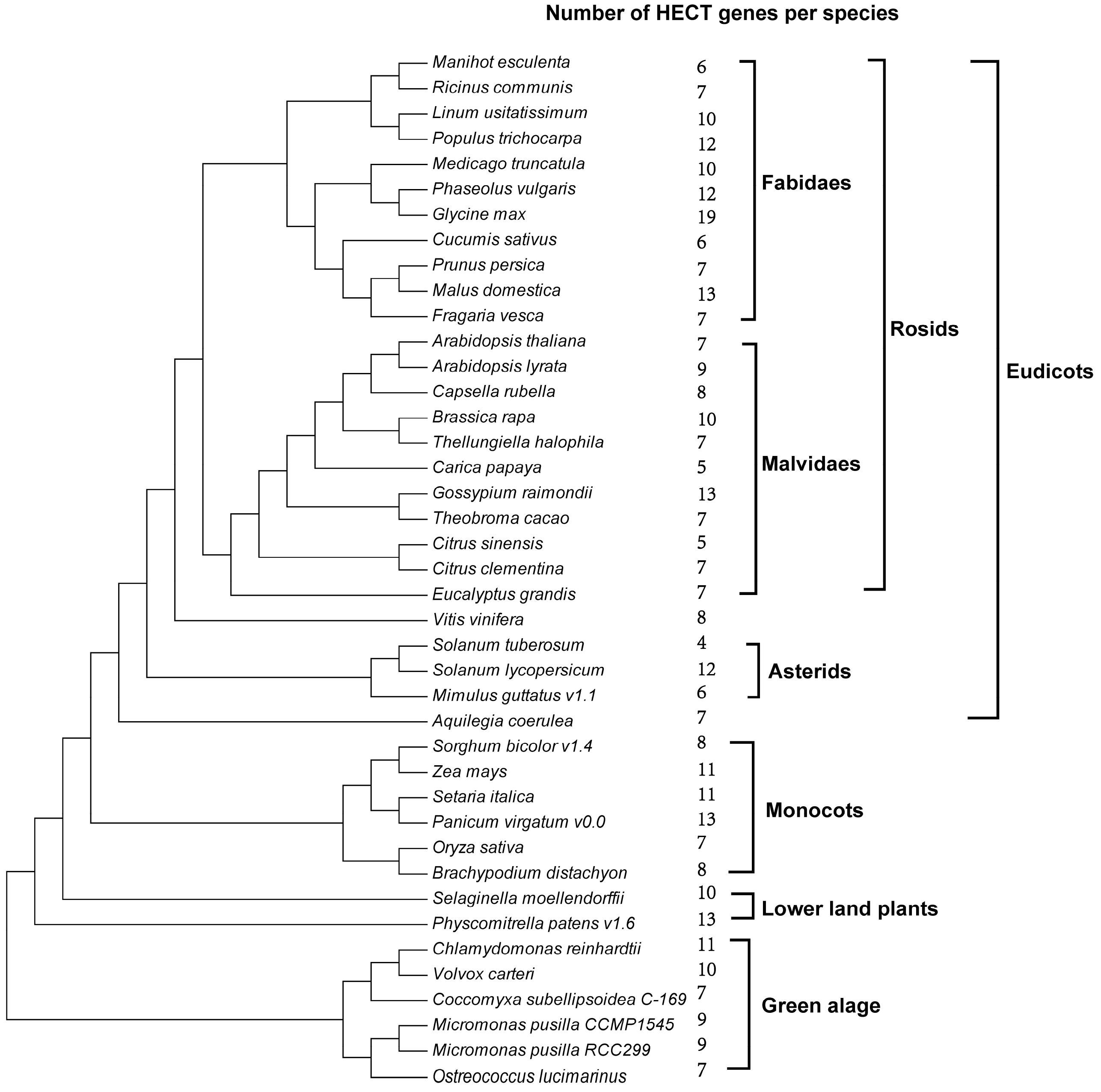

Figure S1. Number of HECT genes per plant genome. The HECT gene numbers per species are given based on the 41-species tree in phytozome v9.1.

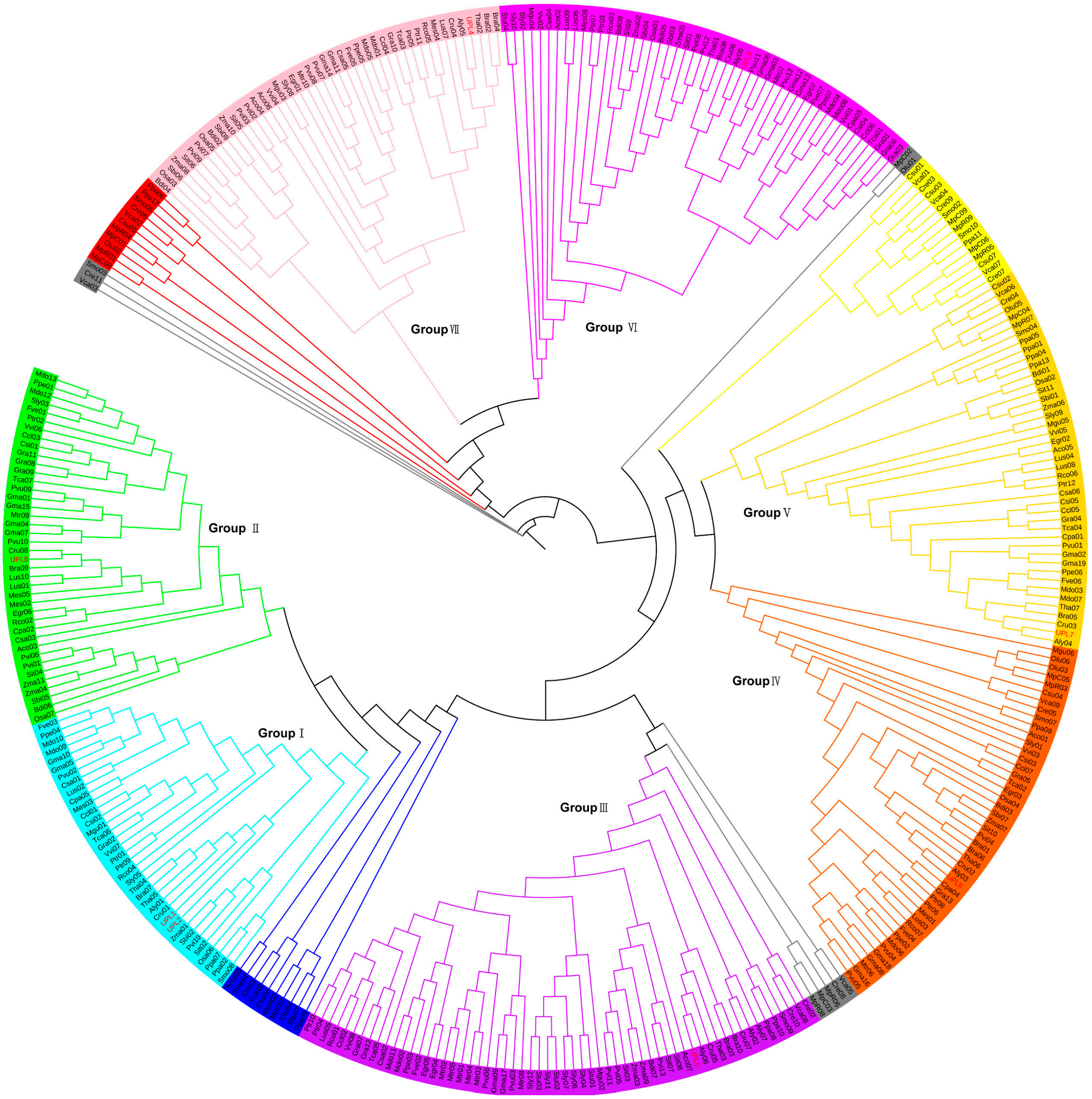

Figure S2. Phylogenetic relationships of 365 plant HECT genes. The maximum likelihood tree constructed from 365 plant HECT domains is shown.

Group Ⅰ

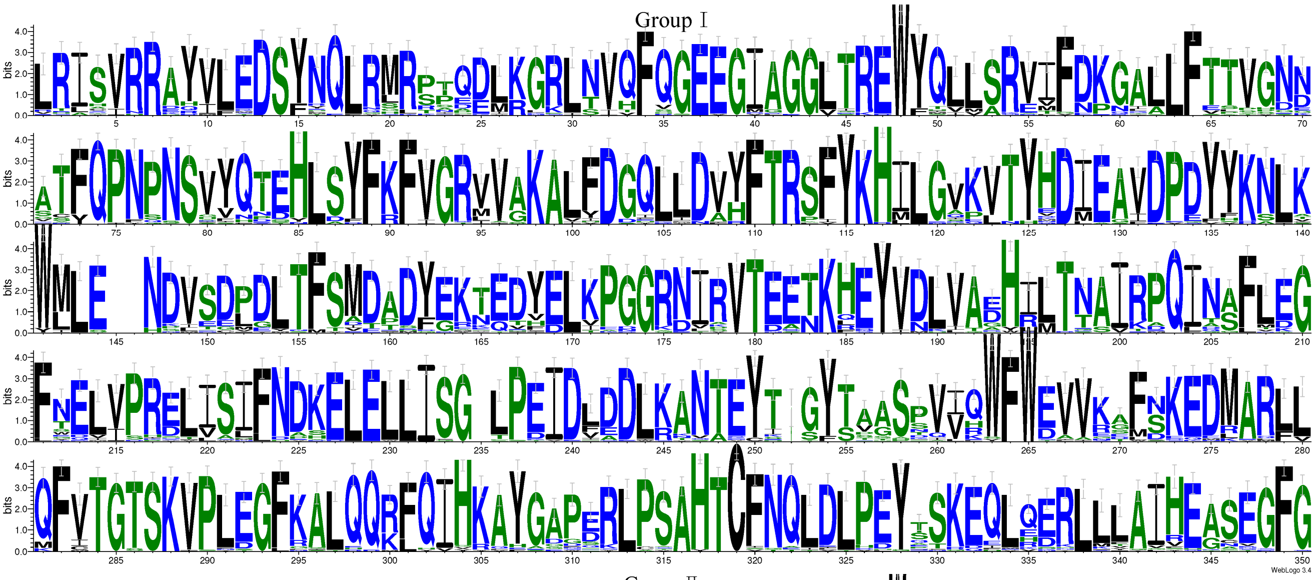

Group Ⅱ

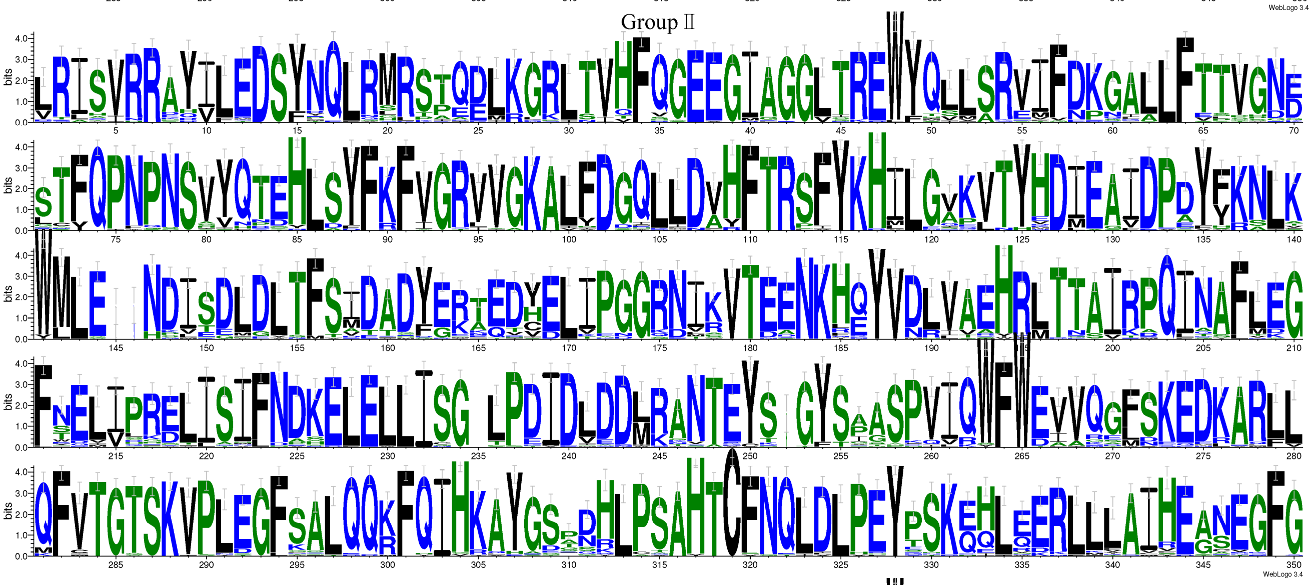

Group Ⅲ

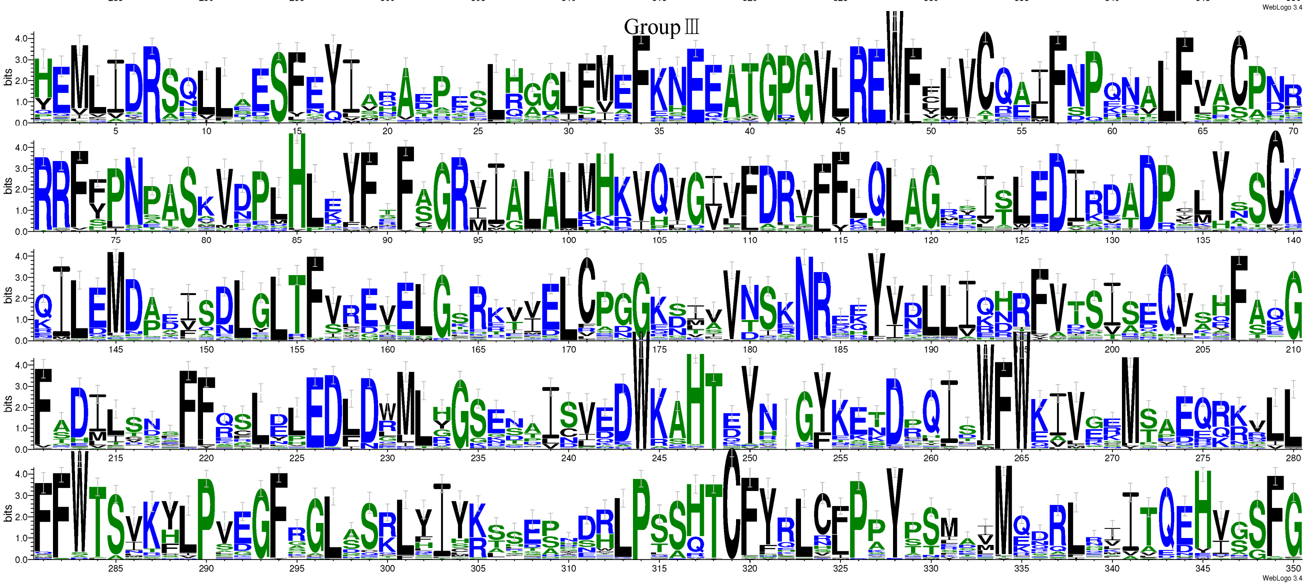

Figure S3. *Cont.*

Group Ⅳ

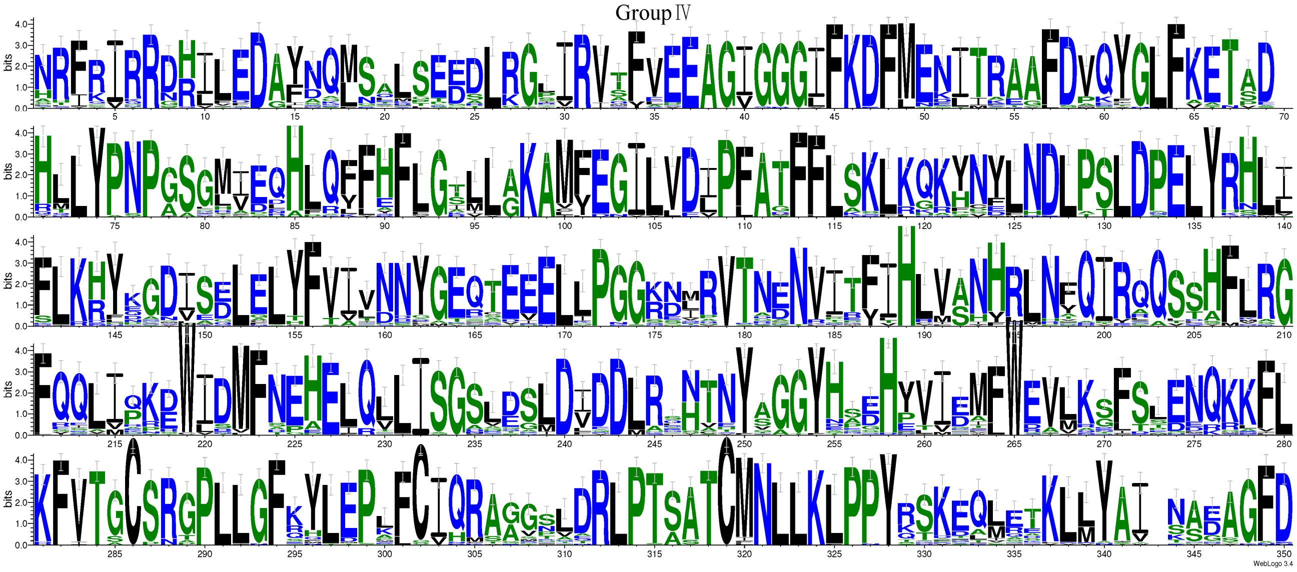

Group Ⅴ

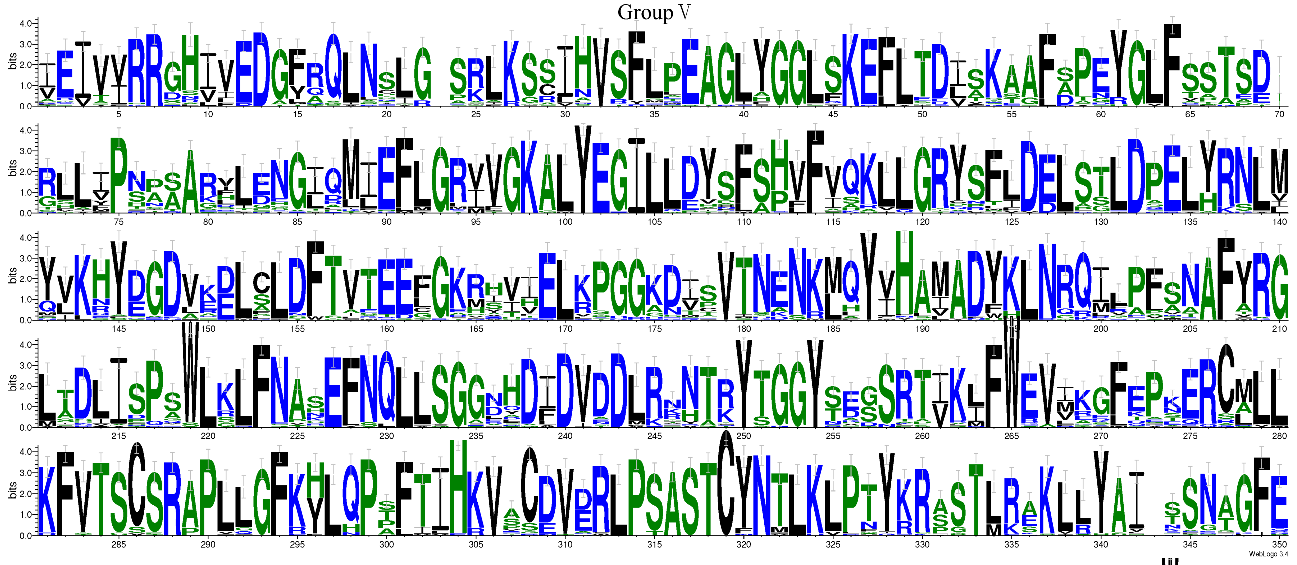

Group Ⅵ

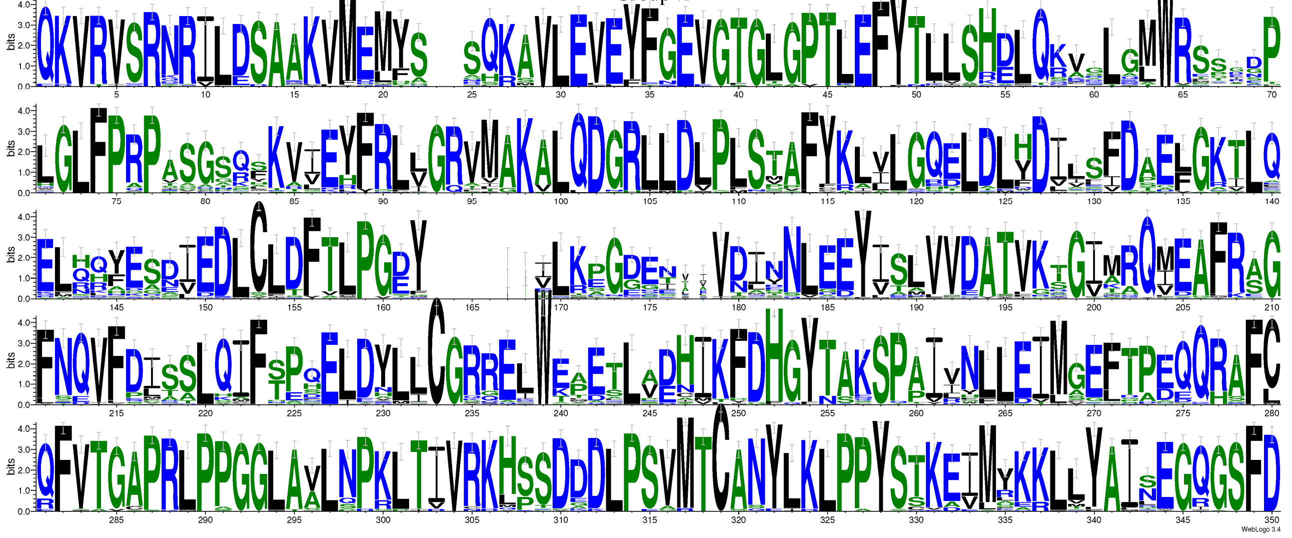

Figure S3. *Cont.*

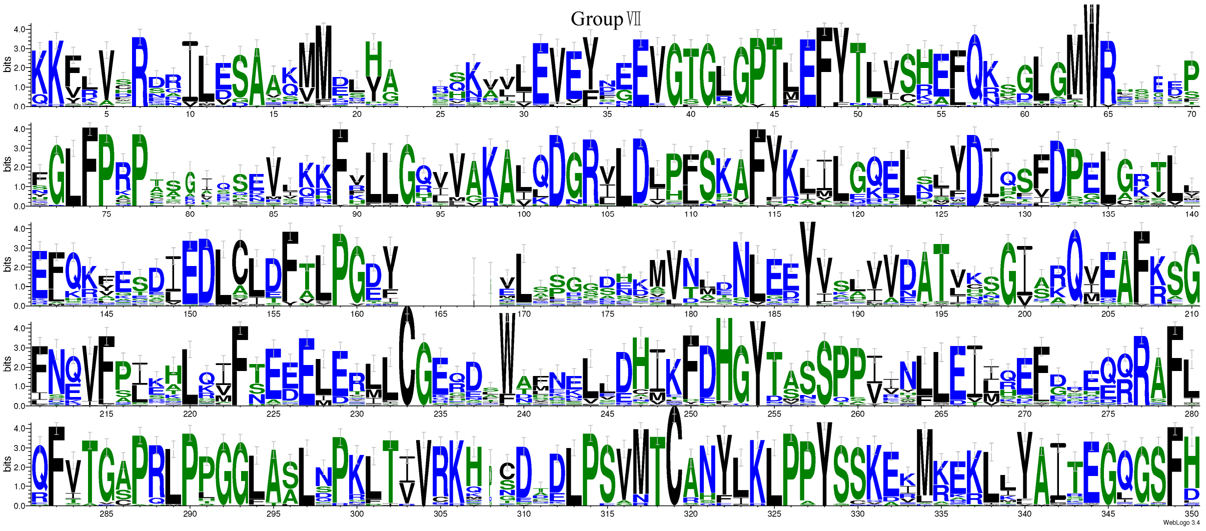

Figure S3. Logo representations of highly conserved residues of HECT domains in seven plant HECT gene groups.

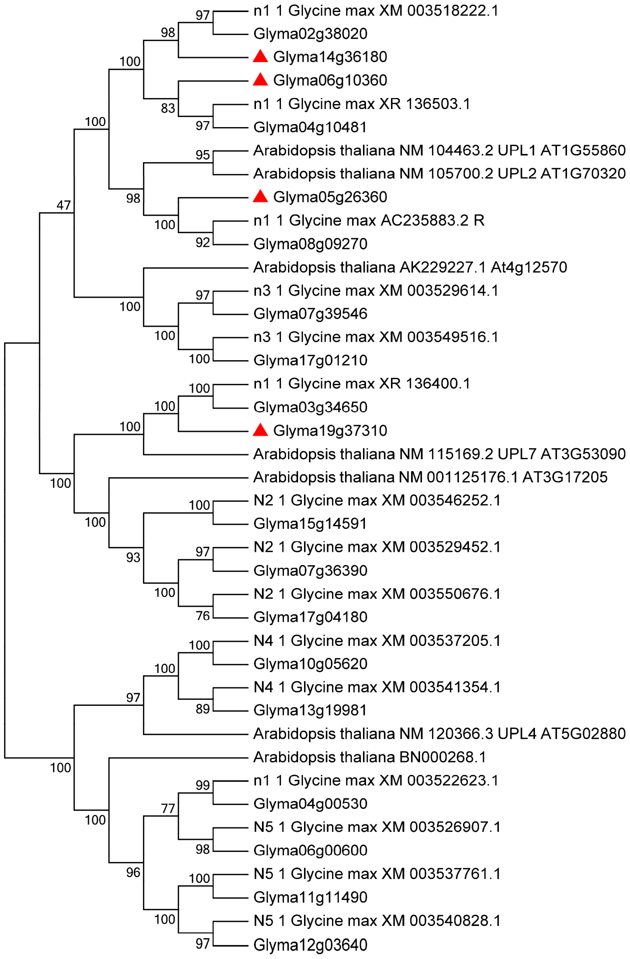

Figure S4. Comparison of soybean HECT genes with previous study by using phylogenetic relationships. The tree was constructed from multiple sequence alignments of the 15 soybean HECT genes in Marin's study and the 19 HECT soybean genes in this study with seven *Arabidopsis thaliana* HECT genes as outgroups by using MEGA6 with neighbor-joining method. The red triangles in the figure represent the four newly annotated genes.

File S1. Multiple sequence alignments of the 365 plant HECT domains filtered by trimal v1.4 with gappyout option.

File S2. FASTA format multiple sequence alignments of the full-length HECT proteins in *Arabidopsis thaliana*, *Glycine max*, *Medicago truncatula*, and *Phaseolus vulgaris*. MAFFT program [42] was used to align the full-length amino acid sequences of the four plant HECT genes.

File S3. FASTA format multiple sequence alignments of 19 full-length HECT proteins in soybean. MAFFT program [42] was used to align the full-length amino acid sequences of the 19 soybean HECT genes.
